# Supplementary material for: A loss-of-function mutation in human Oxidation Resistance 1 disrupts the spatial–temporal regulation of histone arginine methylation in neurodevelopment
Source: Genome Biol. 2023 Sep 29;24:216. doi: 10.1186/s13059-023-03037-1 (PMC10540402; doi:10.1186/s13059-023-03037-1)
Supplement: Supplementary file 3 — Additional file 3. Antibody List. [file 13059_2023_3037_MOESM3_ESM.pdf]

Antibodies	Working condition	Sourse,RRID
anti-ACTIN-B (AC-15)	1:2000	(Santa Cruz Biotechnology Cat# sc-69879, RRID:AB_1119529)
anti-ACTIN-B	1:2000	(Abcam Cat# ab8227, RRID:AB_2305186)
anti-GAPDH-HRP	1:5000	(Abcam Cat# ab9482, RRID:AB_307272)
anti-Histone H3 antibody - Nuclear Loading	1:2000	(Abcam Cat# ab1791, RRID:AB_302613)
anti-Histone H3R2 Dimethyl Symmetric (H3R2me2s)	1:1000 in WB and 1:200 in IF	EpiGentek, Cat. A-3705-100
anti-Histone H4 (symmetric di methyl R3) antibody - ChIP Grade	1:1000	(Abcam Cat# ab5823, RRID:AB_10562795)
anti-OXR1	1:1000 in WB and 1:200 in IF& PLA	(Bethyl Cat# A302-035A, RRID:AB_1576567)
anti-OXR1	3ug/1mg pre-clear cell lysate in IP	(Bethyl Cat# A302-036A, RRID:AB_1576565)
anti-PRMT1	1:1000 in WB	(Cell Signaling Technology Cat# 2449, RRID:AB_2237696)
Anti-PRMT1 antibody [PRMT1-171]	1:200 in PLA	(Abcam Cat# ab12189, RRID:AB_298919)
anti-PRMT5	1:1000 in WB and 1:200 in PLA	(Thermo Fisher Scientific Cat# MA5-31465, RRID:AB_2787097)
anti-PRMT5	1:1000 in WB	(Cell Signaling Technology Cat# 2252, RRID:AB_10694541)
IgG-HRP anti-mouse	1:10000	(Sigma-Aldrich Cat# A9044, RRID:AB_258431)
IgG-HRP anti-rabbit	1:10000	(Abcam Cat# ab6721, RRID:AB_955447)
Alexa Fluor® 488 Donkey anti-Goat IgG (H+L) Cross-Adsorbed Secondary Antibody	1:500	(Thermo Fisher Scientific Cat# A-11055, RRID:AB_2534102)
Alexa Fluor® 594 Donkey Anti-Mouse IgG (H+L) highly Cross-Adsorbed Secondary Antibody	1:500	(Thermo Fisher Scientific Cat# A-21203, RRID:AB_141633)
Alexa Fluor® 594 Donkey anti-Rabbit IgG (H+L) Highly Cross-Adsorbed Secondary Antibody	1:500	(Thermo Fisher Scientific Cat# A-21207, RRID:AB_141637)
Alexa Fluor®488 Goat Anti-Rabbit IgG (H+L) Cross-Adsorbed Secondary Antibody	1:500	(Thermo Fisher Scientific Cat# A-11034, RRID:AB_2576217)
Alex Fluor® 488 Goat anti-Rat IgG (H+L) Cross-Adsorbed Secondary Antibody	1:500	(Thermo Fisher Scientific Cat# A-11006, RRID:AB_2534074)
Alexa Fluor ® 488 Goat anti-Mouse IgG (H+L) highly Cross-Adsorbed Secondary Antibody	1:500	(Thermo Fisher Scientific Cat# A-11029, RRID:AB_2534088)
Alexa Fluor® 555 Goat anti-Guinea Pig IgG (H+L) Highly Cross-Adsorbed Secondary Antibody	1:500	(Thermo Fisher Scientific Cat# A-21435, RRID:AB_2535856)
Alexa Fluor®594 Goat Anti-Mouse IgG (H+L) Cross-Adsorbed Secondary Antibody	1:500	(Thermo Fisher Scientific Cat# A-11032, RRID:AB_2534091)
Alexa Fluor®594 Goat Anti-rabbit IgG (H+L) Cross-Adsorbed Secondary Antibody	1:500	(Thermo Fisher Scientific Cat# A-11037, RRID:AB_2534095)
anti-P21 (F5)	1:2000	(Santa Cruz Biotechnology Cat# sc-6246, RRID:AB_628073)
anti-HO-1	1:1000	(Santa Cruz Biotechnology Cat# sc-10789, RRID:AB_648281)
anti-Caspase9	1:1000	(Cell Signaling Technology Cat# 9502, RRID:AB_2068621)
anti-Neuron-specific beta -III Tubulin Antibody	1:400 in IF of planar neural differenciat	(R and D Systems Cat# MAB1195, RRID:AB_357520)
anti-Doublecortin (DCX)	1:1000	(Abcam Cat# ab18723, RRID:AB_732011)
anti-MAP2	1:1000	(Abcam Cat# ab5392, RRID:AB_2138153)
anti-SOX2	1:200	(Cell Signaling Technology Cat# 3579, RRID:AB_2195767)
anti-SOX2	1:1000 in IF	(Abcam Cat# ab97959, RRID:AB_2341193)
anti-OCT4	1:200	(Cell Signaling Technology Cat# 2840, RRID:AB_2167691)
anti-NANOG	1:200	(Cell Signaling Technology Cat# 4903, RRID:AB_10559205)
anti-SSEA4	1:200	(Cell Signaling Technology Cat# 4755, RRID:AB_1264259)
Anti-human Sox17	1:250	(R and D Systems Cat# AF1924, RRID:AB_355060)
Anti-human/mouse Brachuary	1:250	(R and D Systems Cat# AF2085, RRID:AB_2200235)
Anti-human CD184 (CXCR4)	1:200	Stemcell Technologies, Cat. 60089
Human HNF-3 beta /FoxA2 Antibody	1:300	(R and D Systems Cat# AF2400, RRID:AB_2294104)
Anti-Tyrosine Hydroxylase Antibody	1:300	(Millipore Cat# AB152, RRID:AB_390204)
anti-KI67	1:300	(Thermo Fisher Scientific Cat# 14-5698-80, RRID:AB_10853185)
anti-cCAS3	1:300	(Cell Signaling Technology Cat# 9661, RRID:AB_2341188)
Anti-Ctip2 antibody [25B6]	1:200	(Abcam Cat# ab18465, RRID:AB_2064130)
anti-TBR1	1;300	(Abcam Cat# ab31940, RRID:AB_2200219)
anti-REELIN	1:200	(Millipore Cat# MAB5366, RRID:AB_2285132)
anti-Nestin	1:200	(BD Biosciences Cat# 611658, RRID:AB_399176)
anti-POMC	1:250	(Phoenix Pharmaceuticals Cat# H-029-30, RRID:AB_2307442)
anti-OTP	1:300	(Takara bio via AH diagnostics AS, Cat.M195-TAK)
EN-2 Antibody (1E1)	1:50	(Santa Cruz Biotechnology, Cat. sc-293311)
anti-NeuN	1:500	(Abcam Cat# ab104224, RRID:AB_10711040)
c-Myc (D84C12)	1:1000	(Cell Signaling Technology Cat# 5605, RRID:AB_1903938)
anti IgG antibody	1ug/1mg pre-clear cell lysate in IP	(Sigma-Aldrich Cat# I5006, RRID:AB_1163659)
anti-PRMT2 antibody [N1C2]	1:1000	(GeneTex Cat# GTX103749, RRID:AB_1951450)
anti-PRMT3	1:1000	(Thermo Fisher Scientific Cat# 730020, RRID:AB_2532823)
abti-PRMT4/CARM1 (3H2) Mouse mAb	1:1000	(Cell Signaling Technology Cat# 12495, RRID:AB_2797935)
Anti-PRMT6 antibody [2C3-F1-G1]	1:1000	Abcam, Cat. Ab151191
anti-PRMT7	1: 500	(GeneTex , Cat. GTX116570
anti-PRMT8	1ug/mL	(Abcam, Cat. Ab168134)
anti PRMT9 (FBXO11 Monoclonal Antibody [4C12])	1ug/mL	Epigentek , Cat. Cat. A-3112-100
Histone H3R2 Dimethyl symmetric (H3R2me2s) Polyclonal Antibody	1:1000 in WB,1:200 in IF	Epigentek, Cat. A-3705-100
Histone H3R2 Dimethyl Asymmetric (H3R2me2a) Polyclonal Antibody	1:1000 in WB,1:500 in IF	Epigentek, Cat. A-3714-050
Histone H3R8 Dimethyl Symmetric (H3R8me2s) Polyclonal Antibody	1:1000	Epigentek, Cat. c10016-A-3706-050
Histone H3R8 Dimethyl Asymmetric (H3R8me2a) Polyclonal Antibody	1:1000	Epigentek, Cat. c10016-A-3716-050
Histone H3R17 Dimethyl Asymmetric (H3R17me2a) Polyclonal Antibody	1:1000	Epigentek, Cat. c10017-A-3709
Histone H3R17 Dimethyl symmetric (H3R17me2s) Polyclonal Antibody	1:1000	Epigentek, Cat. 10017-A-3711
Histone H3R26Dimethyl Asymmetric (H3R26me2a) Polyclonal Antibody	1:1000	Epigentek, Cat. c10018-A-3707
Histone H3R26 Dimethyl symmetric (H3R26me2s) Polyclonal Antibody	1:1000	Epigentek, Cat. c10018-A-3712
Anti-Histone H4 (asymmetric di methyl R3) antibody - ChIP Grade	1:1000	(Active Motif Cat# 39705, RRID:AB_2793313)
Anti-Histone H4 (symmetric di methyl R3) antibody - ChIP Grade	1:1000 in WB,1:300 in IF	(Abcam Cat# ab5823, RRID:AB_10562795)
Tri-Methyl-Histone H3 (Lys4) Antibody	1:1000 in WB,1:500 in IF	(Cell Signaling Technology Cat# 9727, RRID:AB_561095)
Histone H3K27ac antibody (pAb)	1:2000	(Active Motif Cat# 39133, RRID:AB_2561016)
Histone H3K27me3 antibody (pAb)	1:2000	(Active Motif Cat# 39155, RRID:AB_2561020)
H3K9me3 polyclonal antibody - Classic (50 µg/33 µl)	1:1000	Diagenode, Cat. C15410056
