# Supplementary material for: A loss-of-function mutation in human Oxidation Resistance 1 disrupts the spatial–temporal regulation of histone arginine methylation in neurodevelopment
Source: Genome Biol. 2023 Sep 29;24:216. doi: 10.1186/s13059-023-03037-1 (PMC10540402; doi:10.1186/s13059-023-03037-1)
Supplement: Supplementary file 4 — Additional file 4. Uncropped gel and microscopy images. [file 13059_2023_3037_MOESM4_ESM.zip › Gel_Microscopy_images_GenomeBiology/gel_images.pdf]

Figure.1e

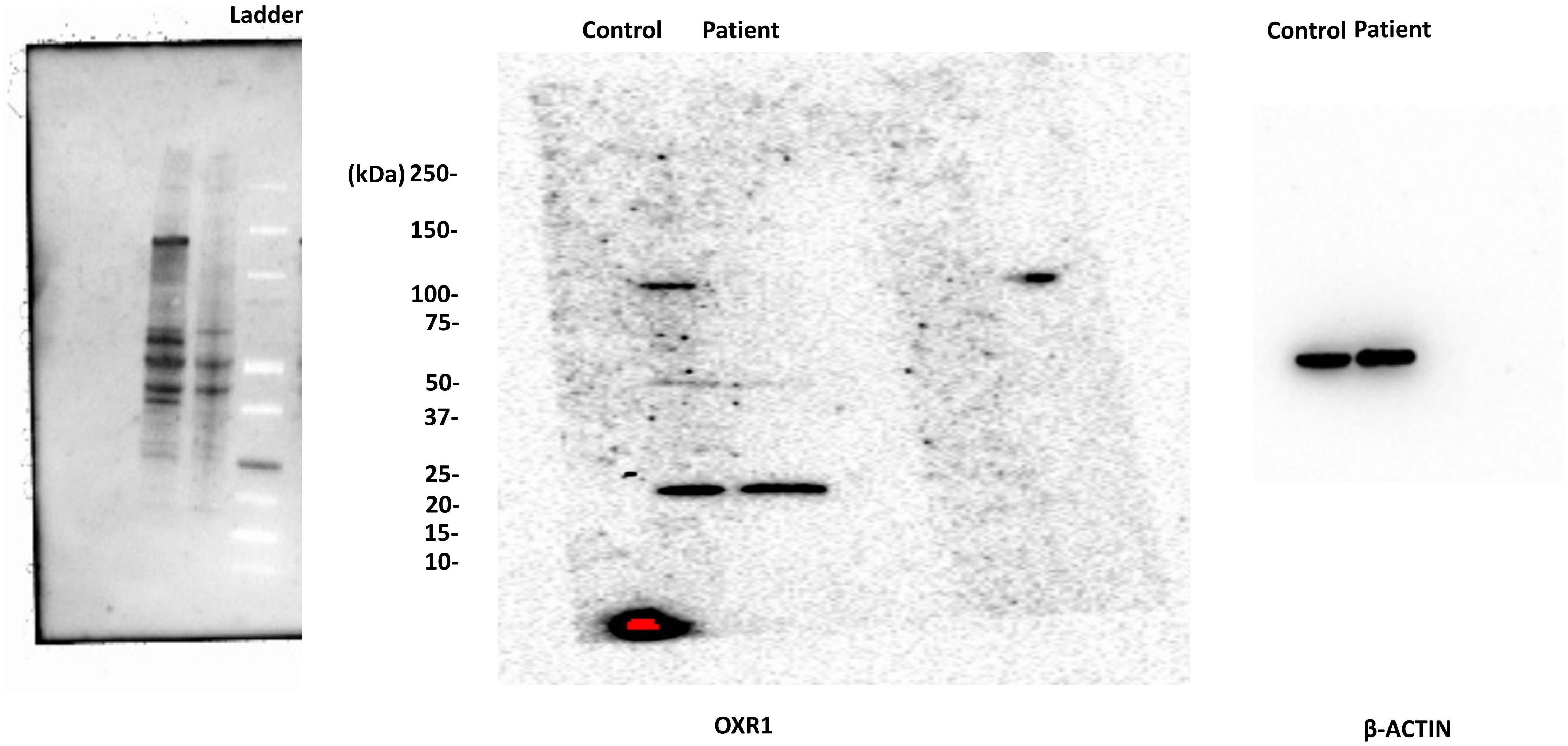
Ladder
Control
Patient
(kDa) 250-
150-
100-
75-
50-
37-
25-
20-
15-
10-
OXR1
Control
Patient
β-ACTIN

Figure.s1

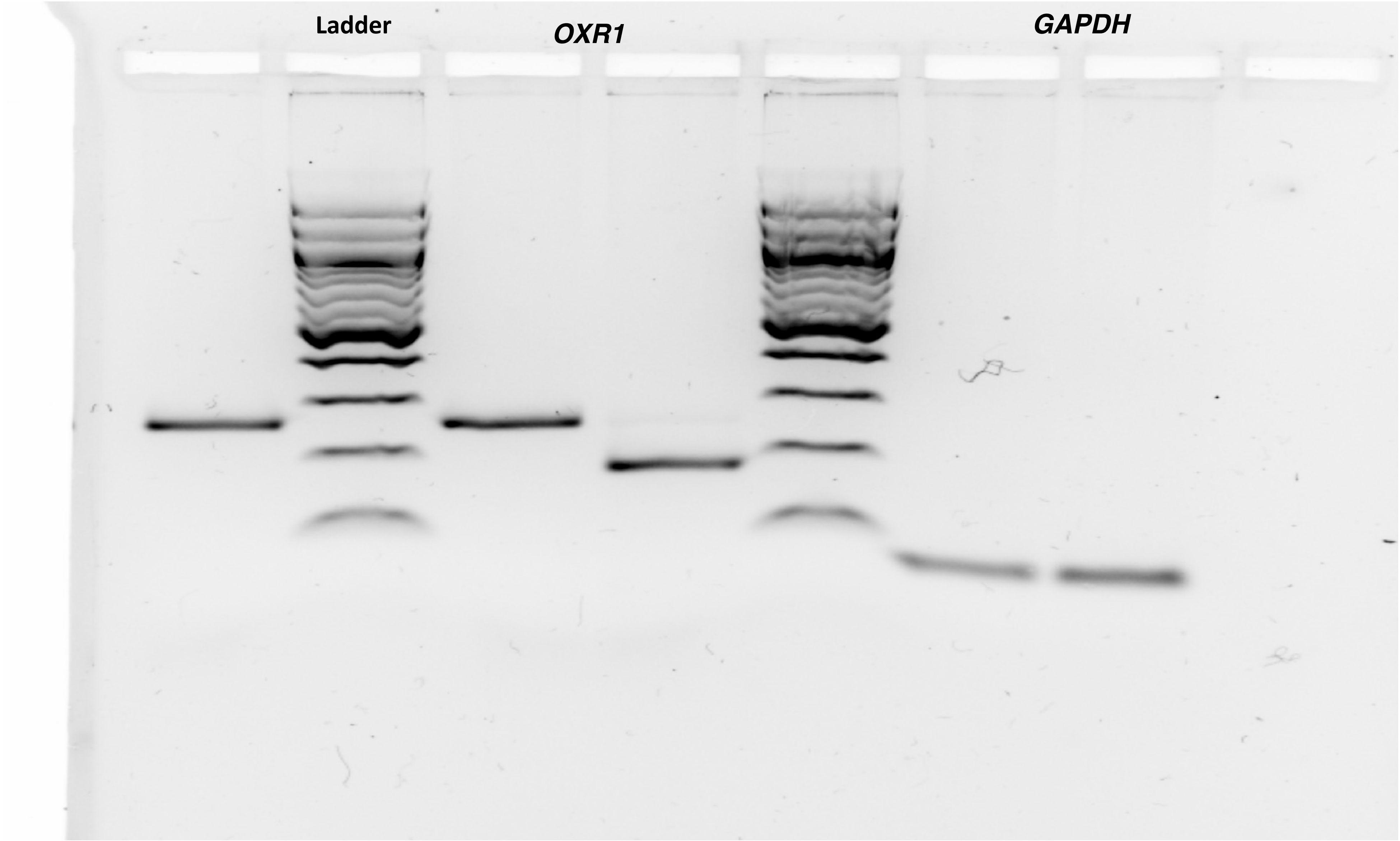

Figure.2d

P21

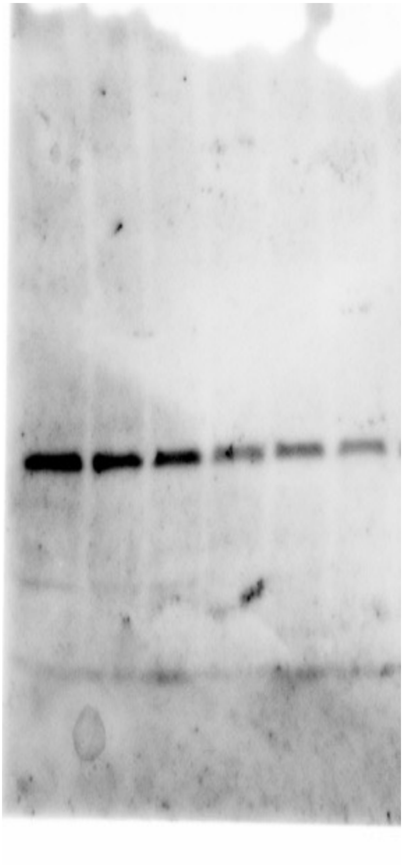

Ho-1

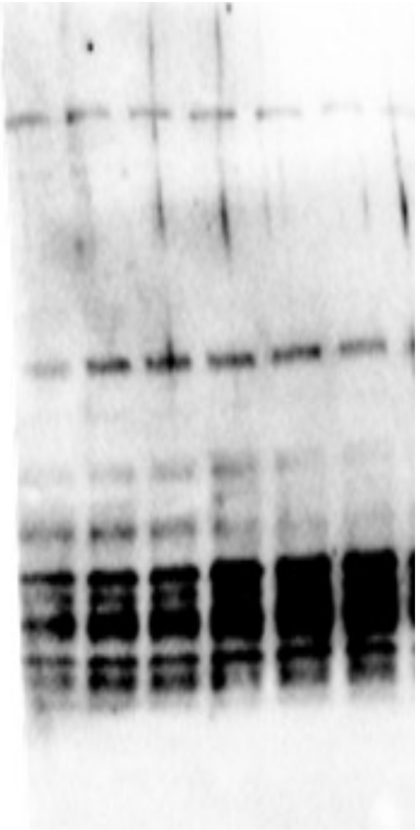

Figure.2g

Caspase-9

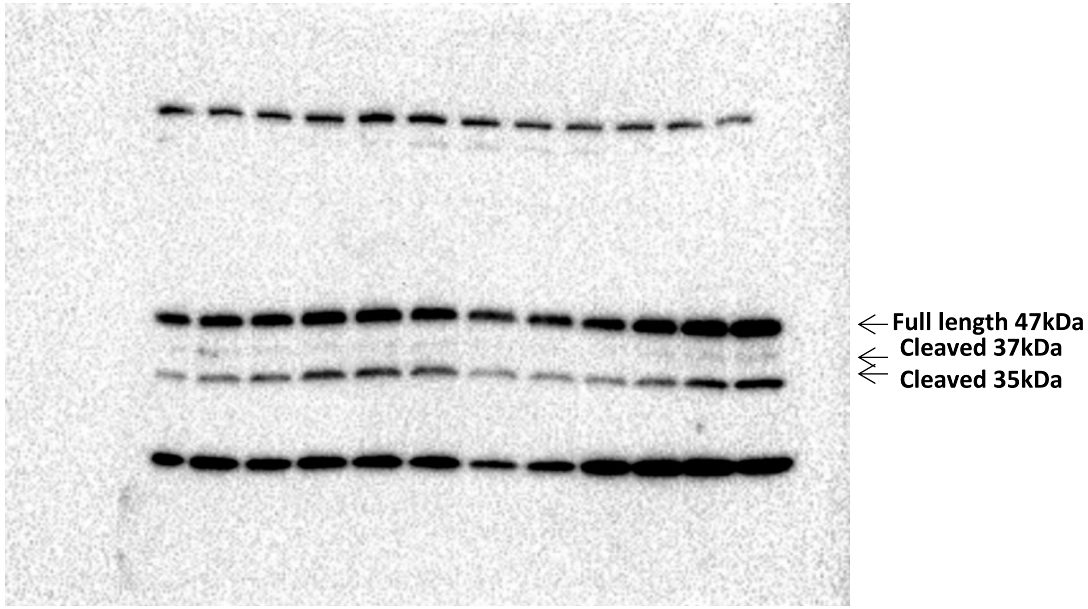

β-actin

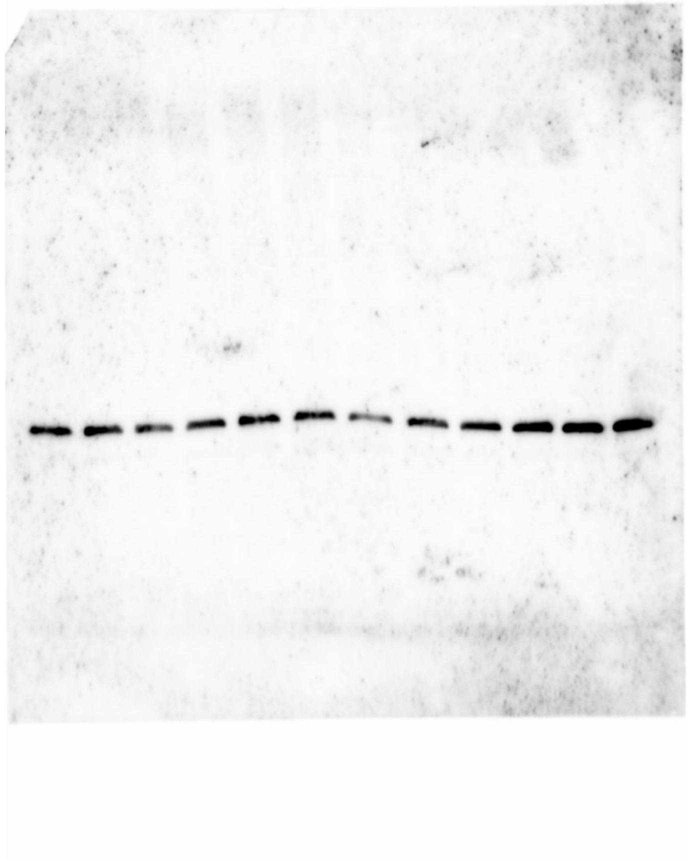

Figure.s3a

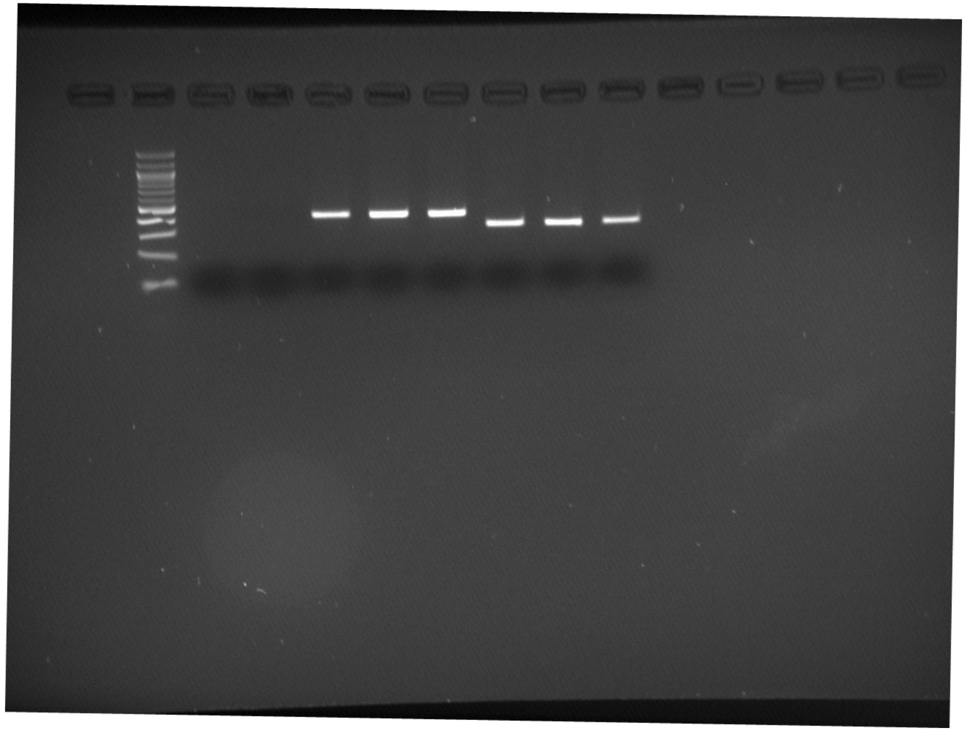

Figure.s3b

1 2 3 4 5 6 7 8 9 10 11 12 13 14 15 16 17 18 19 20 21 22 23 24 25 26 27 28 29 30
JAN FEB MAR APR MAY JUN JUL AUG SEP OCT NOV DEC 20

Feb 23 2016

β-Actin
For Feb 18. 2016

1 antibody 1:5000

2nd antibody 1:1000
Anti mouse.

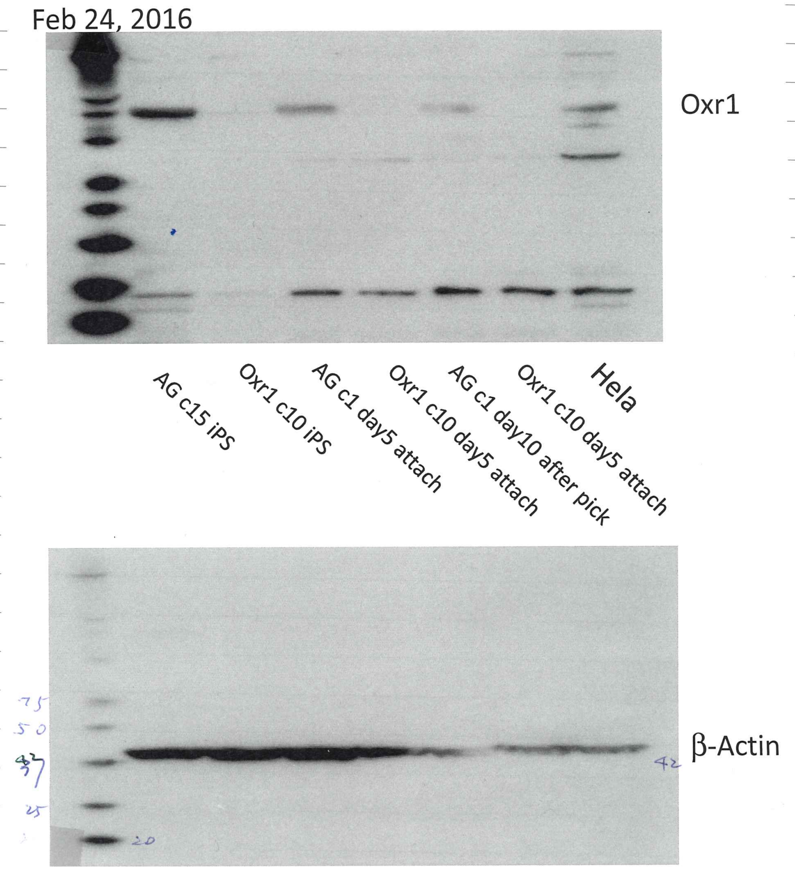

Figure.s3i

08 03. 2016

U U U U U U U U U U
M 3 5 22 30A 30B 32 41 Hela H2O

reused 1st antibody 10% gel.
~~[illegible]~~ 2nd antibody

protein 50ug/ul

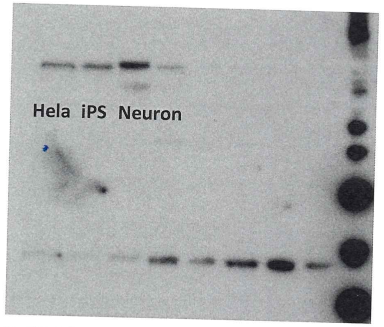

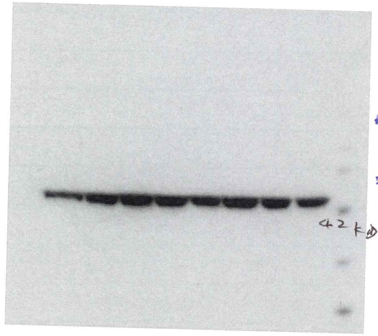

β
— actin
1st
1:10000
2nd
1:2000

Figure.4b

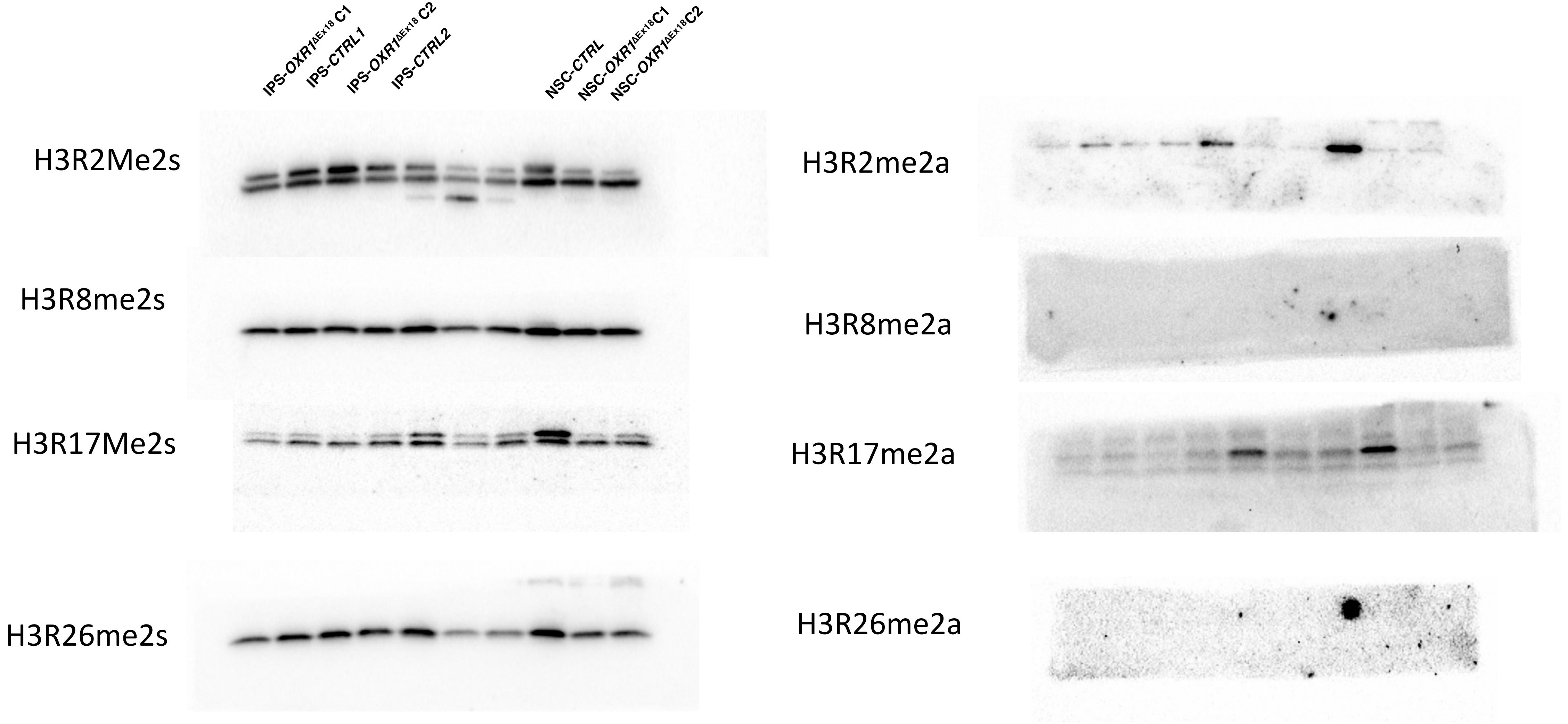
IPS-OXR1$^{\Delta Ex18}$ C1
IPS-CTRL1
IPS-OXR1$^{\Delta Ex18}$ C2
IPS-CTRL2
NSC-CTRL
NSC-OXR1$^{\Delta Ex18}$C1
NSC-OXR1$^{\Delta Ex18}$C2
H3R2Me2s
H3R8me2s
H3R17Me2s
H3R26me2s
H3R2me2a
H3R8me2a
H3R17me2a
H3R26me2a

Figure.4b

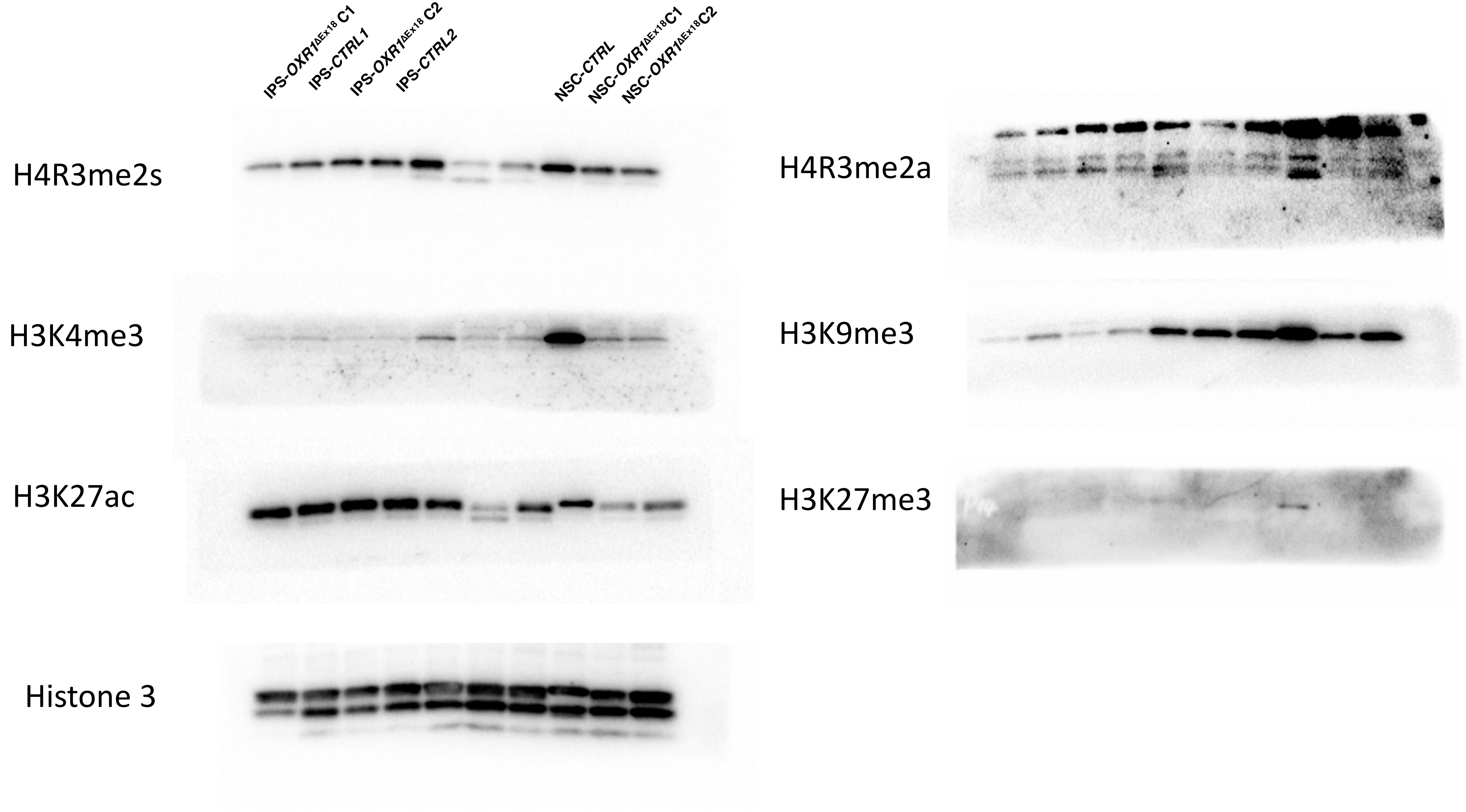
IPS-OXR1ΔEx18 C1
IPS-CTRL1
IPS-OXR1ΔEx18 C2
IPS-CTRL2
NSC-CTRL
NSC-OXR1ΔEx18C1
NSC-OXR1ΔEx18C2
H4R3me2s
H3K4me3
H3K27ac
Histone 3
H4R3me2a
H3K9me3
H3K27me3

Figure.4b

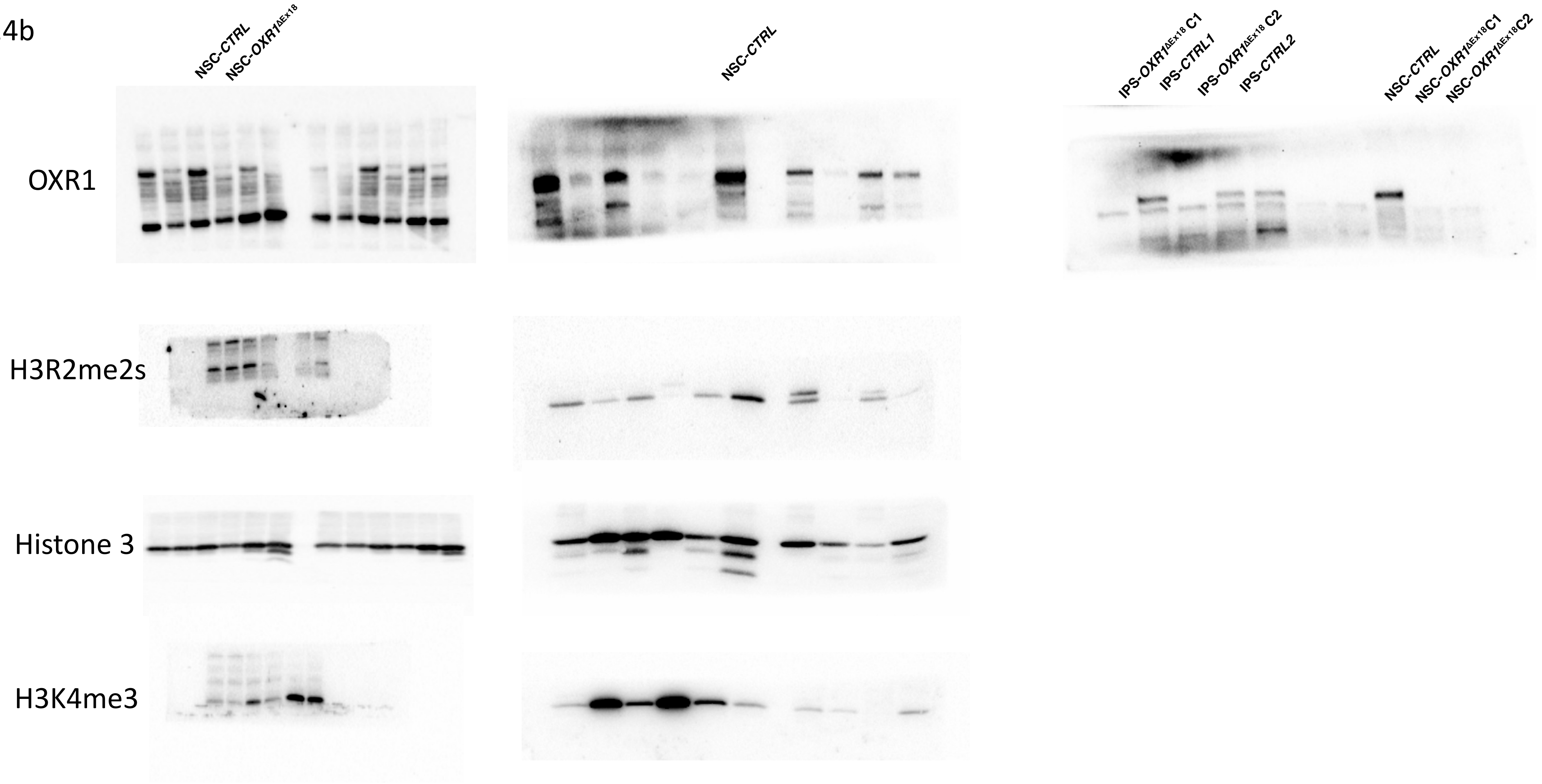
NSC-CTRL
NSC-OXR1$^{\Delta Ex18}$
NSC-CTRL
IPS-OXR1$^{\Delta Ex18}$ C1
IPS-CTRL1
IPS-OXR1$^{\Delta Ex18}$ C2
IPS-CTRL2
NSC-CTRL
NSC-OXR1$^{\Delta Ex18}$C1
NSC-OXR1$^{\Delta Ex18}$C2
OXR1
H3R2me2s
Histone 3
H3K4me3

Figure.4b

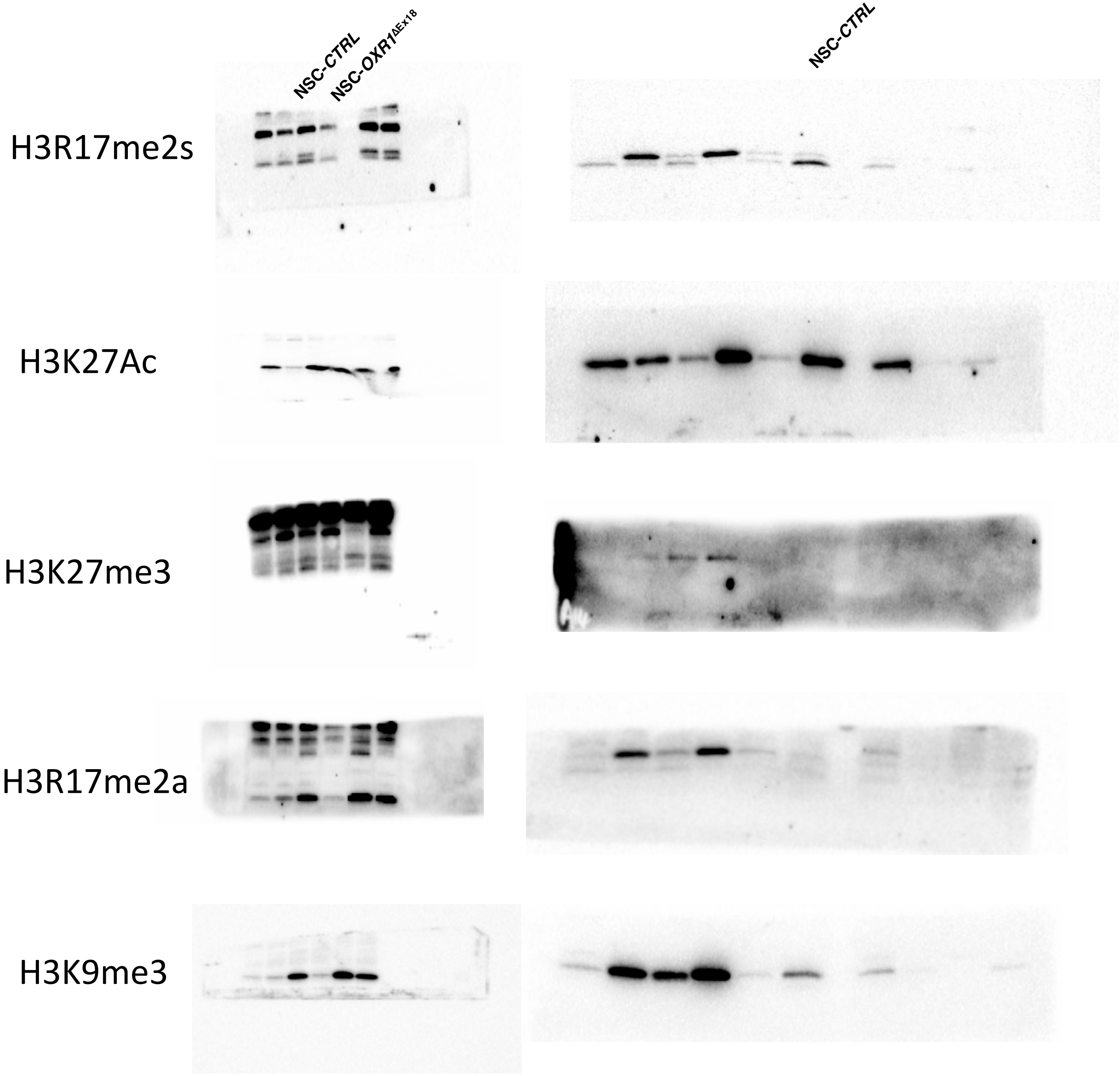
NSC-CTRL
NSC-OXR1ΔEx18
NSC-CTRL
H3R17me2s
H3K27Ac
H3K27me3
H3R17me2a
H3K9me3

Figure.4b

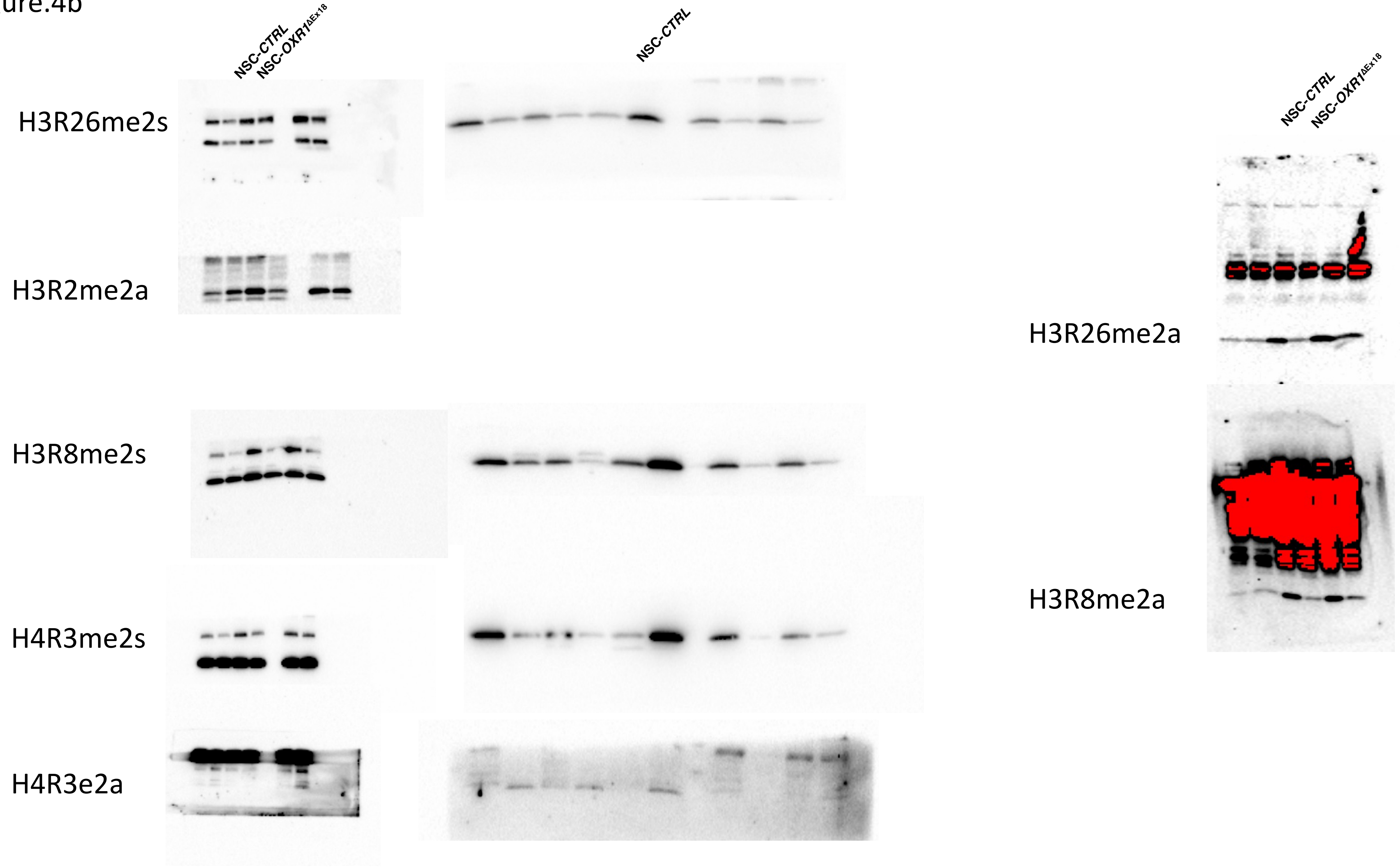
NSC-CTRL
NSC-OXR1$^{\Delta Ex18}$
NSC-CTRL
NSC-CTRL
NSC-OXR1$^{\Delta Ex18}$
H3R26me2s
H3R2me2a
H3R26me2a
H3R8me2s
H3R8me2a
H4R3me2s
H4R3e2a

Figure.S4b

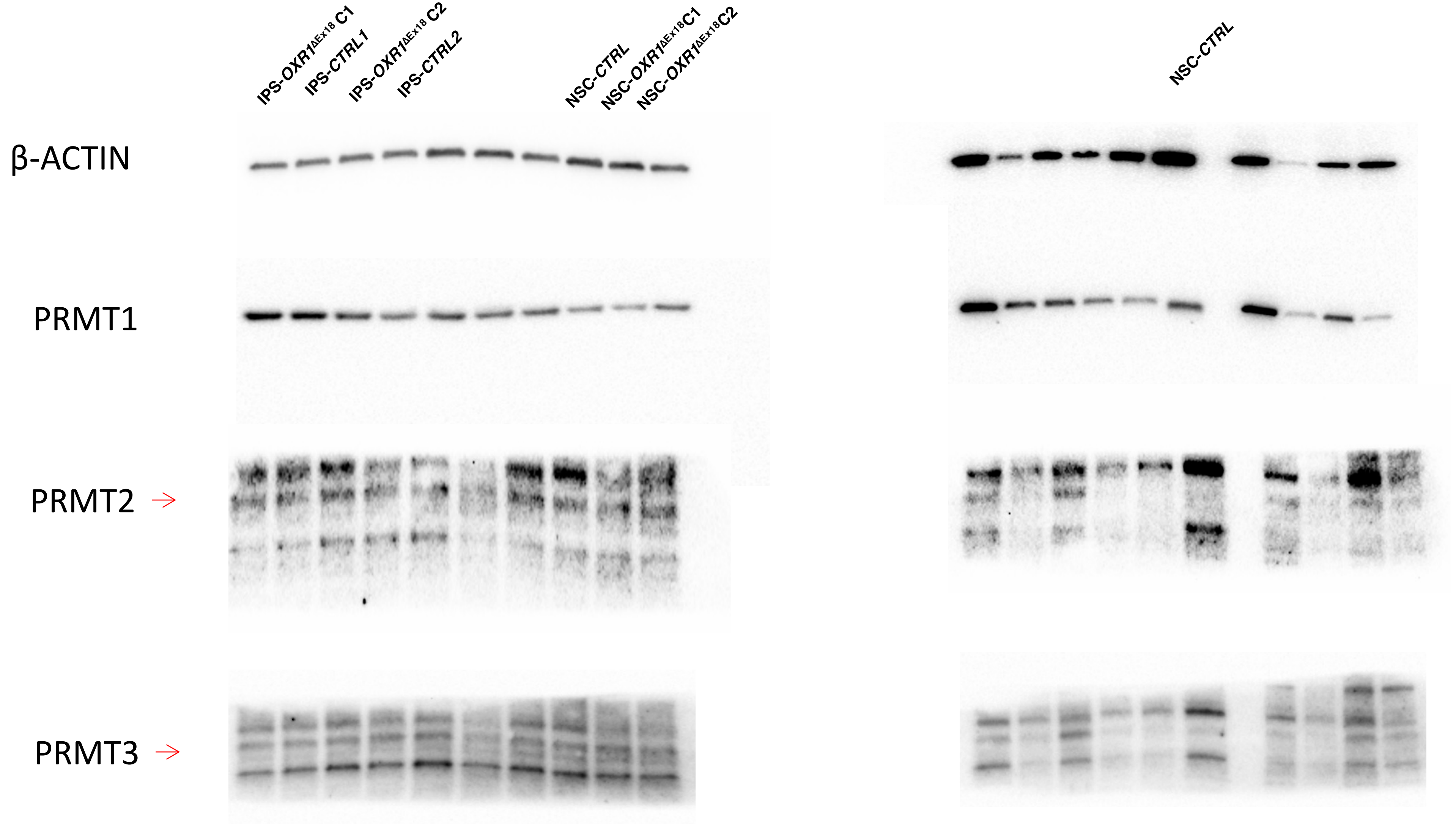

IPS-OXR1ΔEx18 C1
IPS-CTRL1
IPS-OXR1ΔEx18 C2
IPS-CTRL2
NSC-CTRL
NSC-OXR1ΔEx18C1
NSC-OXR1ΔEx18C2
NSC-CTRL
β-ACTIN
PRMT1
PRMT2
PRMT3

Figure.S4b

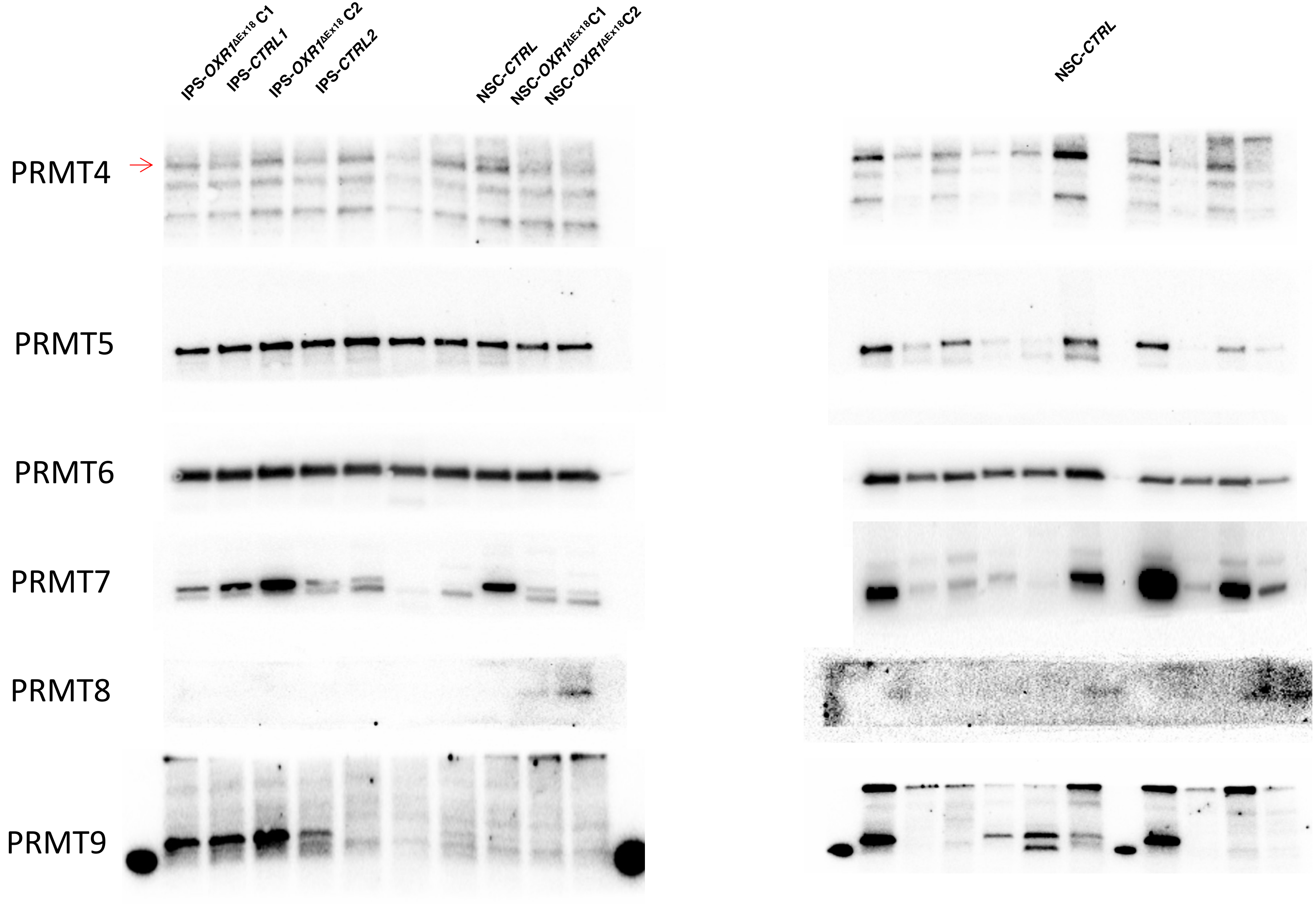

IPS-OXR1$^{\Delta Ex18}$ C1
IPS-CTRL1
IPS-OXR1$^{\Delta Ex18}$ C2
IPS-CTRL2
NSC-CTRL
NSC-OXR1$^{\Delta Ex18}$C1
NSC-OXR1$^{\Delta Ex18}$C2
NSC-CTRL
PRMT4
PRMT5
PRMT6
PRMT7
PRMT8
PRMT9

Figure.s4a

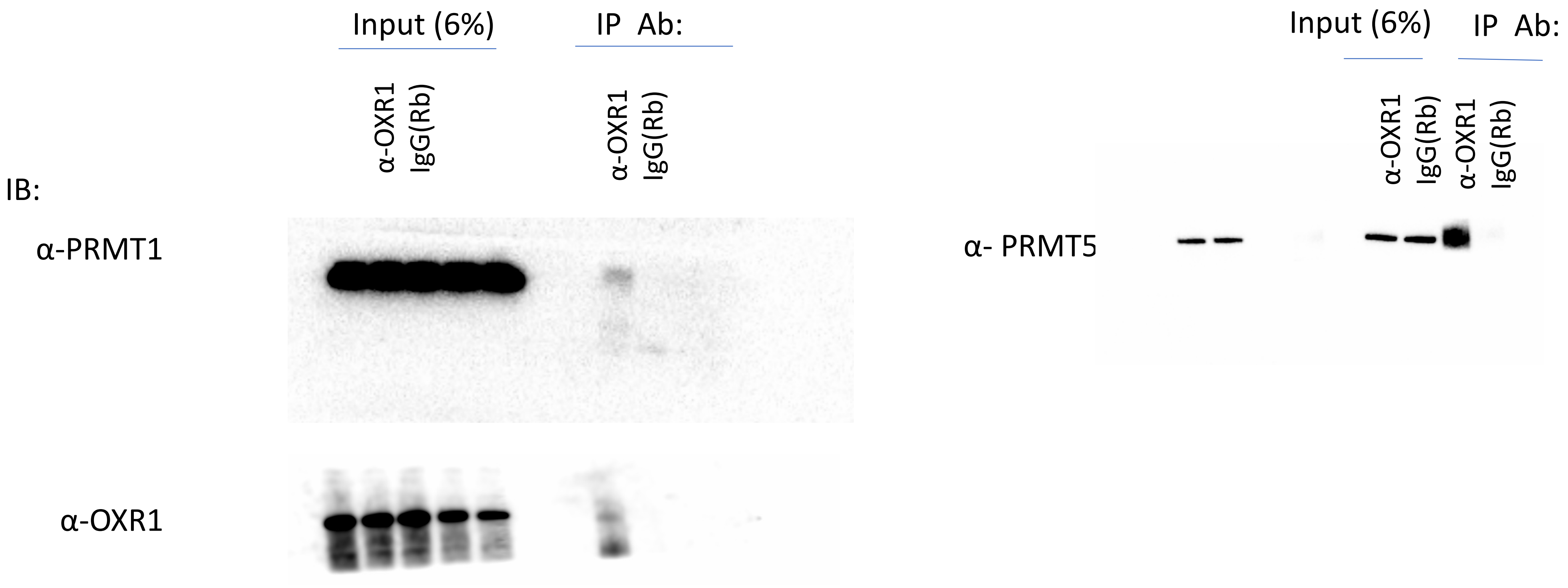

Input (6%)
IP Ab:
α-OXR1
IgG(Rb)
α-OXR1
IgG(Rb)
IB:
α-PRMT1
α-OXR1
Input (6%)
IP Ab:
α-OXR1
IgG(Rb)
α-OXR1
IgG(Rb)
α- PRMT5
