# Supplementary figures and images for: A loss-of-function mutation in human Oxidation Resistance 1 disrupts the spatial–temporal regulation of histone arginine methylation in neurodevelopment (part 2 of 2)
Source: Genome Biol. 2023 Sep 29;24:216. doi: 10.1186/s13059-023-03037-1 (PMC10540402; doi:10.1186/s13059-023-03037-1)

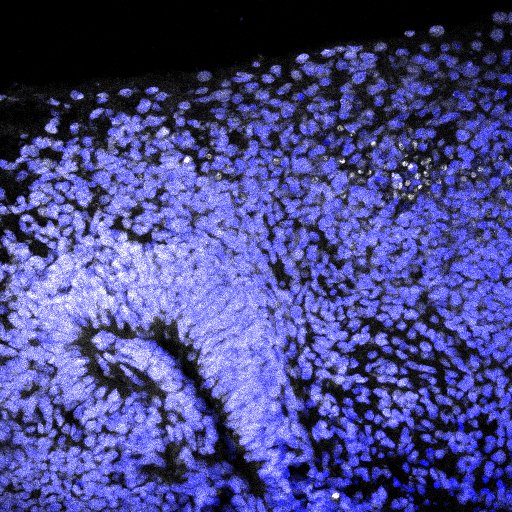

Supplement: Supplementary file 4 — Additional file 4. Uncropped gel and microscopy images. [file 13059_2023_3037_MOESM4_ESM.zip › Gel_Microscopy_images_GenomeBiology/microscopy_images/Figure S7/S7d/s7d_H3R2me2s_day50_ctrl_c2-1.jpg]

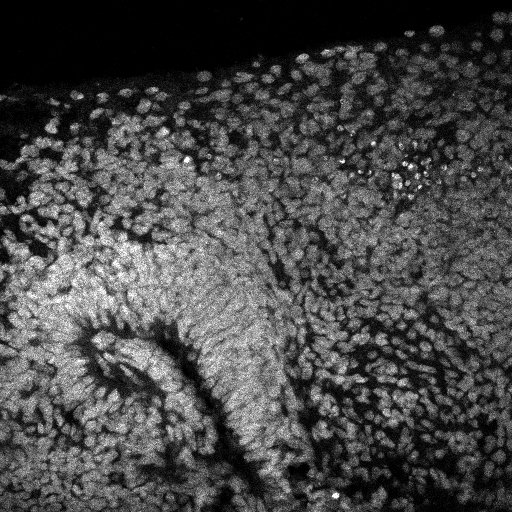

Supplement: Supplementary file 4 — Additional file 4. Uncropped gel and microscopy images. [file 13059_2023_3037_MOESM4_ESM.zip › Gel_Microscopy_images_GenomeBiology/microscopy_images/Figure S7/S7d/s7d_H3R2me2s_day50_ctrl_c2-2.jpg]

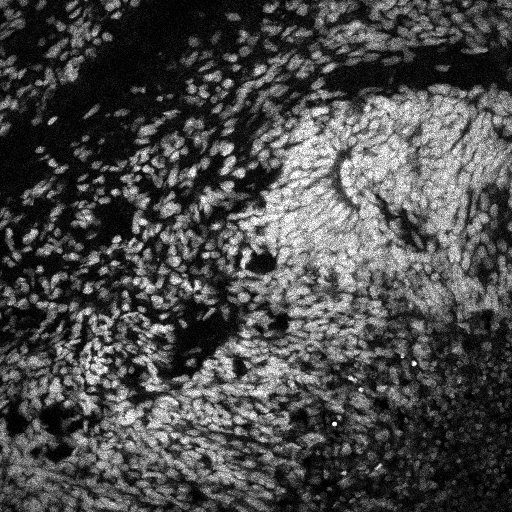

Supplement: Supplementary file 4 — Additional file 4. Uncropped gel and microscopy images. [file 13059_2023_3037_MOESM4_ESM.zip › Gel_Microscopy_images_GenomeBiology/microscopy_images/Figure S7/S7d/s7d_H3R2me2s_day50_oxr1_c2-2.jpg]

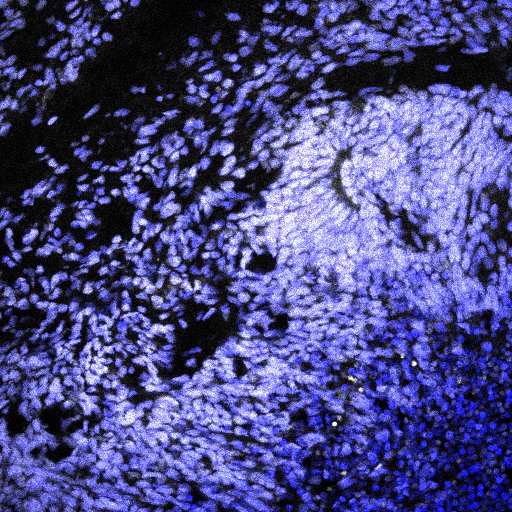

Supplement: Supplementary file 4 — Additional file 4. Uncropped gel and microscopy images. [file 13059_2023_3037_MOESM4_ESM.zip › Gel_Microscopy_images_GenomeBiology/microscopy_images/Figure S7/S7d/s7d_H3R2me2s_day50_oxr1_c2-1.jpg]

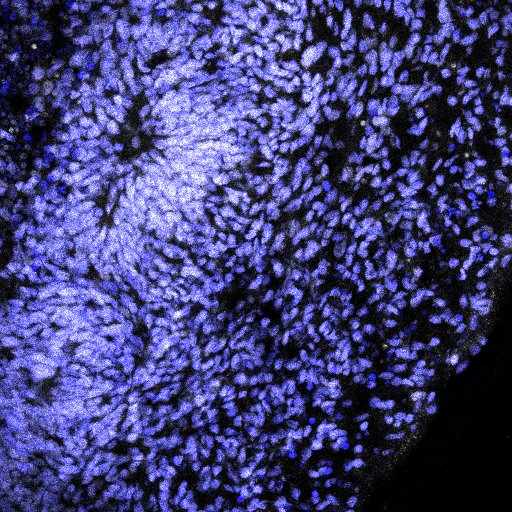

Supplement: Supplementary file 4 — Additional file 4. Uncropped gel and microscopy images. [file 13059_2023_3037_MOESM4_ESM.zip › Gel_Microscopy_images_GenomeBiology/microscopy_images/Figure S7/S7d/s7d_H3R2me2s_day50_ctrl_c1-1.jpg]

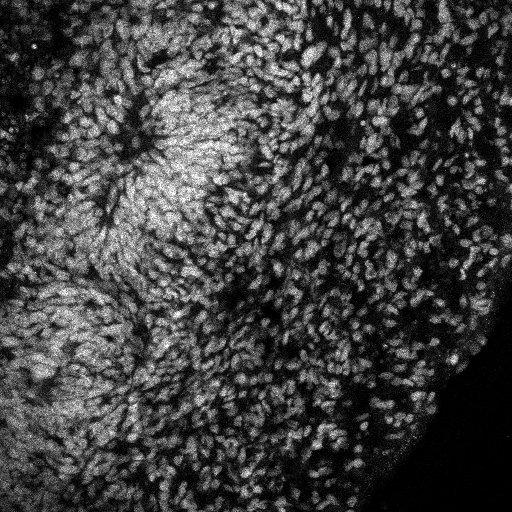

Supplement: Supplementary file 4 — Additional file 4. Uncropped gel and microscopy images. [file 13059_2023_3037_MOESM4_ESM.zip › Gel_Microscopy_images_GenomeBiology/microscopy_images/Figure S7/S7d/s7d_H3R2me2s_day50_ctrl_c1-2.jpg]

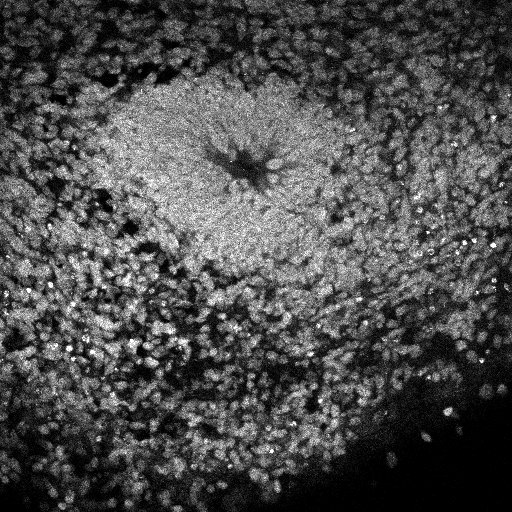

Supplement: Supplementary file 4 — Additional file 4. Uncropped gel and microscopy images. [file 13059_2023_3037_MOESM4_ESM.zip › Gel_Microscopy_images_GenomeBiology/microscopy_images/Figure S7/S7d/s7d_H3R2me2s_day50_oxr1_c1-2.jpg]

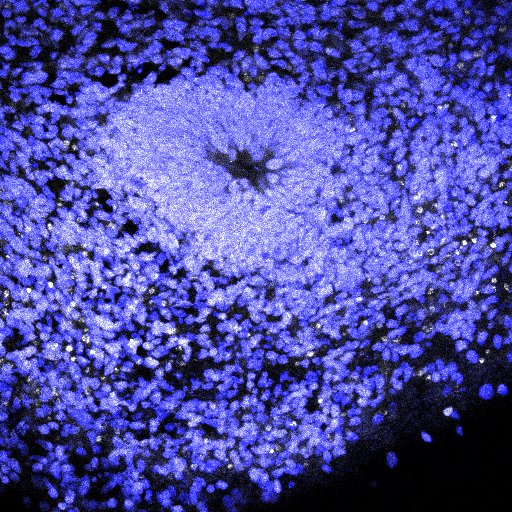

Supplement: Supplementary file 4 — Additional file 4. Uncropped gel and microscopy images. [file 13059_2023_3037_MOESM4_ESM.zip › Gel_Microscopy_images_GenomeBiology/microscopy_images/Figure S7/S7d/s7d_H3R2me2s_day50_oxr1_c1-1.jpg]

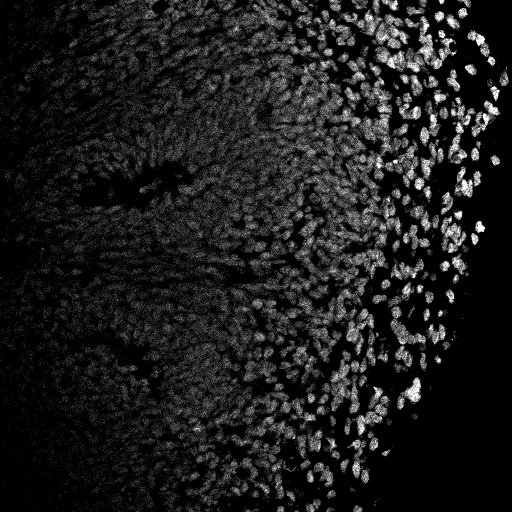

Supplement: Supplementary file 4 — Additional file 4. Uncropped gel and microscopy images. [file 13059_2023_3037_MOESM4_ESM.zip › Gel_Microscopy_images_GenomeBiology/microscopy_images/Figure S7/S7f/s7f_H3K4me3_day50_ctrl_c1-2.jpg]

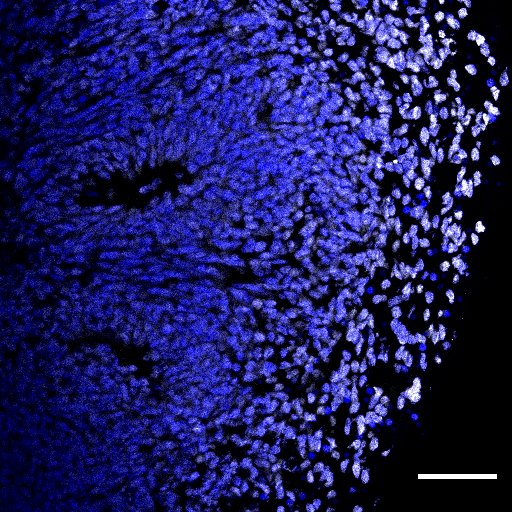

Supplement: Supplementary file 4 — Additional file 4. Uncropped gel and microscopy images. [file 13059_2023_3037_MOESM4_ESM.zip › Gel_Microscopy_images_GenomeBiology/microscopy_images/Figure S7/S7f/s7f_H3K4me3_day50_ctrl_c1-1.jpg]

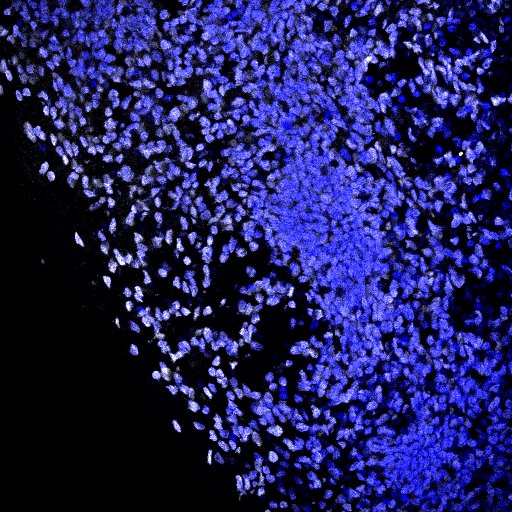

Supplement: Supplementary file 4 — Additional file 4. Uncropped gel and microscopy images. [file 13059_2023_3037_MOESM4_ESM.zip › Gel_Microscopy_images_GenomeBiology/microscopy_images/Figure S7/S7f/s7f_H3K4me3_day50_oxr1_c1-1.jpg]

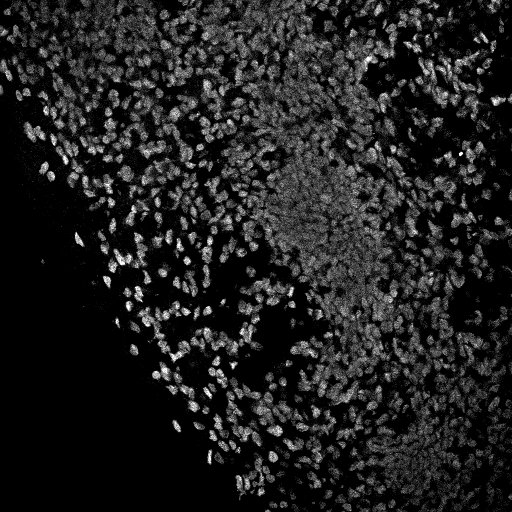

Supplement: Supplementary file 4 — Additional file 4. Uncropped gel and microscopy images. [file 13059_2023_3037_MOESM4_ESM.zip › Gel_Microscopy_images_GenomeBiology/microscopy_images/Figure S7/S7f/s7f_H3K4me3_day50_oxr1_c1-2.jpg]

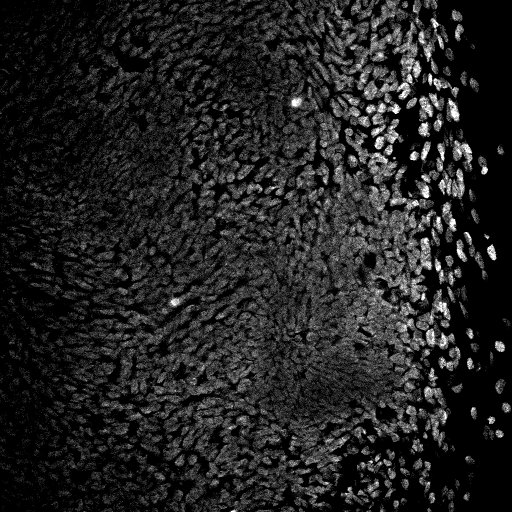

Supplement: Supplementary file 4 — Additional file 4. Uncropped gel and microscopy images. [file 13059_2023_3037_MOESM4_ESM.zip › Gel_Microscopy_images_GenomeBiology/microscopy_images/Figure S7/S7f/s7f_H3K4me3_day50_ctrl_c2-2.jpg]

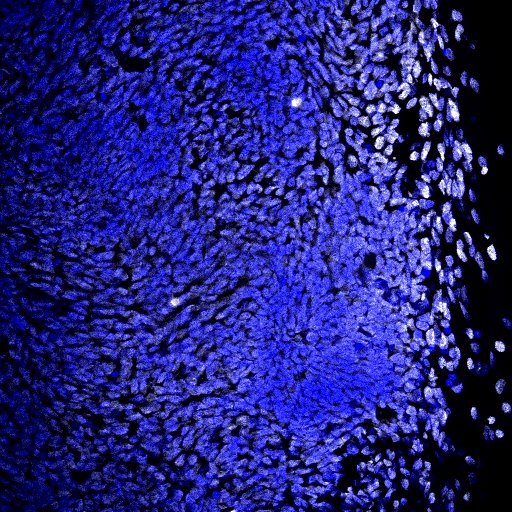

Supplement: Supplementary file 4 — Additional file 4. Uncropped gel and microscopy images. [file 13059_2023_3037_MOESM4_ESM.zip › Gel_Microscopy_images_GenomeBiology/microscopy_images/Figure S7/S7f/s7f_H3K4me3_day50_ctrl_c2-1.jpg]

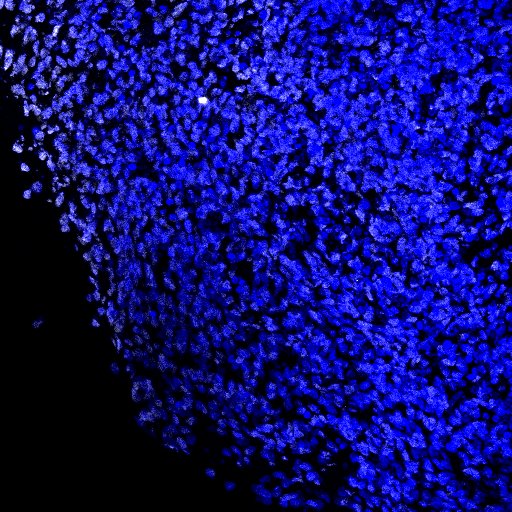

Supplement: Supplementary file 4 — Additional file 4. Uncropped gel and microscopy images. [file 13059_2023_3037_MOESM4_ESM.zip › Gel_Microscopy_images_GenomeBiology/microscopy_images/Figure S7/S7f/s7f_H3K4me3_day50_oxr1_c2-1.jpg]

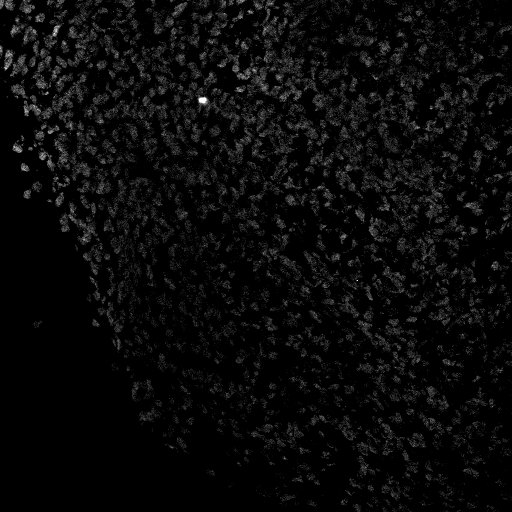

Supplement: Supplementary file 4 — Additional file 4. Uncropped gel and microscopy images. [file 13059_2023_3037_MOESM4_ESM.zip › Gel_Microscopy_images_GenomeBiology/microscopy_images/Figure S7/S7f/s7f_H3K4me3_day50_oxr1_c2-2.jpg]

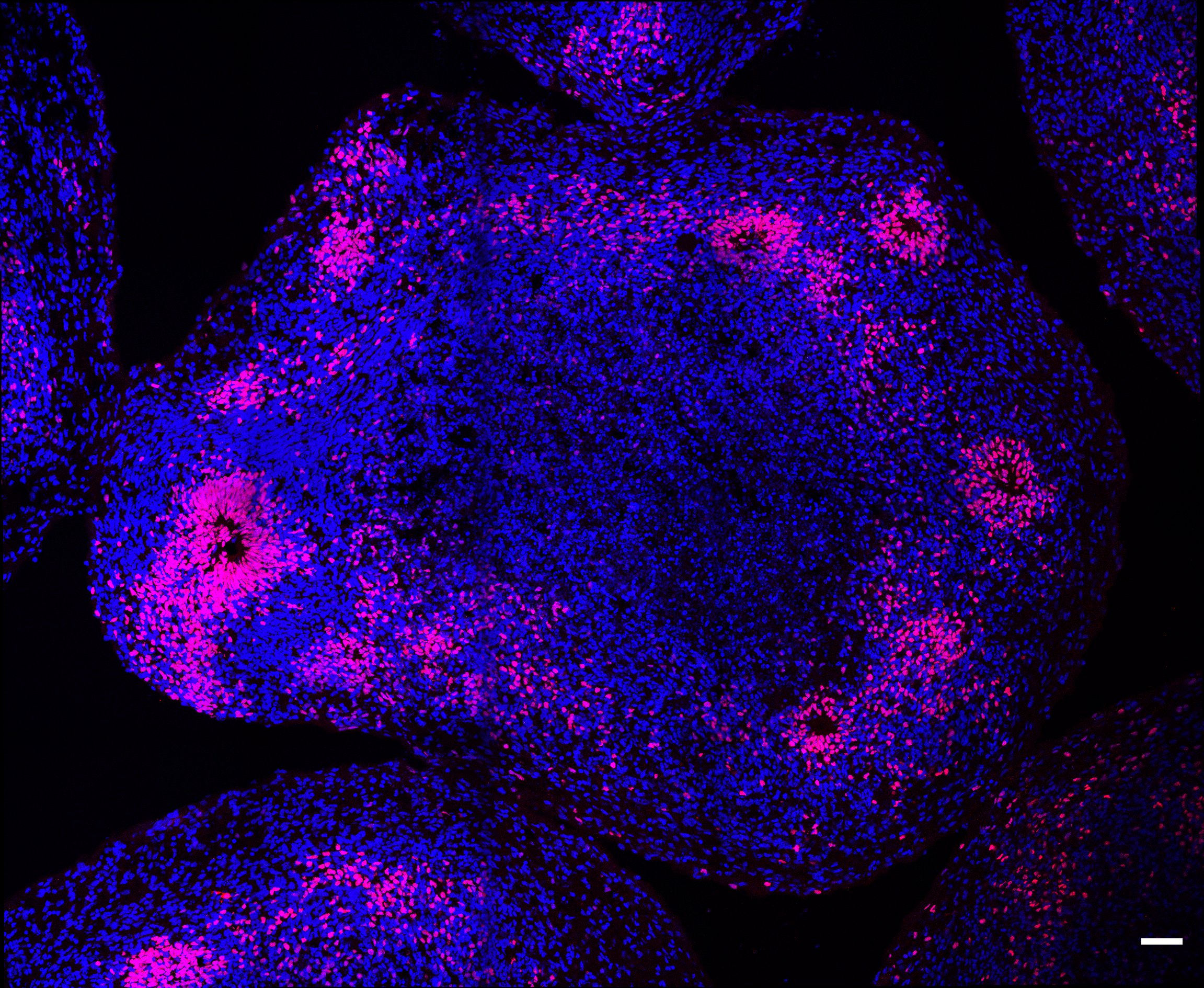

Supplement: Supplementary file 4 — Additional file 4. Uncropped gel and microscopy images. [file 13059_2023_3037_MOESM4_ESM.zip › Gel_Microscopy_images_GenomeBiology/microscopy_images/Figure 5/5g/fig5g_sox2_day65_oxr1_c1.jpg]

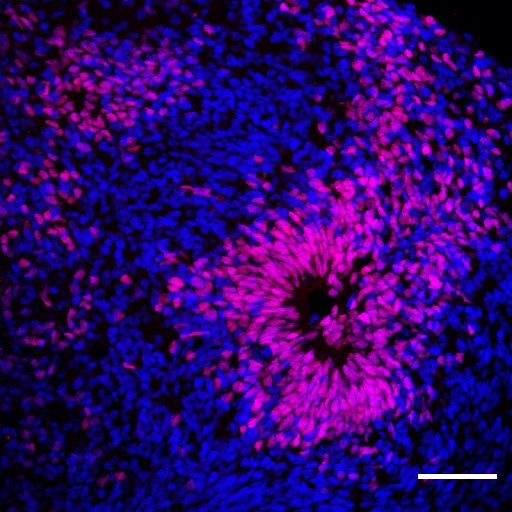

Supplement: Supplementary file 4 — Additional file 4. Uncropped gel and microscopy images. [file 13059_2023_3037_MOESM4_ESM.zip › Gel_Microscopy_images_GenomeBiology/microscopy_images/Figure 5/5g/fig5g_sox2_day65_oxr1_c1-2.jpg]

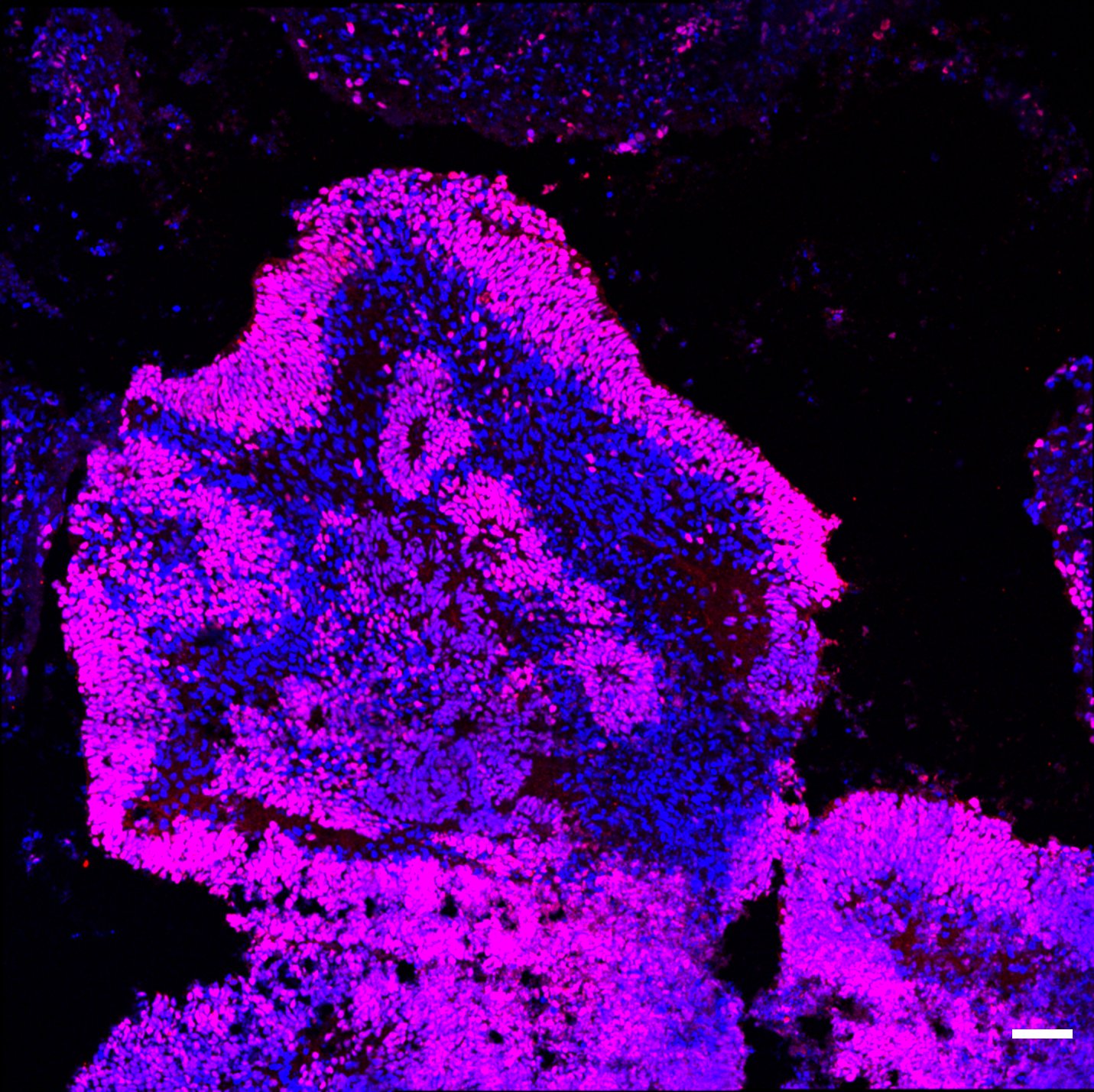

Supplement: Supplementary file 4 — Additional file 4. Uncropped gel and microscopy images. [file 13059_2023_3037_MOESM4_ESM.zip › Gel_Microscopy_images_GenomeBiology/microscopy_images/Figure 5/5g/fig5g_sox2_day50_ctrl_c2 .jpg]

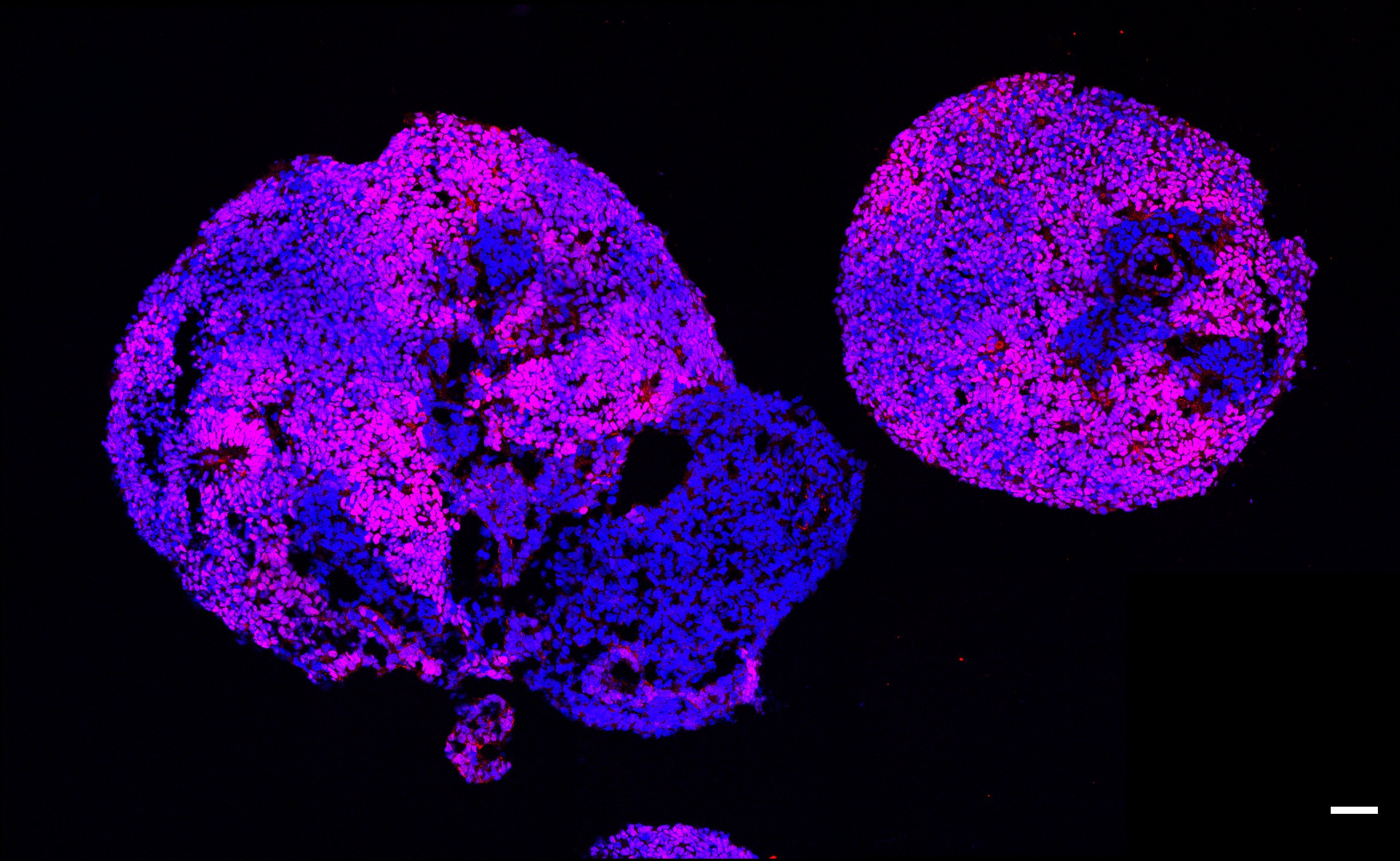

Supplement: Supplementary file 4 — Additional file 4. Uncropped gel and microscopy images. [file 13059_2023_3037_MOESM4_ESM.zip › Gel_Microscopy_images_GenomeBiology/microscopy_images/Figure 5/5g/fig5g_sox2_day20_ctrl_c1.jpg]

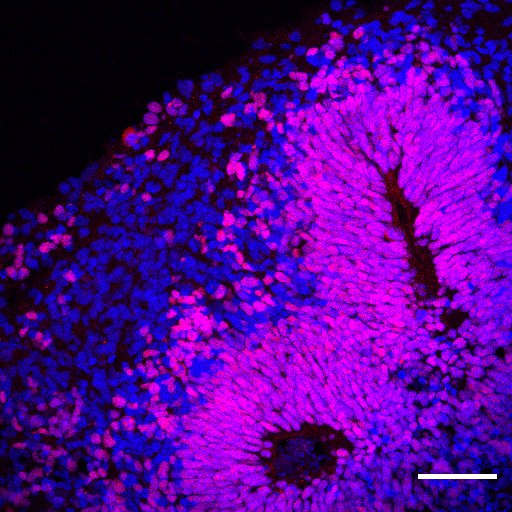

Supplement: Supplementary file 4 — Additional file 4. Uncropped gel and microscopy images. [file 13059_2023_3037_MOESM4_ESM.zip › Gel_Microscopy_images_GenomeBiology/microscopy_images/Figure 5/5g/fig5g_sox2_day50_ctrl_c1.jpg]

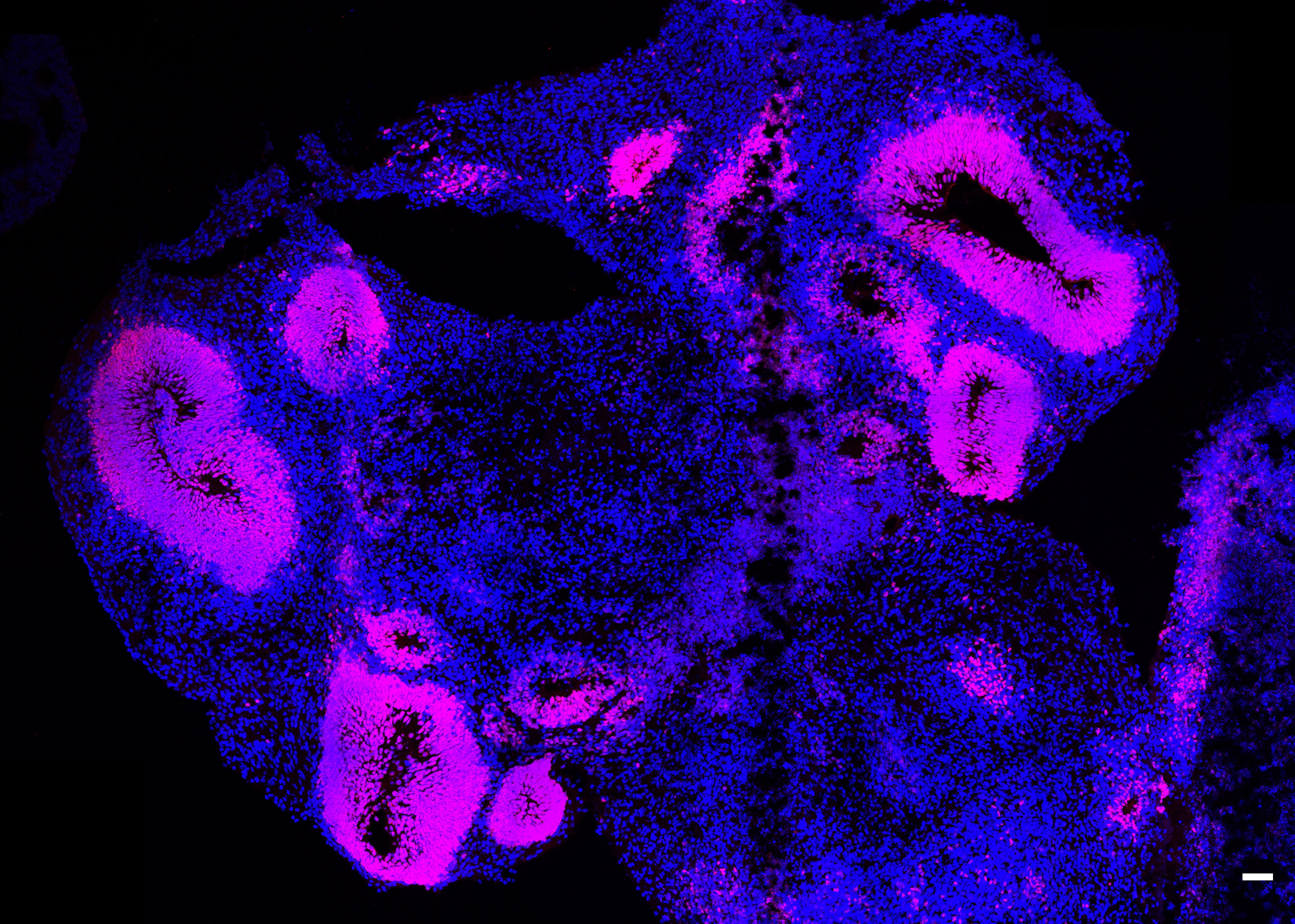

Supplement: Supplementary file 4 — Additional file 4. Uncropped gel and microscopy images. [file 13059_2023_3037_MOESM4_ESM.zip › Gel_Microscopy_images_GenomeBiology/microscopy_images/Figure 5/5g/fig5g_sox2_day59_ctrl_c1.jpg]

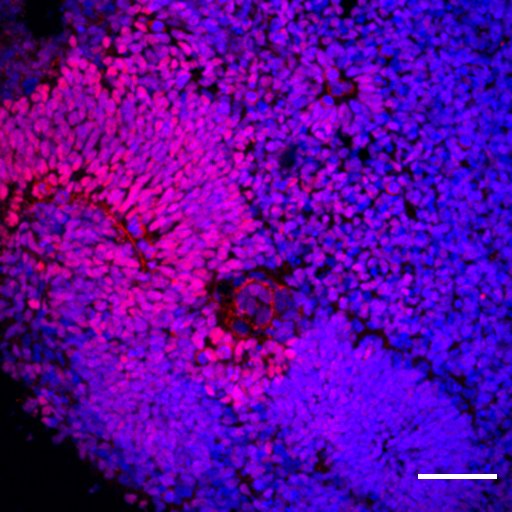

Supplement: Supplementary file 4 — Additional file 4. Uncropped gel and microscopy images. [file 13059_2023_3037_MOESM4_ESM.zip › Gel_Microscopy_images_GenomeBiology/microscopy_images/Figure 5/5g/fig5g_sox2_day20_ctrl_c2.jpg]

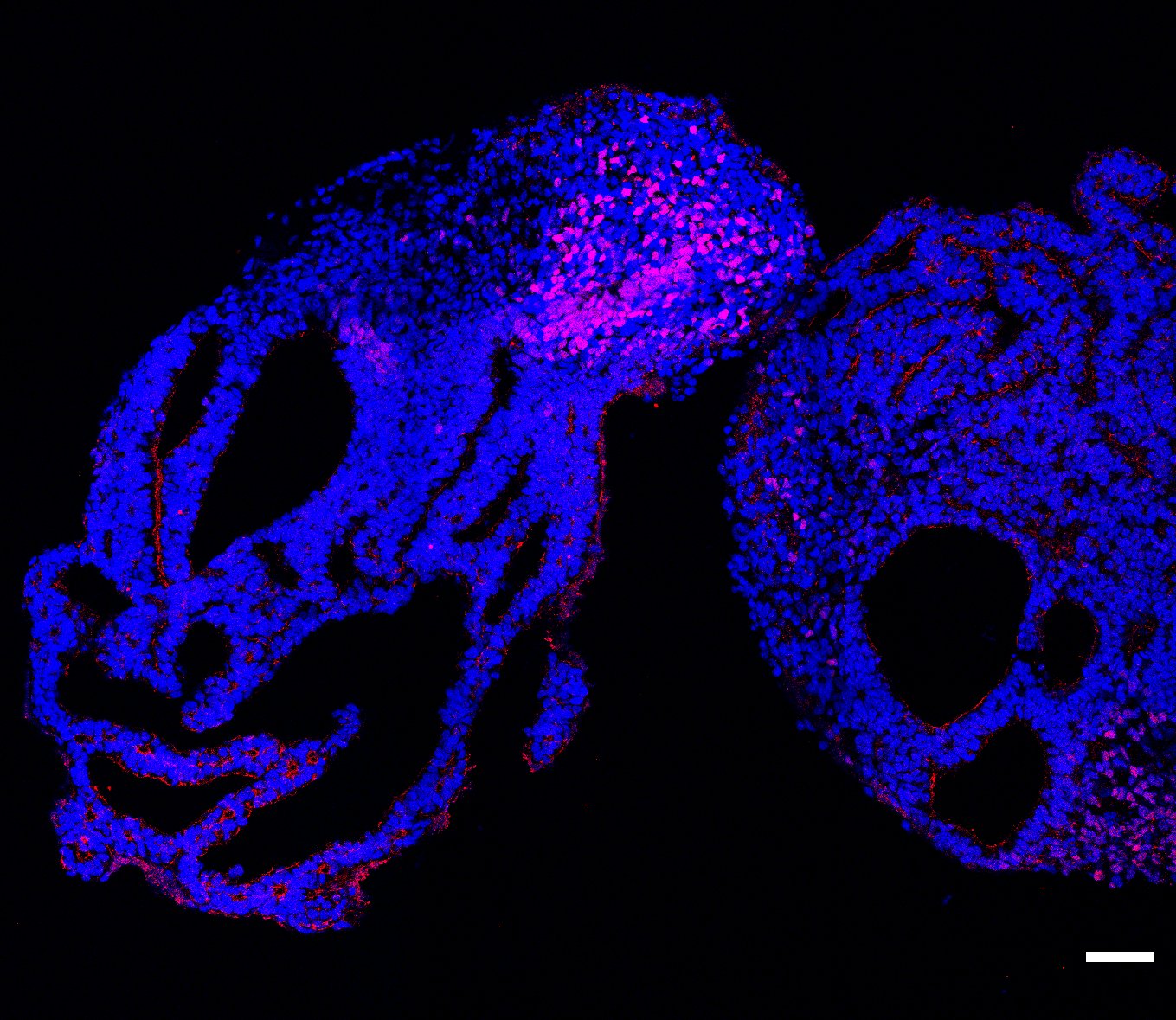

Supplement: Supplementary file 4 — Additional file 4. Uncropped gel and microscopy images. [file 13059_2023_3037_MOESM4_ESM.zip › Gel_Microscopy_images_GenomeBiology/microscopy_images/Figure 5/5g/fig5g_sox2_day50_oxr1_c1-1.jpg]

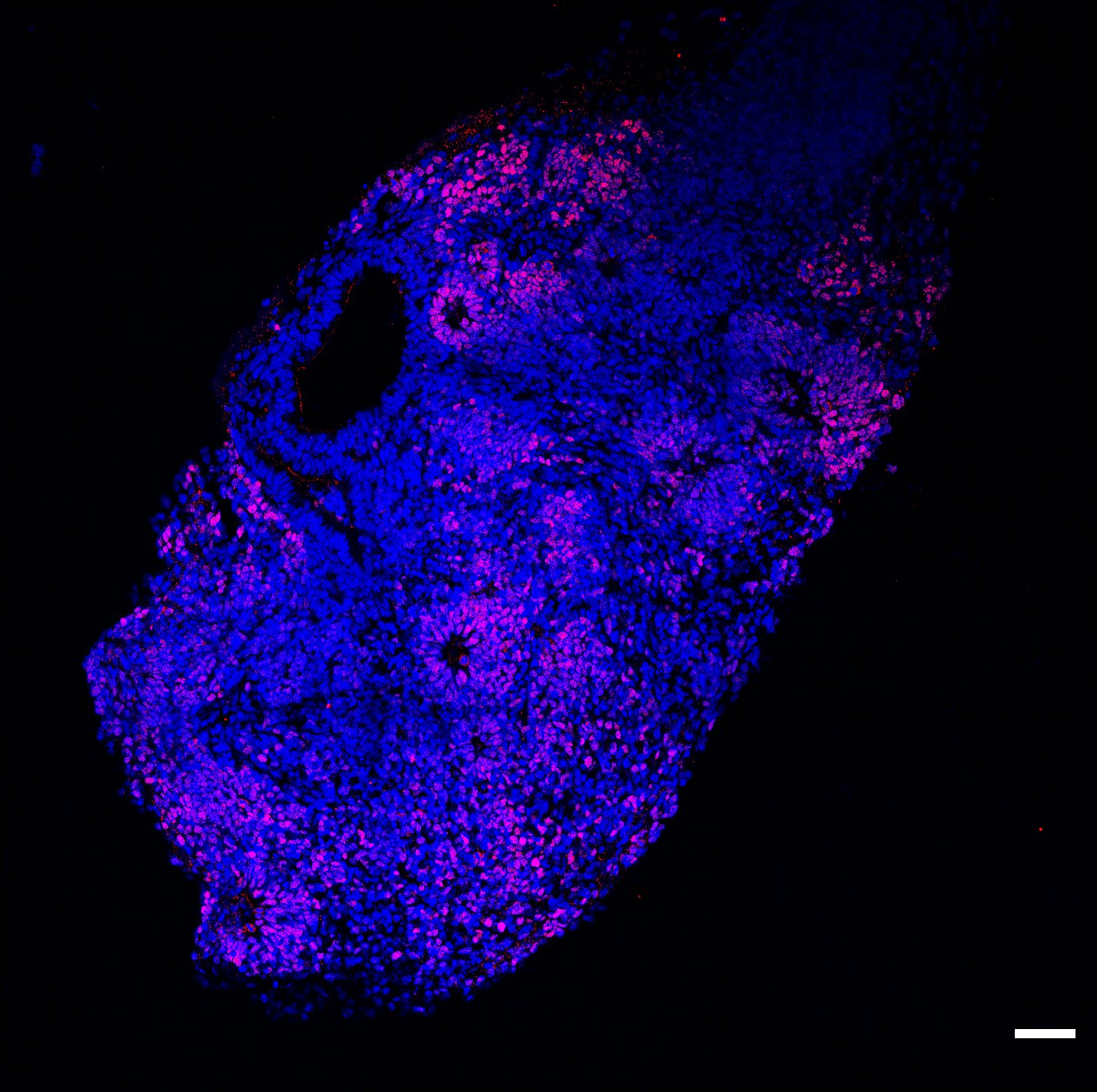

Supplement: Supplementary file 4 — Additional file 4. Uncropped gel and microscopy images. [file 13059_2023_3037_MOESM4_ESM.zip › Gel_Microscopy_images_GenomeBiology/microscopy_images/Figure 5/5g/fig5g_sox2_day50_oxr1_c1-2.jpg]

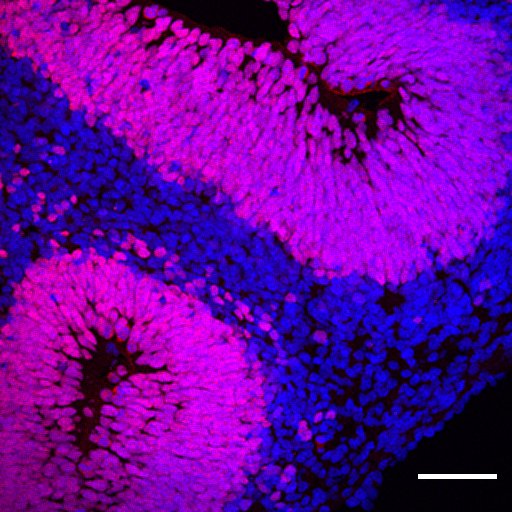

Supplement: Supplementary file 4 — Additional file 4. Uncropped gel and microscopy images. [file 13059_2023_3037_MOESM4_ESM.zip › Gel_Microscopy_images_GenomeBiology/microscopy_images/Figure 5/5g/fig5g_sox2_day59_ctrl_c1-1.jpg]

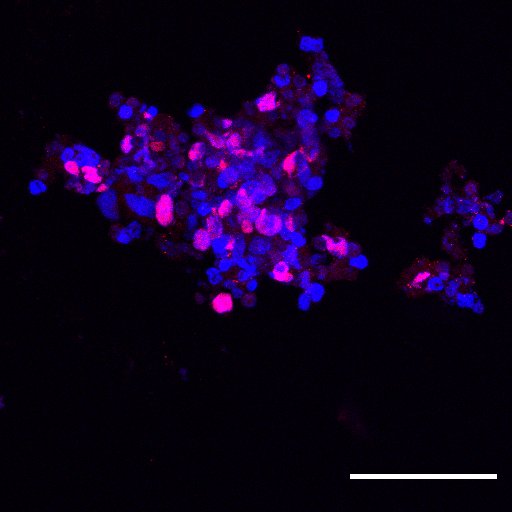

Supplement: Supplementary file 4 — Additional file 4. Uncropped gel and microscopy images. [file 13059_2023_3037_MOESM4_ESM.zip › Gel_Microscopy_images_GenomeBiology/microscopy_images/Figure 5/5g/fig5g_sox2_day20_oxr1_c1.jpg]

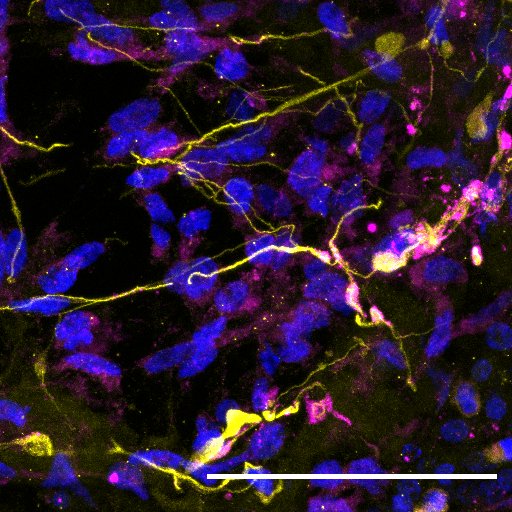

Supplement: Supplementary file 4 — Additional file 4. Uncropped gel and microscopy images. [file 13059_2023_3037_MOESM4_ESM.zip › Gel_Microscopy_images_GenomeBiology/microscopy_images/Figure 5/5c/fig5c_OXR1_REELIN_ctrl_c2-4.jpg]

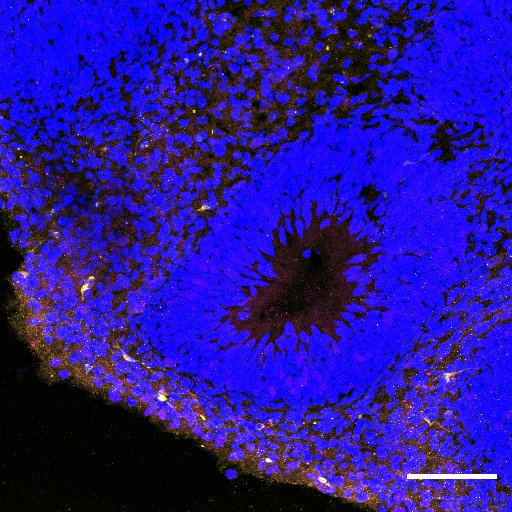

Supplement: Supplementary file 4 — Additional file 4. Uncropped gel and microscopy images. [file 13059_2023_3037_MOESM4_ESM.zip › Gel_Microscopy_images_GenomeBiology/microscopy_images/Figure 5/5c/fig5c_OXR1_REELIN_oxr1_c2-1.jpg]

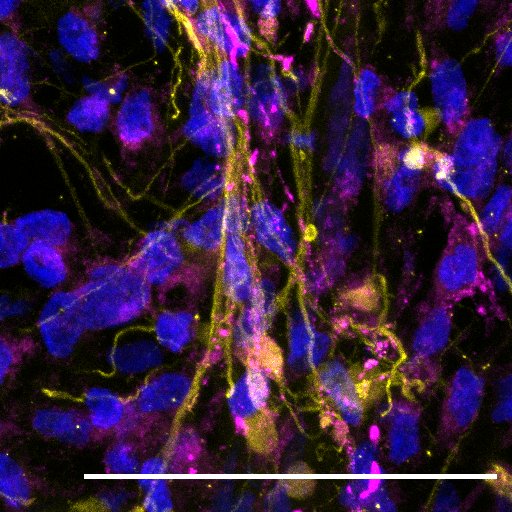

Supplement: Supplementary file 4 — Additional file 4. Uncropped gel and microscopy images. [file 13059_2023_3037_MOESM4_ESM.zip › Gel_Microscopy_images_GenomeBiology/microscopy_images/Figure 5/5c/fig5c_OXR1_REELIN_ctrl_c2-5.jpg]

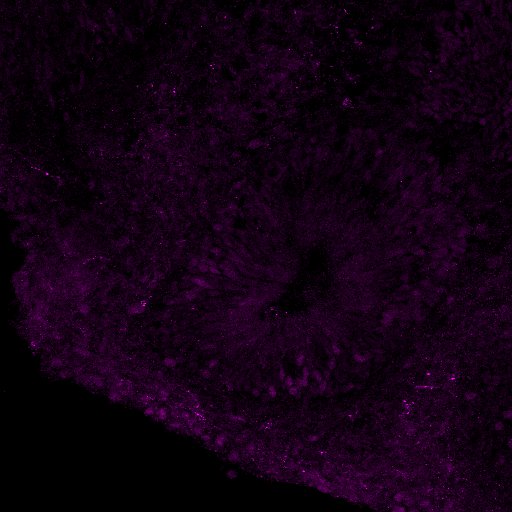

Supplement: Supplementary file 4 — Additional file 4. Uncropped gel and microscopy images. [file 13059_2023_3037_MOESM4_ESM.zip › Gel_Microscopy_images_GenomeBiology/microscopy_images/Figure 5/5c/fig5c_OXR1_REELIN_oxr1_c2-3.jpg]

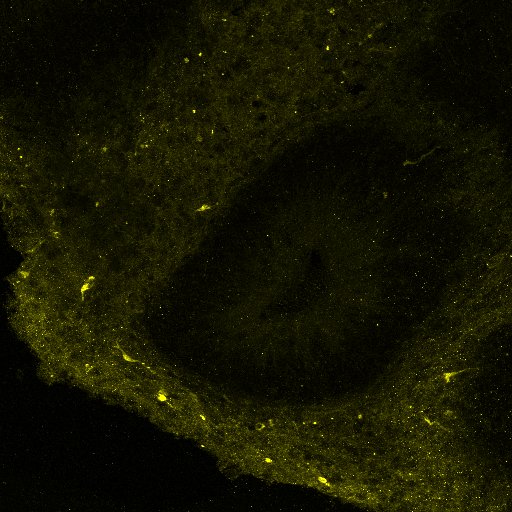

Supplement: Supplementary file 4 — Additional file 4. Uncropped gel and microscopy images. [file 13059_2023_3037_MOESM4_ESM.zip › Gel_Microscopy_images_GenomeBiology/microscopy_images/Figure 5/5c/fig5c_OXR1_REELIN_oxr1_c2-2.jpg]

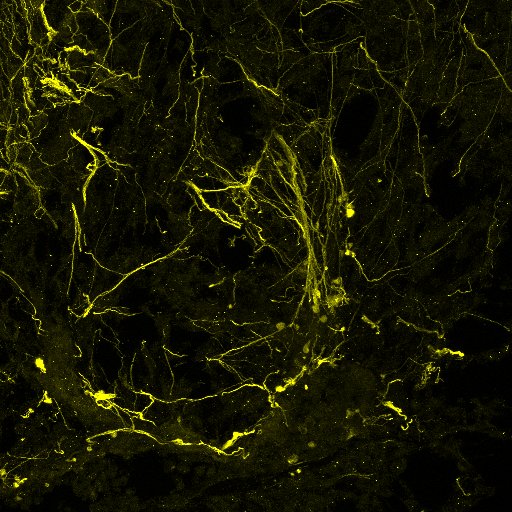

Supplement: Supplementary file 4 — Additional file 4. Uncropped gel and microscopy images. [file 13059_2023_3037_MOESM4_ESM.zip › Gel_Microscopy_images_GenomeBiology/microscopy_images/Figure 5/5c/fig5c_OXR1_REELIN_ctrl_c2-2.jpg]

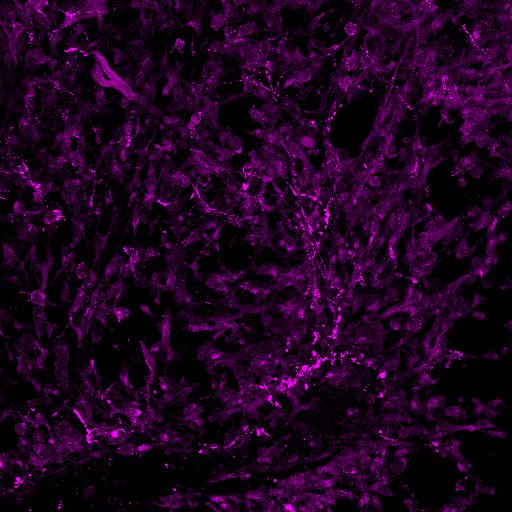

Supplement: Supplementary file 4 — Additional file 4. Uncropped gel and microscopy images. [file 13059_2023_3037_MOESM4_ESM.zip › Gel_Microscopy_images_GenomeBiology/microscopy_images/Figure 5/5c/fig5c_OXR1_REELIN_ctrl_c2-3.jpg]

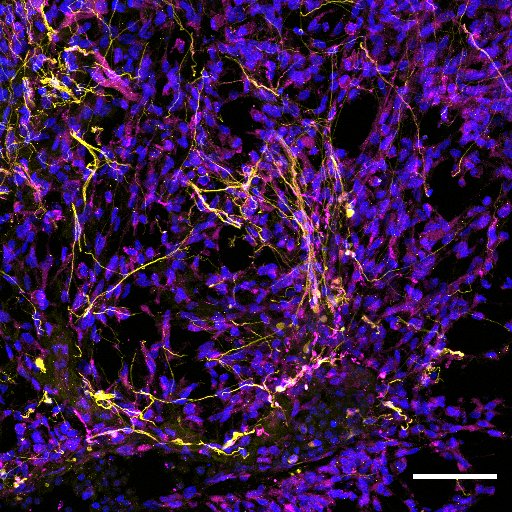

Supplement: Supplementary file 4 — Additional file 4. Uncropped gel and microscopy images. [file 13059_2023_3037_MOESM4_ESM.zip › Gel_Microscopy_images_GenomeBiology/microscopy_images/Figure 5/5c/fig5c_OXR1_REELIN_ctrl_c2-1.jpg]

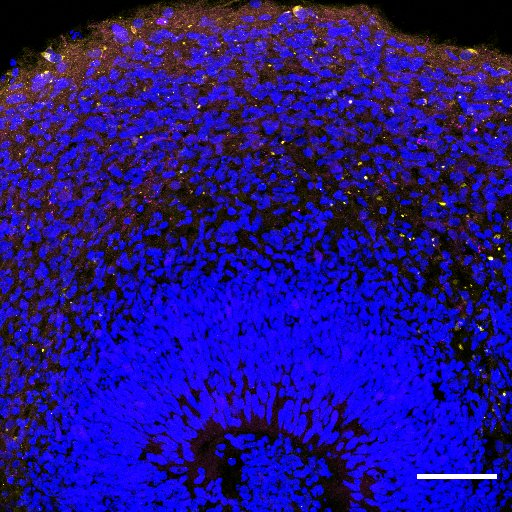

Supplement: Supplementary file 4 — Additional file 4. Uncropped gel and microscopy images. [file 13059_2023_3037_MOESM4_ESM.zip › Gel_Microscopy_images_GenomeBiology/microscopy_images/Figure 5/5c/fig5c_OXR1_REELIN_oxr1_c1-1.jpg]

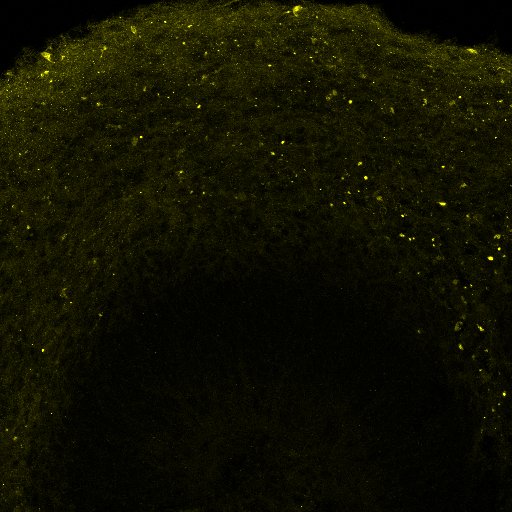

Supplement: Supplementary file 4 — Additional file 4. Uncropped gel and microscopy images. [file 13059_2023_3037_MOESM4_ESM.zip › Gel_Microscopy_images_GenomeBiology/microscopy_images/Figure 5/5c/fig5c_OXR1_REELIN_oxr1_c1-2.jpg]

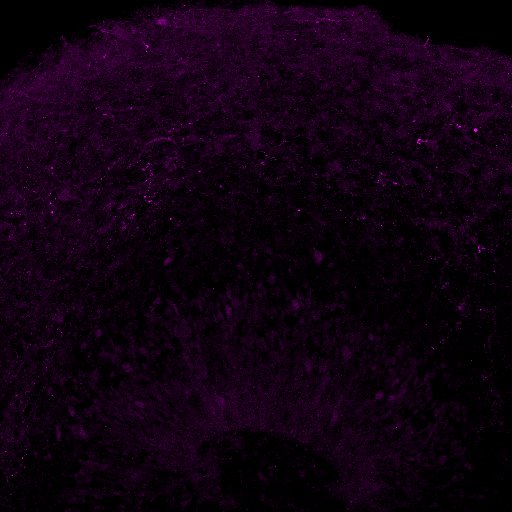

Supplement: Supplementary file 4 — Additional file 4. Uncropped gel and microscopy images. [file 13059_2023_3037_MOESM4_ESM.zip › Gel_Microscopy_images_GenomeBiology/microscopy_images/Figure 5/5c/fig5c_OXR1_REELIN_oxr1_c1-3.jpg]

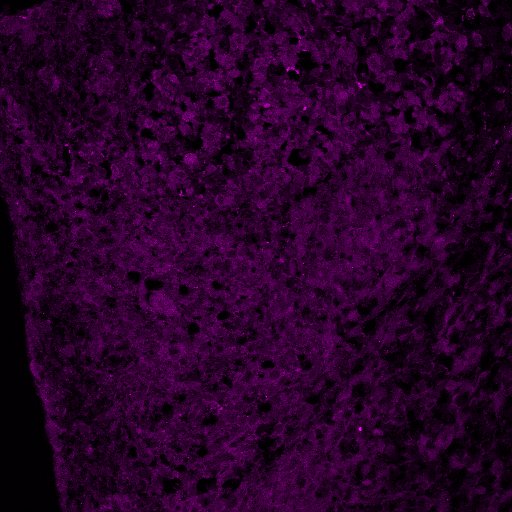

Supplement: Supplementary file 4 — Additional file 4. Uncropped gel and microscopy images. [file 13059_2023_3037_MOESM4_ESM.zip › Gel_Microscopy_images_GenomeBiology/microscopy_images/Figure 5/5c/fig5c_OXR1_REELIN_ctrl_c1-3.jpg]

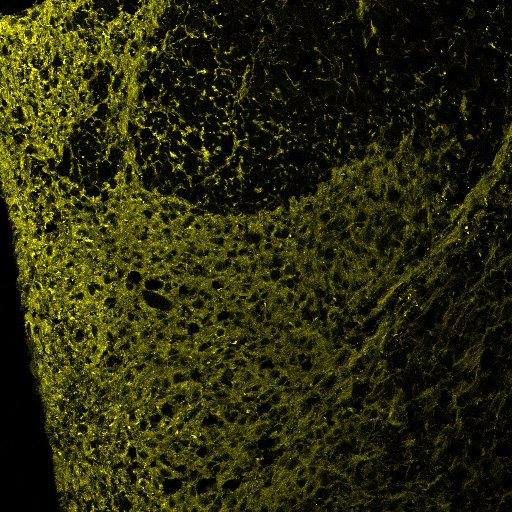

Supplement: Supplementary file 4 — Additional file 4. Uncropped gel and microscopy images. [file 13059_2023_3037_MOESM4_ESM.zip › Gel_Microscopy_images_GenomeBiology/microscopy_images/Figure 5/5c/fig5c_OXR1_REELIN_ctrl_c1-2.jpg]

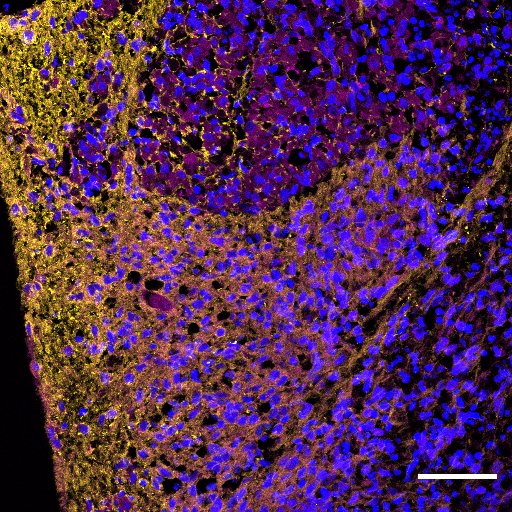

Supplement: Supplementary file 4 — Additional file 4. Uncropped gel and microscopy images. [file 13059_2023_3037_MOESM4_ESM.zip › Gel_Microscopy_images_GenomeBiology/microscopy_images/Figure 5/5c/fig5c_OXR1_REELIN_ctrl_c1-1.jpg]

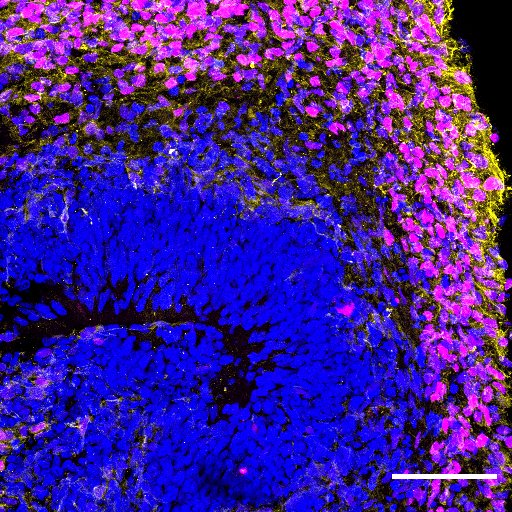

Supplement: Supplementary file 4 — Additional file 4. Uncropped gel and microscopy images. [file 13059_2023_3037_MOESM4_ESM.zip › Gel_Microscopy_images_GenomeBiology/microscopy_images/Figure 5/5b/fig5b_MAP2_TBR1_oxr1_c2-1.jpg]

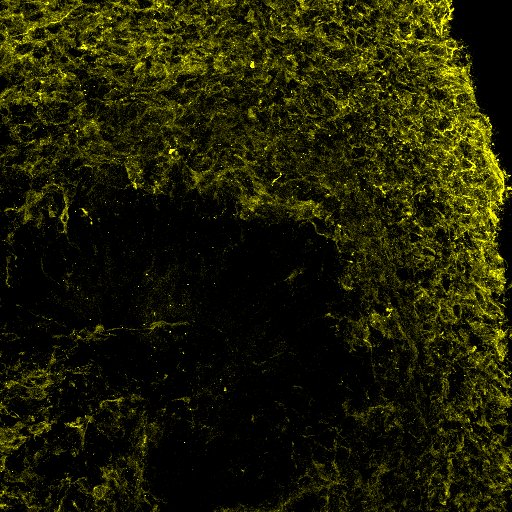

Supplement: Supplementary file 4 — Additional file 4. Uncropped gel and microscopy images. [file 13059_2023_3037_MOESM4_ESM.zip › Gel_Microscopy_images_GenomeBiology/microscopy_images/Figure 5/5b/fig5b_MAP2_TBR1_oxr1_c2-3.jpg]

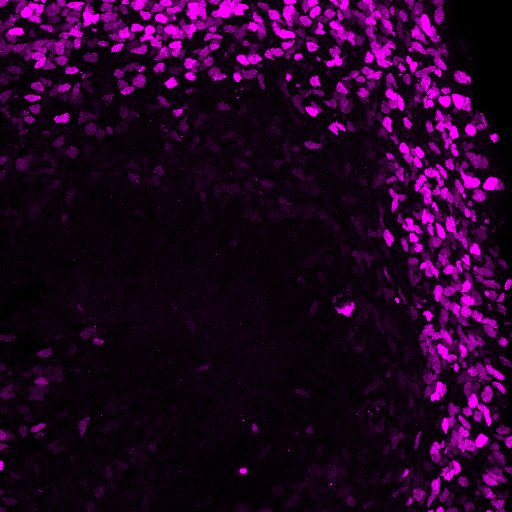

Supplement: Supplementary file 4 — Additional file 4. Uncropped gel and microscopy images. [file 13059_2023_3037_MOESM4_ESM.zip › Gel_Microscopy_images_GenomeBiology/microscopy_images/Figure 5/5b/fig5b_MAP2_TBR1_oxr1_c2-2.jpg]

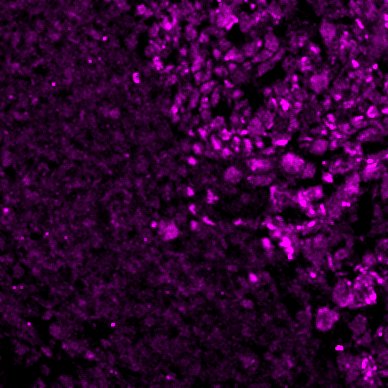

Supplement: Supplementary file 4 — Additional file 4. Uncropped gel and microscopy images. [file 13059_2023_3037_MOESM4_ESM.zip › Gel_Microscopy_images_GenomeBiology/microscopy_images/Figure 5/5b/fig5b_MAP2_TBR1_ctrl_c2-2.jpg]

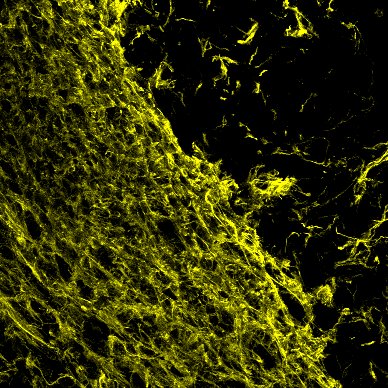

Supplement: Supplementary file 4 — Additional file 4. Uncropped gel and microscopy images. [file 13059_2023_3037_MOESM4_ESM.zip › Gel_Microscopy_images_GenomeBiology/microscopy_images/Figure 5/5b/fig5b_MAP2_TBR1_ctrl_c2-3.jpg]

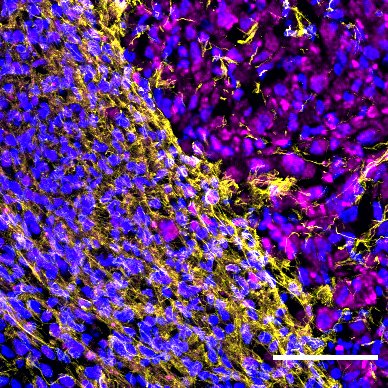

Supplement: Supplementary file 4 — Additional file 4. Uncropped gel and microscopy images. [file 13059_2023_3037_MOESM4_ESM.zip › Gel_Microscopy_images_GenomeBiology/microscopy_images/Figure 5/5b/fig5b_MAP2_TBR1_ctrl_c2-1.jpg]

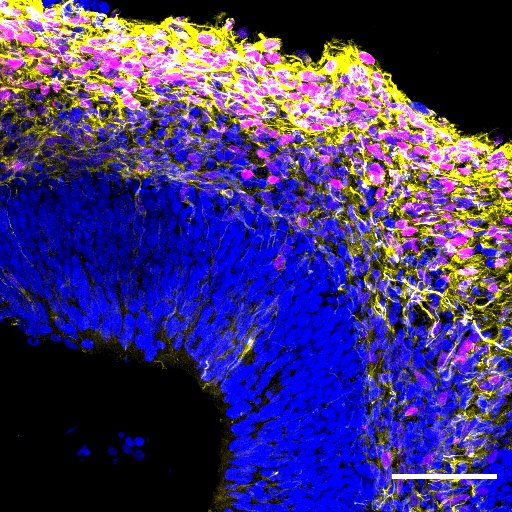

Supplement: Supplementary file 4 — Additional file 4. Uncropped gel and microscopy images. [file 13059_2023_3037_MOESM4_ESM.zip › Gel_Microscopy_images_GenomeBiology/microscopy_images/Figure 5/5b/fig5b_MAP2_TBR1_oxr1_c1-1.jpg]

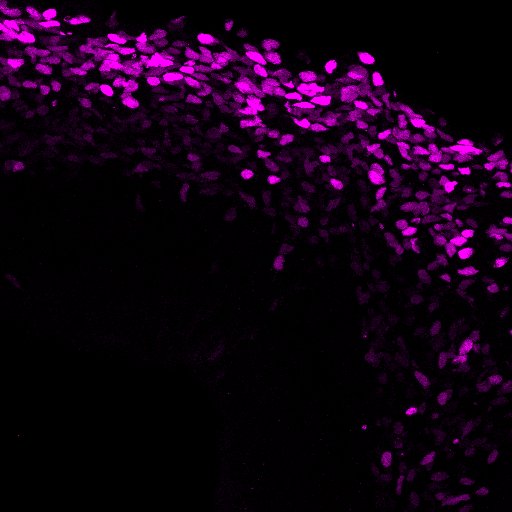

Supplement: Supplementary file 4 — Additional file 4. Uncropped gel and microscopy images. [file 13059_2023_3037_MOESM4_ESM.zip › Gel_Microscopy_images_GenomeBiology/microscopy_images/Figure 5/5b/fig5b_MAP2_TBR1_oxr1_c1-2.jpg]

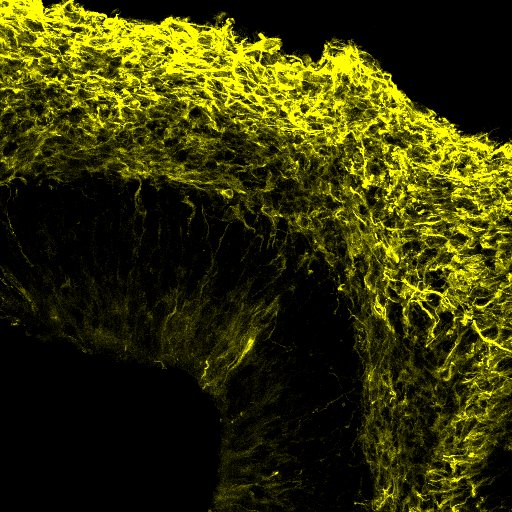

Supplement: Supplementary file 4 — Additional file 4. Uncropped gel and microscopy images. [file 13059_2023_3037_MOESM4_ESM.zip › Gel_Microscopy_images_GenomeBiology/microscopy_images/Figure 5/5b/fig5b_MAP2_TBR1_oxr1_c1-3.jpg]

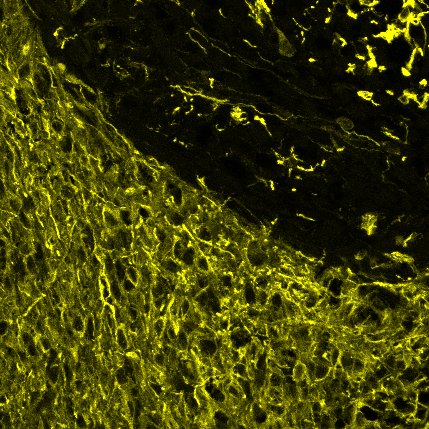

Supplement: Supplementary file 4 — Additional file 4. Uncropped gel and microscopy images. [file 13059_2023_3037_MOESM4_ESM.zip › Gel_Microscopy_images_GenomeBiology/microscopy_images/Figure 5/5b/fig5b_MAP2_TBR1_ctrl_c1-3.jpg]

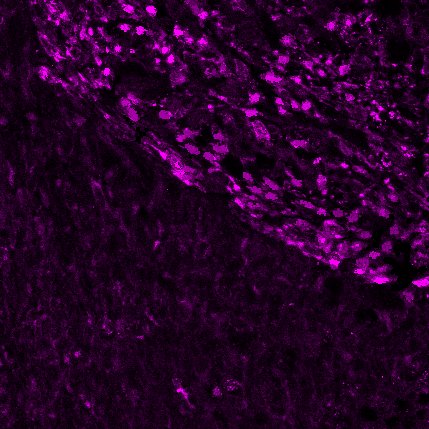

Supplement: Supplementary file 4 — Additional file 4. Uncropped gel and microscopy images. [file 13059_2023_3037_MOESM4_ESM.zip › Gel_Microscopy_images_GenomeBiology/microscopy_images/Figure 5/5b/fig5b_MAP2_TBR1_ctrl_c1-2.jpg]

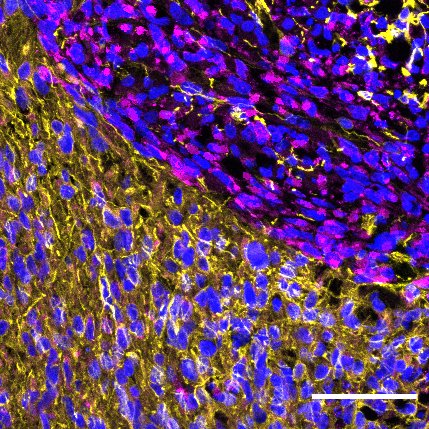

Supplement: Supplementary file 4 — Additional file 4. Uncropped gel and microscopy images. [file 13059_2023_3037_MOESM4_ESM.zip › Gel_Microscopy_images_GenomeBiology/microscopy_images/Figure 5/5b/fig5b_MAP2_TBR1_ctrl_c1-1.jpg]

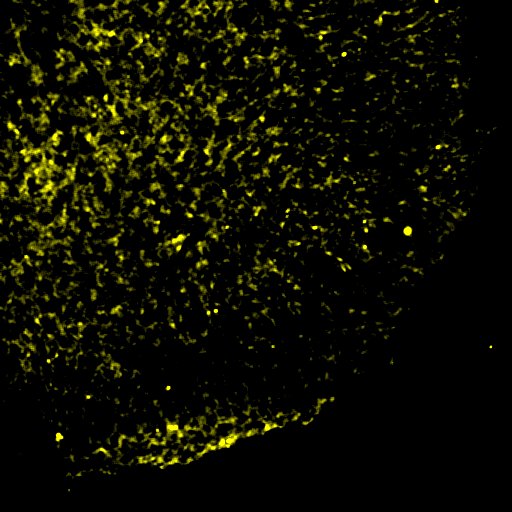

Supplement: Supplementary file 4 — Additional file 4. Uncropped gel and microscopy images. [file 13059_2023_3037_MOESM4_ESM.zip › Gel_Microscopy_images_GenomeBiology/microscopy_images/Figure S5/s5d/POMC_OTP/figS5d_OTP1_POMC_Ctrl_1-2.jpg]

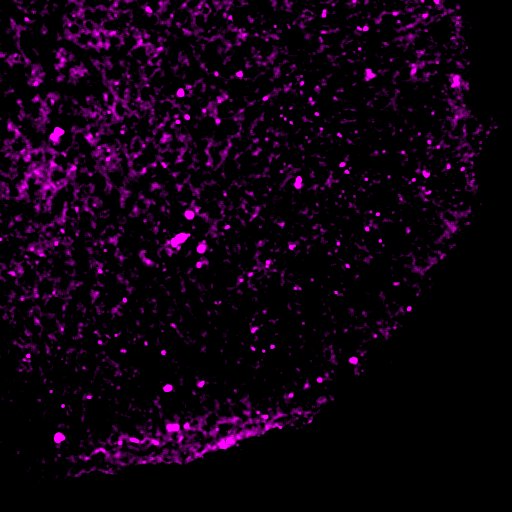

Supplement: Supplementary file 4 — Additional file 4. Uncropped gel and microscopy images. [file 13059_2023_3037_MOESM4_ESM.zip › Gel_Microscopy_images_GenomeBiology/microscopy_images/Figure S5/s5d/POMC_OTP/figS5d_OTP1_POMC_Ctrl_1-1.jpg]

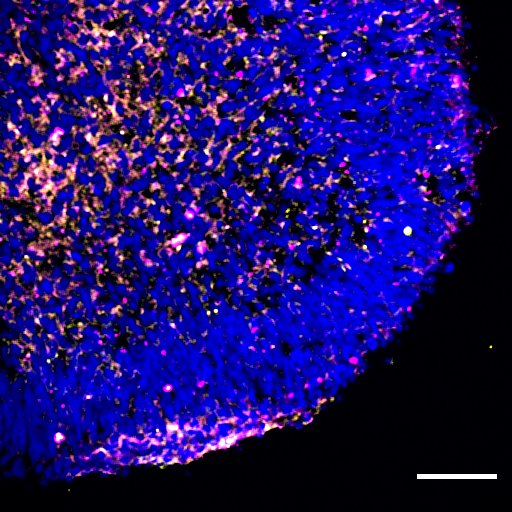

Supplement: Supplementary file 4 — Additional file 4. Uncropped gel and microscopy images. [file 13059_2023_3037_MOESM4_ESM.zip › Gel_Microscopy_images_GenomeBiology/microscopy_images/Figure S5/s5d/POMC_OTP/figS5d_OTP1_POMC_Ctrl_1.jpg]

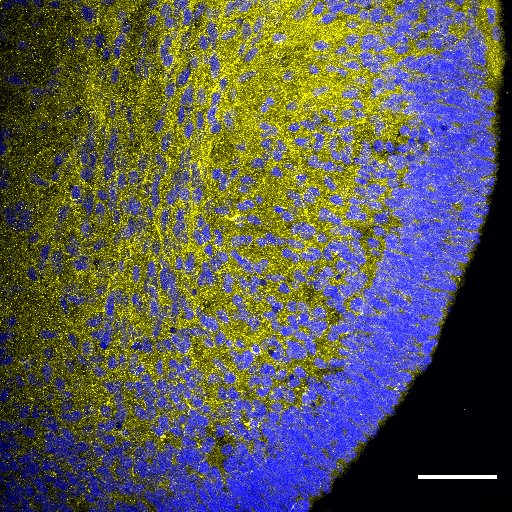

Supplement: Supplementary file 4 — Additional file 4. Uncropped gel and microscopy images. [file 13059_2023_3037_MOESM4_ESM.zip › Gel_Microscopy_images_GenomeBiology/microscopy_images/Figure S5/s5d/OXR1/s5d_OXR1_day50_1.jpg]

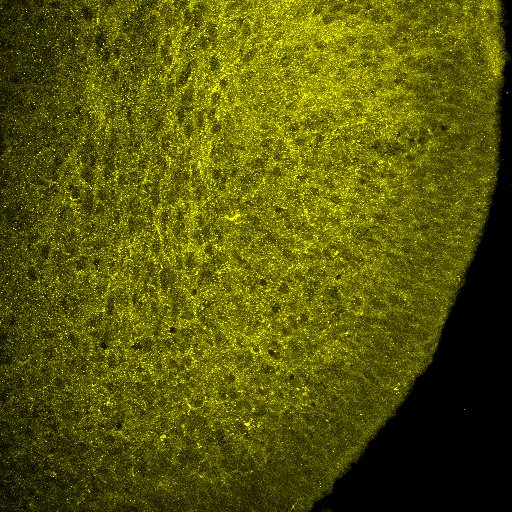

Supplement: Supplementary file 4 — Additional file 4. Uncropped gel and microscopy images. [file 13059_2023_3037_MOESM4_ESM.zip › Gel_Microscopy_images_GenomeBiology/microscopy_images/Figure S5/s5d/OXR1/s5d_OXR1_day50_2.jpg]

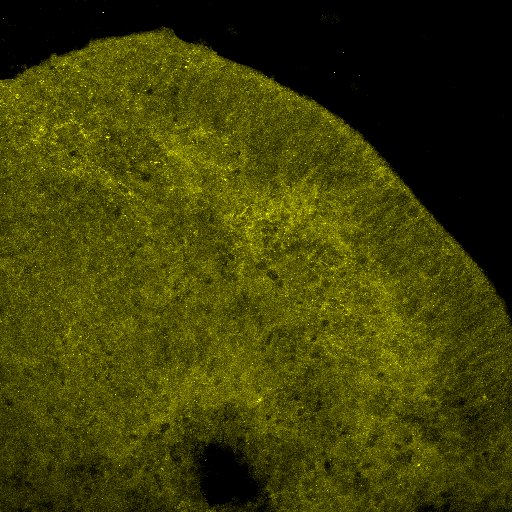

Supplement: Supplementary file 4 — Additional file 4. Uncropped gel and microscopy images. [file 13059_2023_3037_MOESM4_ESM.zip › Gel_Microscopy_images_GenomeBiology/microscopy_images/Figure S5/s5d/OXR1/s5d_OXR1_day36_2.jpg]

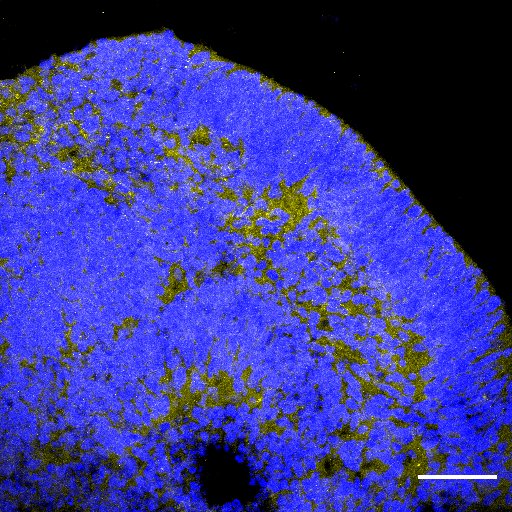

Supplement: Supplementary file 4 — Additional file 4. Uncropped gel and microscopy images. [file 13059_2023_3037_MOESM4_ESM.zip › Gel_Microscopy_images_GenomeBiology/microscopy_images/Figure S5/s5d/OXR1/s5d_OXR1_day36_1.jpg]

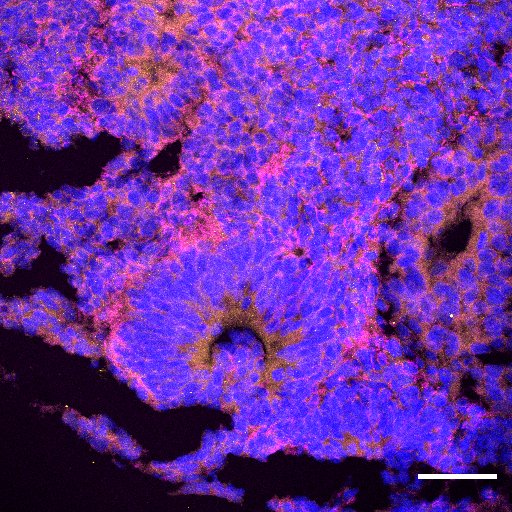

Supplement: Supplementary file 4 — Additional file 4. Uncropped gel and microscopy images. [file 13059_2023_3037_MOESM4_ESM.zip › Gel_Microscopy_images_GenomeBiology/microscopy_images/Figure S5/s5d/OXR1/s5d_OXR1_day20_1.jpg]

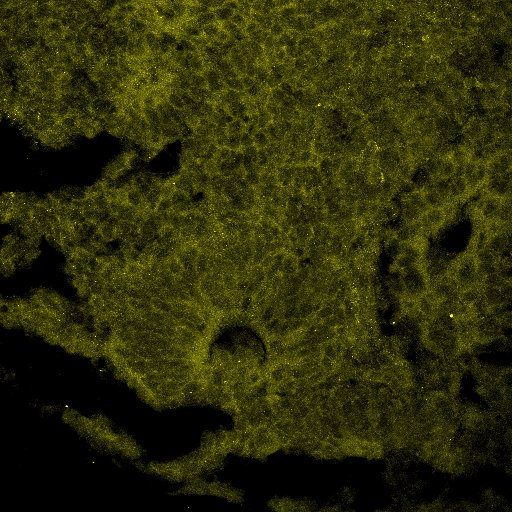

Supplement: Supplementary file 4 — Additional file 4. Uncropped gel and microscopy images. [file 13059_2023_3037_MOESM4_ESM.zip › Gel_Microscopy_images_GenomeBiology/microscopy_images/Figure S5/s5d/OXR1/s5d_OXR1_day20_2.jpg]

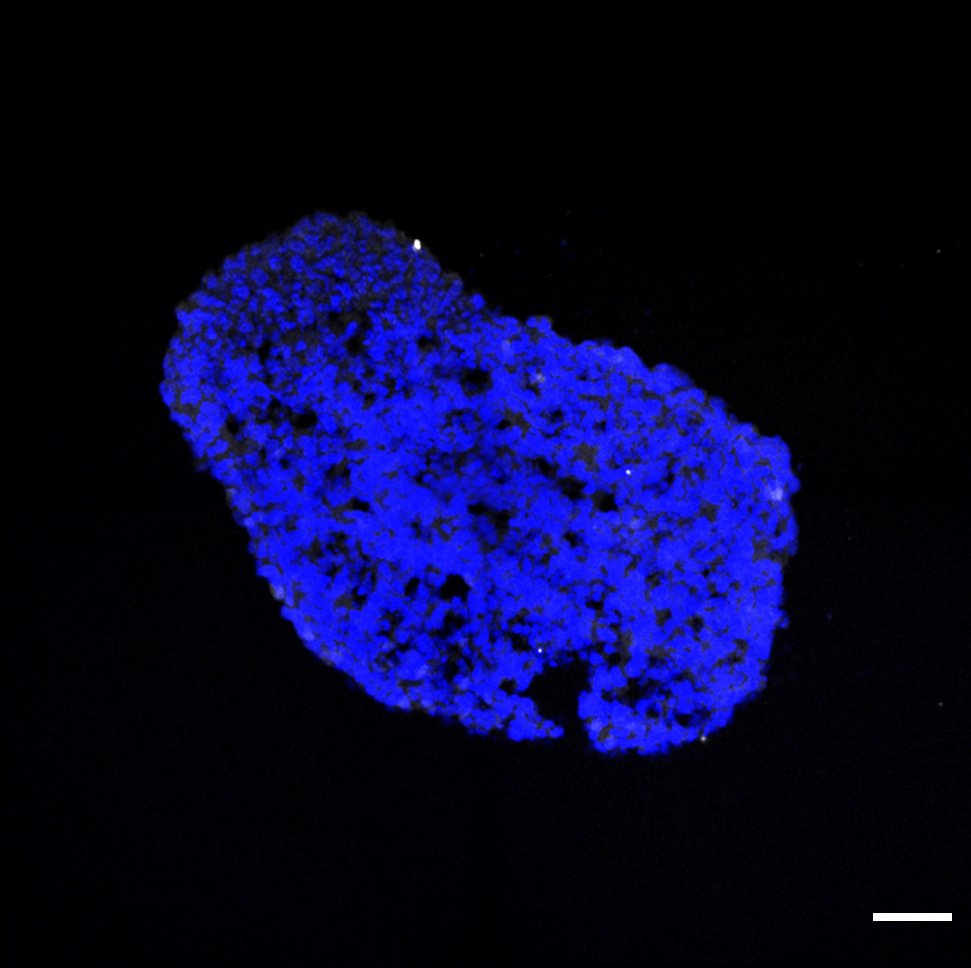

Supplement: Supplementary file 4 — Additional file 4. Uncropped gel and microscopy images. [file 13059_2023_3037_MOESM4_ESM.zip › Gel_Microscopy_images_GenomeBiology/microscopy_images/Figure 5/5h/5h-NeuN/fig5h_NeuN_day20_oxr1_c1.jpg]

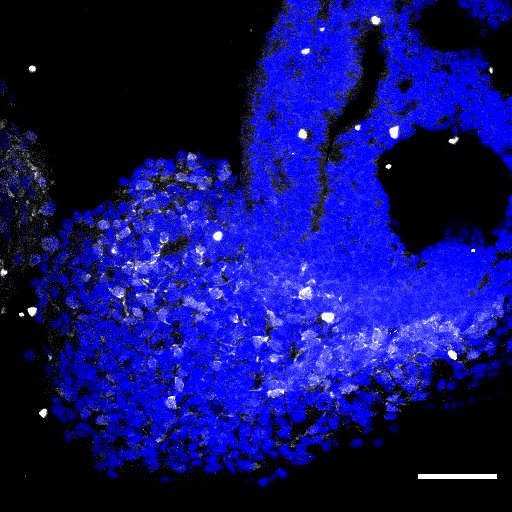

Supplement: Supplementary file 4 — Additional file 4. Uncropped gel and microscopy images. [file 13059_2023_3037_MOESM4_ESM.zip › Gel_Microscopy_images_GenomeBiology/microscopy_images/Figure 5/5h/5h-NeuN/fig5h_NeuN_day50_oxr1_c1.jpg]

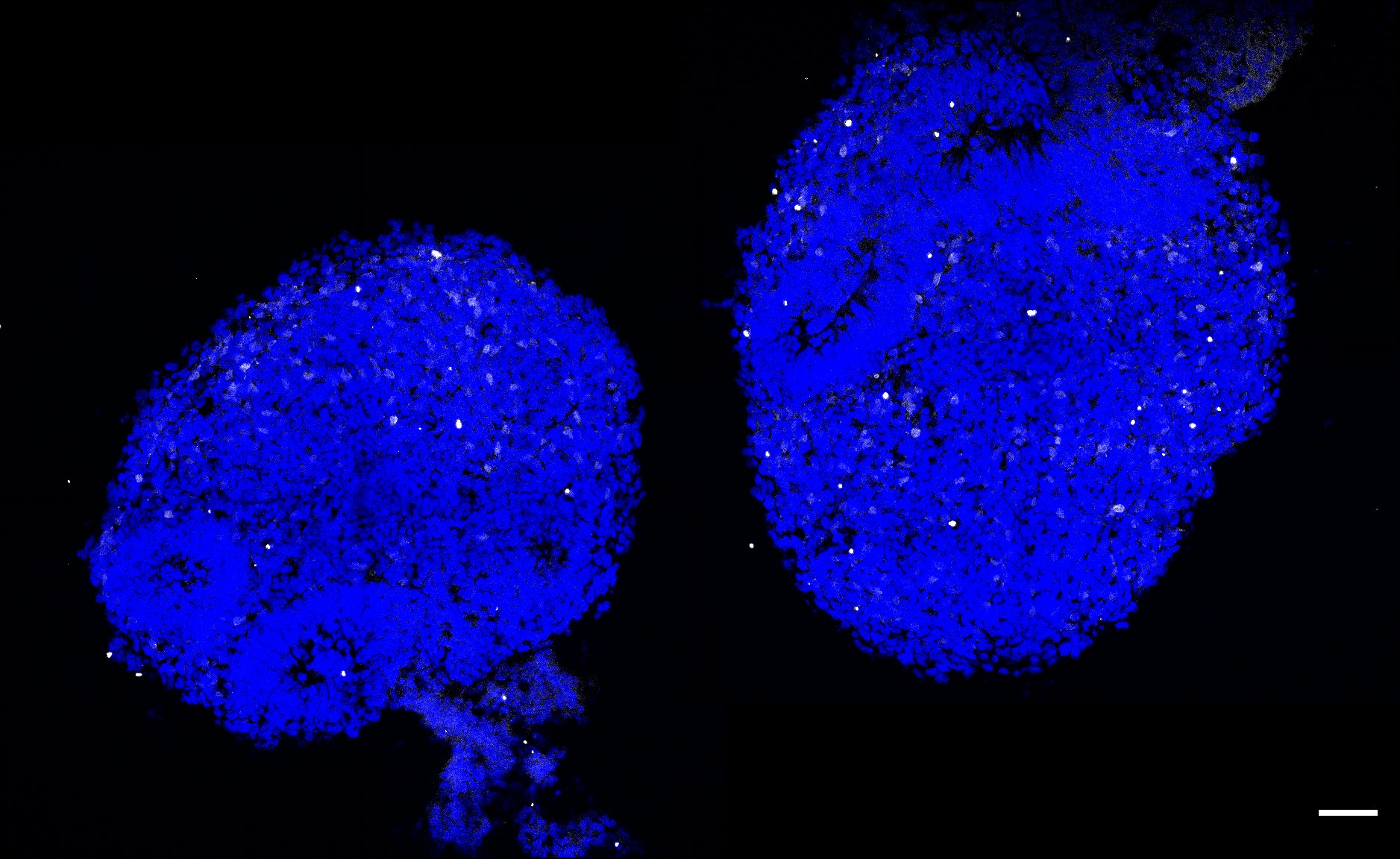

Supplement: Supplementary file 4 — Additional file 4. Uncropped gel and microscopy images. [file 13059_2023_3037_MOESM4_ESM.zip › Gel_Microscopy_images_GenomeBiology/microscopy_images/Figure 5/5h/5h-NeuN/fig5h_NeuN_day20_ctrl_c1.jpg]

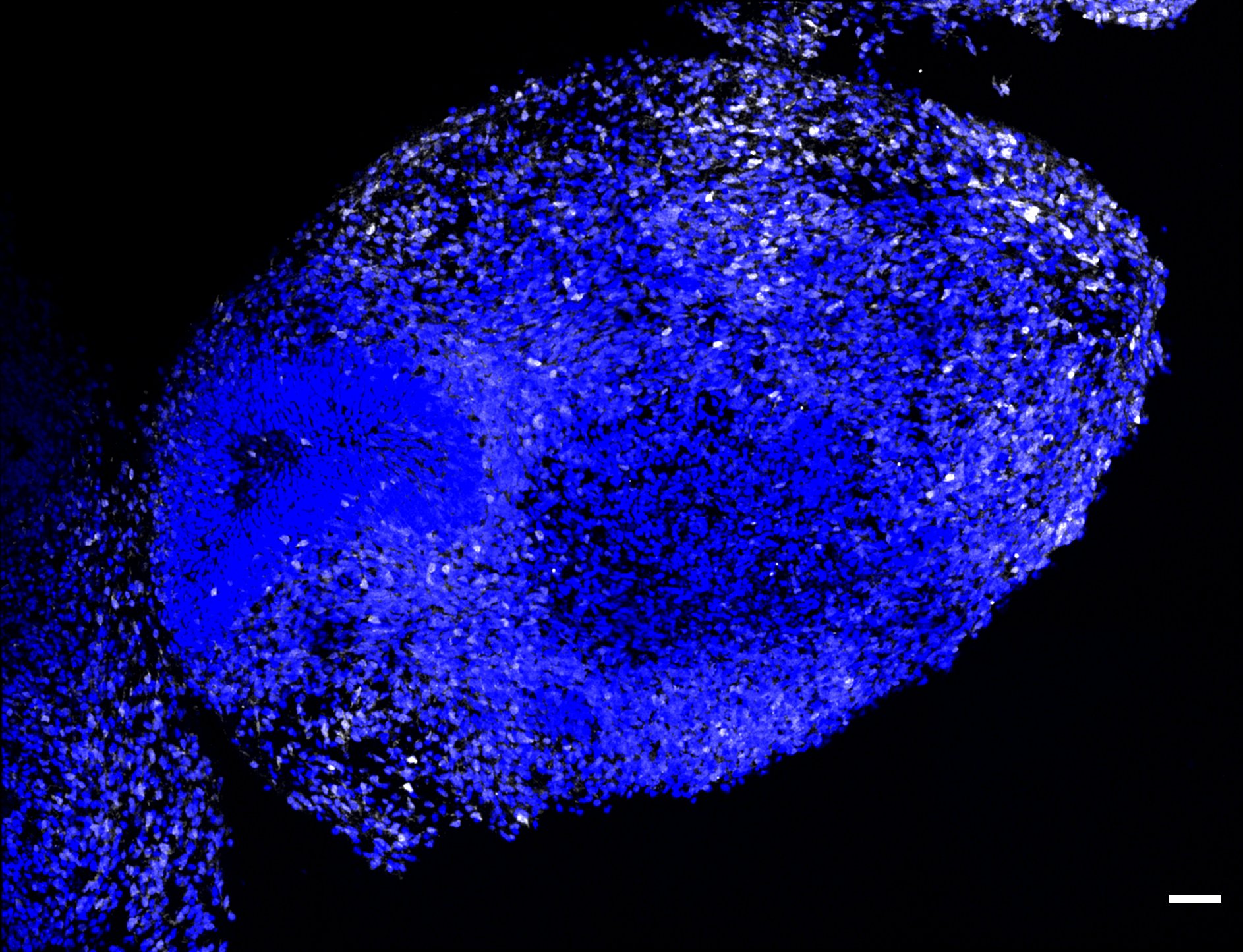

Supplement: Supplementary file 4 — Additional file 4. Uncropped gel and microscopy images. [file 13059_2023_3037_MOESM4_ESM.zip › Gel_Microscopy_images_GenomeBiology/microscopy_images/Figure 5/5h/5h-NeuN/fig5h_NeuN_day50_ctrl_c1.jpg]

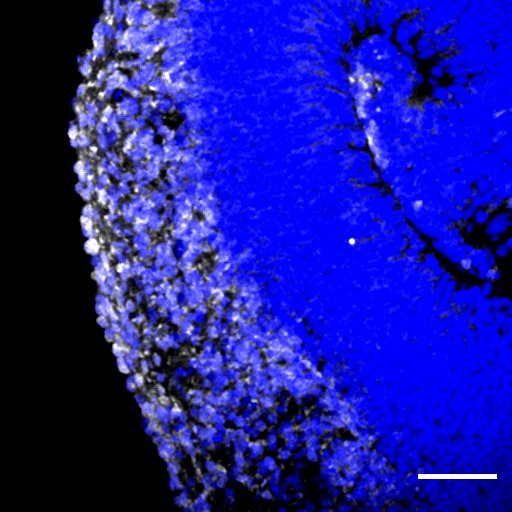

Supplement: Supplementary file 4 — Additional file 4. Uncropped gel and microscopy images. [file 13059_2023_3037_MOESM4_ESM.zip › Gel_Microscopy_images_GenomeBiology/microscopy_images/Figure 5/5h/5h-NeuN/fig5h_NeuN_day59_ctrl_c1.jpg]

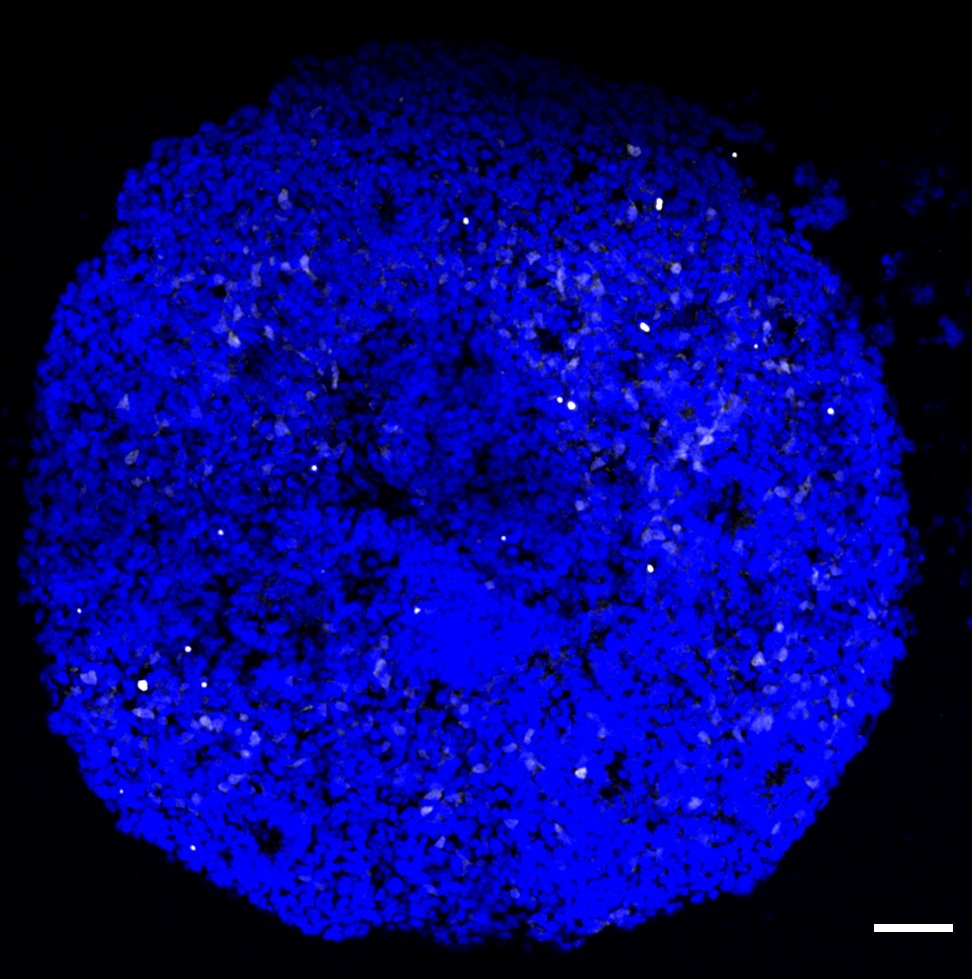

Supplement: Supplementary file 4 — Additional file 4. Uncropped gel and microscopy images. [file 13059_2023_3037_MOESM4_ESM.zip › Gel_Microscopy_images_GenomeBiology/microscopy_images/Figure 5/5h/5h-NeuN/fig5h_NeuN_day20_ctrl_c2.jpg]

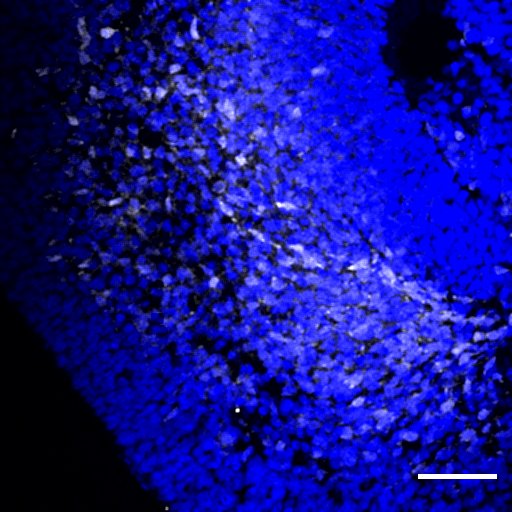

Supplement: Supplementary file 4 — Additional file 4. Uncropped gel and microscopy images. [file 13059_2023_3037_MOESM4_ESM.zip › Gel_Microscopy_images_GenomeBiology/microscopy_images/Figure 5/5h/5h-NeuN/fig5h_NeuN_day50_ctrl_c2.jpg]

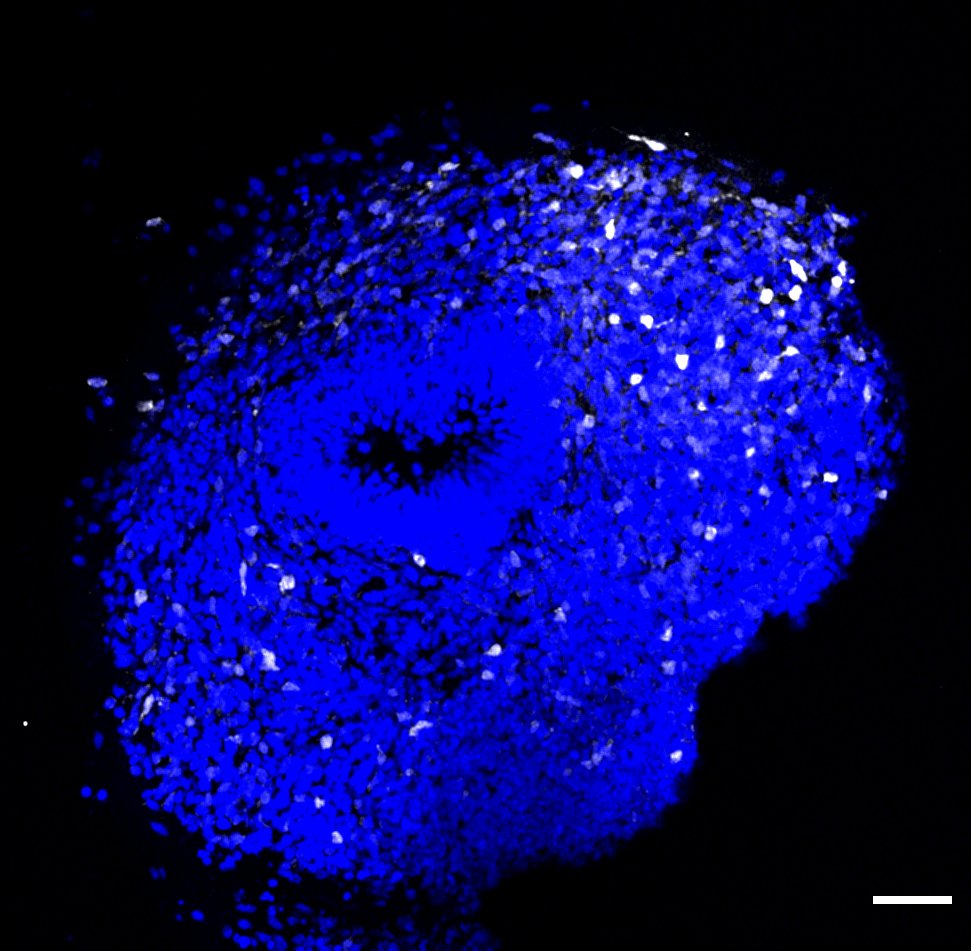

Supplement: Supplementary file 4 — Additional file 4. Uncropped gel and microscopy images. [file 13059_2023_3037_MOESM4_ESM.zip › Gel_Microscopy_images_GenomeBiology/microscopy_images/Figure 5/5h/5h-NeuN/fig5h_NeuN_day65_oxr1_c1.jpg]

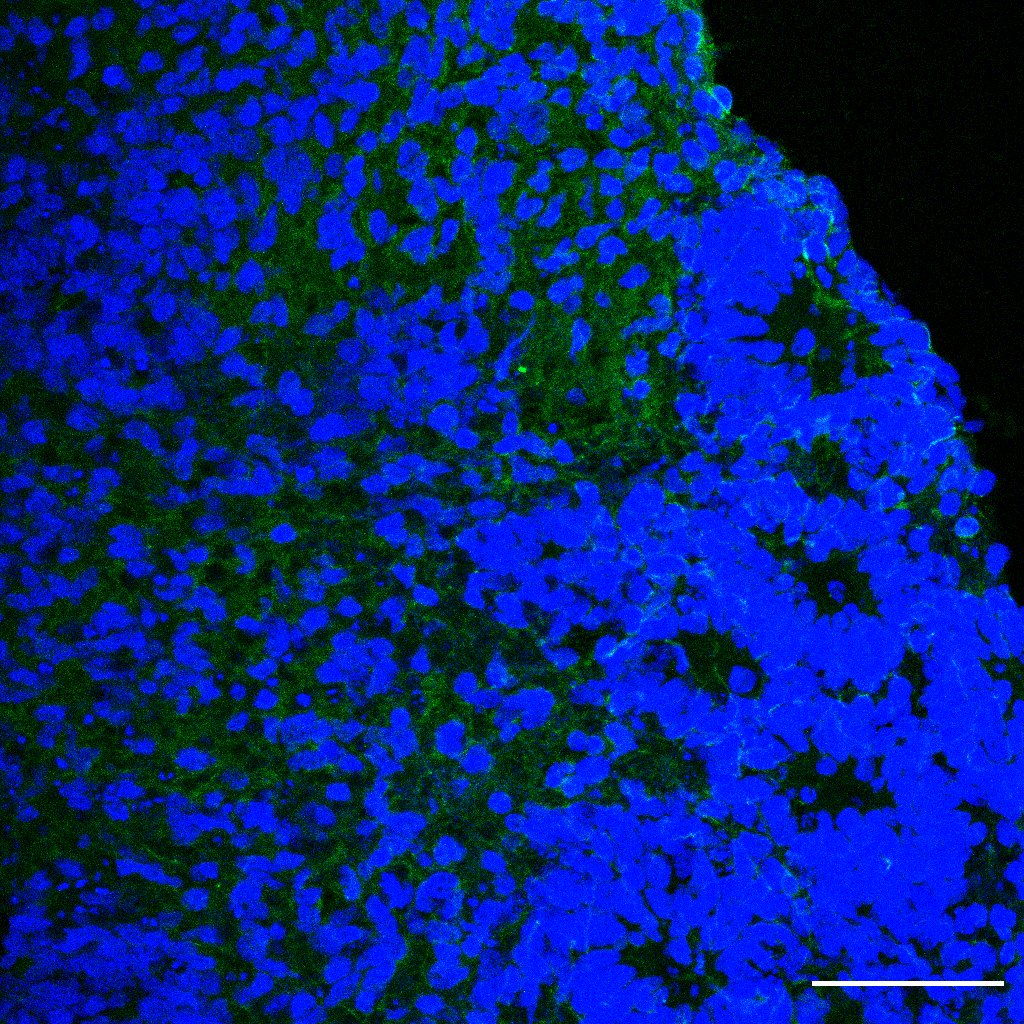

Supplement: Supplementary file 4 — Additional file 4. Uncropped gel and microscopy images. [file 13059_2023_3037_MOESM4_ESM.zip › Gel_Microscopy_images_GenomeBiology/microscopy_images/Figure 5/5h/5h-DCX/fig5h_DCX_day50_oxr1_c1.jpg]

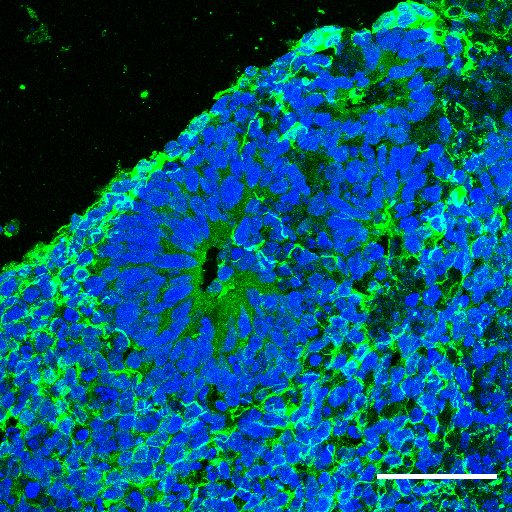

Supplement: Supplementary file 4 — Additional file 4. Uncropped gel and microscopy images. [file 13059_2023_3037_MOESM4_ESM.zip › Gel_Microscopy_images_GenomeBiology/microscopy_images/Figure 5/5h/5h-DCX/fig5h_DCX_day50_ctrl_c1.jpg]

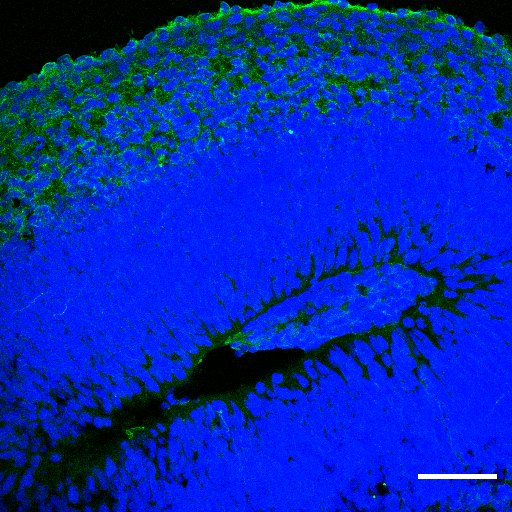

Supplement: Supplementary file 4 — Additional file 4. Uncropped gel and microscopy images. [file 13059_2023_3037_MOESM4_ESM.zip › Gel_Microscopy_images_GenomeBiology/microscopy_images/Figure 5/5h/5h-DCX/fig5h_DCX_day59_ctrl_c1.jpg]

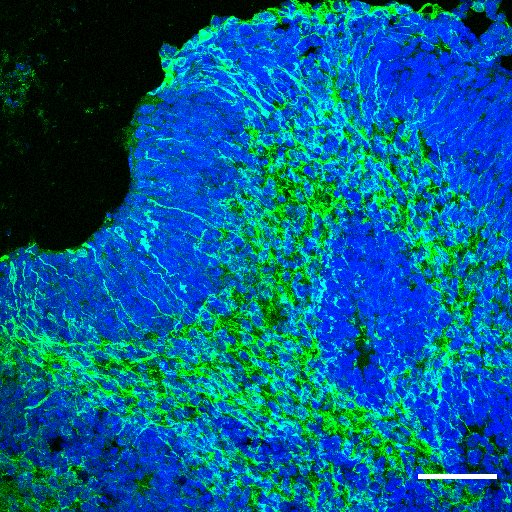

Supplement: Supplementary file 4 — Additional file 4. Uncropped gel and microscopy images. [file 13059_2023_3037_MOESM4_ESM.zip › Gel_Microscopy_images_GenomeBiology/microscopy_images/Figure 5/5h/5h-DCX/fig5h_DCX_day50_ctrl_c2.jpg]

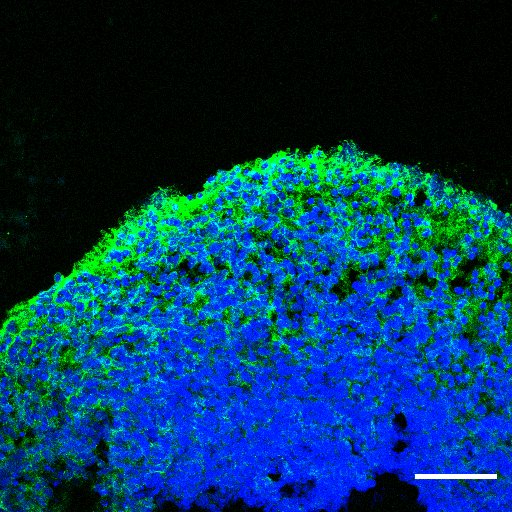

Supplement: Supplementary file 4 — Additional file 4. Uncropped gel and microscopy images. [file 13059_2023_3037_MOESM4_ESM.zip › Gel_Microscopy_images_GenomeBiology/microscopy_images/Figure 5/5h/5h-DCX/fig5h_DCX_day65_oxr1_c1.jpg]
