# Supplementary figures and images for: A loss-of-function mutation in human Oxidation Resistance 1 disrupts the spatial–temporal regulation of histone arginine methylation in neurodevelopment (part 1 of 2)
Source: Genome Biol. 2023 Sep 29;24:216. doi: 10.1186/s13059-023-03037-1 (PMC10540402; doi:10.1186/s13059-023-03037-1)

## Slide 1
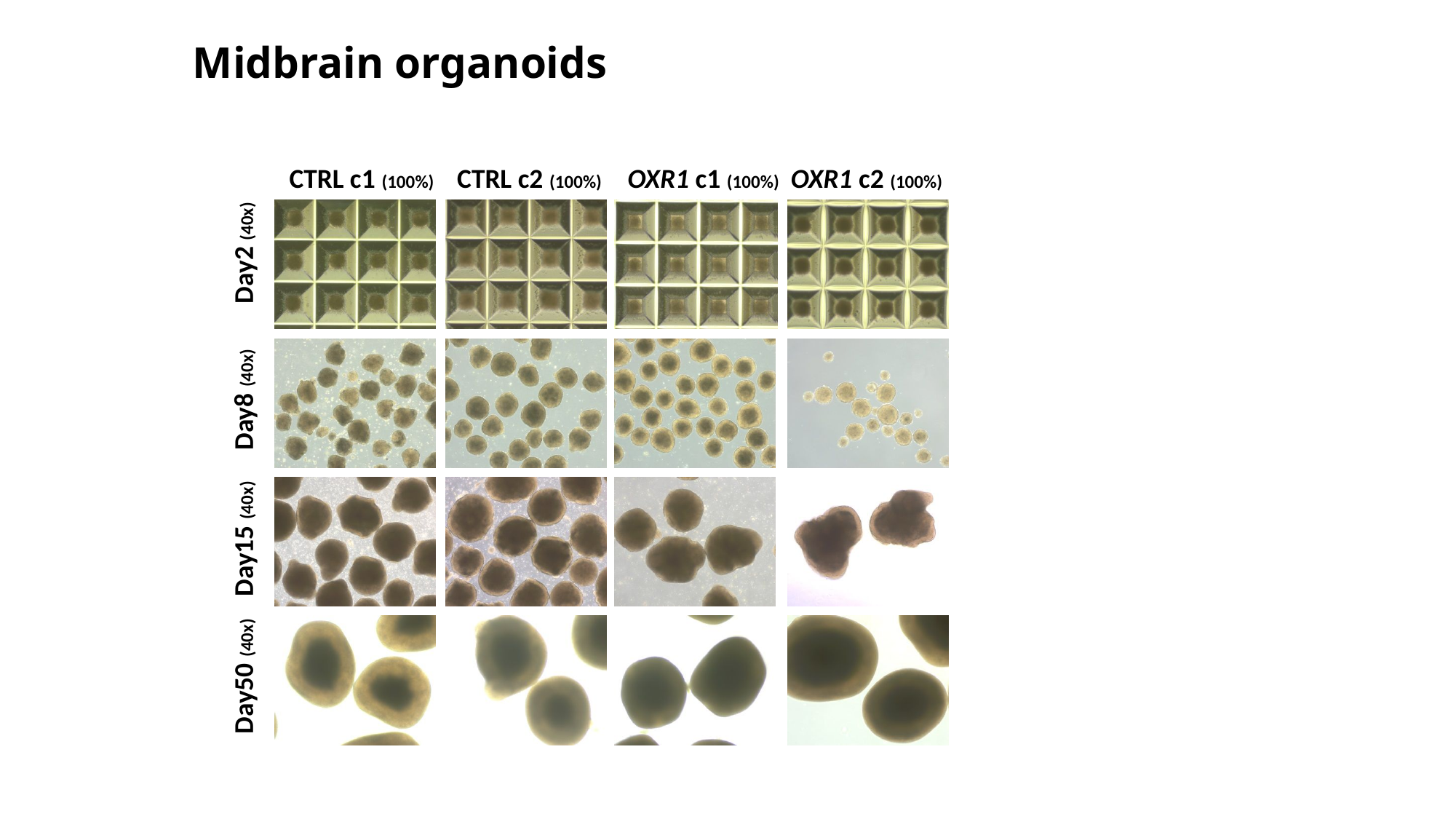

# Midbrain organoids
CTRL c1 (100%)
CTRL c2 (100%)
OXR1 c1 (100%)
OXR1 c2 (100%)
Day2 (40x)
Day8 (40x)
Day15 (40x)
Day50 (40x)

Supplement: Supplementary file 4 — Additional file 4. Uncropped gel and microscopy images. [file 13059_2023_3037_MOESM4_ESM.zip › Gel_Microscopy_images_GenomeBiology/microscopy_images/Figure 7/Fig7a.pptx]

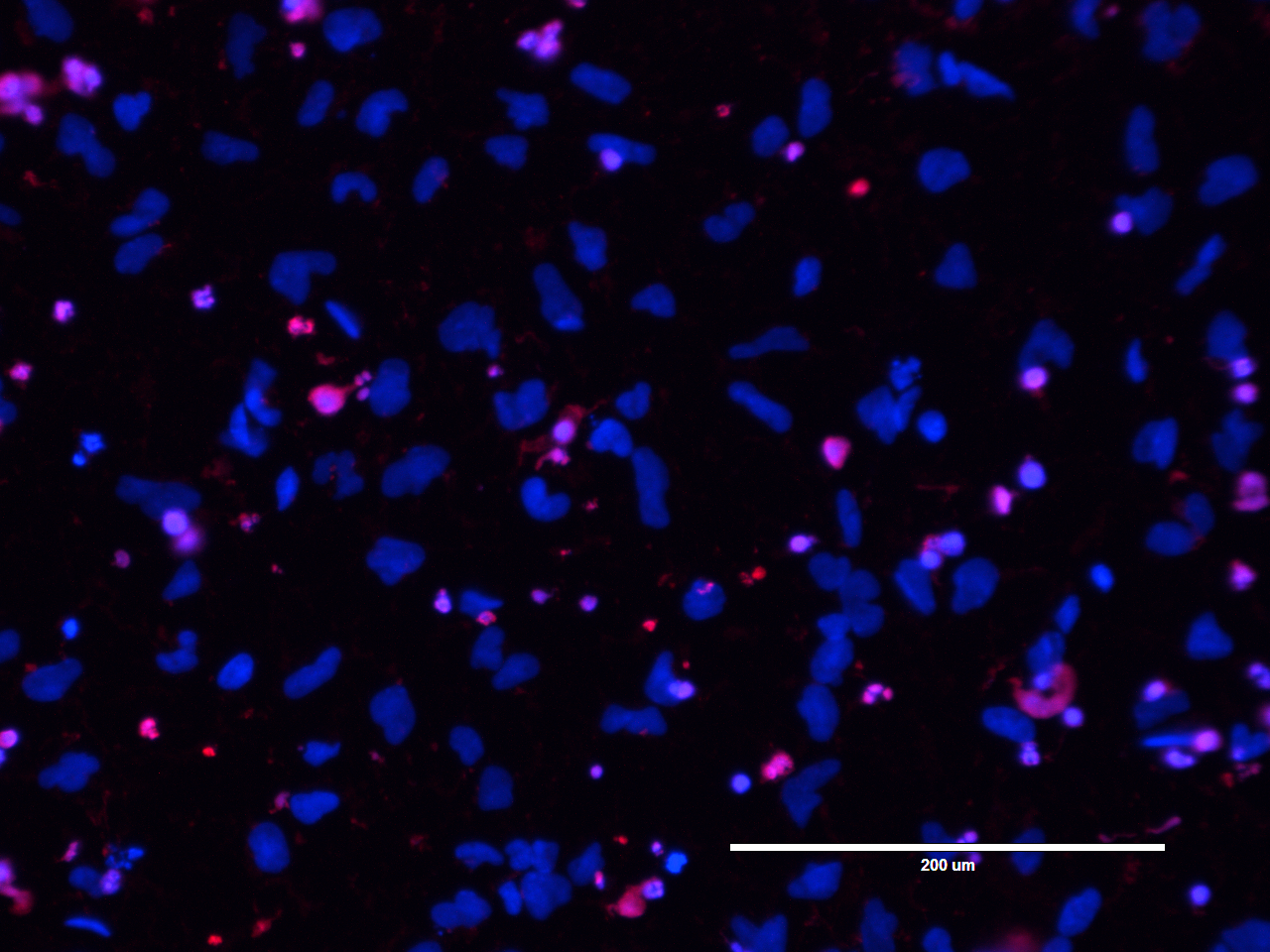

Supplement: Supplementary file 4 — Additional file 4. Uncropped gel and microscopy images. [file 13059_2023_3037_MOESM4_ESM.zip › Gel_Microscopy_images_GenomeBiology/microscopy_images/Figure S3/S3_k_Apoptosis_ctrl.tif]

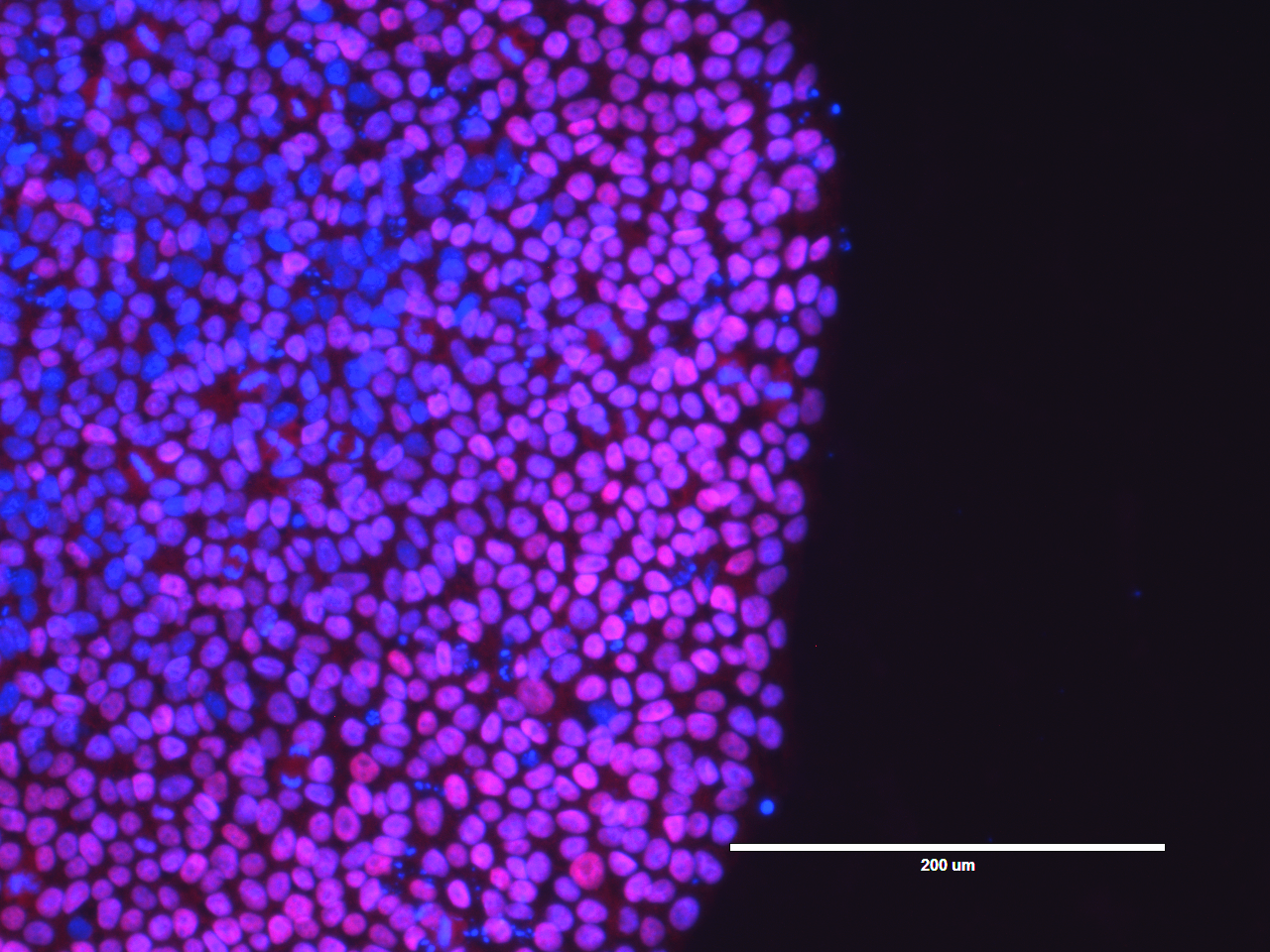

Supplement: Supplementary file 4 — Additional file 4. Uncropped gel and microscopy images. [file 13059_2023_3037_MOESM4_ESM.zip › Gel_Microscopy_images_GenomeBiology/microscopy_images/Figure S3/S3_d_Oct4_Oxr.tif]

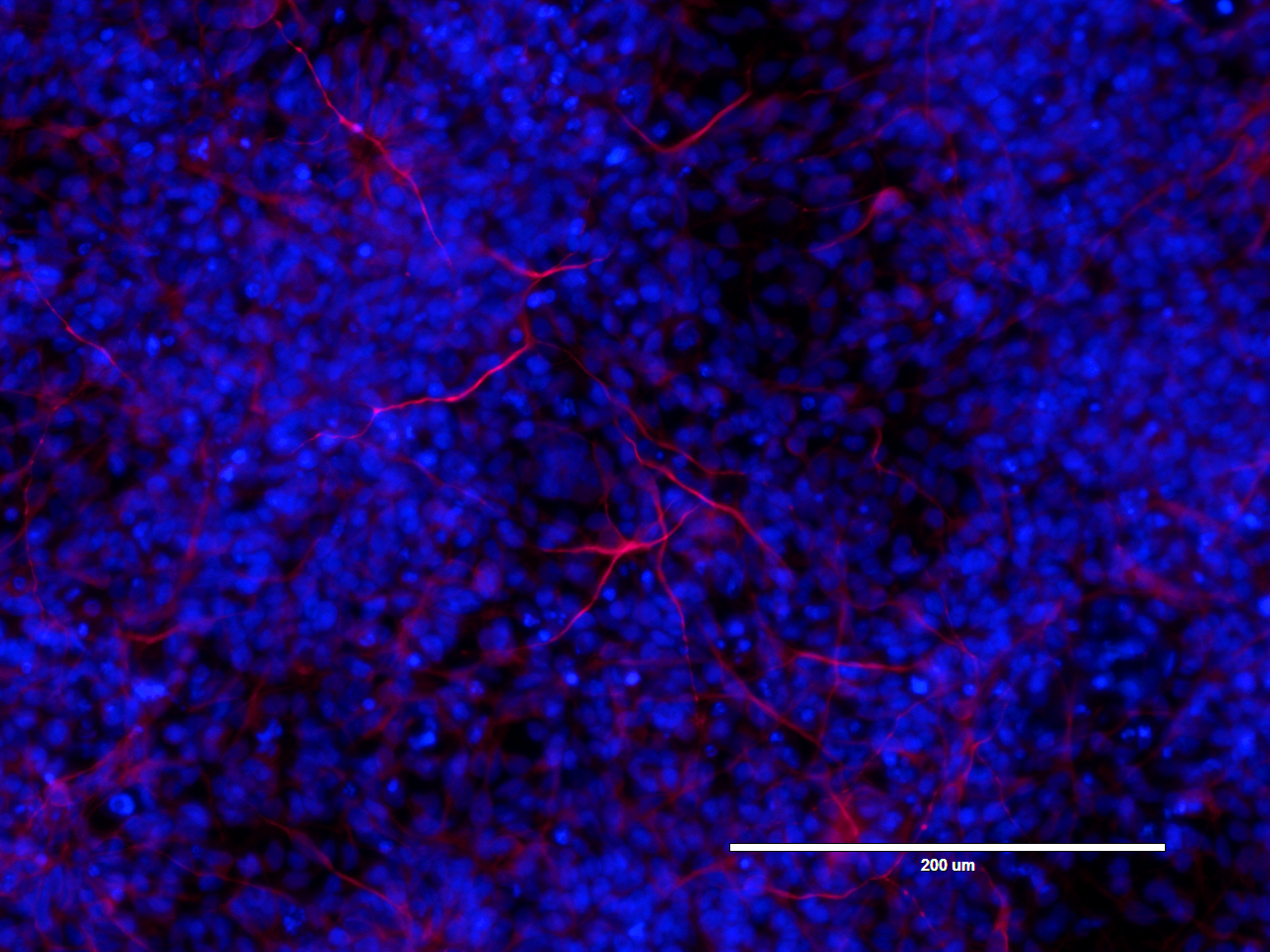

Supplement: Supplementary file 4 — Additional file 4. Uncropped gel and microscopy images. [file 13059_2023_3037_MOESM4_ESM.zip › Gel_Microscopy_images_GenomeBiology/microscopy_images/Figure S3/S3_j_Map2_Oxr.tif]

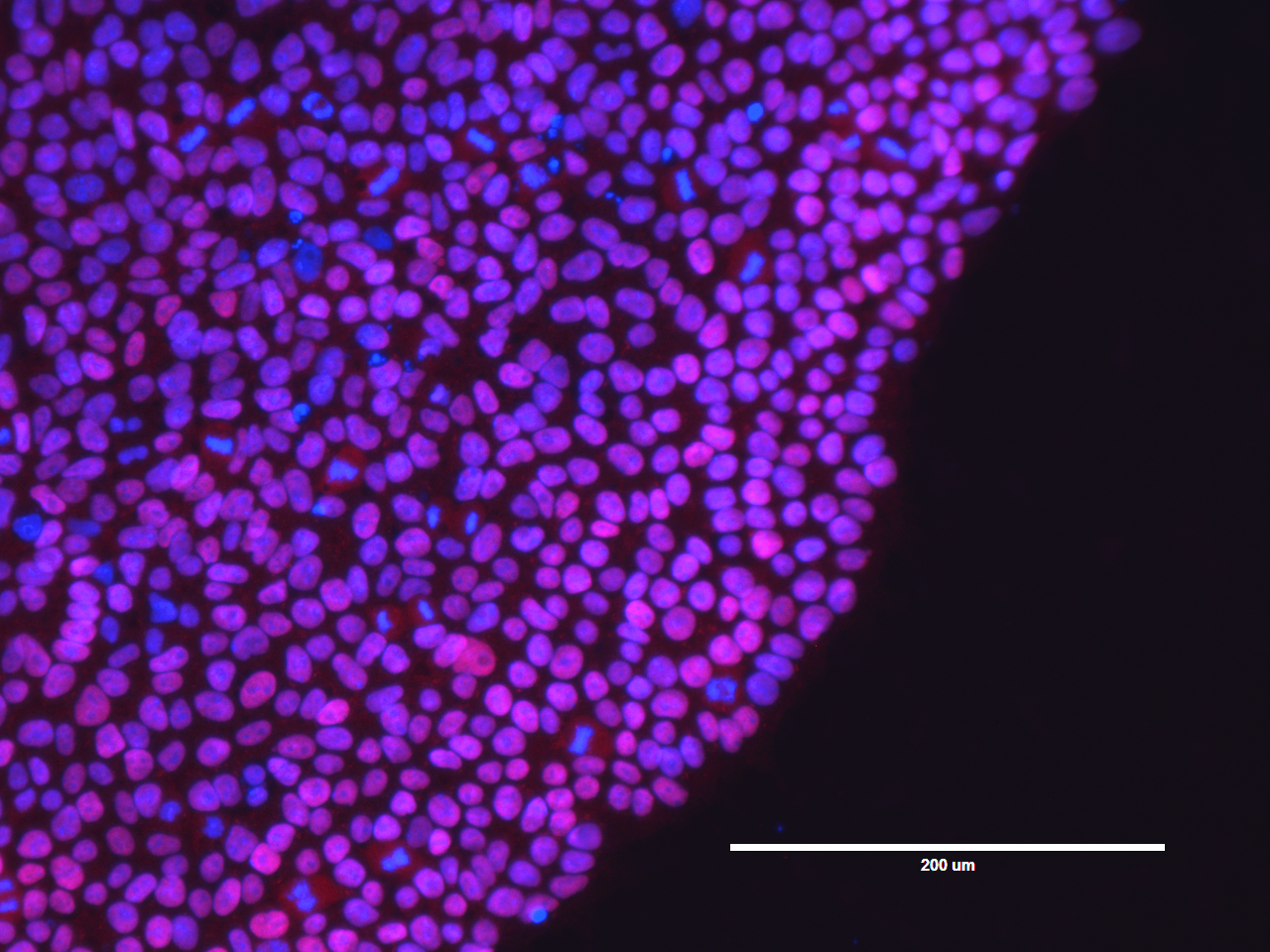

Supplement: Supplementary file 4 — Additional file 4. Uncropped gel and microscopy images. [file 13059_2023_3037_MOESM4_ESM.zip › Gel_Microscopy_images_GenomeBiology/microscopy_images/Figure S3/S3_d_Sox2_ctrl.tif]

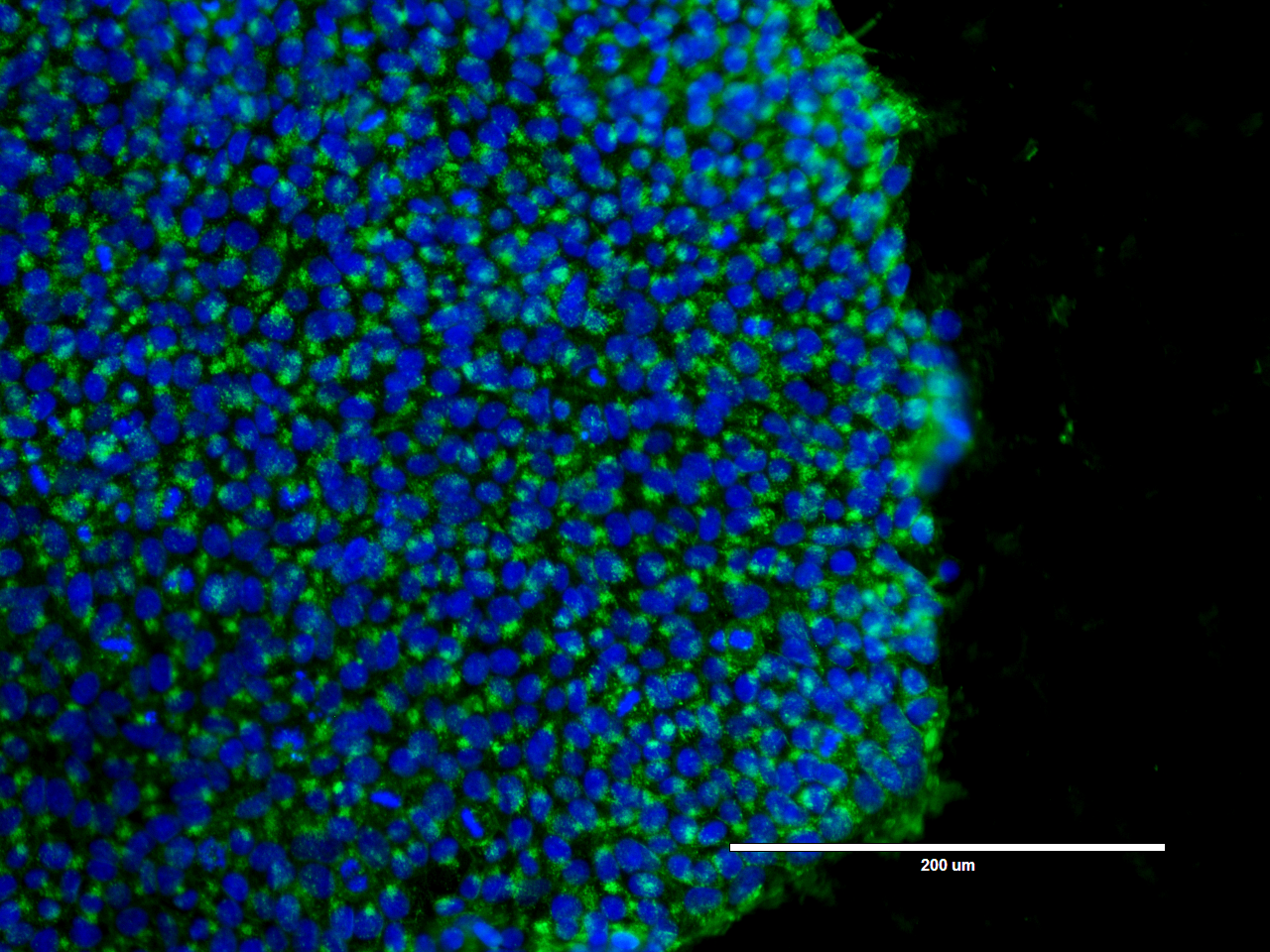

Supplement: Supplementary file 4 — Additional file 4. Uncropped gel and microscopy images. [file 13059_2023_3037_MOESM4_ESM.zip › Gel_Microscopy_images_GenomeBiology/microscopy_images/Figure S3/S3_d_SSEA4_Oxr1.tif]

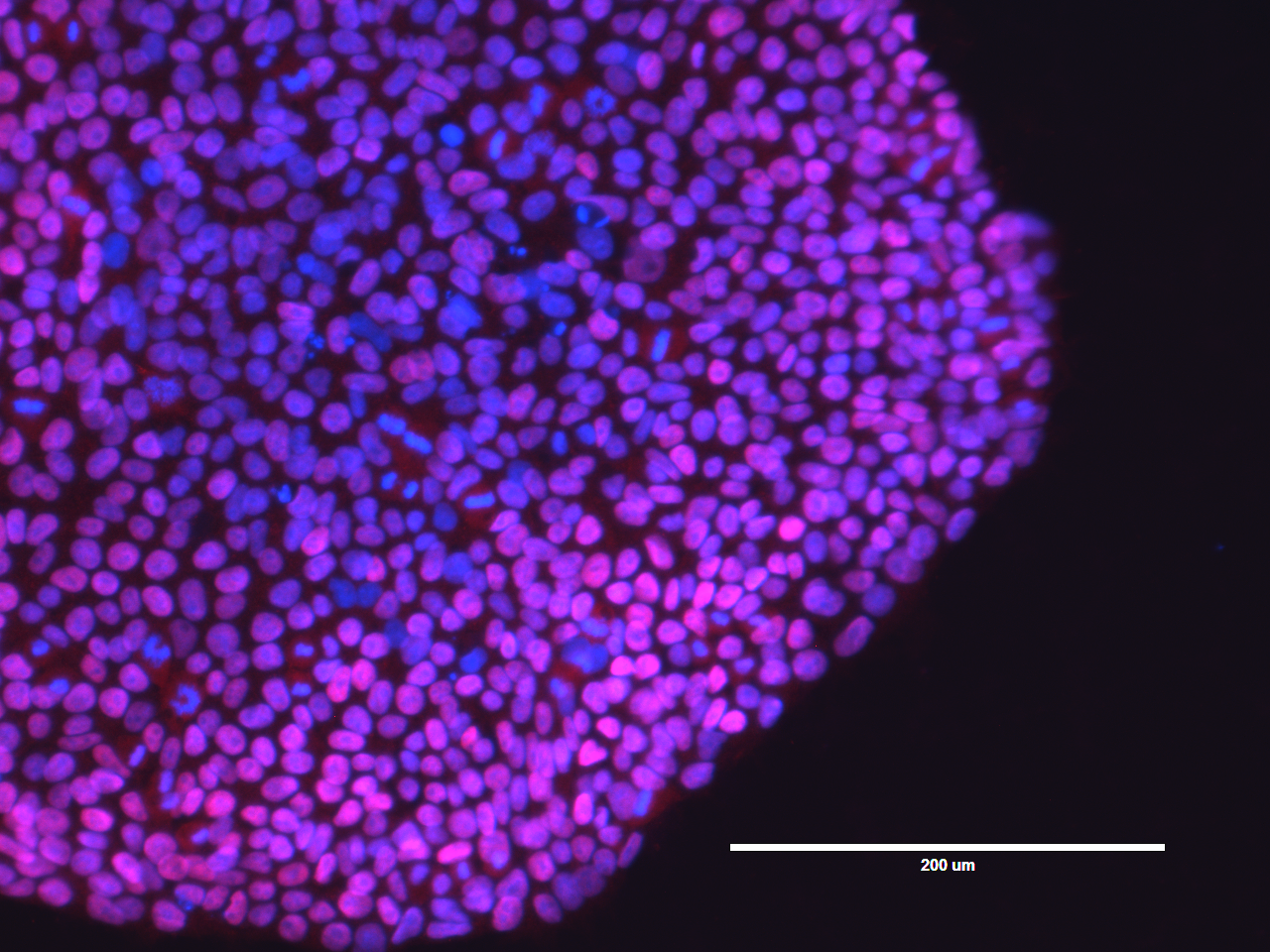

Supplement: Supplementary file 4 — Additional file 4. Uncropped gel and microscopy images. [file 13059_2023_3037_MOESM4_ESM.zip › Gel_Microscopy_images_GenomeBiology/microscopy_images/Figure S3/S3_d_Oct4_ctrl.tif]

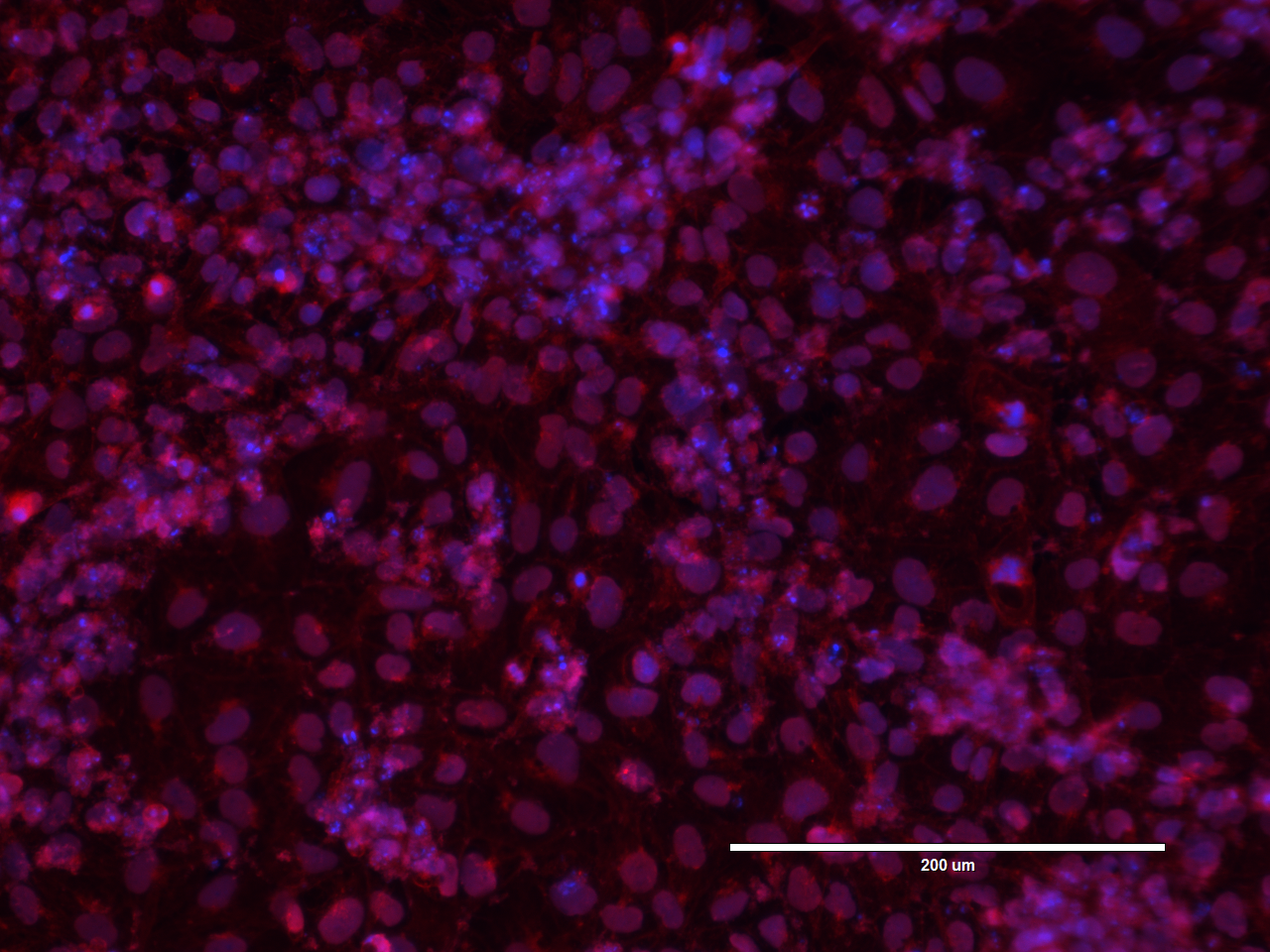

Supplement: Supplementary file 4 — Additional file 4. Uncropped gel and microscopy images. [file 13059_2023_3037_MOESM4_ESM.zip › Gel_Microscopy_images_GenomeBiology/microscopy_images/Figure S3/S3_f_Sox17_Oxr1.tif]

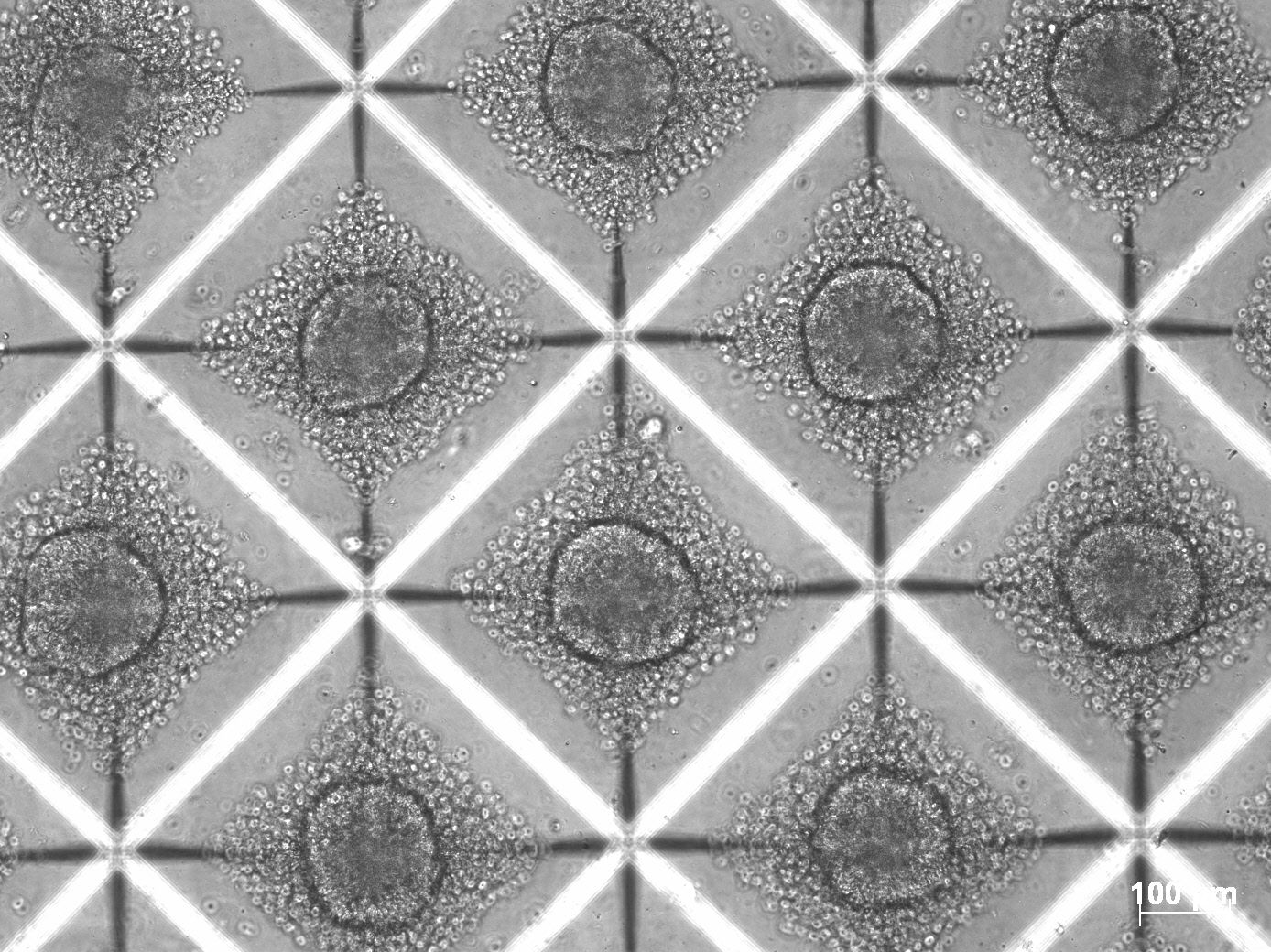

Supplement: Supplementary file 4 — Additional file 4. Uncropped gel and microscopy images. [file 13059_2023_3037_MOESM4_ESM.zip › Gel_Microscopy_images_GenomeBiology/microscopy_images/Figure S3/S3_h_EB_ctrl.jpg]

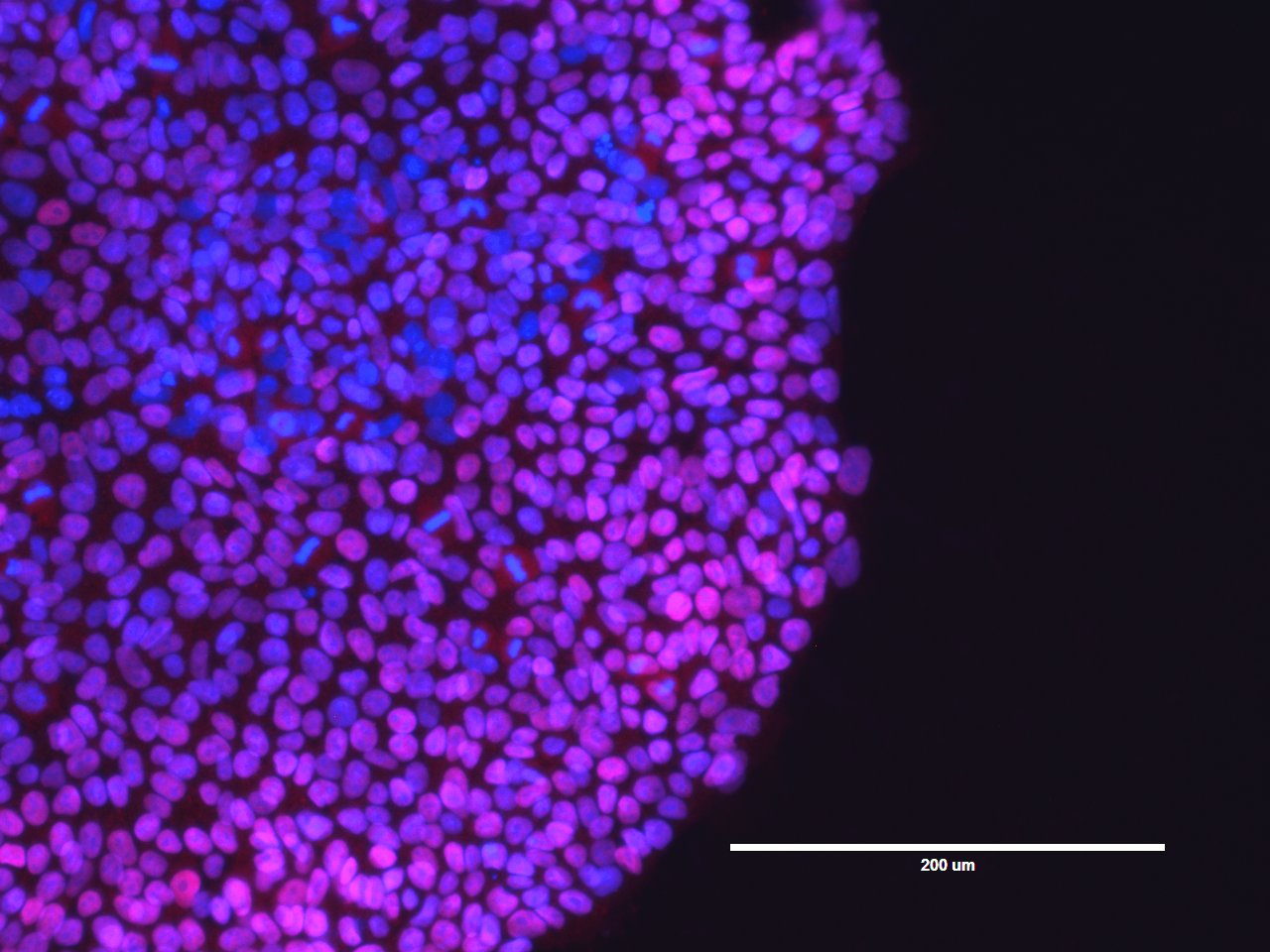

Supplement: Supplementary file 4 — Additional file 4. Uncropped gel and microscopy images. [file 13059_2023_3037_MOESM4_ESM.zip › Gel_Microscopy_images_GenomeBiology/microscopy_images/Figure S3/S3_d_NANOG_Oxr.tif]

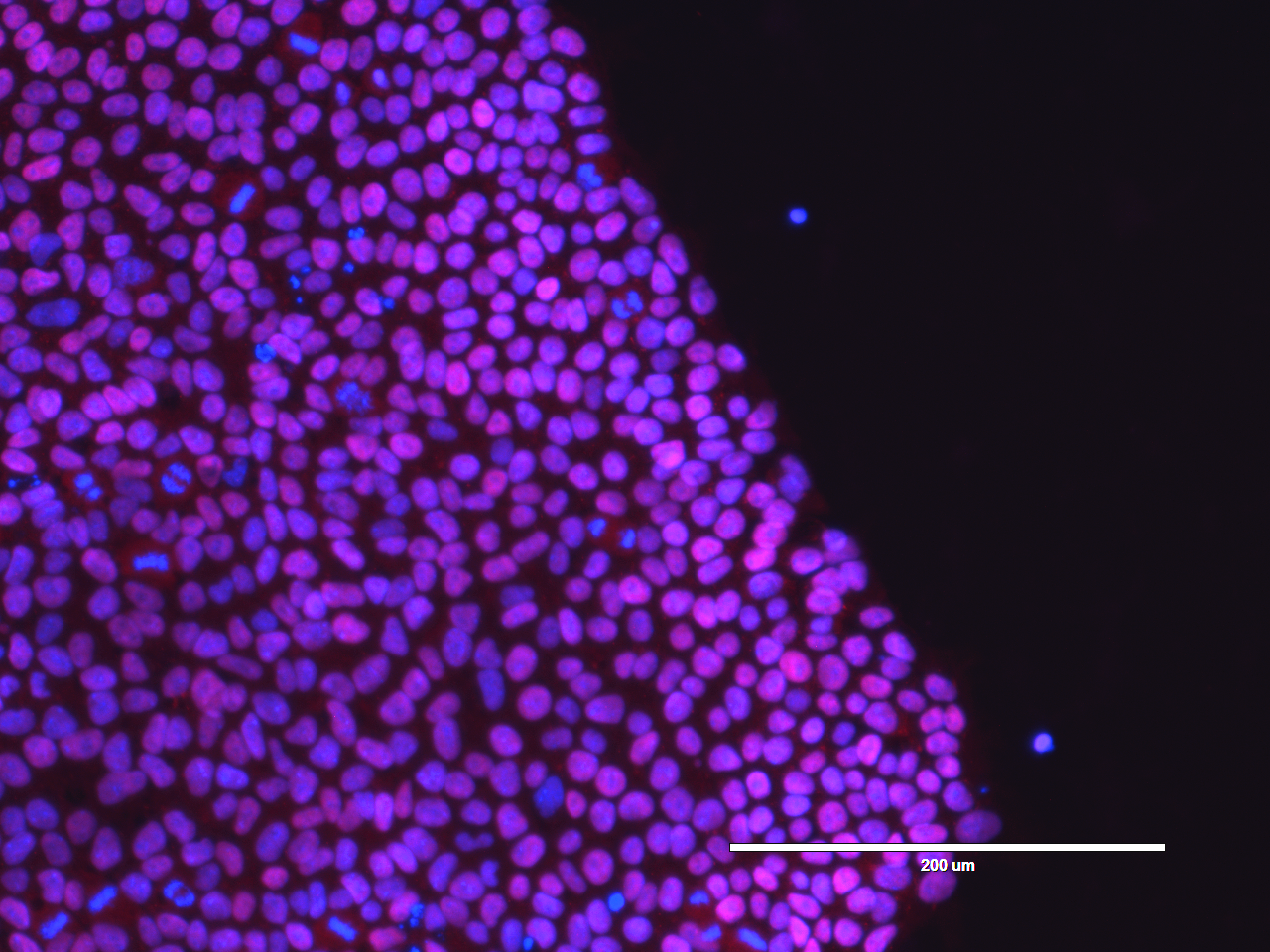

Supplement: Supplementary file 4 — Additional file 4. Uncropped gel and microscopy images. [file 13059_2023_3037_MOESM4_ESM.zip › Gel_Microscopy_images_GenomeBiology/microscopy_images/Figure S3/S3_d_Sox2_Oxr.tif]

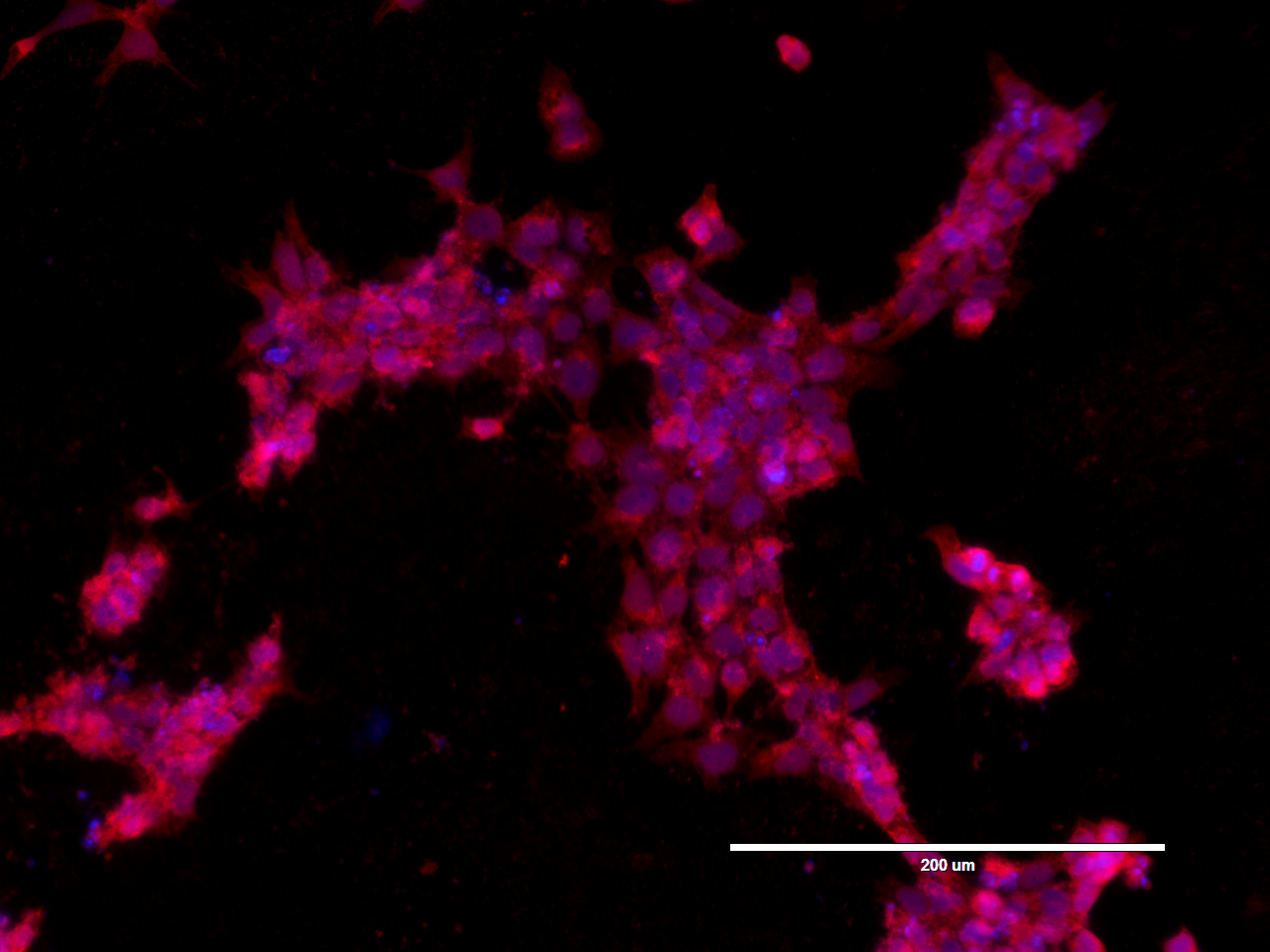

Supplement: Supplementary file 4 — Additional file 4. Uncropped gel and microscopy images. [file 13059_2023_3037_MOESM4_ESM.zip › Gel_Microscopy_images_GenomeBiology/microscopy_images/Figure S3/S3_f_Bra_Oxr.tif]

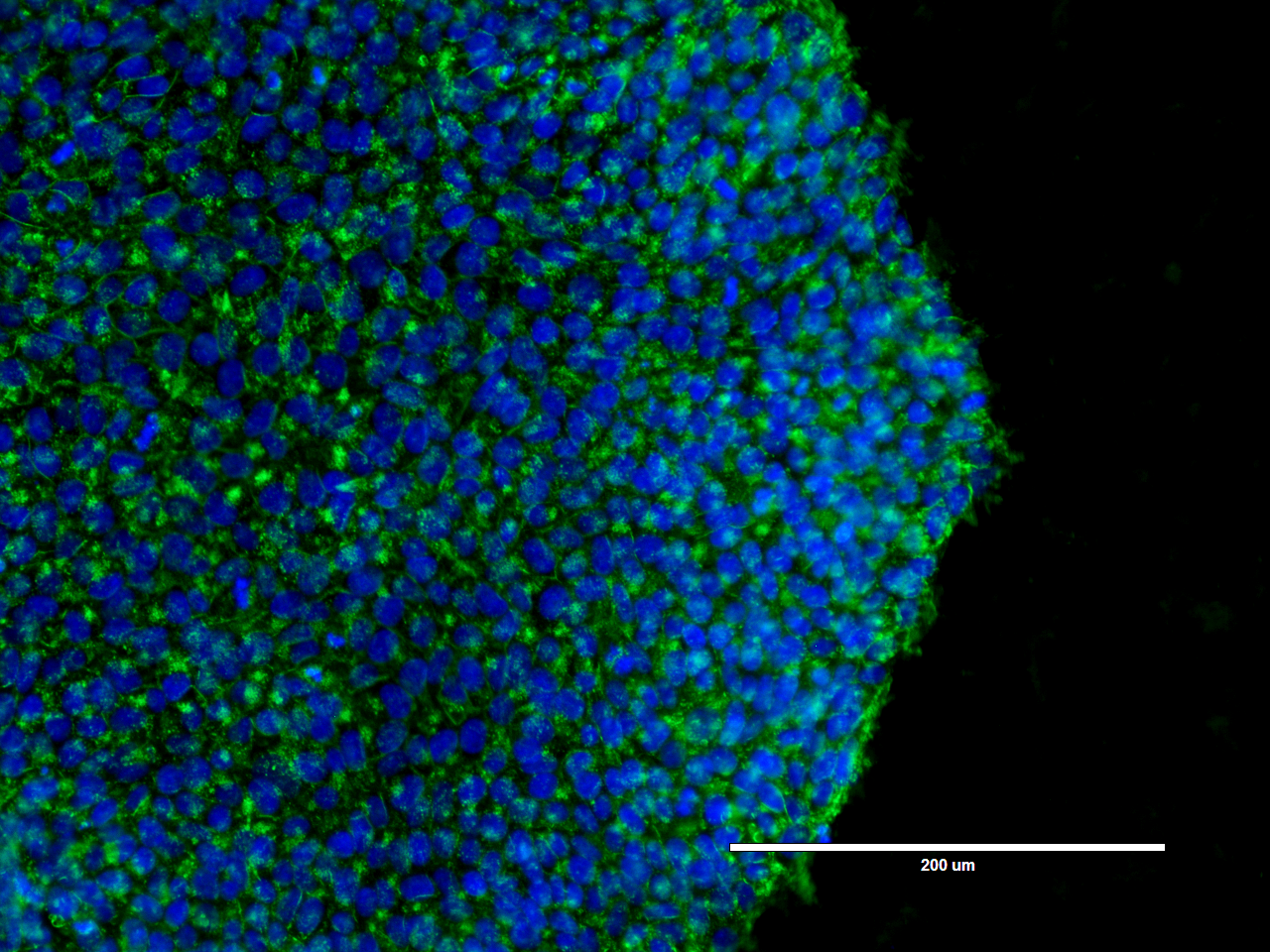

Supplement: Supplementary file 4 — Additional file 4. Uncropped gel and microscopy images. [file 13059_2023_3037_MOESM4_ESM.zip › Gel_Microscopy_images_GenomeBiology/microscopy_images/Figure S3/S3_d_SSEA4_ctrl.tif]

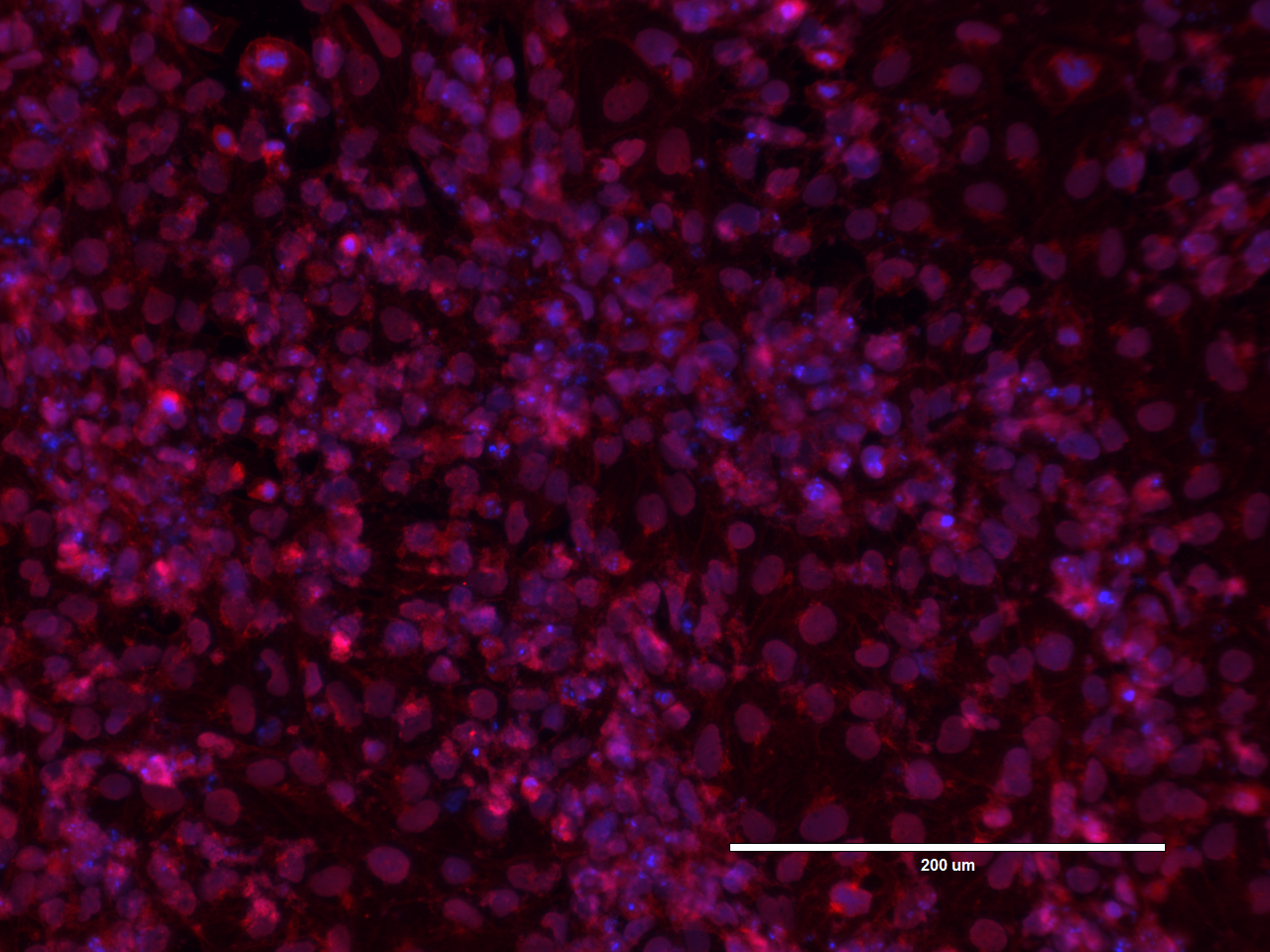

Supplement: Supplementary file 4 — Additional file 4. Uncropped gel and microscopy images. [file 13059_2023_3037_MOESM4_ESM.zip › Gel_Microscopy_images_GenomeBiology/microscopy_images/Figure S3/S3_f_Sox17_ctrl.tif]

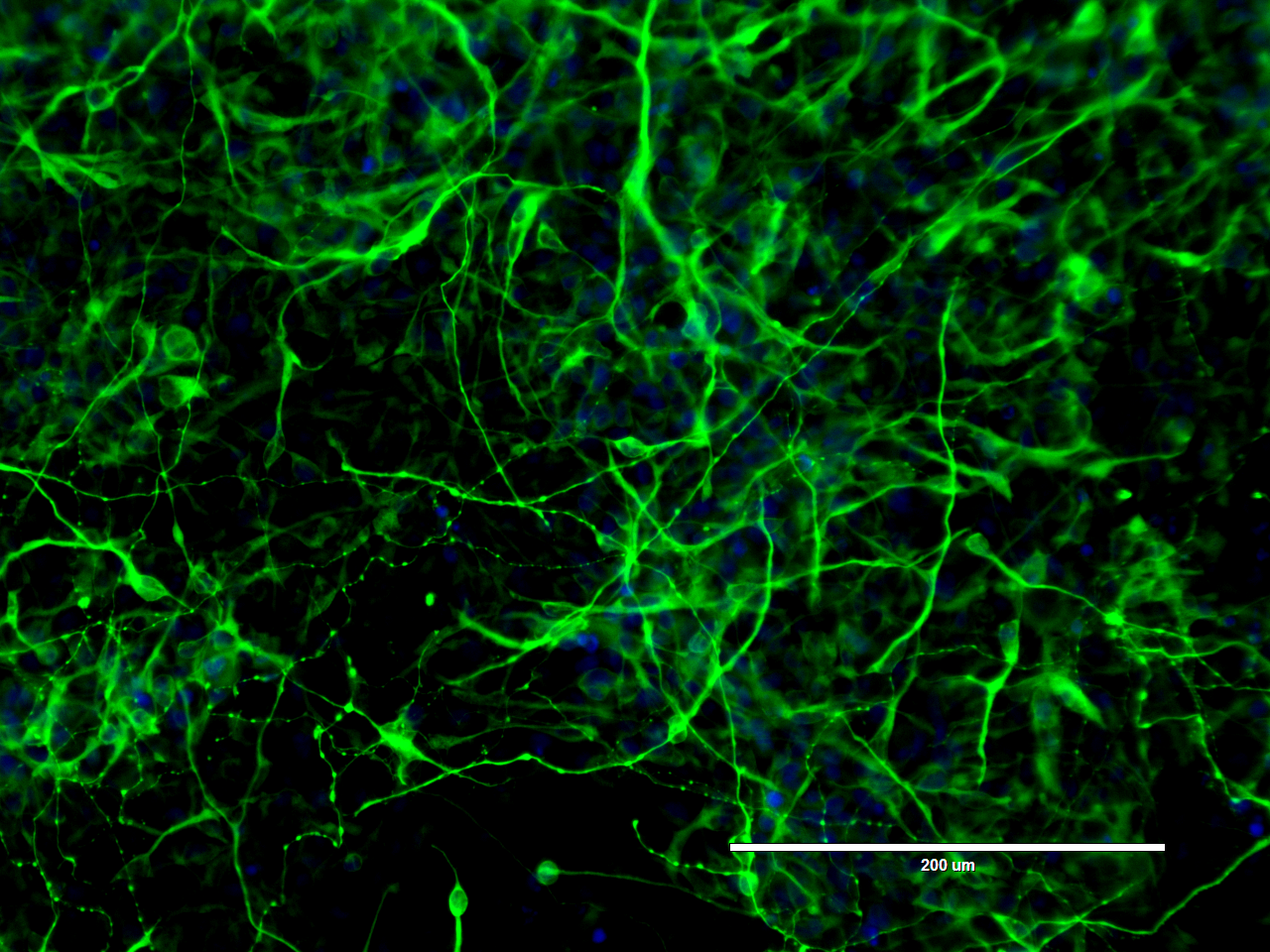

Supplement: Supplementary file 4 — Additional file 4. Uncropped gel and microscopy images. [file 13059_2023_3037_MOESM4_ESM.zip › Gel_Microscopy_images_GenomeBiology/microscopy_images/Figure S3/S3_j_Tuj1_ctrl.tif]

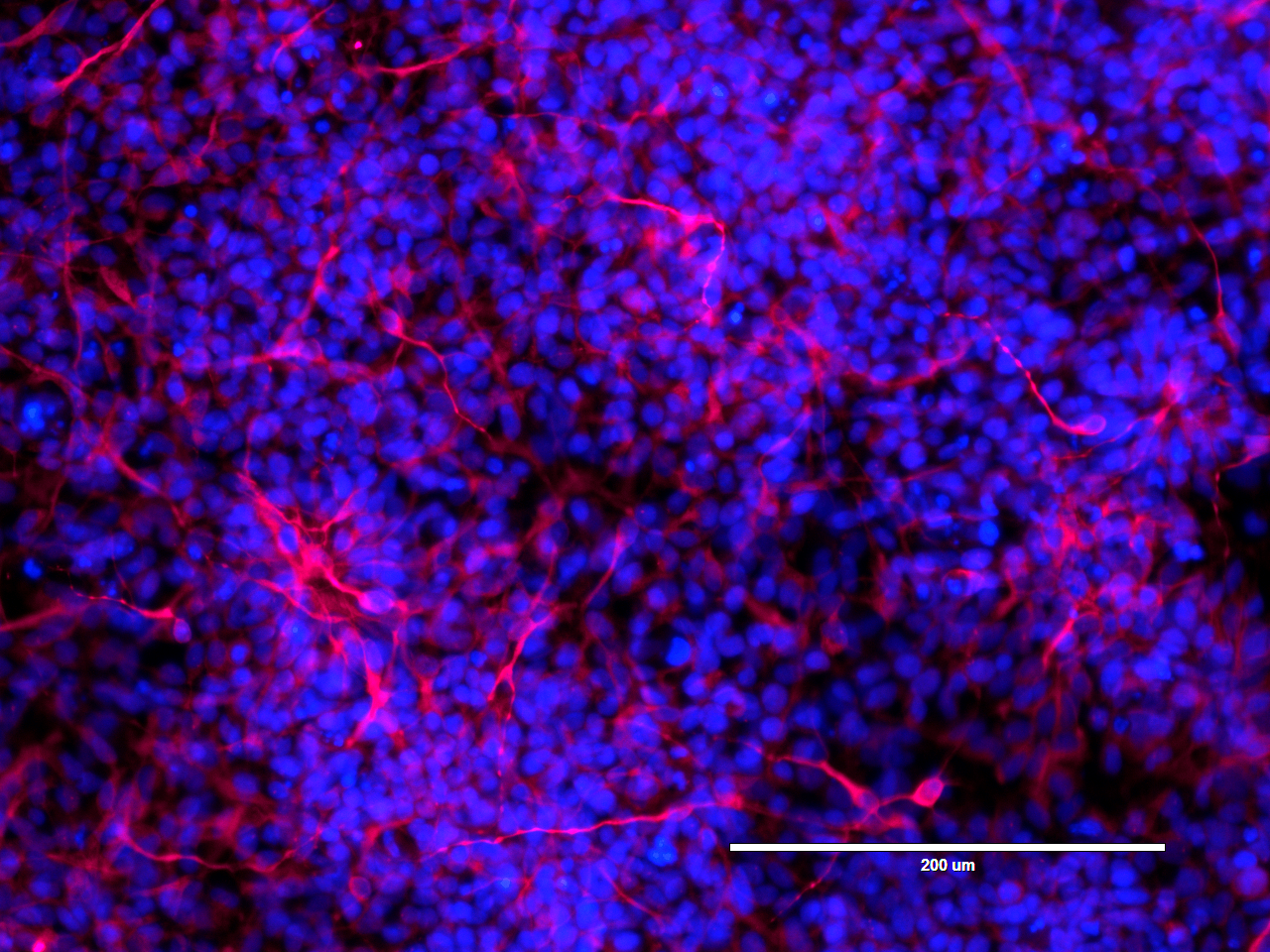

Supplement: Supplementary file 4 — Additional file 4. Uncropped gel and microscopy images. [file 13059_2023_3037_MOESM4_ESM.zip › Gel_Microscopy_images_GenomeBiology/microscopy_images/Figure S3/S3_j_Map2_ctrl.tif]

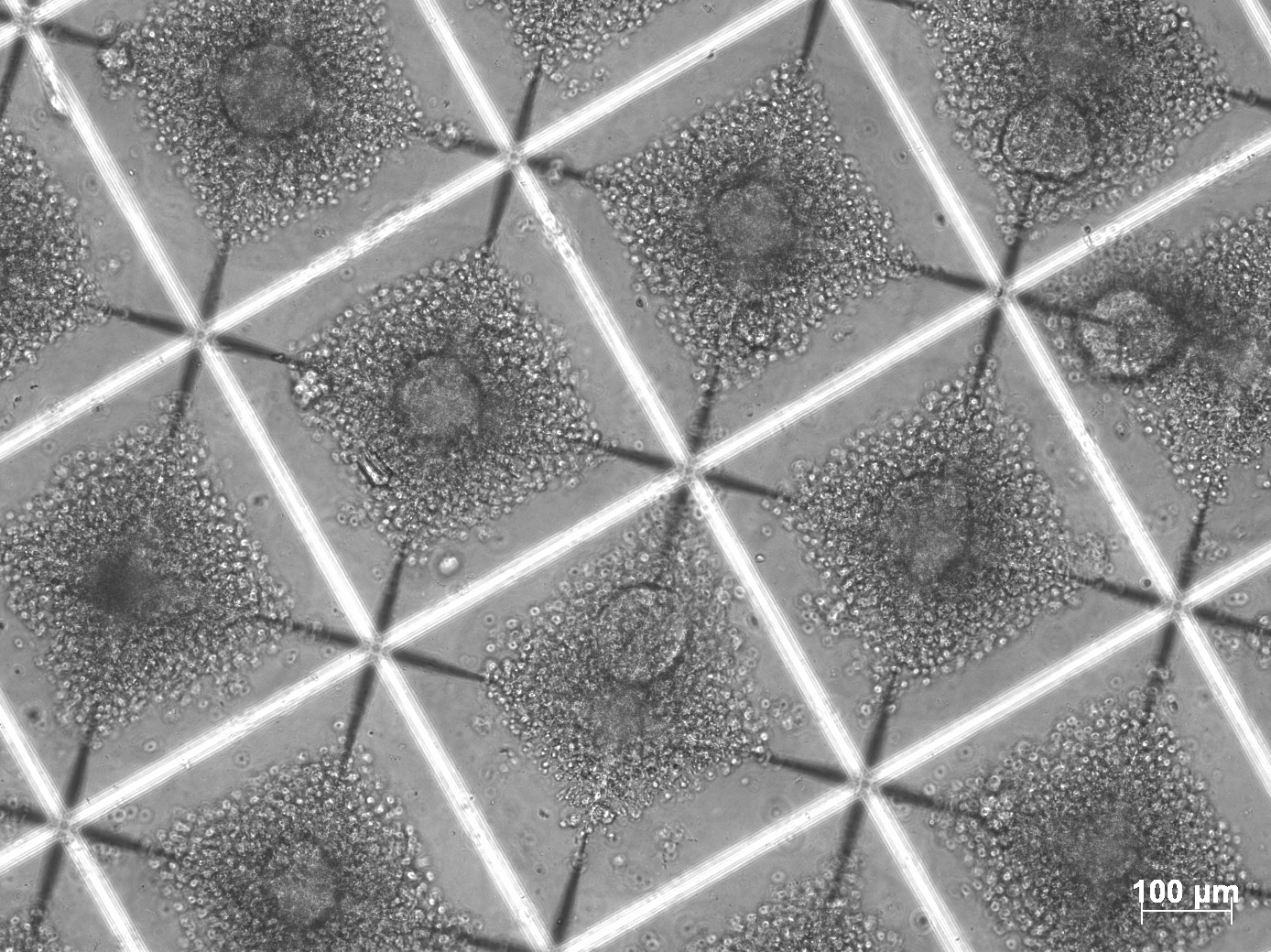

Supplement: Supplementary file 4 — Additional file 4. Uncropped gel and microscopy images. [file 13059_2023_3037_MOESM4_ESM.zip › Gel_Microscopy_images_GenomeBiology/microscopy_images/Figure S3/S3_h_EB_Oxr1.jpg]

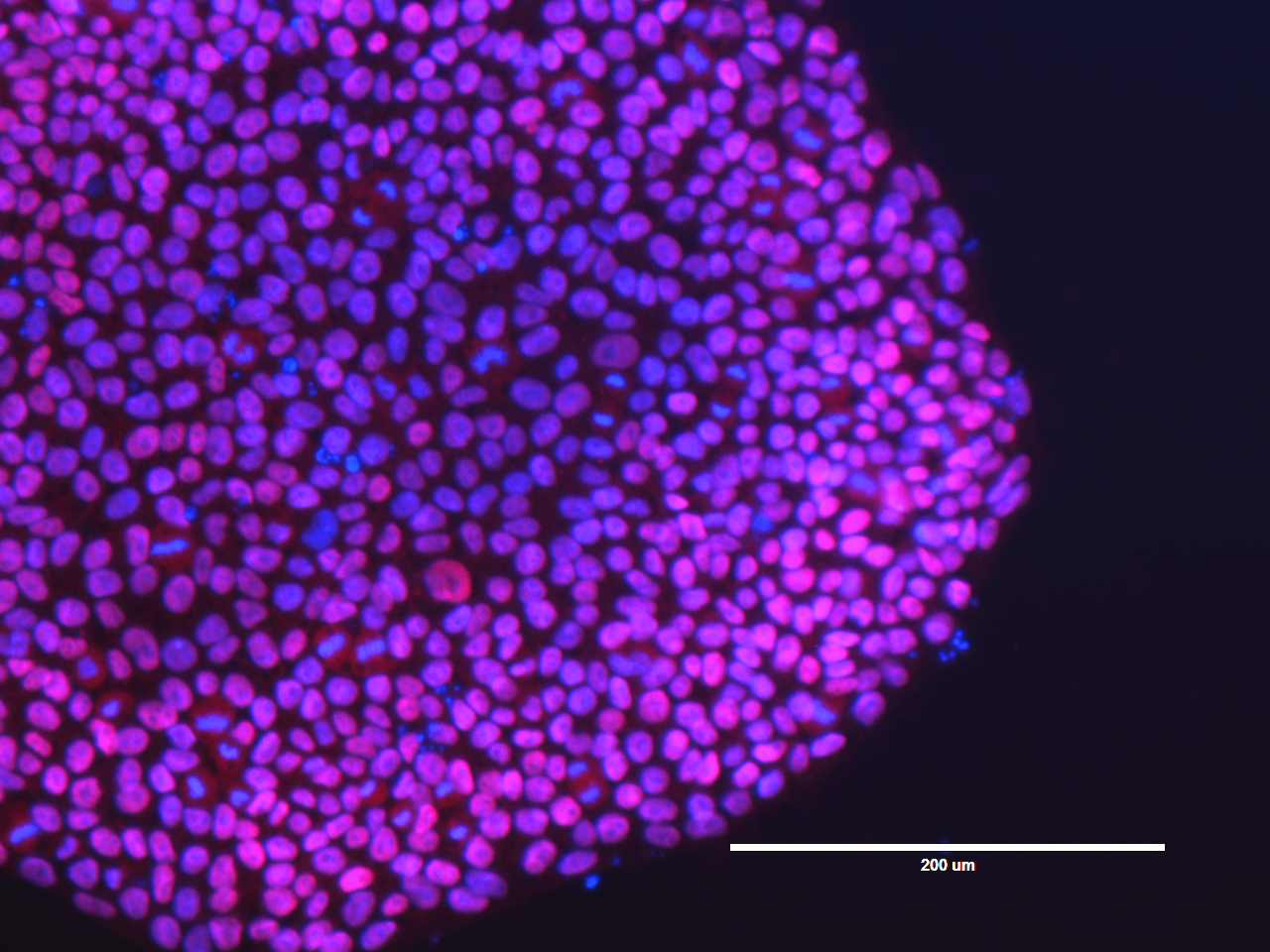

Supplement: Supplementary file 4 — Additional file 4. Uncropped gel and microscopy images. [file 13059_2023_3037_MOESM4_ESM.zip › Gel_Microscopy_images_GenomeBiology/microscopy_images/Figure S3/S3_d_NANOG_ctrl.tif]

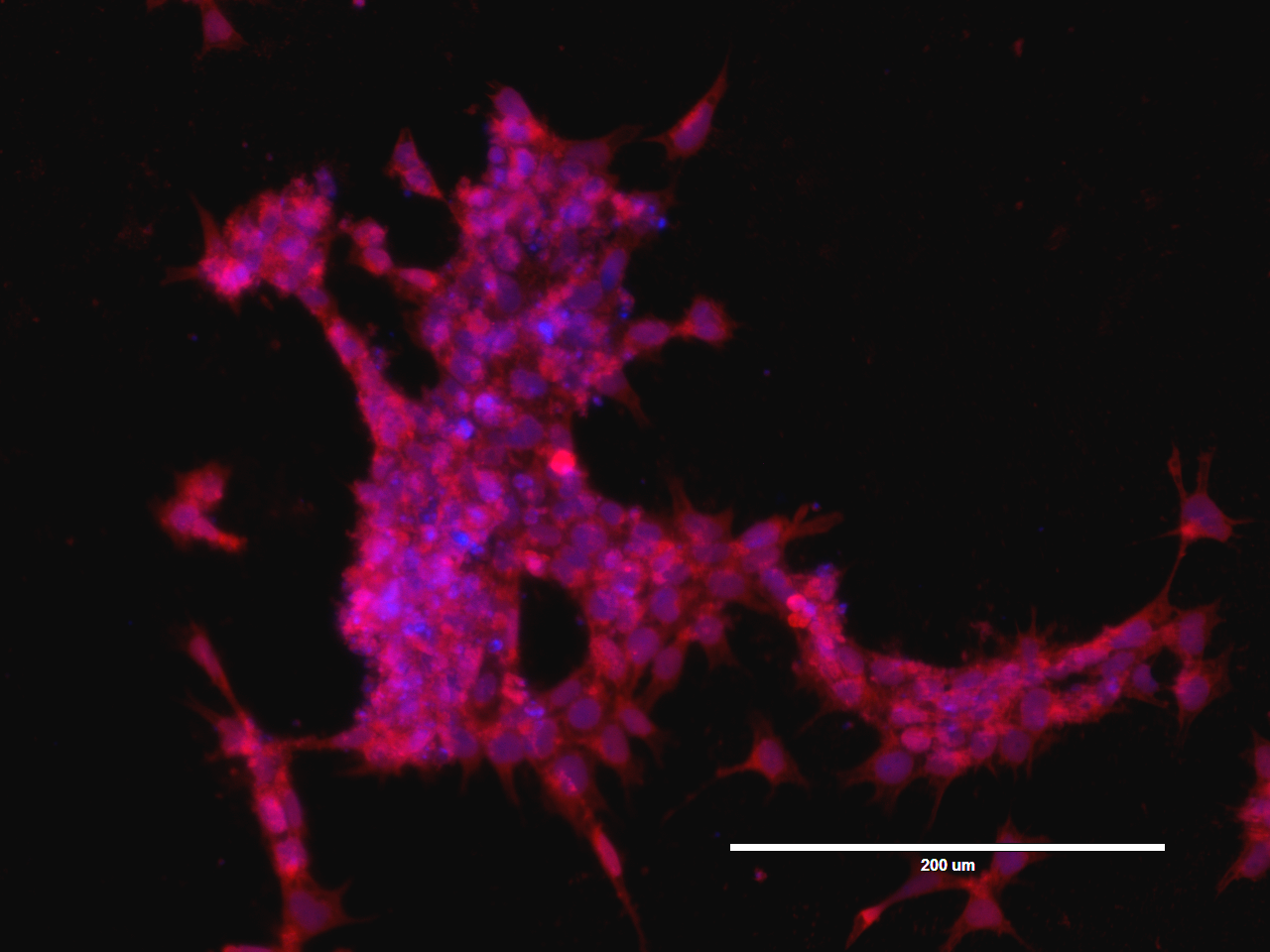

Supplement: Supplementary file 4 — Additional file 4. Uncropped gel and microscopy images. [file 13059_2023_3037_MOESM4_ESM.zip › Gel_Microscopy_images_GenomeBiology/microscopy_images/Figure S3/S3_f_Bra_ctrl.tif]

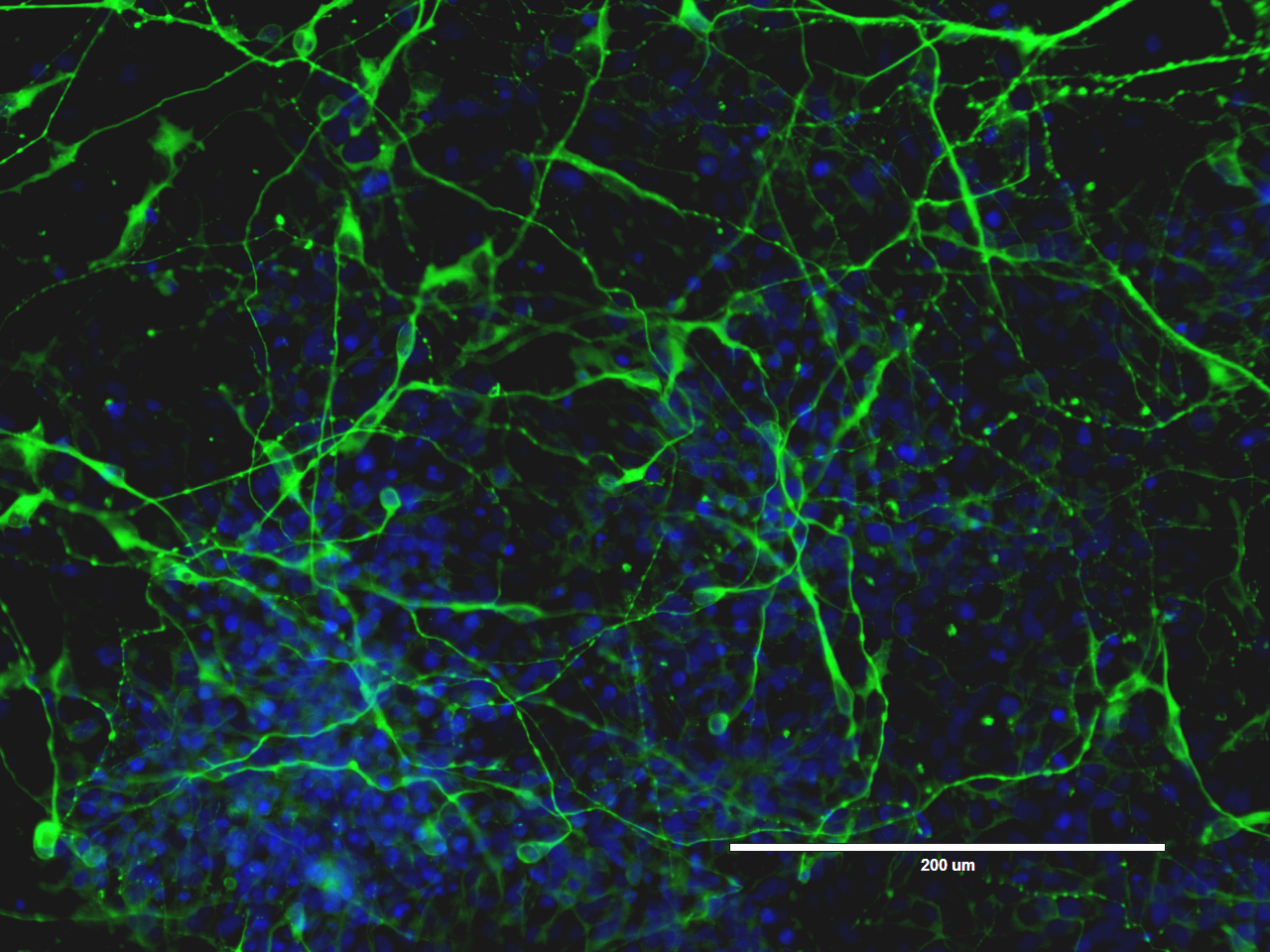

Supplement: Supplementary file 4 — Additional file 4. Uncropped gel and microscopy images. [file 13059_2023_3037_MOESM4_ESM.zip › Gel_Microscopy_images_GenomeBiology/microscopy_images/Figure S3/S3_j_Tuj1_Oxr.tif]

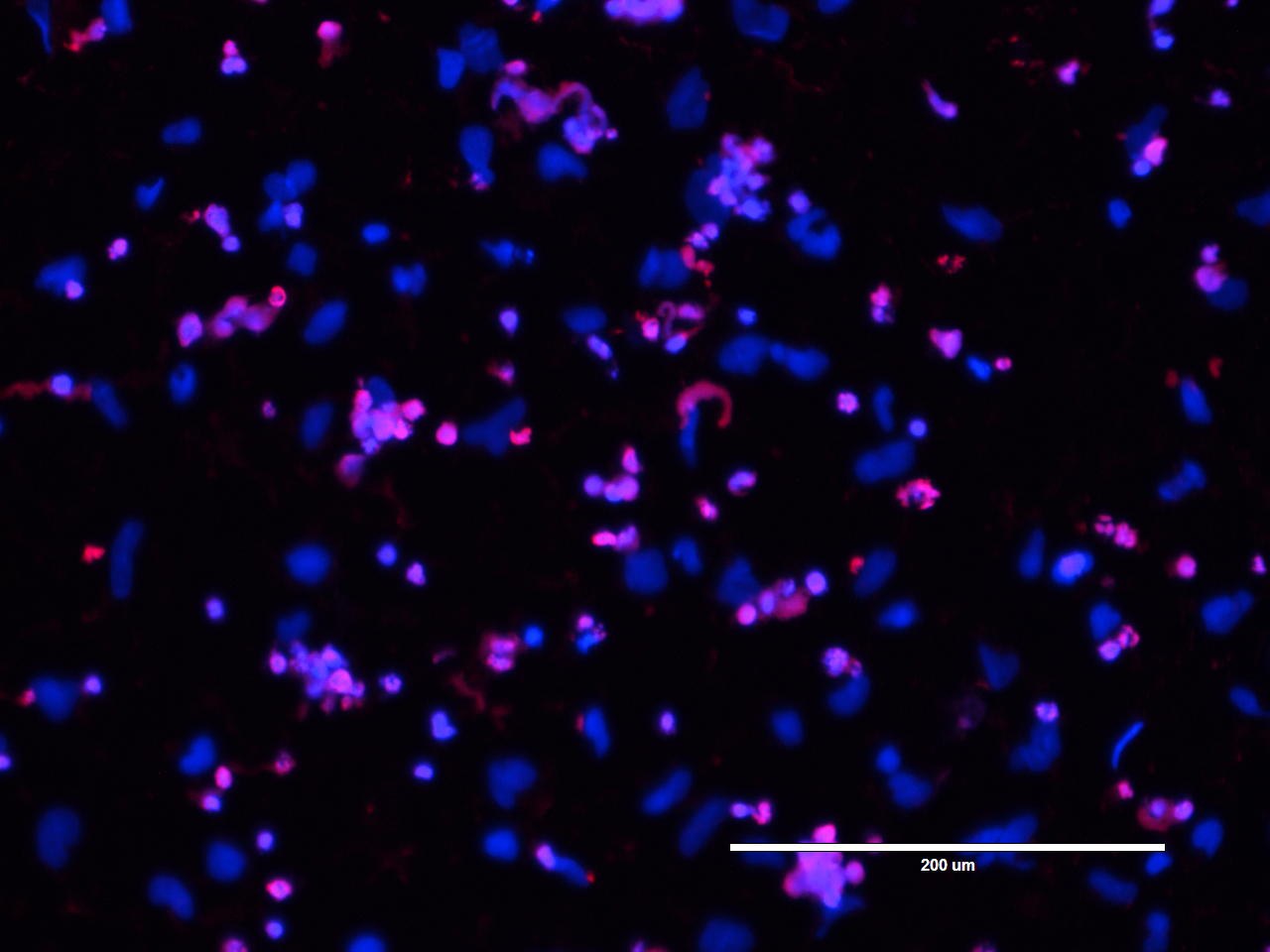

Supplement: Supplementary file 4 — Additional file 4. Uncropped gel and microscopy images. [file 13059_2023_3037_MOESM4_ESM.zip › Gel_Microscopy_images_GenomeBiology/microscopy_images/Figure S3/S3_k_Apoptosis_Oxr.tif]

## Slide 1
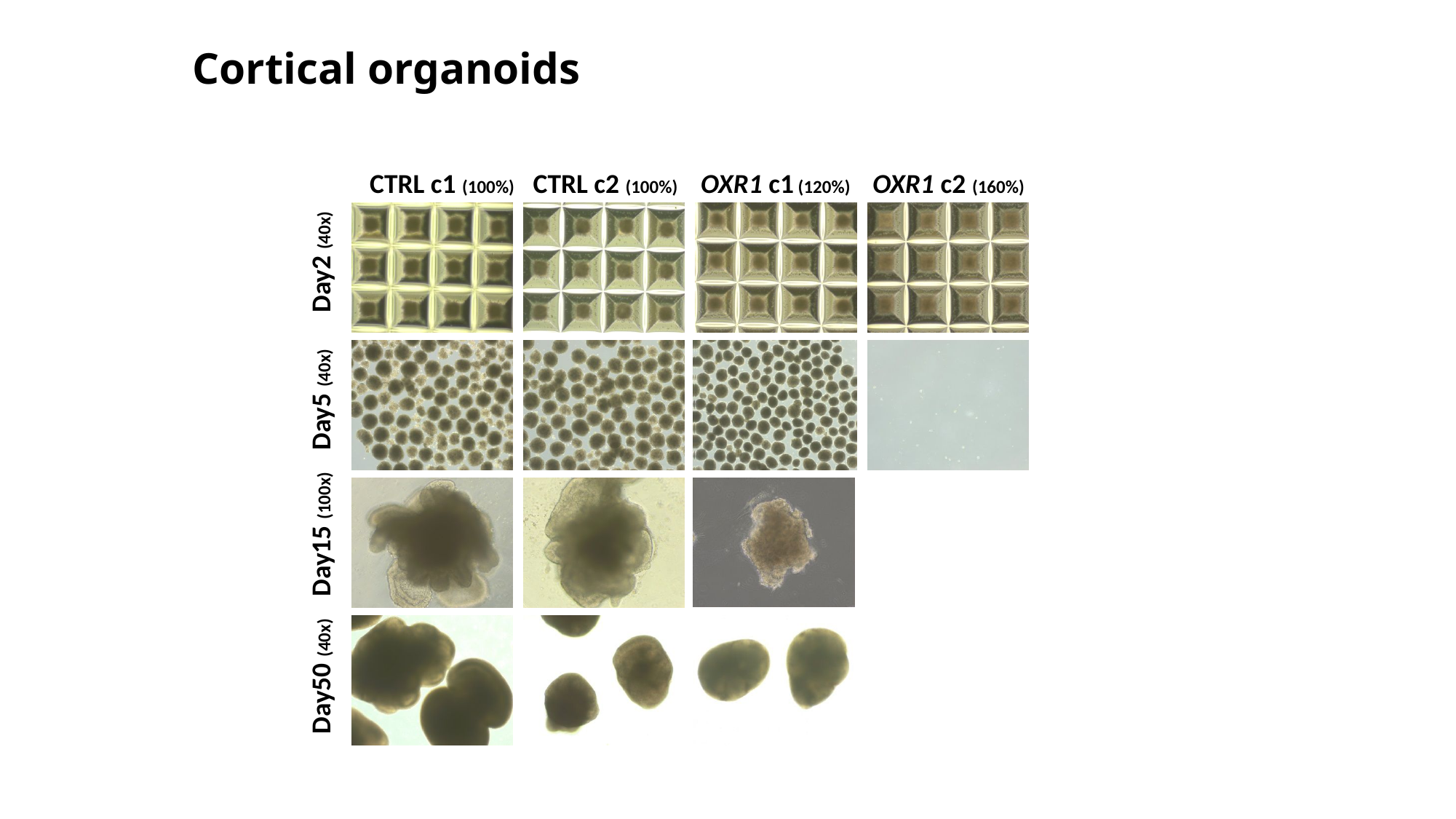

# Cortical organoids
CTRL c1 (100%)
CTRL c2 (100%)
OXR1 c1 (120%)
OXR1 c2 (160%)
Day2 (40x)
Day5 (40x)
Day15 (100x)
Day50 (40x)

Supplement: Supplementary file 4 — Additional file 4. Uncropped gel and microscopy images. [file 13059_2023_3037_MOESM4_ESM.zip › Gel_Microscopy_images_GenomeBiology/microscopy_images/Figure 6/Fig6a.pptx]

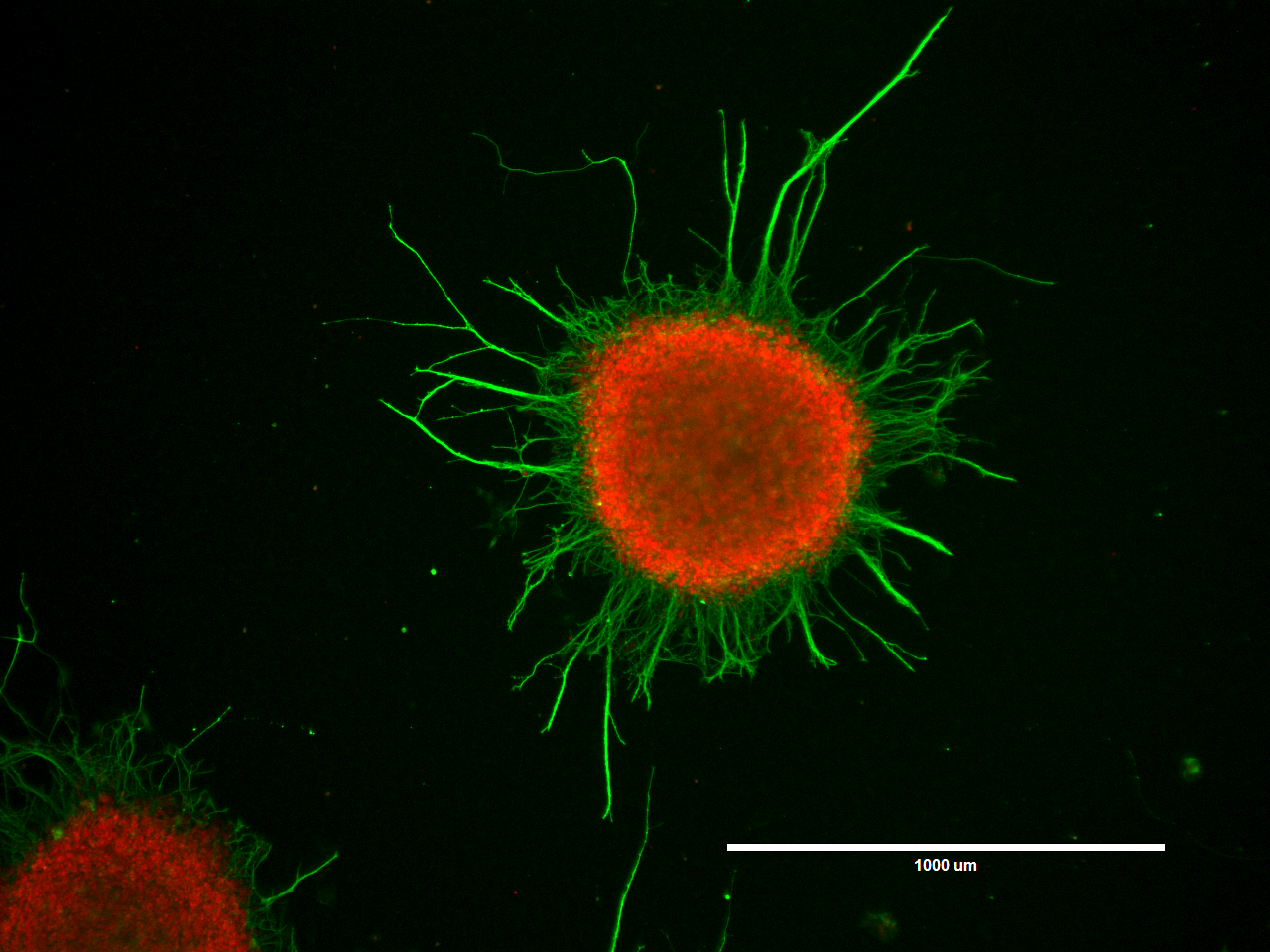

Supplement: Supplementary file 4 — Additional file 4. Uncropped gel and microscopy images. [file 13059_2023_3037_MOESM4_ESM.zip › Gel_Microscopy_images_GenomeBiology/microscopy_images/Figure 3/fig3_a_Tuj1_Oxr1.tif]

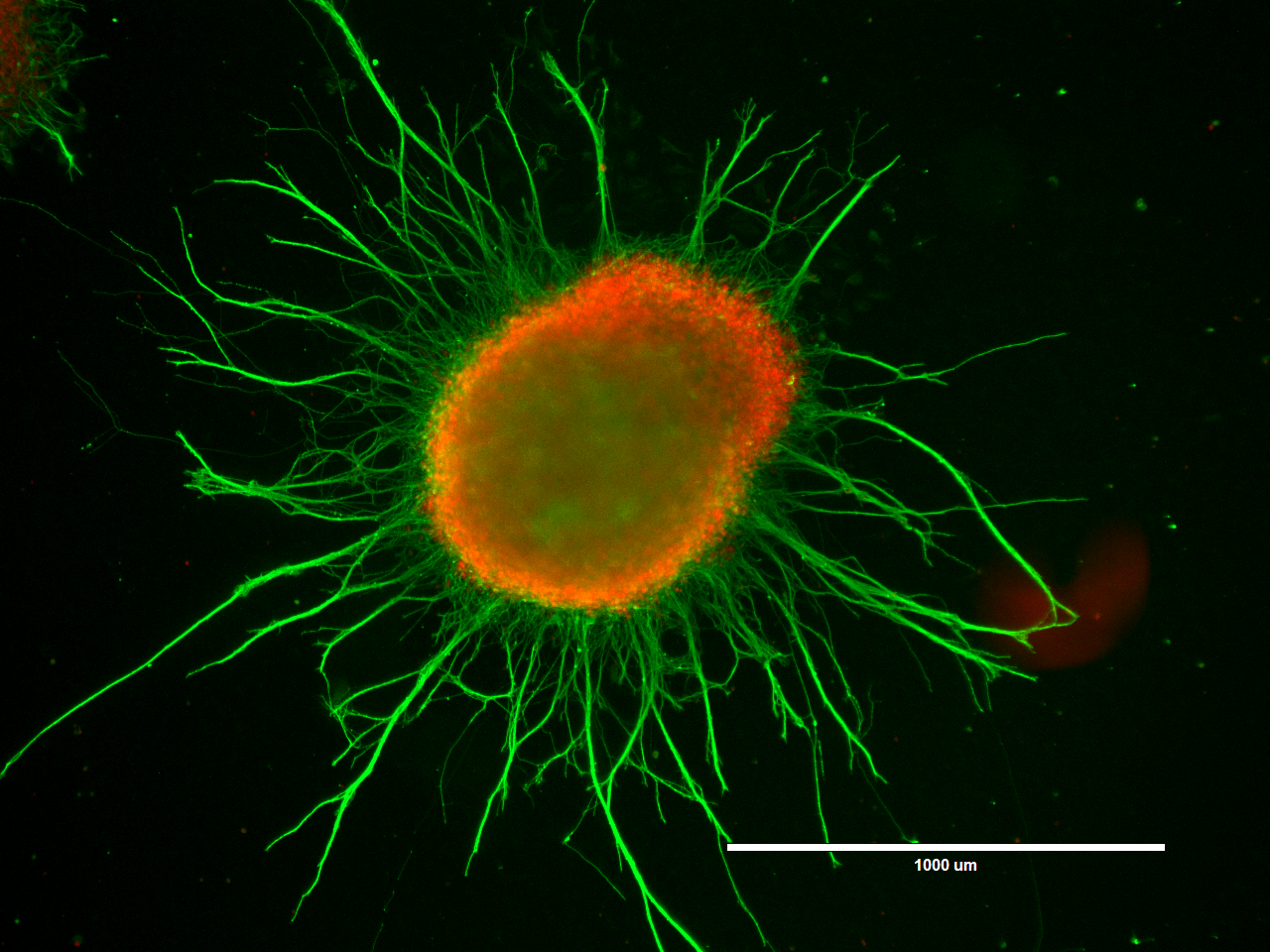

Supplement: Supplementary file 4 — Additional file 4. Uncropped gel and microscopy images. [file 13059_2023_3037_MOESM4_ESM.zip › Gel_Microscopy_images_GenomeBiology/microscopy_images/Figure 3/fig3_a_Tuj1_ctrl.tif]

## Slide 1
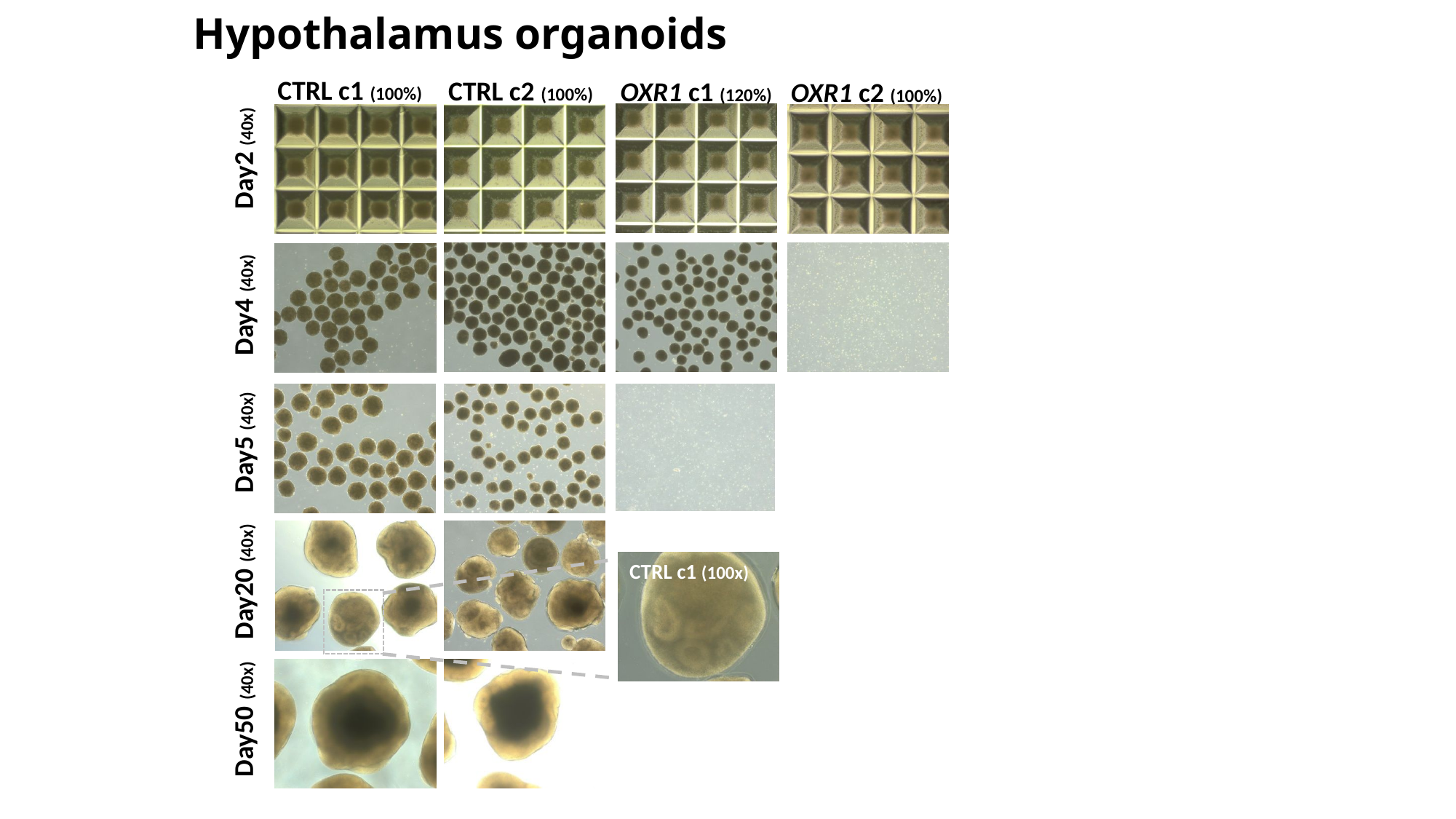

# Hypothalamus organoids
CTRL c1 (100%)
CTRL c2 (100%)
OXR1 c1 (120%)
OXR1 c2 (100%)
Day2 (40x)
Day4 (40x)
Day5 (40x)
Day20 (40x)
CTRL c1 (100x)
Day50 (40x)

Supplement: Supplementary file 4 — Additional file 4. Uncropped gel and microscopy images. [file 13059_2023_3037_MOESM4_ESM.zip › Gel_Microscopy_images_GenomeBiology/microscopy_images/Figure 5/Fig5e.pptx]

## Slide 1
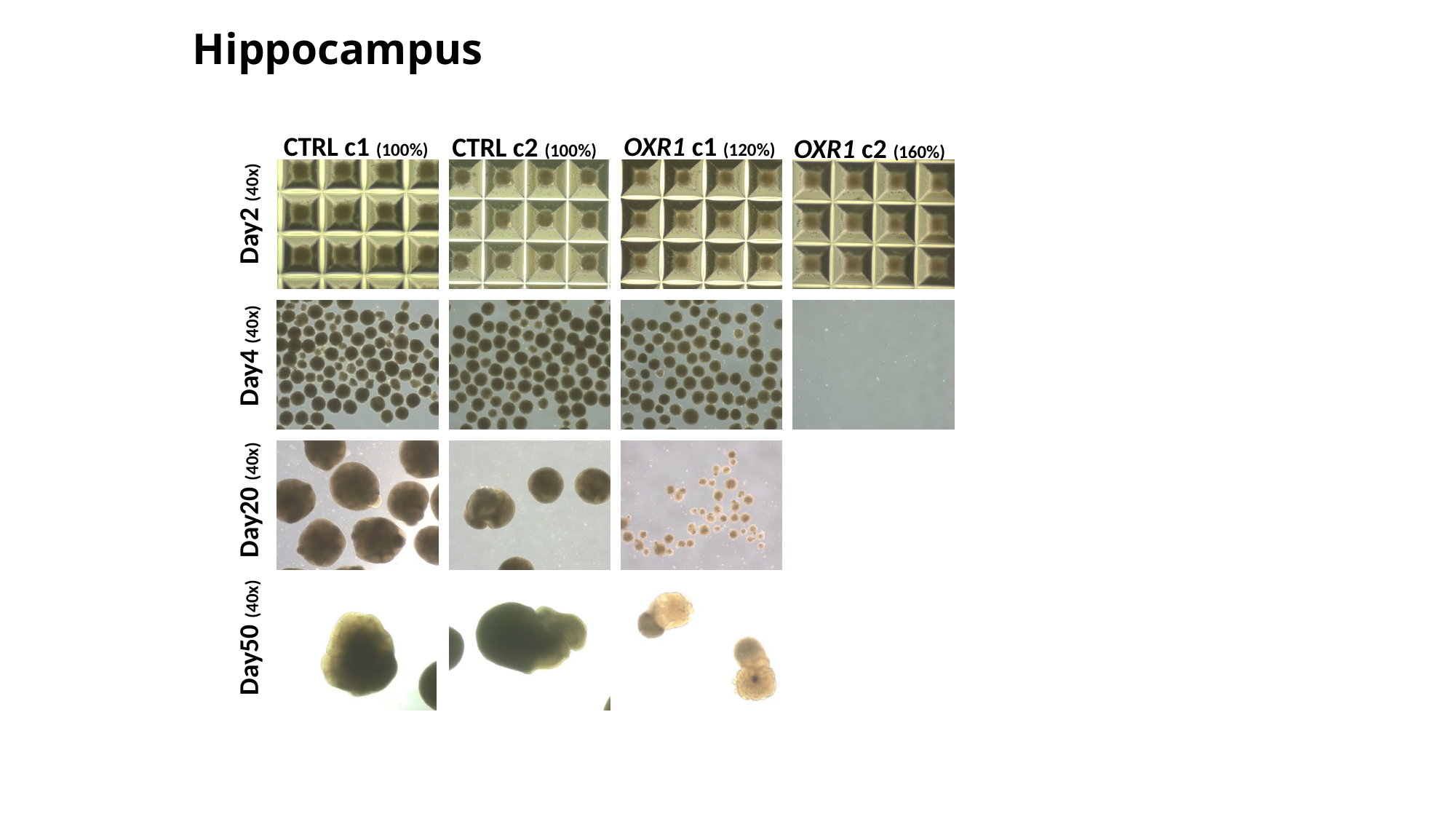

Hippocampus
CTRL c1 (100%)
OXR1 c1 (120%)
CTRL c2 (100%)
OXR1 c2 (160%)
Day2 (40x)
Day4 (40x)
Day20 (40x)
Day50 (40x)

Supplement: Supplementary file 4 — Additional file 4. Uncropped gel and microscopy images. [file 13059_2023_3037_MOESM4_ESM.zip › Gel_Microscopy_images_GenomeBiology/microscopy_images/Figure 5/Fig5f.pptx]

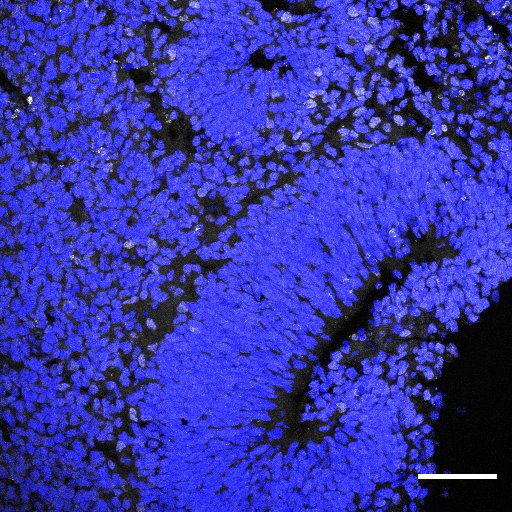

Supplement: Supplementary file 4 — Additional file 4. Uncropped gel and microscopy images. [file 13059_2023_3037_MOESM4_ESM.zip › Gel_Microscopy_images_GenomeBiology/microscopy_images/Figure 7/7d/fig7d_en2_day20_oxr1_c1-1.jpg]

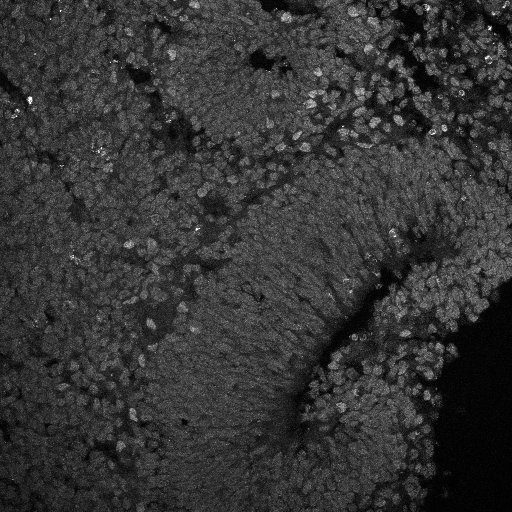

Supplement: Supplementary file 4 — Additional file 4. Uncropped gel and microscopy images. [file 13059_2023_3037_MOESM4_ESM.zip › Gel_Microscopy_images_GenomeBiology/microscopy_images/Figure 7/7d/fig7d_en2_day20_oxr1_c1-2.jpg]

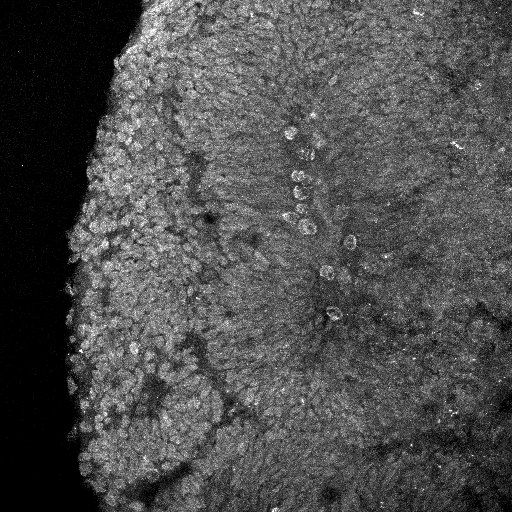

Supplement: Supplementary file 4 — Additional file 4. Uncropped gel and microscopy images. [file 13059_2023_3037_MOESM4_ESM.zip › Gel_Microscopy_images_GenomeBiology/microscopy_images/Figure 7/7d/fig7d_en2_day20_ctrl_c1-2.jpg]

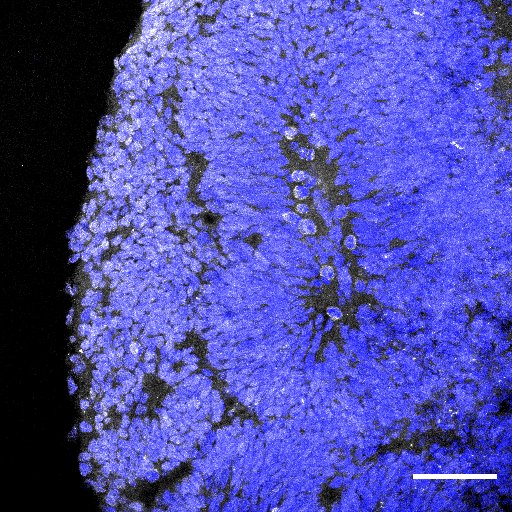

Supplement: Supplementary file 4 — Additional file 4. Uncropped gel and microscopy images. [file 13059_2023_3037_MOESM4_ESM.zip › Gel_Microscopy_images_GenomeBiology/microscopy_images/Figure 7/7d/fig7d_en2_day20_ctrl_c1-1.jpg]

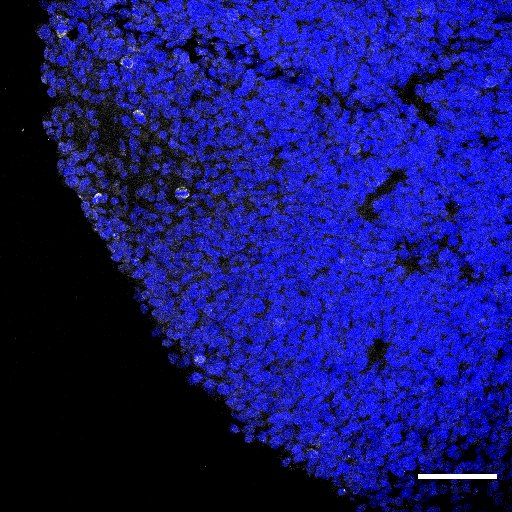

Supplement: Supplementary file 4 — Additional file 4. Uncropped gel and microscopy images. [file 13059_2023_3037_MOESM4_ESM.zip › Gel_Microscopy_images_GenomeBiology/microscopy_images/Figure 7/7d/fig7d_en2_day20_oxr1_c2-1.jpg]

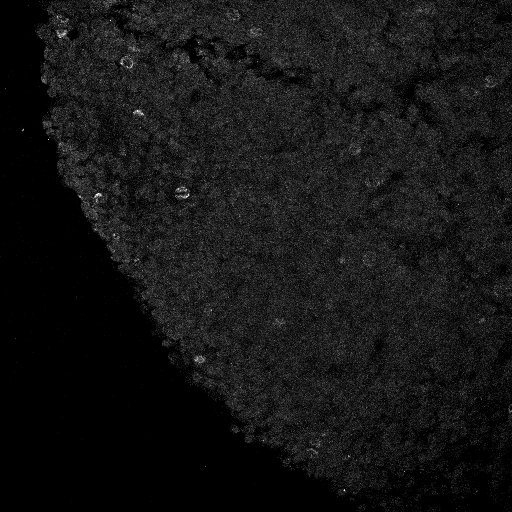

Supplement: Supplementary file 4 — Additional file 4. Uncropped gel and microscopy images. [file 13059_2023_3037_MOESM4_ESM.zip › Gel_Microscopy_images_GenomeBiology/microscopy_images/Figure 7/7d/fig7d_en2_day20_oxr1_c2-2.jpg]

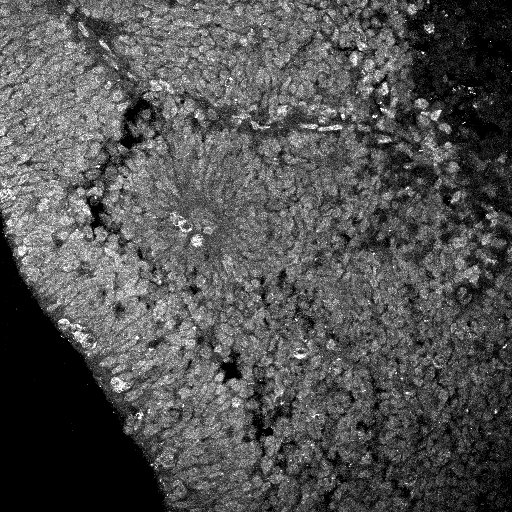

Supplement: Supplementary file 4 — Additional file 4. Uncropped gel and microscopy images. [file 13059_2023_3037_MOESM4_ESM.zip › Gel_Microscopy_images_GenomeBiology/microscopy_images/Figure 7/7d/fig7d_en2_day20_ctrl_c2-2.jpg]

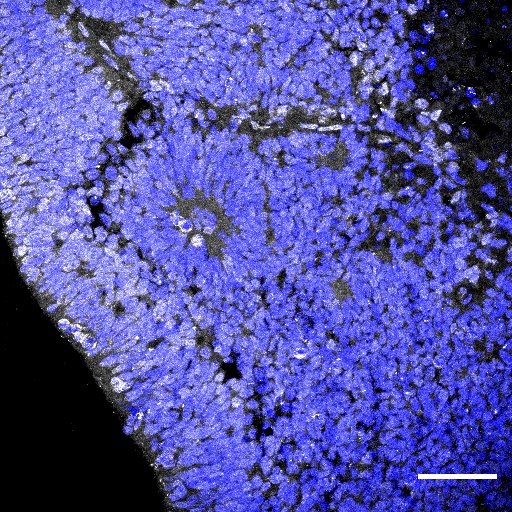

Supplement: Supplementary file 4 — Additional file 4. Uncropped gel and microscopy images. [file 13059_2023_3037_MOESM4_ESM.zip › Gel_Microscopy_images_GenomeBiology/microscopy_images/Figure 7/7d/fig7d_en2_day20_ctrl_c2-1.jpg]

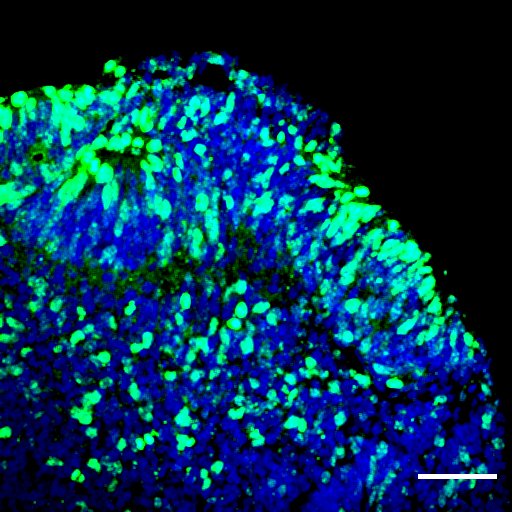

Supplement: Supplementary file 4 — Additional file 4. Uncropped gel and microscopy images. [file 13059_2023_3037_MOESM4_ESM.zip › Gel_Microscopy_images_GenomeBiology/microscopy_images/Figure 7/7c/fig7c_Ki67_day20_ctrl_c2.jpg]

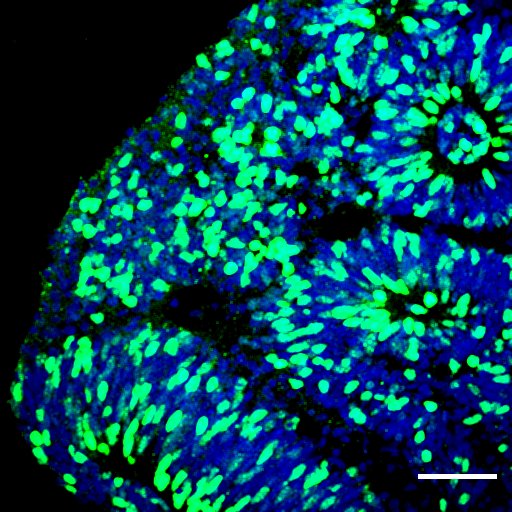

Supplement: Supplementary file 4 — Additional file 4. Uncropped gel and microscopy images. [file 13059_2023_3037_MOESM4_ESM.zip › Gel_Microscopy_images_GenomeBiology/microscopy_images/Figure 7/7c/fig7c_Ki67_day20_ctrl_c1.jpg]

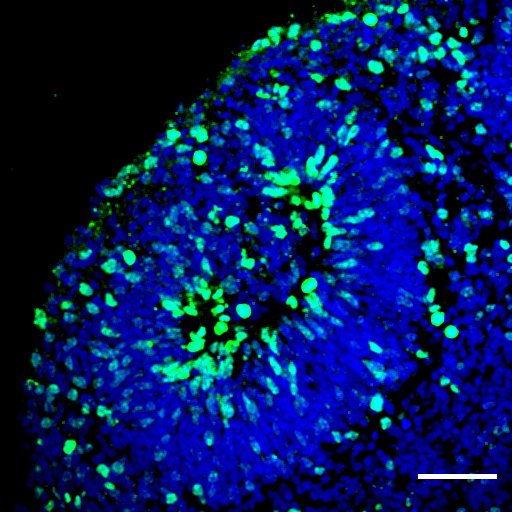

Supplement: Supplementary file 4 — Additional file 4. Uncropped gel and microscopy images. [file 13059_2023_3037_MOESM4_ESM.zip › Gel_Microscopy_images_GenomeBiology/microscopy_images/Figure 7/7c/fig7c_Ki67_day20_oxr1_c1.jpg]

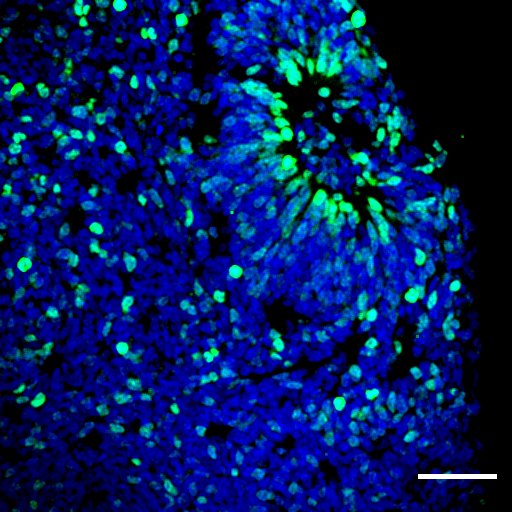

Supplement: Supplementary file 4 — Additional file 4. Uncropped gel and microscopy images. [file 13059_2023_3037_MOESM4_ESM.zip › Gel_Microscopy_images_GenomeBiology/microscopy_images/Figure 7/7c/fig7c_Ki67_day20_oxr1_c2.jpg]

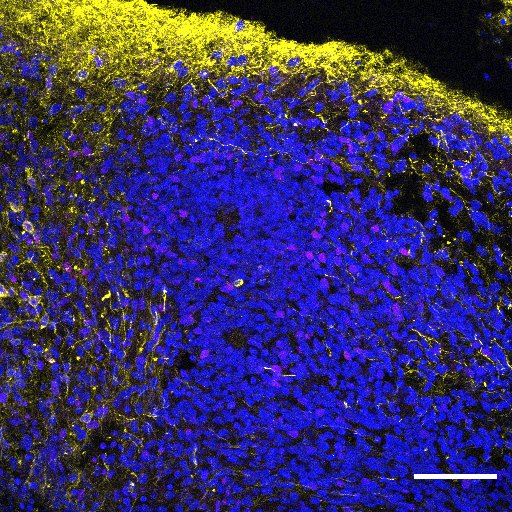

Supplement: Supplementary file 4 — Additional file 4. Uncropped gel and microscopy images. [file 13059_2023_3037_MOESM4_ESM.zip › Gel_Microscopy_images_GenomeBiology/microscopy_images/Figure 7/7b/fig7b_FOXA2_TH_day50_oxr1_c1.jpg]

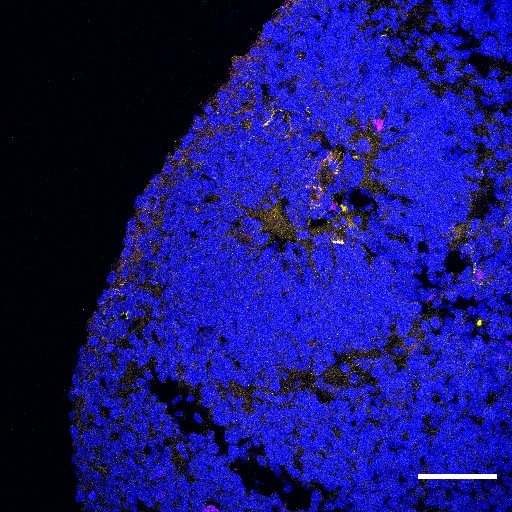

Supplement: Supplementary file 4 — Additional file 4. Uncropped gel and microscopy images. [file 13059_2023_3037_MOESM4_ESM.zip › Gel_Microscopy_images_GenomeBiology/microscopy_images/Figure 7/7b/fig7b_FOXA2_TH_day20_oxr1_c1.jpg]

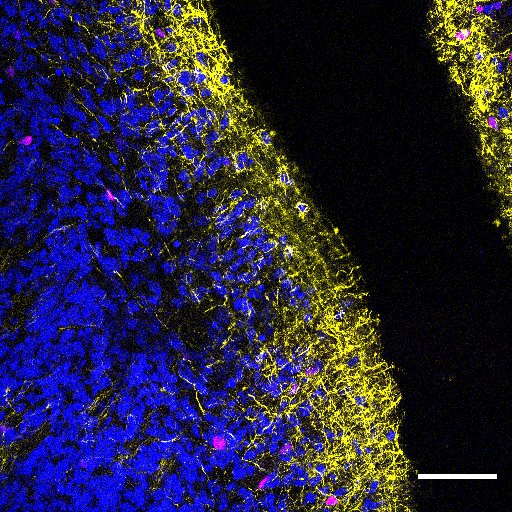

Supplement: Supplementary file 4 — Additional file 4. Uncropped gel and microscopy images. [file 13059_2023_3037_MOESM4_ESM.zip › Gel_Microscopy_images_GenomeBiology/microscopy_images/Figure 7/7b/fig7b_FOXA2_TH_day50_oxr1_c2.jpg]

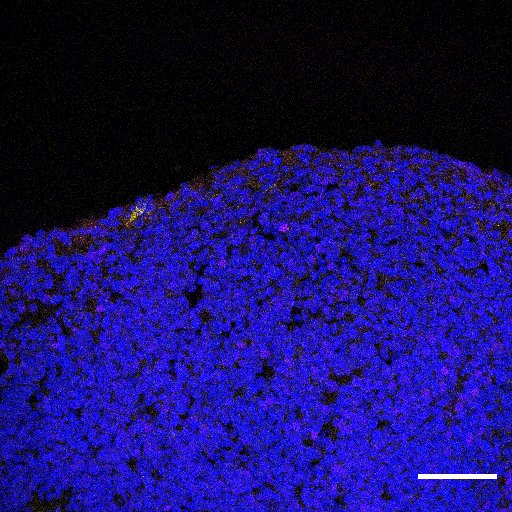

Supplement: Supplementary file 4 — Additional file 4. Uncropped gel and microscopy images. [file 13059_2023_3037_MOESM4_ESM.zip › Gel_Microscopy_images_GenomeBiology/microscopy_images/Figure 7/7b/fig7b_FOXA2_TH_day20_oxr1_c2.jpg]

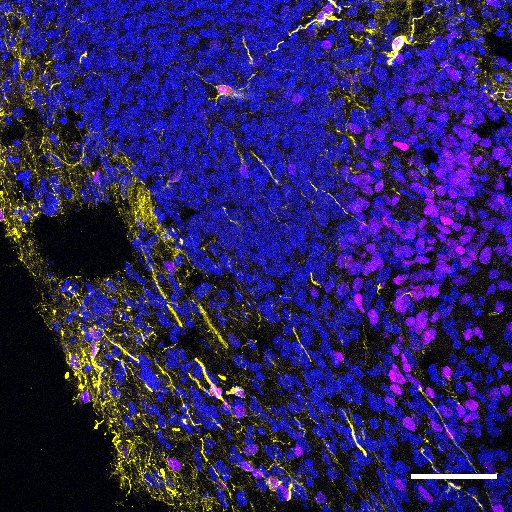

Supplement: Supplementary file 4 — Additional file 4. Uncropped gel and microscopy images. [file 13059_2023_3037_MOESM4_ESM.zip › Gel_Microscopy_images_GenomeBiology/microscopy_images/Figure 7/7b/fig7b_FOXA2_TH_day50_ctrl_c2.jpg]

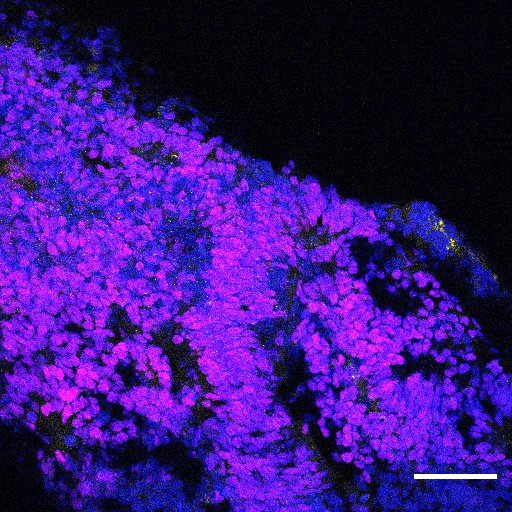

Supplement: Supplementary file 4 — Additional file 4. Uncropped gel and microscopy images. [file 13059_2023_3037_MOESM4_ESM.zip › Gel_Microscopy_images_GenomeBiology/microscopy_images/Figure 7/7b/fig7b_FOXA2_TH_day20_ctrl_c2.jpg]

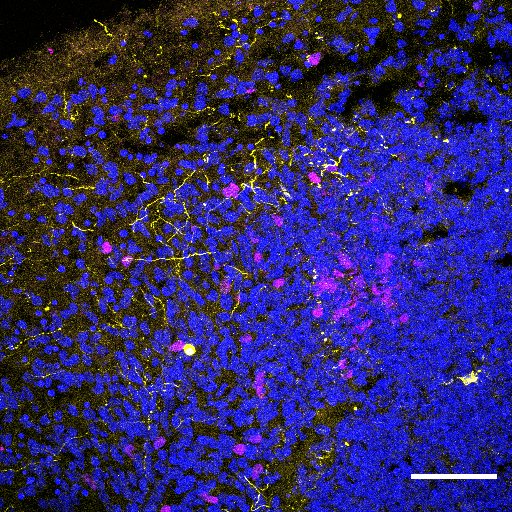

Supplement: Supplementary file 4 — Additional file 4. Uncropped gel and microscopy images. [file 13059_2023_3037_MOESM4_ESM.zip › Gel_Microscopy_images_GenomeBiology/microscopy_images/Figure 7/7b/fig7b_FOXA2_TH_day50_ctrl_c1.jpg]

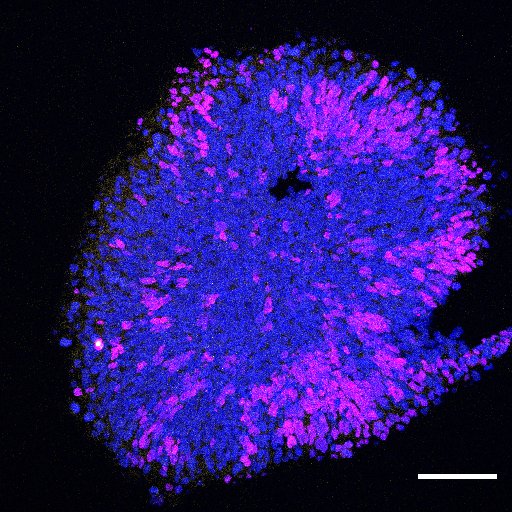

Supplement: Supplementary file 4 — Additional file 4. Uncropped gel and microscopy images. [file 13059_2023_3037_MOESM4_ESM.zip › Gel_Microscopy_images_GenomeBiology/microscopy_images/Figure 7/7b/fig7b_FOXA2_TH_day20_ctrl_c1.jpg]

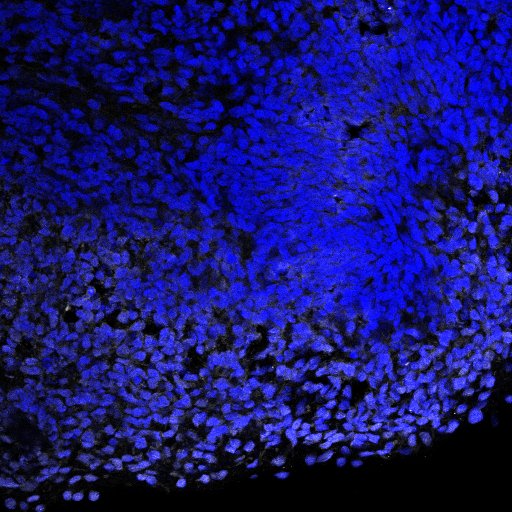

Supplement: Supplementary file 4 — Additional file 4. Uncropped gel and microscopy images. [file 13059_2023_3037_MOESM4_ESM.zip › Gel_Microscopy_images_GenomeBiology/microscopy_images/Figure 7/7e/fig7e_H4R3me2s_day50_oxr1_c1-1.jpg]

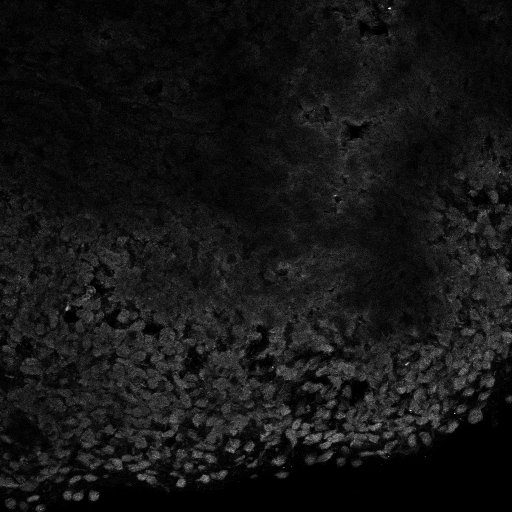

Supplement: Supplementary file 4 — Additional file 4. Uncropped gel and microscopy images. [file 13059_2023_3037_MOESM4_ESM.zip › Gel_Microscopy_images_GenomeBiology/microscopy_images/Figure 7/7e/fig7e_H4R3me2s_day50_oxr1_c1-2.jpg]

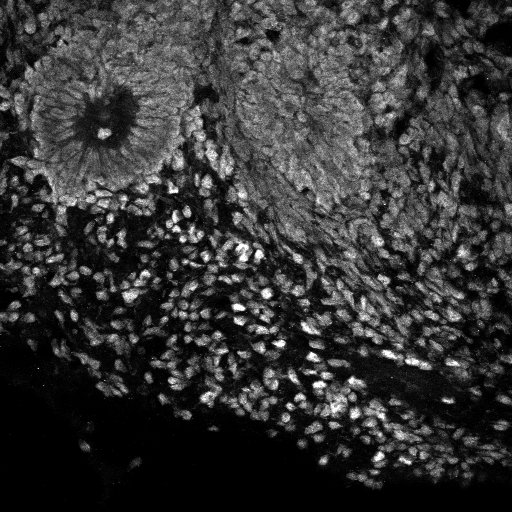

Supplement: Supplementary file 4 — Additional file 4. Uncropped gel and microscopy images. [file 13059_2023_3037_MOESM4_ESM.zip › Gel_Microscopy_images_GenomeBiology/microscopy_images/Figure 7/7e/fig7e_H4R3me2s_day50_ctrl_c1-2.jpg]

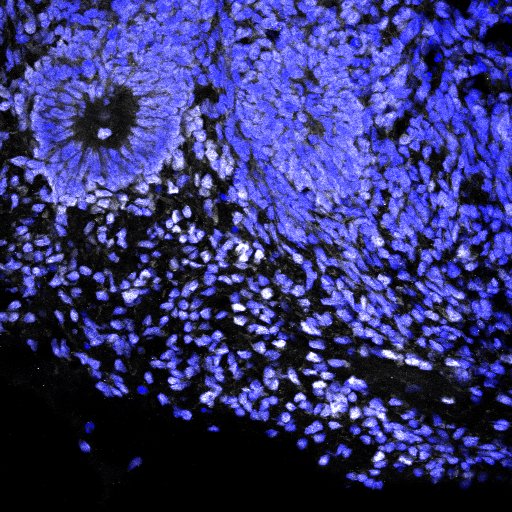

Supplement: Supplementary file 4 — Additional file 4. Uncropped gel and microscopy images. [file 13059_2023_3037_MOESM4_ESM.zip › Gel_Microscopy_images_GenomeBiology/microscopy_images/Figure 7/7e/fig7e_H4R3me2s_day50_ctrl_c1-1.jpg]

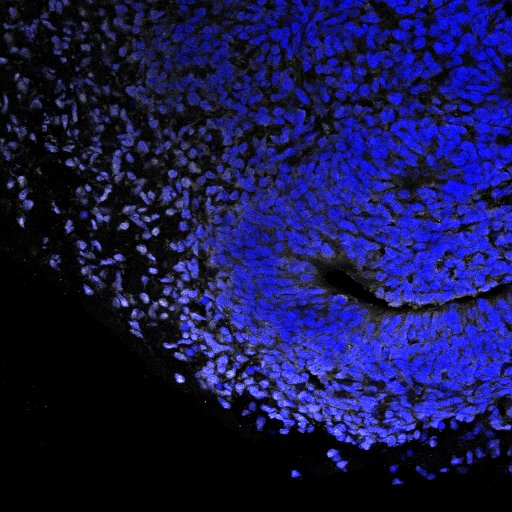

Supplement: Supplementary file 4 — Additional file 4. Uncropped gel and microscopy images. [file 13059_2023_3037_MOESM4_ESM.zip › Gel_Microscopy_images_GenomeBiology/microscopy_images/Figure 7/7e/fig7e_H4R3me2s_day50_oxr1_c2-1.jpg]

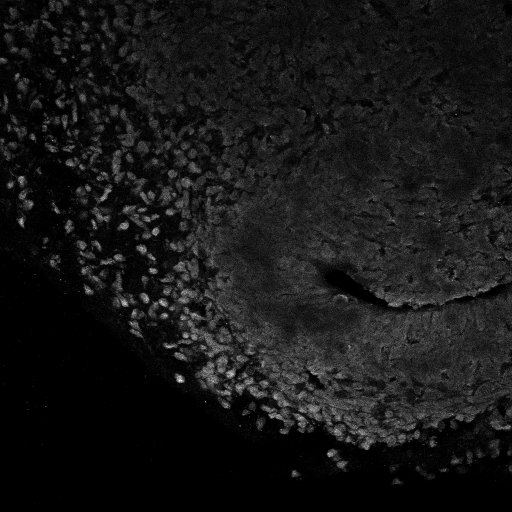

Supplement: Supplementary file 4 — Additional file 4. Uncropped gel and microscopy images. [file 13059_2023_3037_MOESM4_ESM.zip › Gel_Microscopy_images_GenomeBiology/microscopy_images/Figure 7/7e/fig7e_H4R3me2s_day50_oxr1_c2-2.jpg]

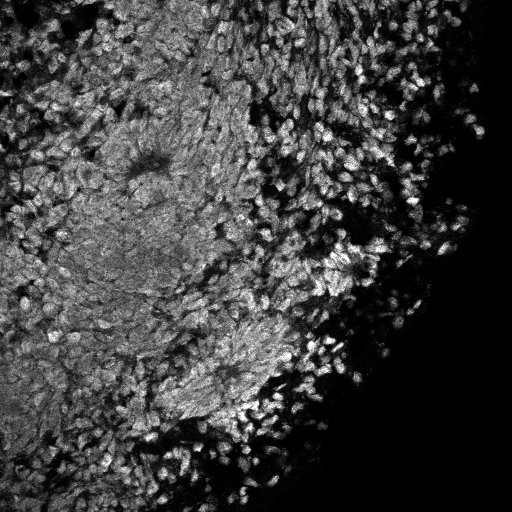

Supplement: Supplementary file 4 — Additional file 4. Uncropped gel and microscopy images. [file 13059_2023_3037_MOESM4_ESM.zip › Gel_Microscopy_images_GenomeBiology/microscopy_images/Figure 7/7e/fig7e_H4R3me2s_day50_ctrl_c2-2.jpg]

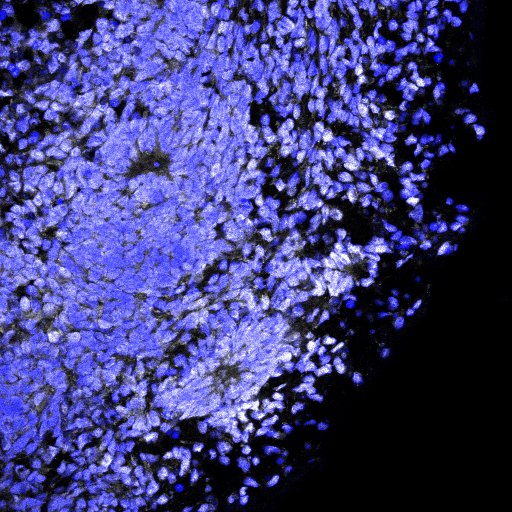

Supplement: Supplementary file 4 — Additional file 4. Uncropped gel and microscopy images. [file 13059_2023_3037_MOESM4_ESM.zip › Gel_Microscopy_images_GenomeBiology/microscopy_images/Figure 7/7e/fig7e_H4R3me2s_day50_ctrl_c2-1.jpg]

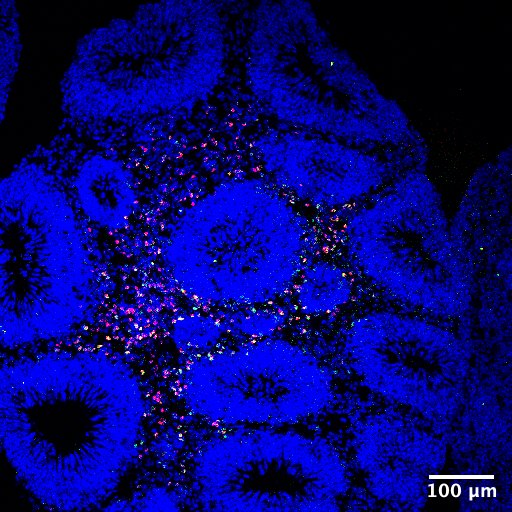

Supplement: Supplementary file 4 — Additional file 4. Uncropped gel and microscopy images. [file 13059_2023_3037_MOESM4_ESM.zip › Gel_Microscopy_images_GenomeBiology/microscopy_images/Figure S5/s5a/S5a_TBR1_CTIP2_Day30_ctrl.jpg]

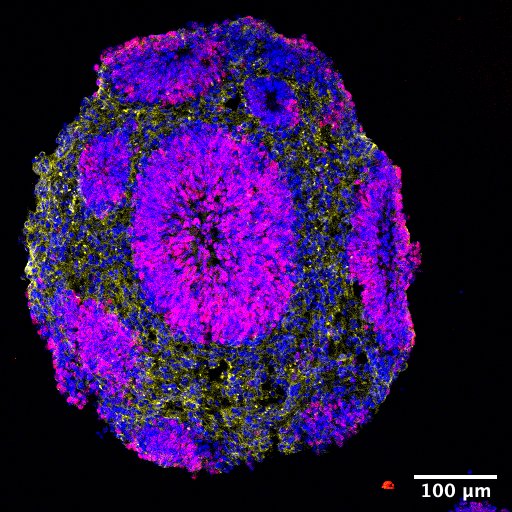

Supplement: Supplementary file 4 — Additional file 4. Uncropped gel and microscopy images. [file 13059_2023_3037_MOESM4_ESM.zip › Gel_Microscopy_images_GenomeBiology/microscopy_images/Figure S5/s5a/S5a_Tuj1_sox2-Day30_ctrl.jpg]

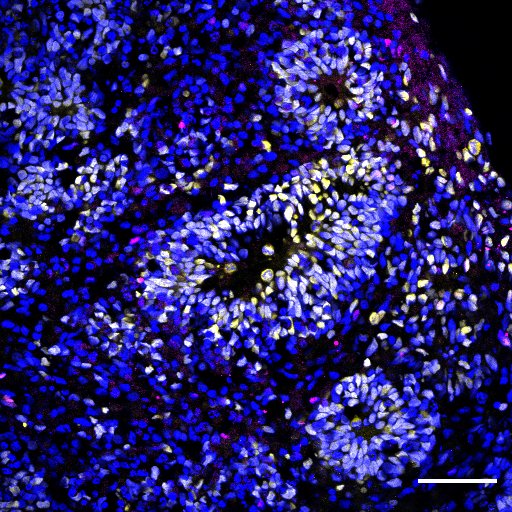

Supplement: Supplementary file 4 — Additional file 4. Uncropped gel and microscopy images. [file 13059_2023_3037_MOESM4_ESM.zip › Gel_Microscopy_images_GenomeBiology/microscopy_images/Figure 6/6b/fig6b_SOX2_CTIP2_day50_oxr1_c3.jpg]

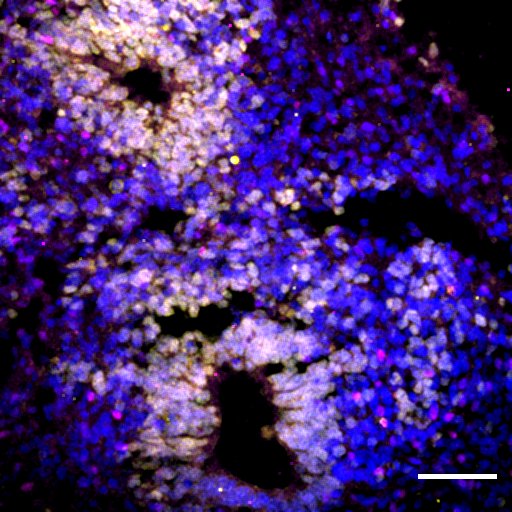

Supplement: Supplementary file 4 — Additional file 4. Uncropped gel and microscopy images. [file 13059_2023_3037_MOESM4_ESM.zip › Gel_Microscopy_images_GenomeBiology/microscopy_images/Figure 6/6b/fig6b_SOX2_CTIP2_day50_oxr1_c1.jpg]

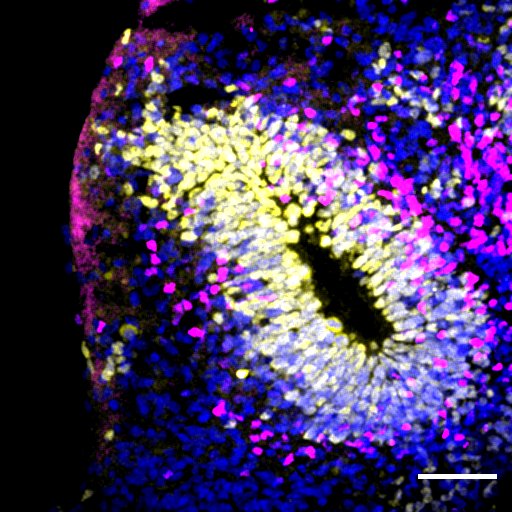

Supplement: Supplementary file 4 — Additional file 4. Uncropped gel and microscopy images. [file 13059_2023_3037_MOESM4_ESM.zip › Gel_Microscopy_images_GenomeBiology/microscopy_images/Figure 6/6b/fig6b_SOX2_CTIP2_day50_ctrl_c1.jpg]

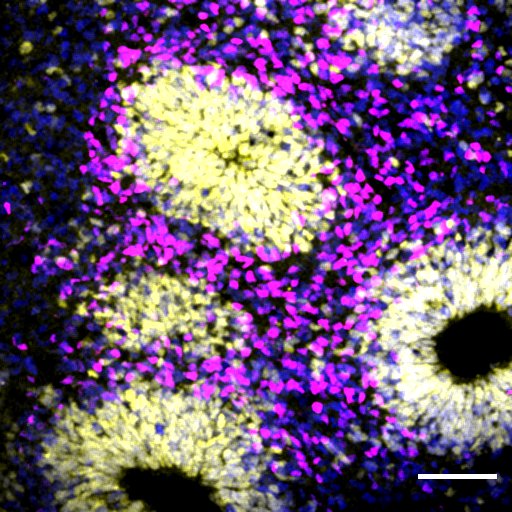

Supplement: Supplementary file 4 — Additional file 4. Uncropped gel and microscopy images. [file 13059_2023_3037_MOESM4_ESM.zip › Gel_Microscopy_images_GenomeBiology/microscopy_images/Figure 6/6b/fig6b_SOX2_CTIP2_day50_ctrl_c2.jpg]

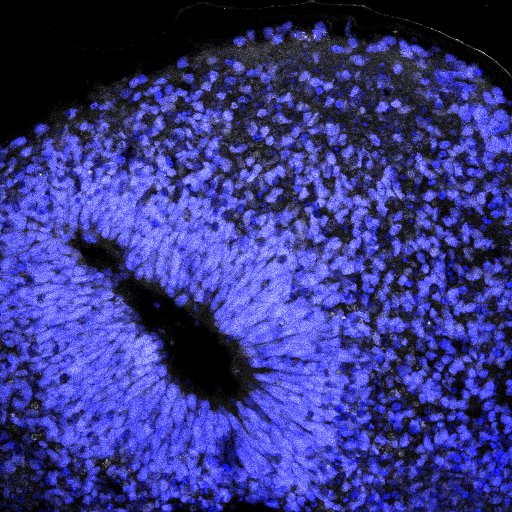

Supplement: Supplementary file 4 — Additional file 4. Uncropped gel and microscopy images. [file 13059_2023_3037_MOESM4_ESM.zip › Gel_Microscopy_images_GenomeBiology/microscopy_images/Figure 6/6e/fig6e_H3R2me2s_day50_ctrl_c2-1.jpg]

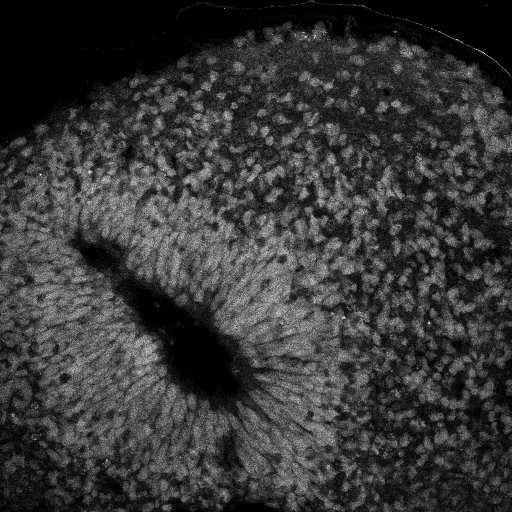

Supplement: Supplementary file 4 — Additional file 4. Uncropped gel and microscopy images. [file 13059_2023_3037_MOESM4_ESM.zip › Gel_Microscopy_images_GenomeBiology/microscopy_images/Figure 6/6e/fig6e_H3R2me2s_day50_ctrl_c2-2.jpg]

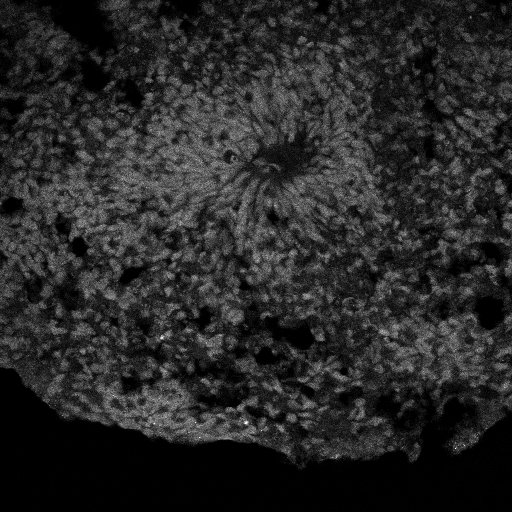

Supplement: Supplementary file 4 — Additional file 4. Uncropped gel and microscopy images. [file 13059_2023_3037_MOESM4_ESM.zip › Gel_Microscopy_images_GenomeBiology/microscopy_images/Figure 6/6e/fig6e_H3R2me2s_day50_oxr1_c1-2.jpg]

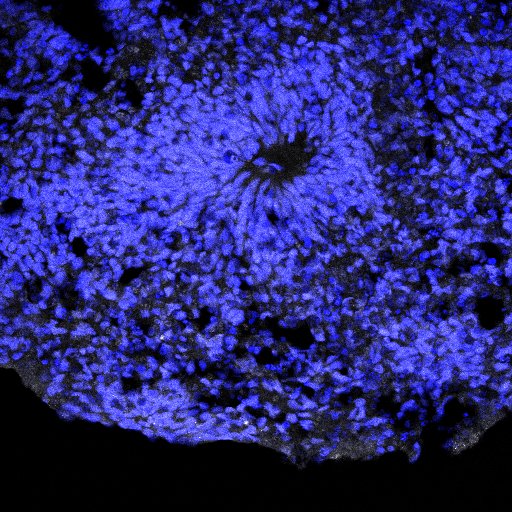

Supplement: Supplementary file 4 — Additional file 4. Uncropped gel and microscopy images. [file 13059_2023_3037_MOESM4_ESM.zip › Gel_Microscopy_images_GenomeBiology/microscopy_images/Figure 6/6e/fig6e_H3R2me2s_day50_oxr1_c1-1.jpg]

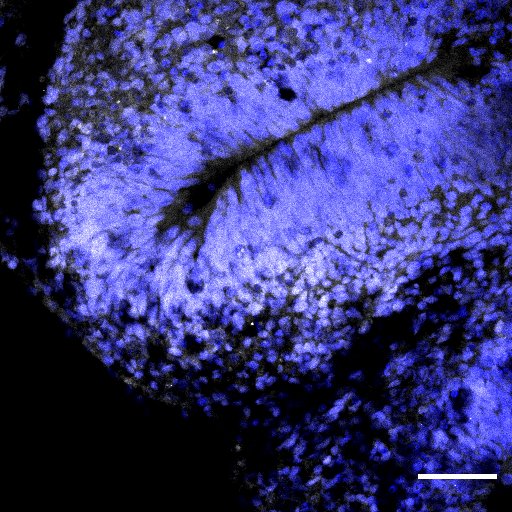

Supplement: Supplementary file 4 — Additional file 4. Uncropped gel and microscopy images. [file 13059_2023_3037_MOESM4_ESM.zip › Gel_Microscopy_images_GenomeBiology/microscopy_images/Figure 6/6e/fig6e_H3R2me2s_day50_ctrl_c1-1.jpg]

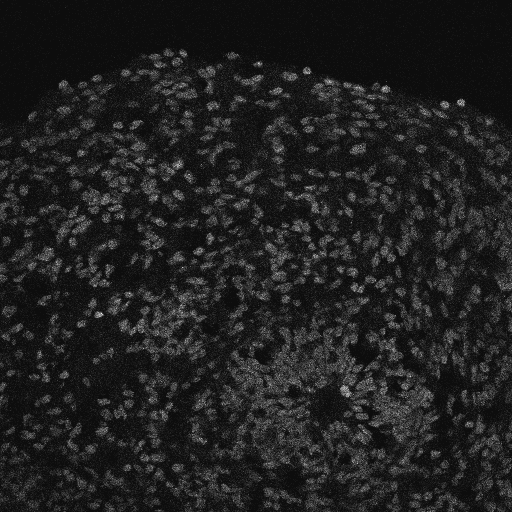

Supplement: Supplementary file 4 — Additional file 4. Uncropped gel and microscopy images. [file 13059_2023_3037_MOESM4_ESM.zip › Gel_Microscopy_images_GenomeBiology/microscopy_images/Figure 6/6e/fig6e_H3R2me2s_day50_oxr1_c2-2.tif]

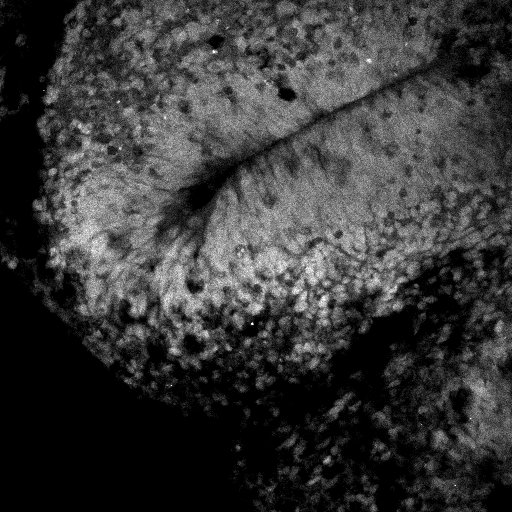

Supplement: Supplementary file 4 — Additional file 4. Uncropped gel and microscopy images. [file 13059_2023_3037_MOESM4_ESM.zip › Gel_Microscopy_images_GenomeBiology/microscopy_images/Figure 6/6e/fig6e_H3R2me2s_day50_ctrl_c1-2.jpg]

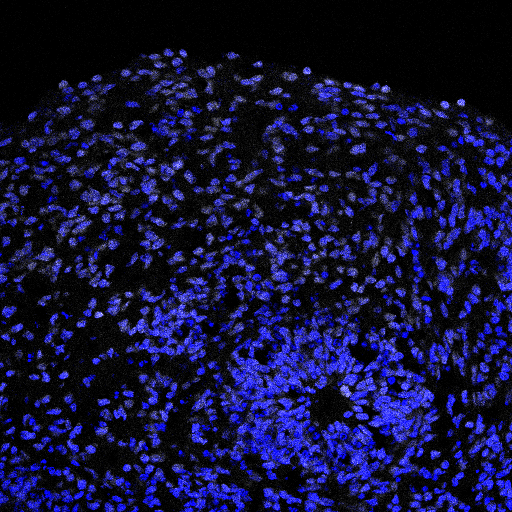

Supplement: Supplementary file 4 — Additional file 4. Uncropped gel and microscopy images. [file 13059_2023_3037_MOESM4_ESM.zip › Gel_Microscopy_images_GenomeBiology/microscopy_images/Figure 6/6e/fig6e_H3R2me2s_day50_oxr1_c2-1.tif]

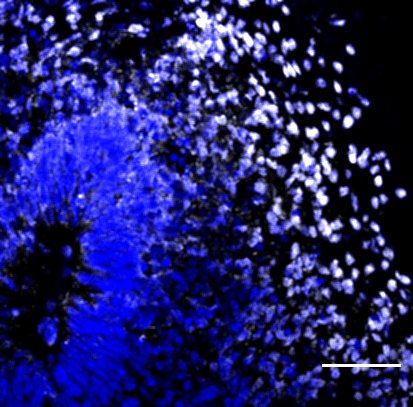

Supplement: Supplementary file 4 — Additional file 4. Uncropped gel and microscopy images. [file 13059_2023_3037_MOESM4_ESM.zip › Gel_Microscopy_images_GenomeBiology/microscopy_images/Figure 6/6d/fig6d_H4R3me2s_day50_ctrl_c1.jpg]

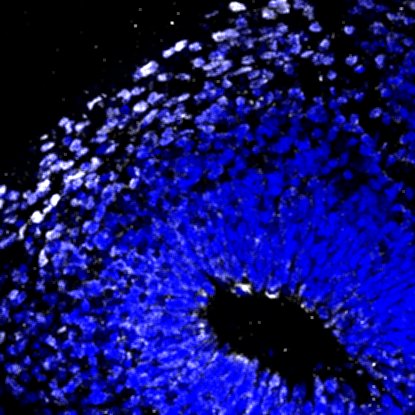

Supplement: Supplementary file 4 — Additional file 4. Uncropped gel and microscopy images. [file 13059_2023_3037_MOESM4_ESM.zip › Gel_Microscopy_images_GenomeBiology/microscopy_images/Figure 6/6d/fig6d_H4R3me2s_day50_ctrl_c2.jpg]

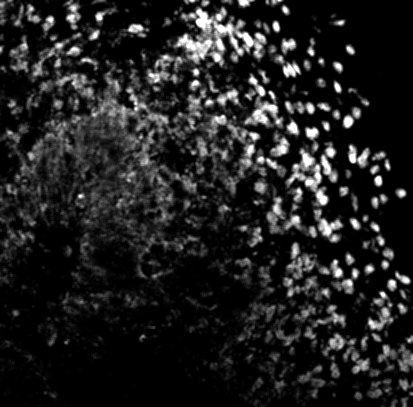

Supplement: Supplementary file 4 — Additional file 4. Uncropped gel and microscopy images. [file 13059_2023_3037_MOESM4_ESM.zip › Gel_Microscopy_images_GenomeBiology/microscopy_images/Figure 6/6d/fig6d_H4R3me2s_day50_ctrl_c1-2.jpg]

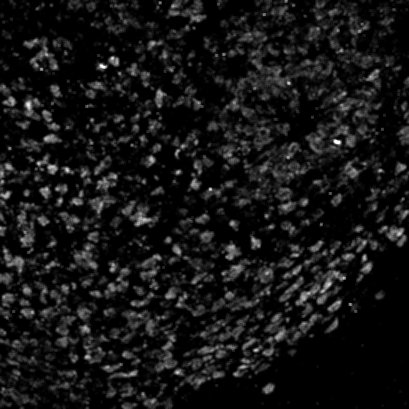

Supplement: Supplementary file 4 — Additional file 4. Uncropped gel and microscopy images. [file 13059_2023_3037_MOESM4_ESM.zip › Gel_Microscopy_images_GenomeBiology/microscopy_images/Figure 6/6d/fig6d_H4R3me2s_day50_oxr1_c1-2.jpg]

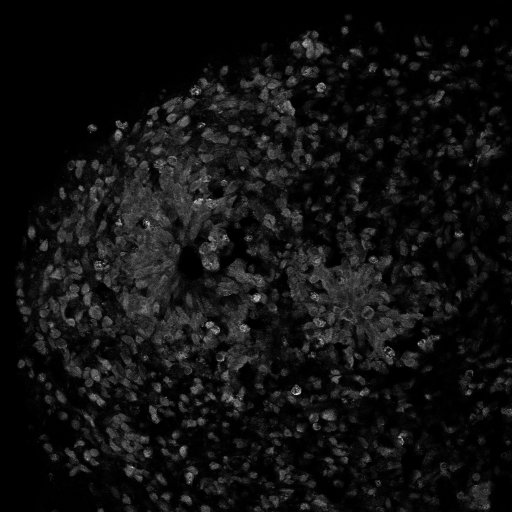

Supplement: Supplementary file 4 — Additional file 4. Uncropped gel and microscopy images. [file 13059_2023_3037_MOESM4_ESM.zip › Gel_Microscopy_images_GenomeBiology/microscopy_images/Figure 6/6d/fig6d_H4R3me2s_day50_oxr1_c3-2.jpg]

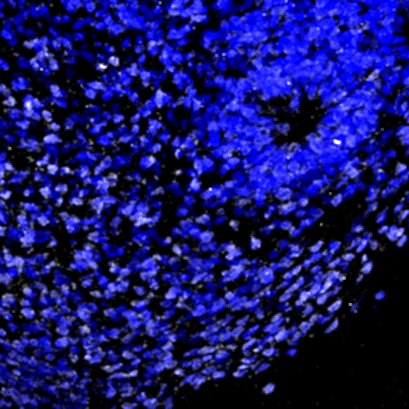

Supplement: Supplementary file 4 — Additional file 4. Uncropped gel and microscopy images. [file 13059_2023_3037_MOESM4_ESM.zip › Gel_Microscopy_images_GenomeBiology/microscopy_images/Figure 6/6d/fig6d_H4R3me2s_day50_oxr1_c1.jpg]

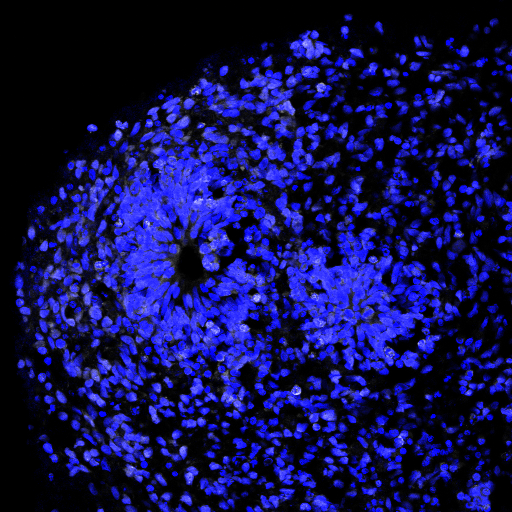

Supplement: Supplementary file 4 — Additional file 4. Uncropped gel and microscopy images. [file 13059_2023_3037_MOESM4_ESM.zip › Gel_Microscopy_images_GenomeBiology/microscopy_images/Figure 6/6d/fig6d_H4R3me2s_day50_oxr1_c3-1.tif]

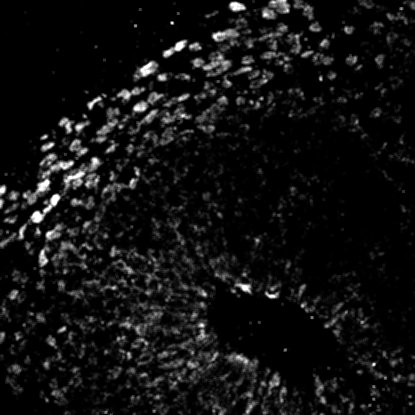

Supplement: Supplementary file 4 — Additional file 4. Uncropped gel and microscopy images. [file 13059_2023_3037_MOESM4_ESM.zip › Gel_Microscopy_images_GenomeBiology/microscopy_images/Figure 6/6d/fig6d_H4R3me2s_day50_ctrl_c2-2.jpg]

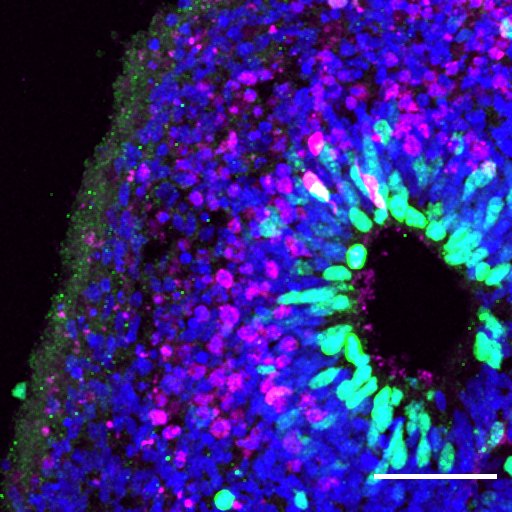

Supplement: Supplementary file 4 — Additional file 4. Uncropped gel and microscopy images. [file 13059_2023_3037_MOESM4_ESM.zip › Gel_Microscopy_images_GenomeBiology/microscopy_images/Figure 6/6c/fig6c_cCAS3_KI67_day50_oxr1_c1.jpg]

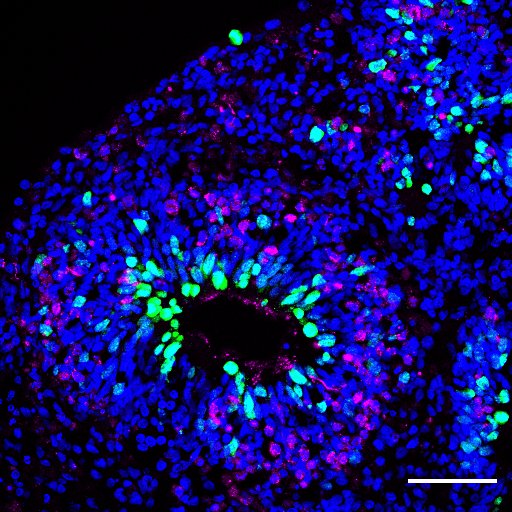

Supplement: Supplementary file 4 — Additional file 4. Uncropped gel and microscopy images. [file 13059_2023_3037_MOESM4_ESM.zip › Gel_Microscopy_images_GenomeBiology/microscopy_images/Figure 6/6c/fig6c_cCAS3_KI67_day50_oxr1_c3.jpg]

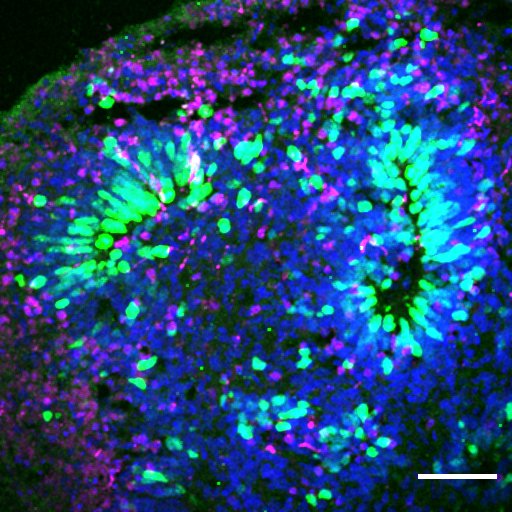

Supplement: Supplementary file 4 — Additional file 4. Uncropped gel and microscopy images. [file 13059_2023_3037_MOESM4_ESM.zip › Gel_Microscopy_images_GenomeBiology/microscopy_images/Figure 6/6c/fig6c_cCAS3_KI67_day50_ctrl_c2.jpg]

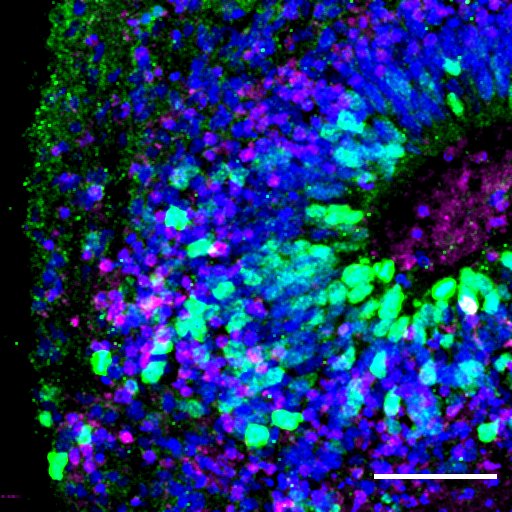

Supplement: Supplementary file 4 — Additional file 4. Uncropped gel and microscopy images. [file 13059_2023_3037_MOESM4_ESM.zip › Gel_Microscopy_images_GenomeBiology/microscopy_images/Figure 6/6c/fig6c_cCAS3_KI67_day50_ctrl_c1.jpg]

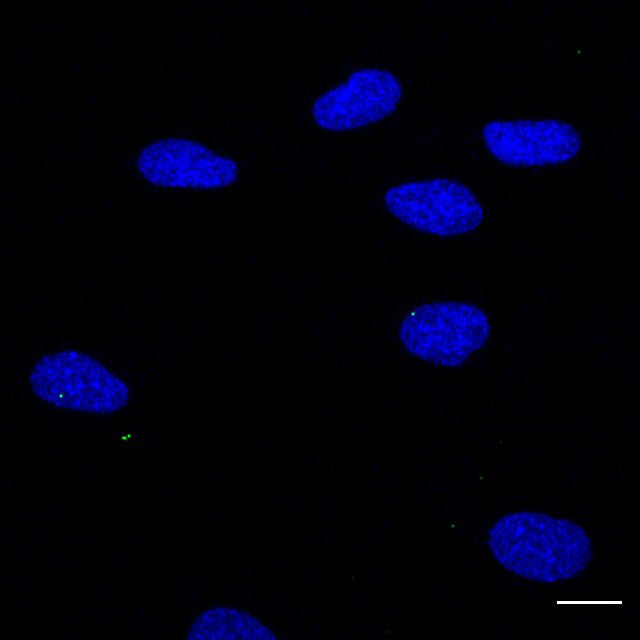

Supplement: Supplementary file 4 — Additional file 4. Uncropped gel and microscopy images. [file 13059_2023_3037_MOESM4_ESM.zip › Gel_Microscopy_images_GenomeBiology/microscopy_images/Figure 4/4a/Fig4a_PRTM5_oxr1.jpg]

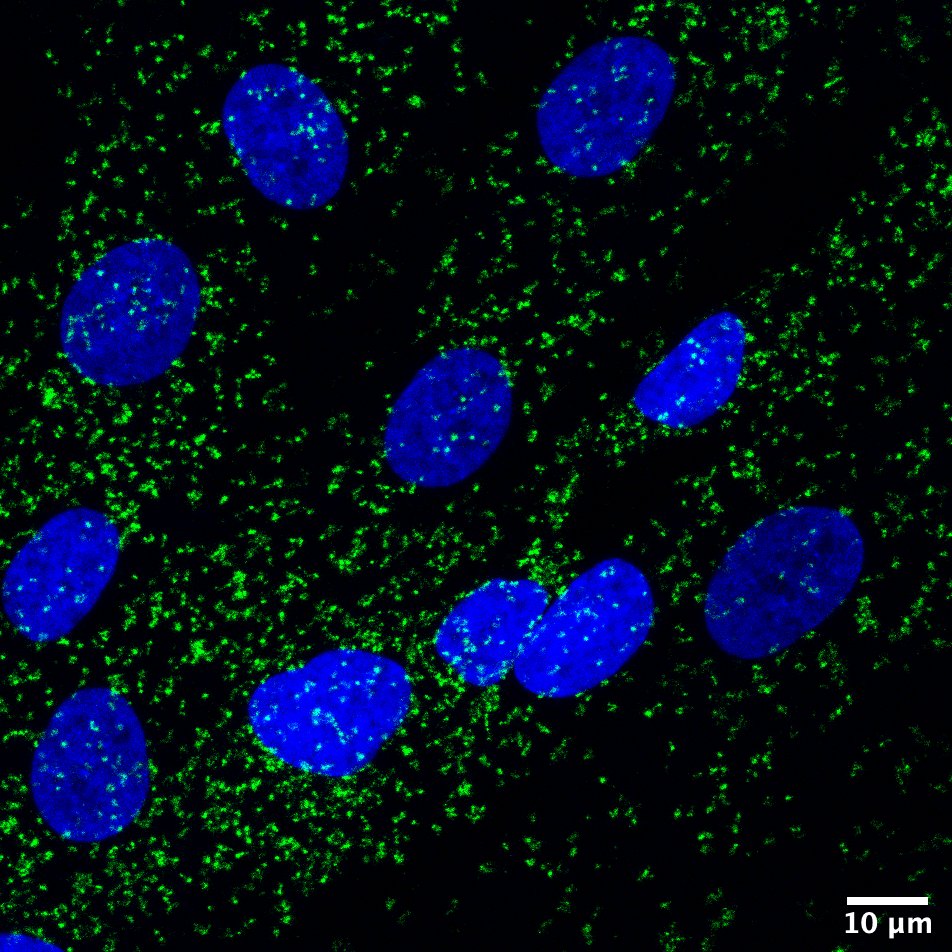

Supplement: Supplementary file 4 — Additional file 4. Uncropped gel and microscopy images. [file 13059_2023_3037_MOESM4_ESM.zip › Gel_Microscopy_images_GenomeBiology/microscopy_images/Figure 4/4a/Fig4a_PRMT5_ctrl.jpg]

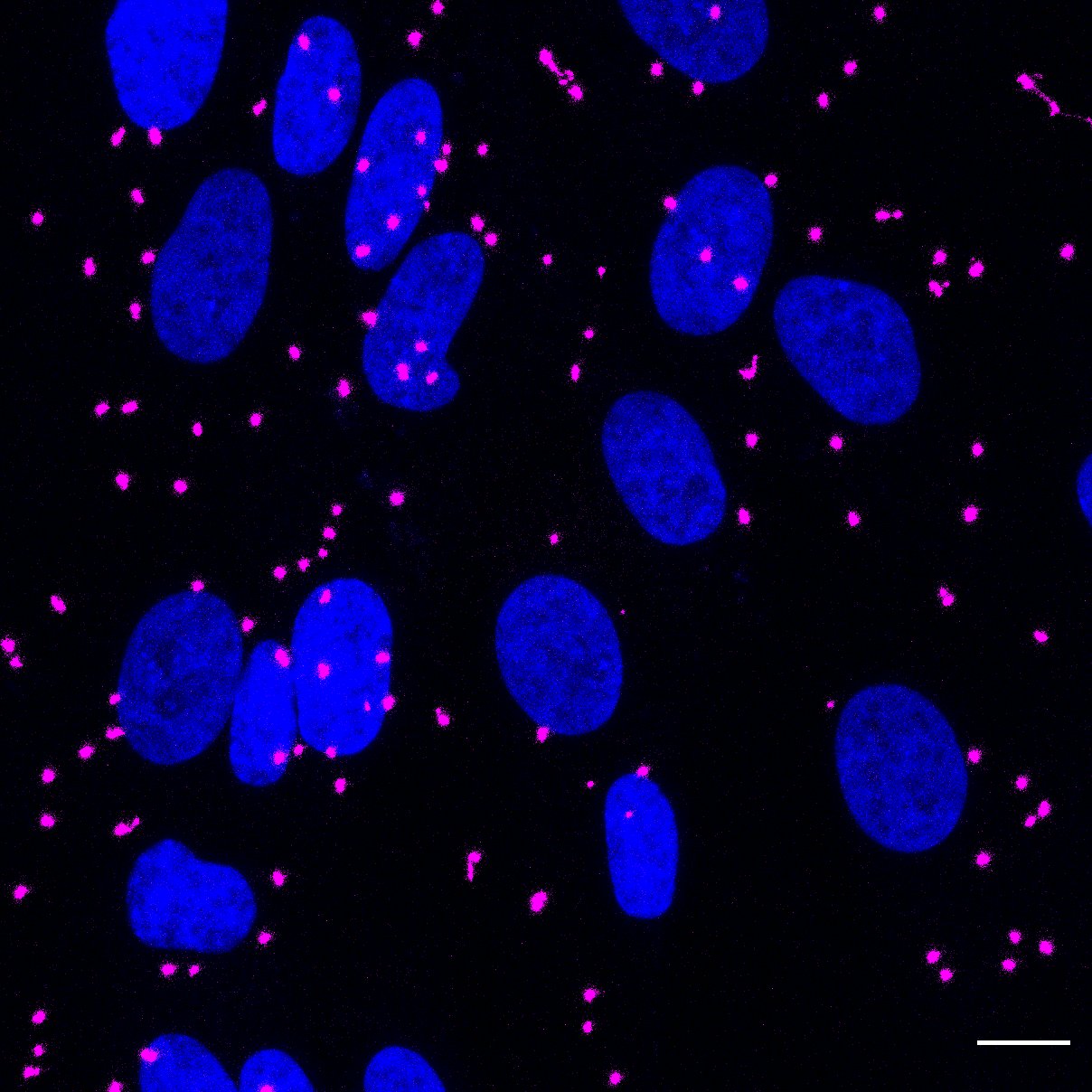

Supplement: Supplementary file 4 — Additional file 4. Uncropped gel and microscopy images. [file 13059_2023_3037_MOESM4_ESM.zip › Gel_Microscopy_images_GenomeBiology/microscopy_images/Figure 4/4a/Fig4a_PRTM1_ctrl.jpg]

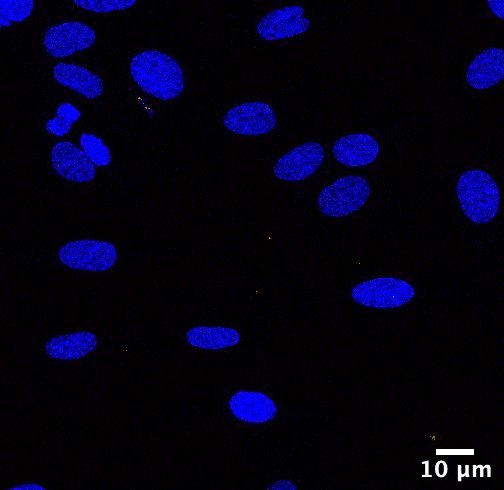

Supplement: Supplementary file 4 — Additional file 4. Uncropped gel and microscopy images. [file 13059_2023_3037_MOESM4_ESM.zip › Gel_Microscopy_images_GenomeBiology/microscopy_images/Figure 4/4a/Fig4a_PRMT1_oxr1.jpg]

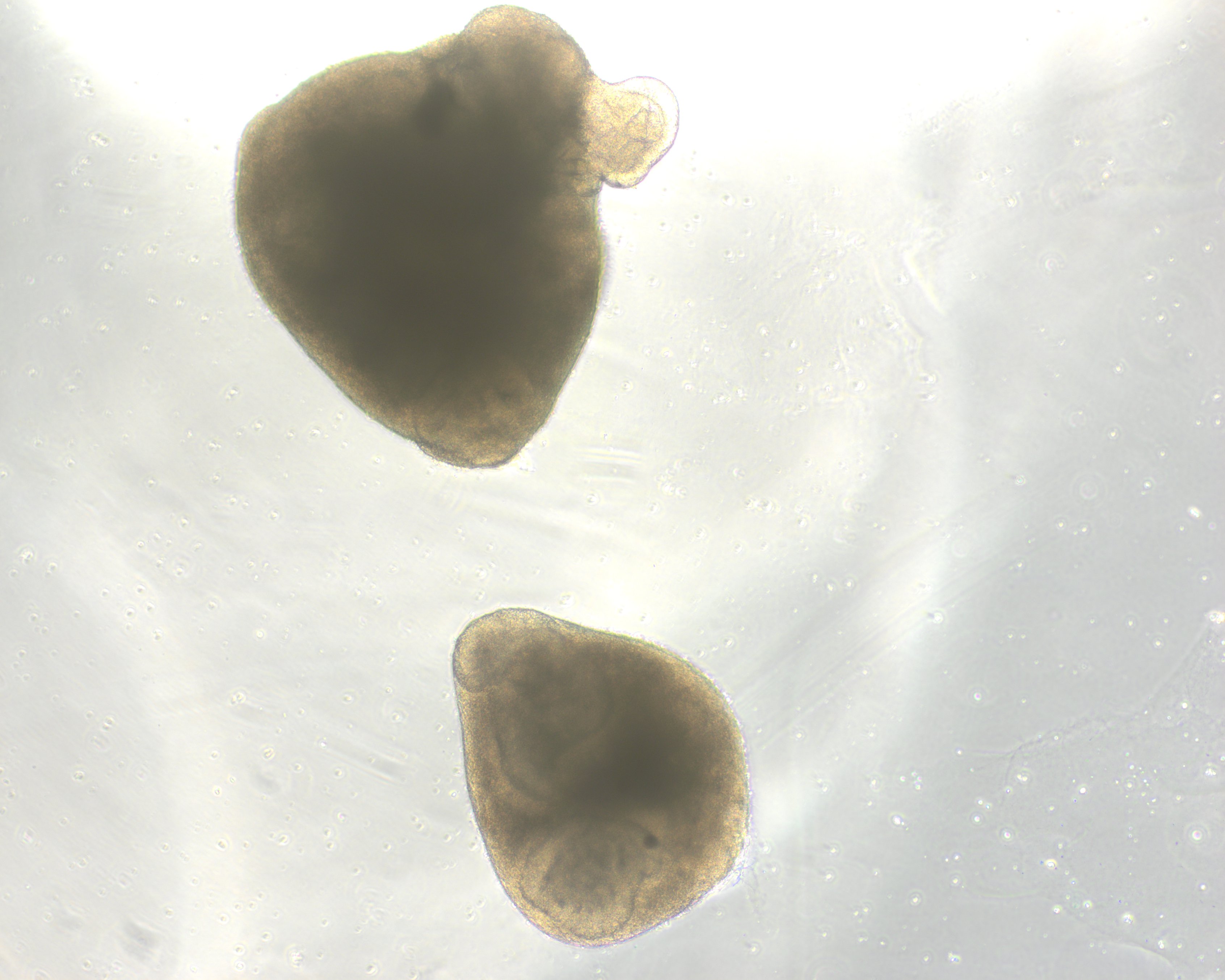

Supplement: Supplementary file 4 — Additional file 4. Uncropped gel and microscopy images. [file 13059_2023_3037_MOESM4_ESM.zip › Gel_Microscopy_images_GenomeBiology/microscopy_images/Figure S6/s6a/ctrl_c1_day15.jpg]

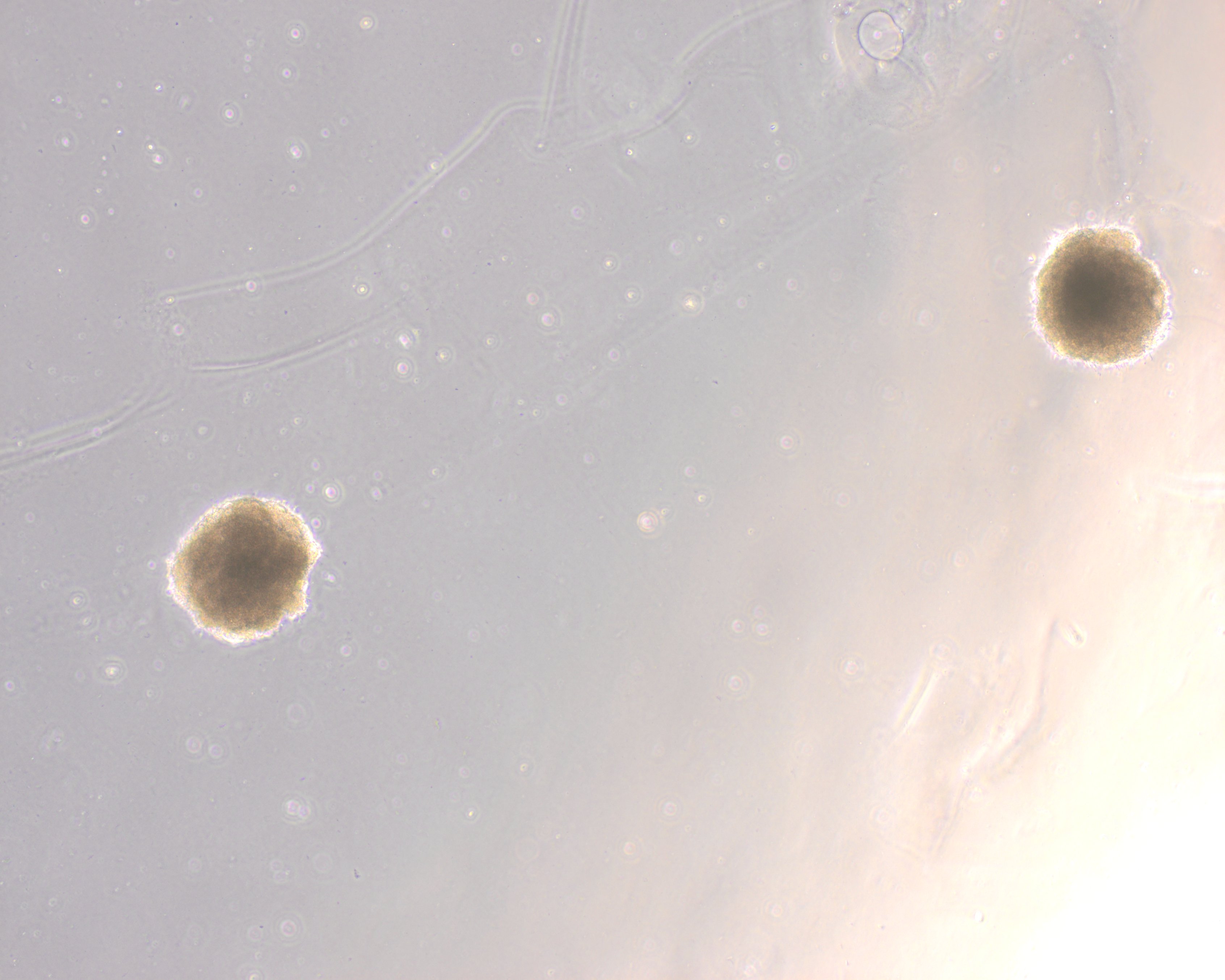

Supplement: Supplementary file 4 — Additional file 4. Uncropped gel and microscopy images. [file 13059_2023_3037_MOESM4_ESM.zip › Gel_Microscopy_images_GenomeBiology/microscopy_images/Figure S6/s6a/oxr1_c3_day15.jpg]

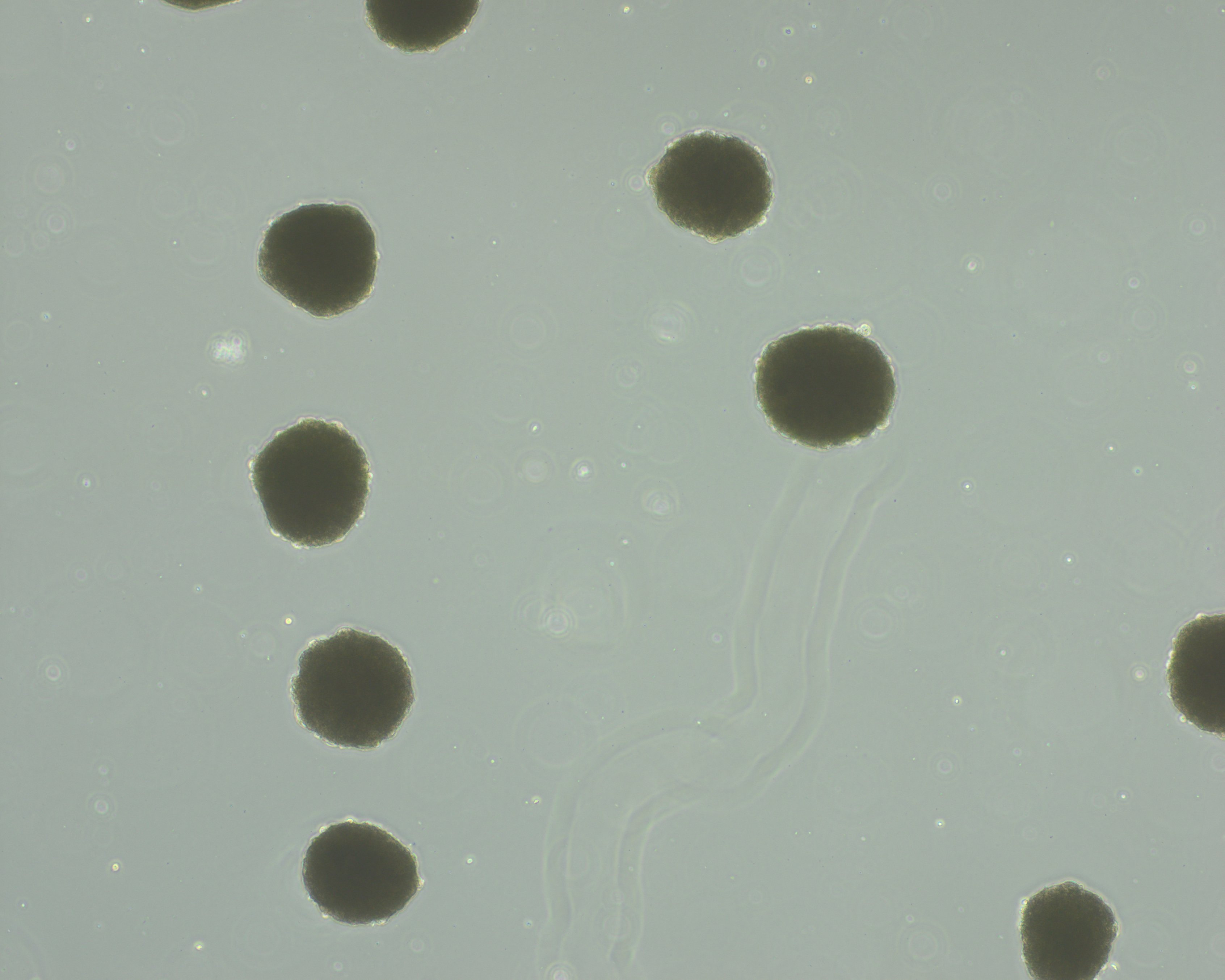

Supplement: Supplementary file 4 — Additional file 4. Uncropped gel and microscopy images. [file 13059_2023_3037_MOESM4_ESM.zip › Gel_Microscopy_images_GenomeBiology/microscopy_images/Figure S6/s6a/oxr1_c3_day5.jpg]

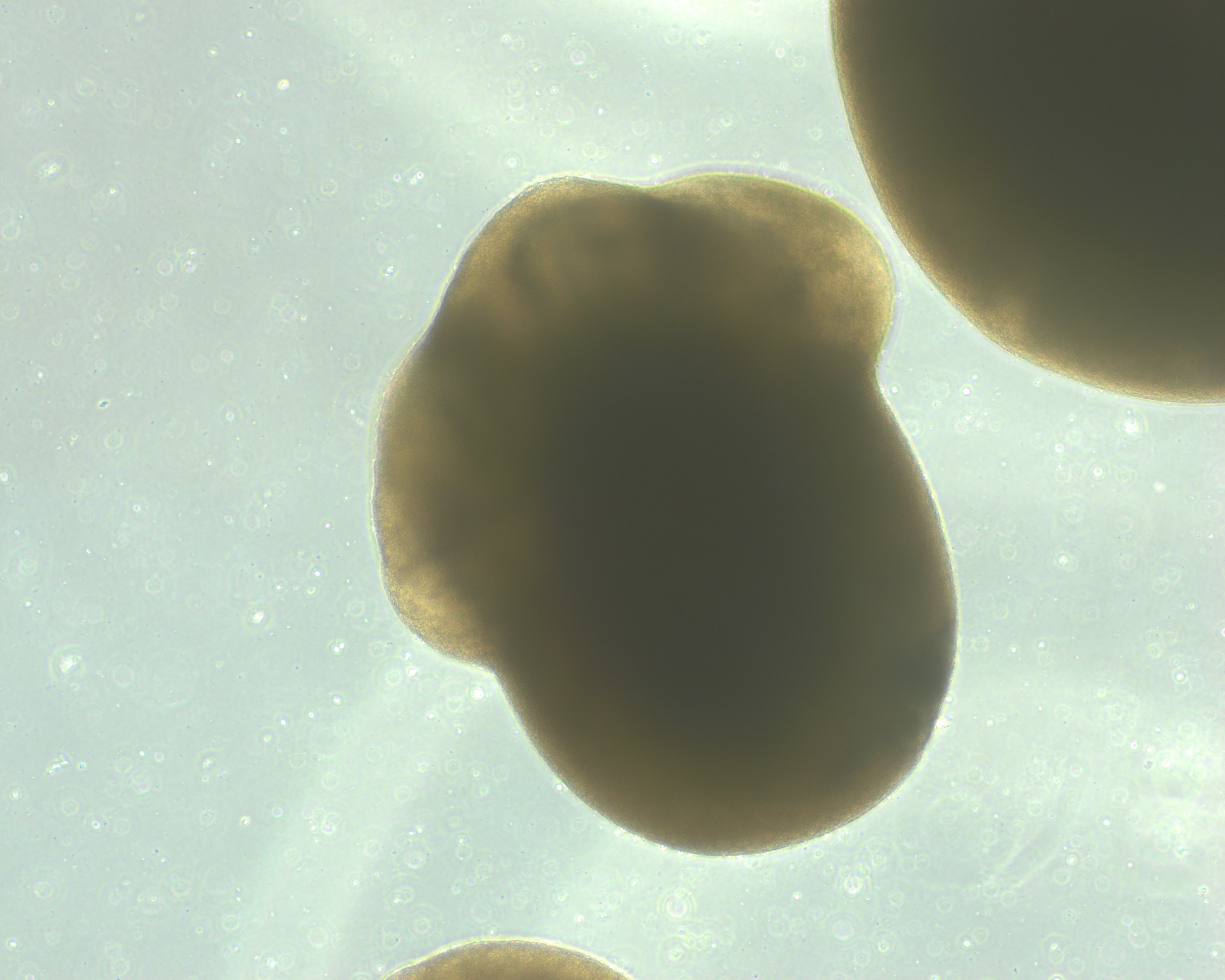

Supplement: Supplementary file 4 — Additional file 4. Uncropped gel and microscopy images. [file 13059_2023_3037_MOESM4_ESM.zip › Gel_Microscopy_images_GenomeBiology/microscopy_images/Figure S6/s6a/ctrl_c1_day50.jpg]

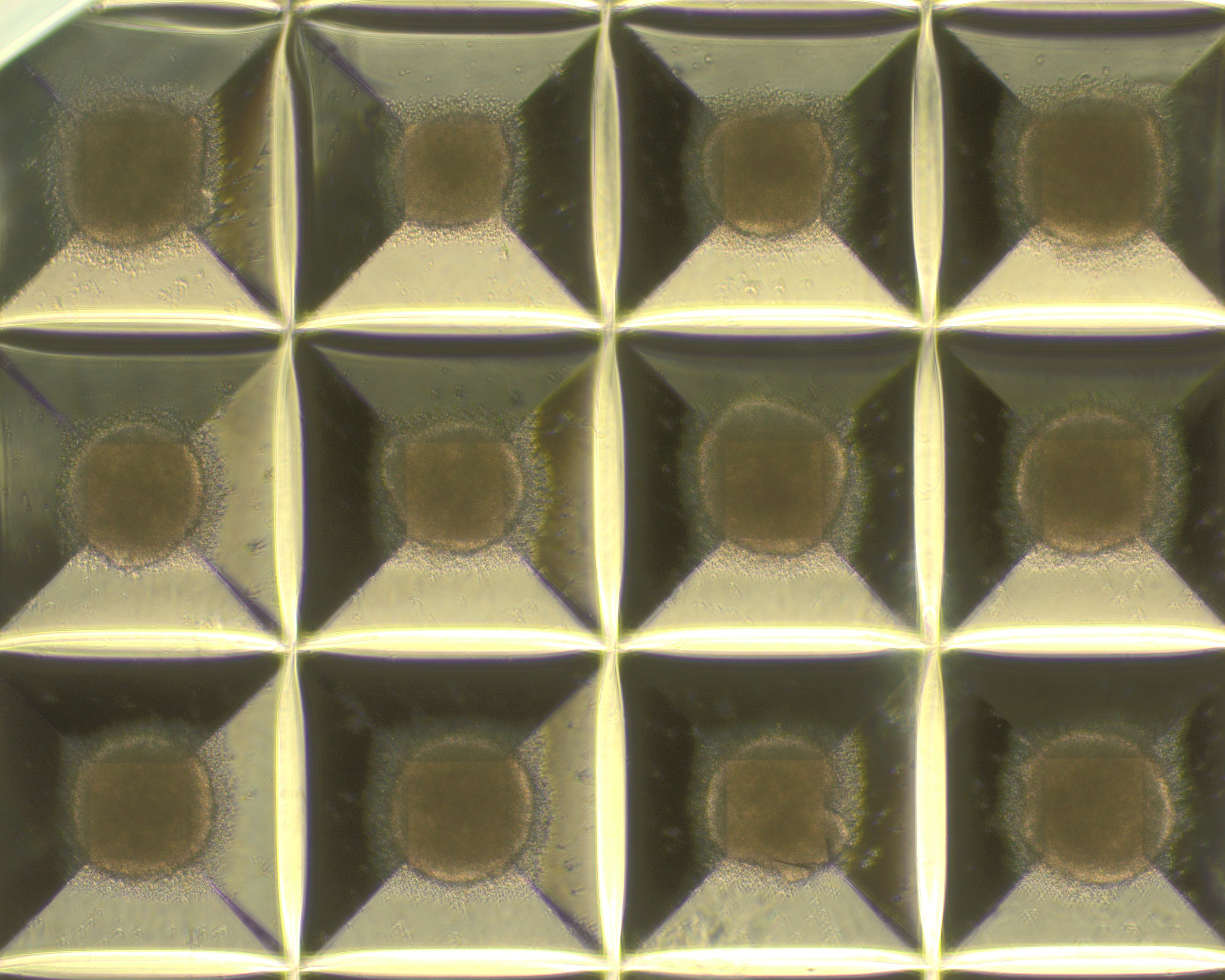

Supplement: Supplementary file 4 — Additional file 4. Uncropped gel and microscopy images. [file 13059_2023_3037_MOESM4_ESM.zip › Gel_Microscopy_images_GenomeBiology/microscopy_images/Figure S6/s6a/oxr1_c3_day2.jpg]

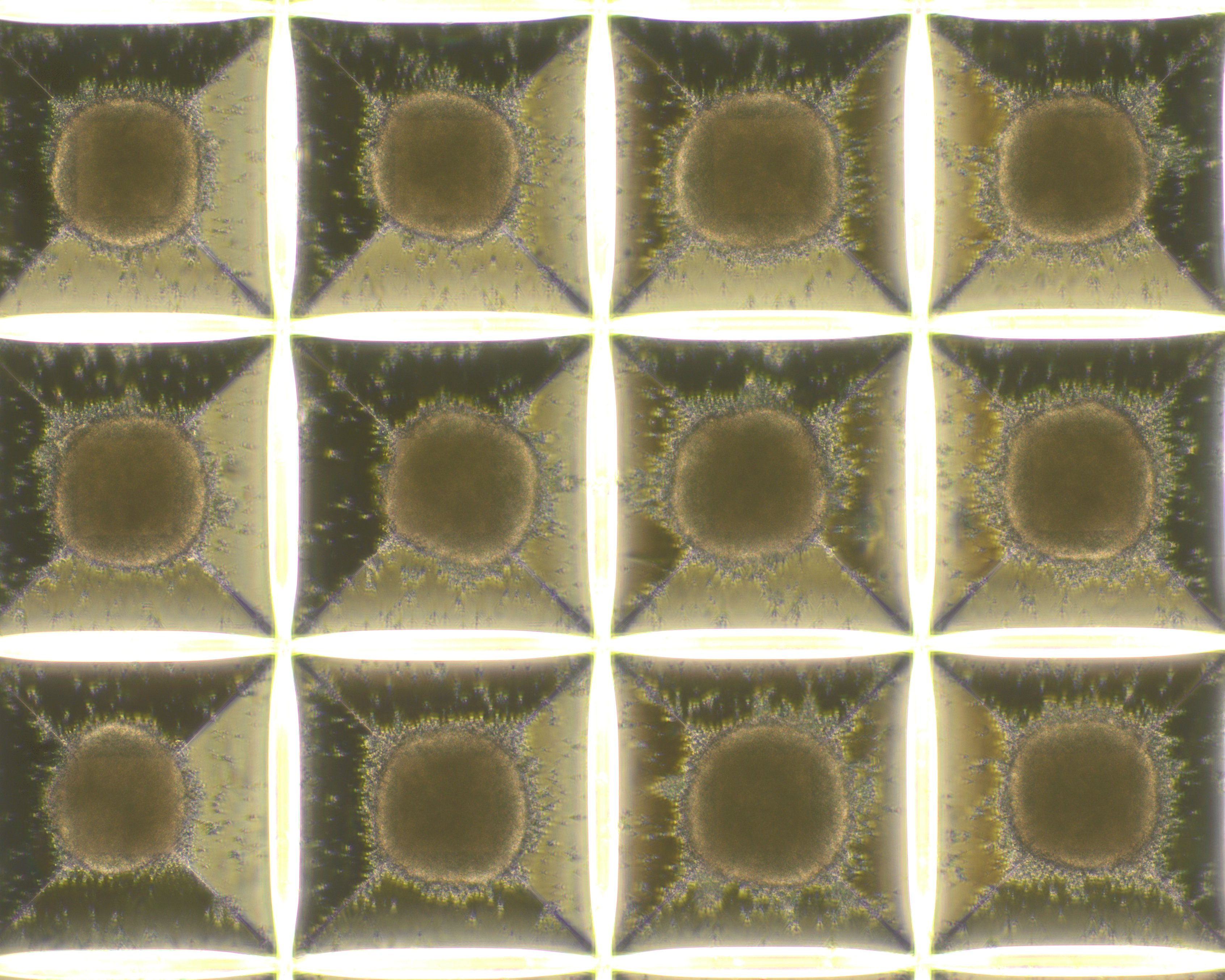

Supplement: Supplementary file 4 — Additional file 4. Uncropped gel and microscopy images. [file 13059_2023_3037_MOESM4_ESM.zip › Gel_Microscopy_images_GenomeBiology/microscopy_images/Figure S6/s6a/ctrl_c1_day2.jpg]

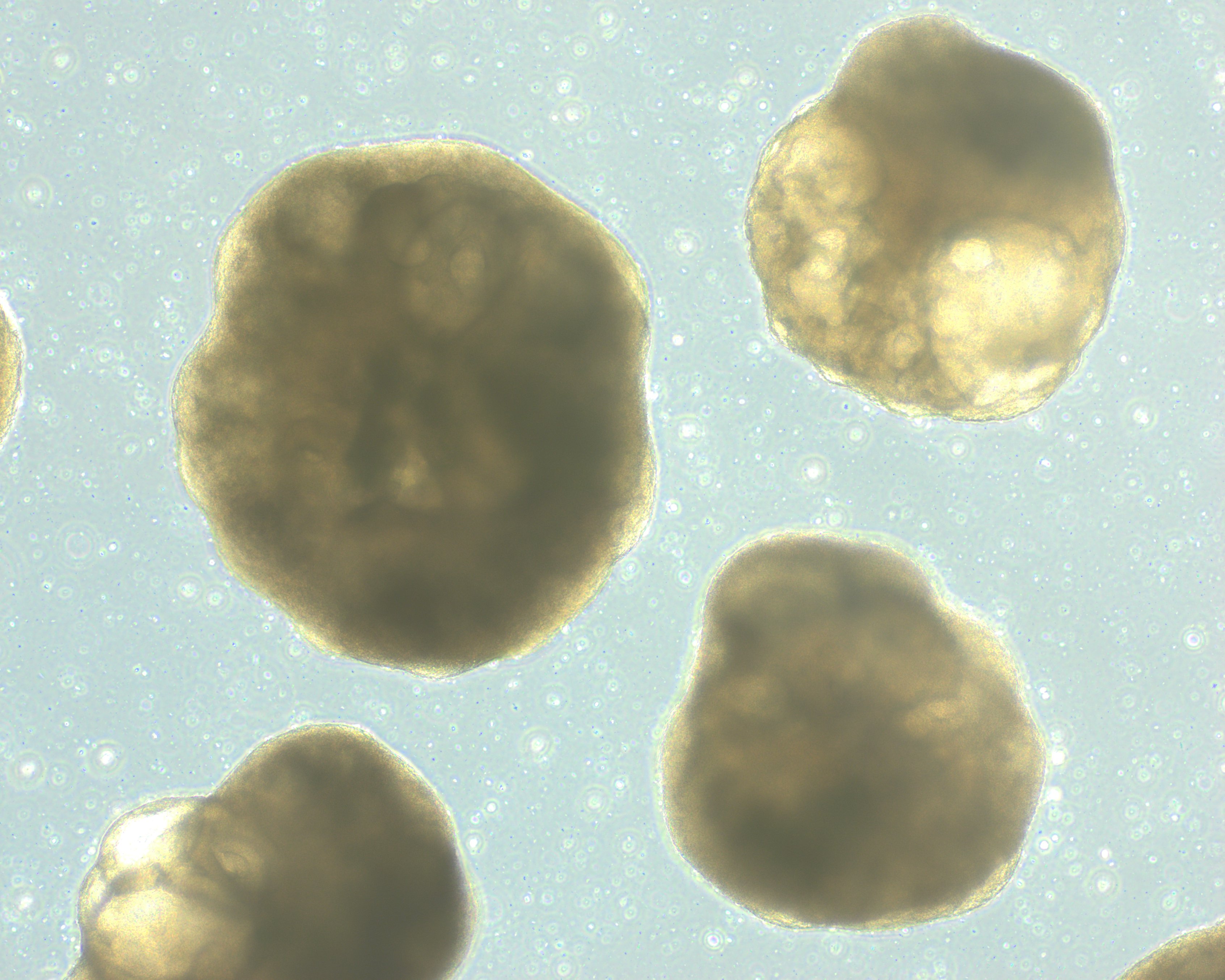

Supplement: Supplementary file 4 — Additional file 4. Uncropped gel and microscopy images. [file 13059_2023_3037_MOESM4_ESM.zip › Gel_Microscopy_images_GenomeBiology/microscopy_images/Figure S6/s6a/oxr1_c3_day50.jpg]

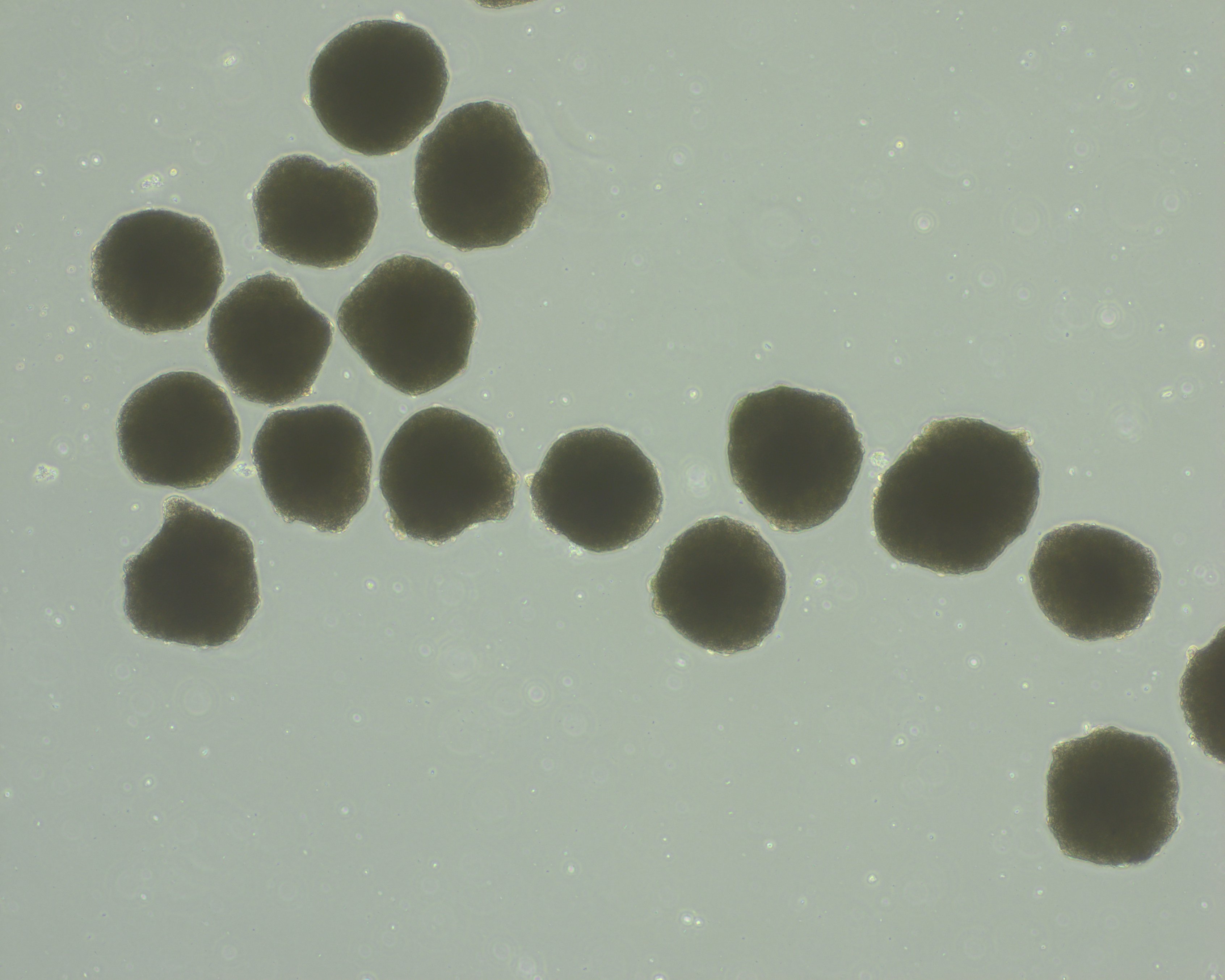

Supplement: Supplementary file 4 — Additional file 4. Uncropped gel and microscopy images. [file 13059_2023_3037_MOESM4_ESM.zip › Gel_Microscopy_images_GenomeBiology/microscopy_images/Figure S6/s6a/ctrl_c1_day5.jpg]

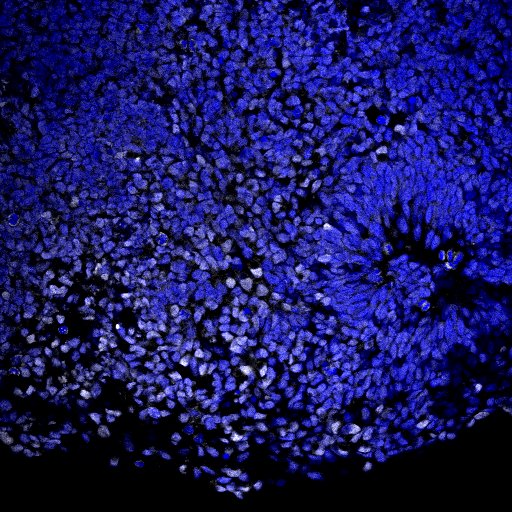

Supplement: Supplementary file 4 — Additional file 4. Uncropped gel and microscopy images. [file 13059_2023_3037_MOESM4_ESM.zip › Gel_Microscopy_images_GenomeBiology/microscopy_images/Figure S7/S7e/s7e_H3R2me2a_day50_oxr1_c1-1.jpg]

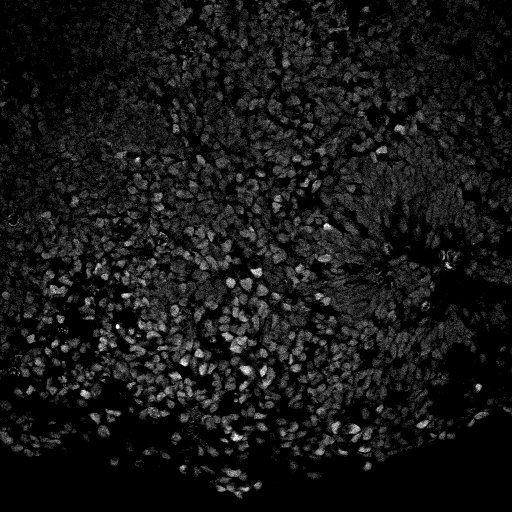

Supplement: Supplementary file 4 — Additional file 4. Uncropped gel and microscopy images. [file 13059_2023_3037_MOESM4_ESM.zip › Gel_Microscopy_images_GenomeBiology/microscopy_images/Figure S7/S7e/s7e_H3R2me2a_day50_oxr1_c1-2.jpg]

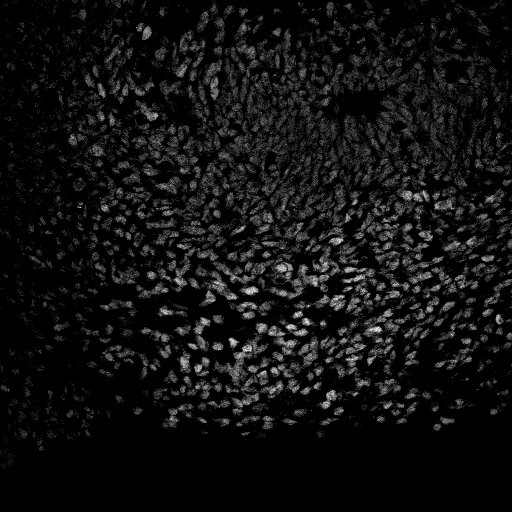

Supplement: Supplementary file 4 — Additional file 4. Uncropped gel and microscopy images. [file 13059_2023_3037_MOESM4_ESM.zip › Gel_Microscopy_images_GenomeBiology/microscopy_images/Figure S7/S7e/s7e_H3R2me2a_day50_ctrl_c1-2.jpg]

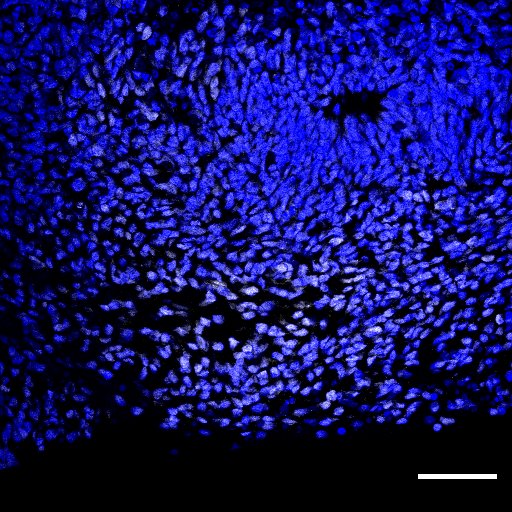

Supplement: Supplementary file 4 — Additional file 4. Uncropped gel and microscopy images. [file 13059_2023_3037_MOESM4_ESM.zip › Gel_Microscopy_images_GenomeBiology/microscopy_images/Figure S7/S7e/s7e_H3R2me2a_day50_ctrl_c1-1.jpg]

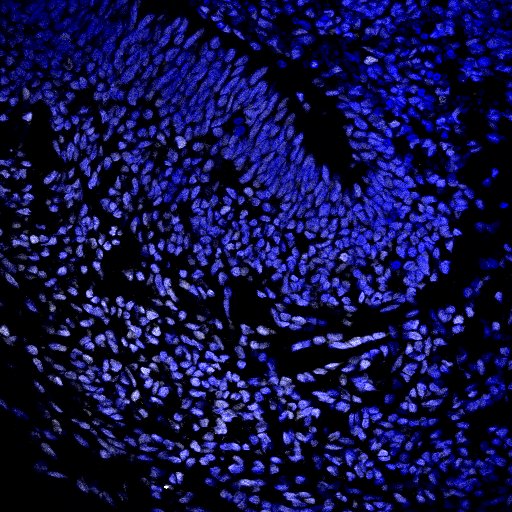

Supplement: Supplementary file 4 — Additional file 4. Uncropped gel and microscopy images. [file 13059_2023_3037_MOESM4_ESM.zip › Gel_Microscopy_images_GenomeBiology/microscopy_images/Figure S7/S7e/s7e_H3R2me2a_day50_oxr1_c2-1.jpg]

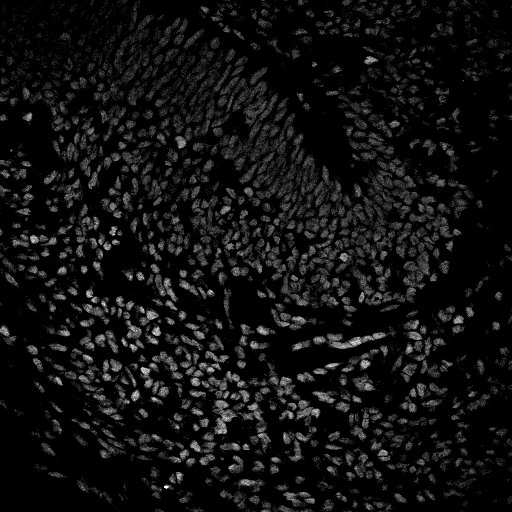

Supplement: Supplementary file 4 — Additional file 4. Uncropped gel and microscopy images. [file 13059_2023_3037_MOESM4_ESM.zip › Gel_Microscopy_images_GenomeBiology/microscopy_images/Figure S7/S7e/s7e_H3R2me2a_day50_oxr1_c2-2.jpg]

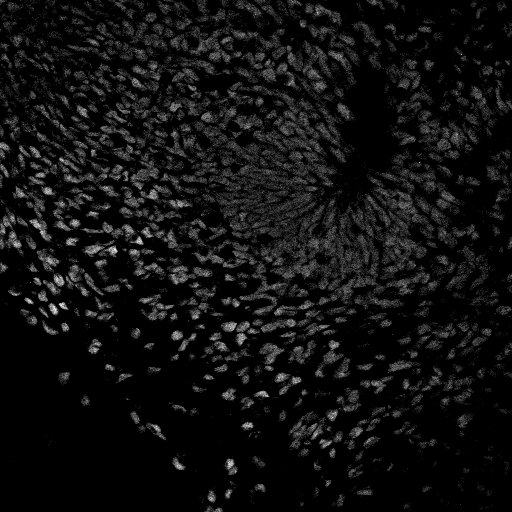

Supplement: Supplementary file 4 — Additional file 4. Uncropped gel and microscopy images. [file 13059_2023_3037_MOESM4_ESM.zip › Gel_Microscopy_images_GenomeBiology/microscopy_images/Figure S7/S7e/s7e_H3R2me2a_day50_ctrl_c2-2.jpg]

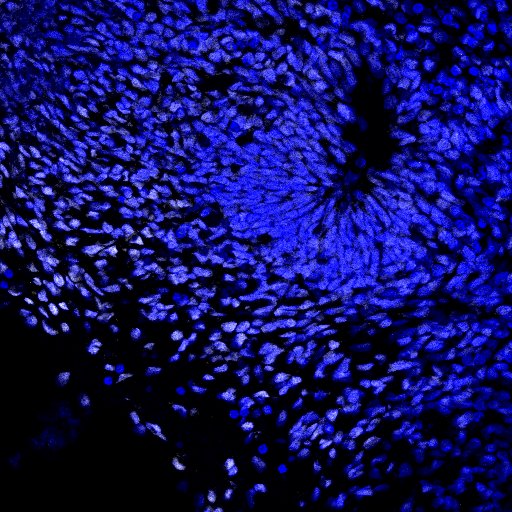

Supplement: Supplementary file 4 — Additional file 4. Uncropped gel and microscopy images. [file 13059_2023_3037_MOESM4_ESM.zip › Gel_Microscopy_images_GenomeBiology/microscopy_images/Figure S7/S7e/s7e_H3R2me2a_day50_ctrl_c2-1.jpg]
