# Supplementary material for: A loss-of-function mutation in human Oxidation Resistance 1 disrupts the spatial–temporal regulation of histone arginine methylation in neurodevelopment
Source: Genome Biol. 2023 Sep 29;24:216. doi: 10.1186/s13059-023-03037-1 (PMC10540402; doi:10.1186/s13059-023-03037-1)
Supplement: Supplementary file 5 — Additional file 5. Peer review history. [file 13059_2023_3037_MOESM5_ESM.docx]

**Review history**

**First round of review**

**Reviewer 1**

Here the authors combine the description of a novel set of patients harboring a recessive loss-of-function mutation in the gene OXR1, followed by a set of cellular experiments to define specific properties of gene 'knockout' in human cells including organoids differentiated from patient iPSCs alongside quantification of histone arginine methylation. The hypothesis is that OXR1 is responsible for maintaining these epigenetic marks via an interaction with protein arginine methyltransferases (PRMTs) and loss of OXR1 leads to disruption of neurodevelopment via this axis. In summary, the data certainly extends previous work and supports previous studies from the submitting authors regarding a role in arginine methylation, yet the considerable variation and quantification methods in the more complex cellular models makes it difficult to define the specific impact of the protein in the suggested range of neurodevelopmental pathways.

As acknowledged by the authors, human OXR1 loss-of-function mutations have been described previously (late 2019, not 'very recently' - this should be removed) and this new family shares the core clinical presentation of ataxia and seizures, with early-onset ataxia also shown in the OXR1 knockout mouse. Therefore the Figure 2 characterization of the human lymphoblasts is a useful addition to previous work, although generally in-line with what has been described previously in mammalian and fly cell lines and models, including rescue with shorter isoforms that is consistent with several other studies, from yeast and flies to mouse.

Figure 2e stats as shown are confusing with comparative 2-way lines - was multiple testing correction included here? where is the OXR1 signal coming from in treated cells? where are the primers in relation to the patient mutation? Is Nrf2 really significantly altered between dosed cell lines?

In Figure 3, the PCA does show trends of clustering between the technical replicates, however one major concern is not only the variation between samples of one type, but also the very large number of DE genes even at the initial iPSC level - almost 5000 (Figure S3M) - this likely represents 30-40% of all genes detectable in the cultures. Given this is the source of the later cell lines, how can more detailed assessments of pathways and cell trajectories be tracked in a biologically meaningful way? Certainly loss of OXR1 has played a part in these gene lists, but if so many fundamental pathways are disrupted, how can the authors be confident regarding specificity of the effects downstream? Comparisons between a small number of genes '.... which correlated to the formation of a thinner corpus callosum' (in humans) seems a long way off and not realistic.

For experimental variation, example graphical representations show wide ranges of DE in many cases; it is accepted that the statistical analysis should not surpass the 'fold-change', however sample c2 Ex18 (KO) (Figure S3f, q, r, s) appears to have quite a alternative signature to the two other Ex18 samples. Some comment is needed here, in particular considering the n=1 starting material.

The work assessing PRDM interactions and downstream responses is more solid; this interaction has been demonstrated by the authors previously, therefore it is sensible to focus on this aspect. In this current study, it is concluded that 'OXR1 may mainly affect the catalytic activity of PRMT-induced epigenetic histone modifications during neurogenesis' based on these data. With (almost) no changes at the iPSC stage - what is the explanation for the large number of DE if not via this pathway?

The overlap with ChIP-seq was an interesting finding (line 230+); can some statistical analysis should be added to these findings.

It is appreciated that work to generate a range of organoid iPSC-derived lines is a considerable task and there is some justification to compare the differentiation routes with respect to the human phenotype, furthermore, the anatomical quantification is not straightforward. In this case, even very early stages showed observable differences in morphology and size, therefore later changes in developmental trajectories are not unexpected. As such, the pathological data in the cultures is expected. What is less convincing is the histone methylation quantification; a second Ex18 exemplar should be shown for 6e, f and j. 6e distribution appears to reflect the graph, but in f and j how the quantification relates to the graph is not clear. An annotated example is needed including the bins etc. as a main figure.


Discussion:

Overall there is a lack of critique of the approaches used and the context of the work in the field.
i) How have others utilized organoid modelling as a tool for this type of histone modification?
ii) How do the organoid cultures compare to known human OXR1 'KO' and mouse KO neuropathology?
iii) Consideration of cell types not represented in the cultures?
iv) Consideration of other TLDc proteins and neurodevelopment e.g. TBC1D24 acute silencing in vivo (PMID 30858606; 24469796)
v) How are the data (statistically) compared to the author's previous work in KD cells? A few genes are picked out (Figure 2), but is there any
correlation with the pathways between studies?

Additional comments:

The datasets are based on iPSCs from a single patient and a single control - can the authors justify this n=1 selection and the source/choice of the control?

The conclusion sentence:

'....highlighting the therapeutic potential of OXR1 in numerous neurodegenerative and neurodevelopmental disorders.' the data presented does not support this statement.

The data showing overlap with neurological disease pathways is not at all convincing

**Reviewer 2**

Lin et al. demonstrate a thorough analysis of the role of the gene OXR1, the loss of which induces severe defects to stem cell differentiation in the neural system. They further show that the interaction of OXR1 and PRMT, dramatically affecting Histone 3 methylation. Additionally, they elegantly demonstrate in multiple cell types, including neurons and brain region-specific organoid models, that aberrant OXR1 induces severe deformities in development. The work will be of general interest to those who study neurogenesis as well as the use of stem cell models, broadly. It also characterizes a novel component of histone methylation, which is of broad interest.
Though this study is very thorough in the breadth of cell-based models generated, there are a number of major and minor issues that must be addressed before acceptance can be recommended. Many of these issues can be resolved with better explanations of the results, but some require thorough re-analysis of the data presented in order to determine their significance.

Major issues:
- Line 127: "OXR1 mRNA levels were similar in control and patient cells" Where are these data shown?
- Line 142: It is unclear how mtDNA rate is measured. Brief description would help.
- Line 150: "OXR1 deficiency reduces cell proliferation…" This is not what the data that this text is describing show, correct? These data show an increase in apoptosis, but no change in cell proliferation is measured.
- Figure 2h-i: It is difficult to assess the relative rescue of the OXR1 vector without healthy controls. It is clear that the vector improves phenotypes, but there is no context for the degree of these benefits.
- There are strong differences between the values in Figures 2b and 2i, despite some very tight error bars. For example, cell viability numbers for OXR1 mutant with 0.25 mM H2O2 treatment is 60% in 2b but less than 20% in 2i. Similarly, the necrosis values for OXR1 mutant in 2b are 20% for 0.25 mM H2O2 treatment but over 60% in 2i. Are these the same treatments? If so, why are the differences in results so dramatic, yet the data suggest low variance between replicates?
-Figure 3c: Some stats say p < 0.000, which is not possible.
- I assume that the numbers to the right of each bar in Figure 3d and S3o represent fold change, but this is not clear.
- Figure S3t: While the organization and characterization of each module is appreciated, the nodes are too small to interpret the data.
- In Figure 4b, were NSC-controls only measured with a single replicate? Why are there no error bars?
- Fig. 4c: How is partially truncated TLDc able to still simulate methylation? It also appears that not adding any OXR1 to the 1:4 PRMT5/MEP50 mix induces the most methylation. How can this be reasoned?
- While qualitatively convincing, very few of the images in Figures 5 and 6 are quantified, making it impossible to assess statistical significance.
- Some explanation of the images in Figures 5 and 6 is sparse. For example, it is not clear if there is any difference in the midbrain organoids in Figure 6b, and there is no explanation of this in the text. Better explanations of the findings will drastically help the reader understand the significance of the result.
- Were all of the factors shown to be altered in the OXR1 organoids also altered in the RNA-seq datasets? Though it is broadly shown that PRMTs-OXR1 influence neural development, the quality of this manuscript would be vastly improved if this was tied together better with the organoid development in the final two figures. As it stands, the readers must assume that this is the mechanism affecting development or are left with this being unclear.
- H3R2me2a or H3R2me2s are specifically mentioned to be unaltered in the text of Figure 6. Does this not directly undermine previous results which demonstrate that those are decreased in iPSCs?

Minor issues:
- Line 76: "Overexpression of Oxr1… shown to delay onset of…" is awkwardly worded.
- Line 90: "No human disease models are available to explore the pathological impact of OXR1 deficiency" but Wang et al. 2019 used human fibroblasts, so this line does not seem to be true.
- Line 121: "OXR1A and OXR1D are expressed in human brain, whereas most other cell types express isoform B and D" Is this suggesting that D is expressed everywhere, A is only in the brain, and B is everywhere expect the brain? This is a little unclear. Also, where is C expressed? No experiments are necessary for this, but this is unclear in the text.
- Figure 2e: Significance indicators are a bit confusing. Does the bracket mean that significance between the listed bars are all two stars? Also, some of the bars are a light gray color and are difficult to see.
- The evidence that neural differentiation is impacted (Fig. S3i, k) should be better discussed in the text. This is a key point in the upcoming parts of this manuscript but is barely mentioned in passing here in line 172.
- Line 179: What is the rationale for looking at OXR1-dependent regulation of transcriptional networks? This should be better explained.
- Fig. 3e-f: For consistency, could the control and OXR1 mutant samples remain on the same side for both of these panels? As it currently stands, 3e has control on the left and 3f has control on the right.
- Fig. 5a: Please add a little space between the points in the graph to make it easier to distinguish individual points. Perhaps making the circles "control" smaller would be sufficient.
- The placement of images in 5g-h is a bit disorderly and makes it difficult to identify which conditions are being shown. Particularly in 5g, it was difficult to even realize that the images on the right for Days 59-65 were magnifications of the images to the left because the images are touching. And is there a label missing from the image to the far right of Day 50 in 5g?
- In many cases 2 OXR1 and 2 control lines are shown, but in others its only 1 line. Did the results shown only work in one of the lines in these cases?
- Why was H4R3me2s looked at in the cortical organoids? Is this a particular marker for organoid development, or was this looked at for another reason? It does tie things to the previous results in Figure 4, but it's not clear if that was the original intent.
- IP methods should be written in the past tense, but in most of the paragraph they are not.

**Authors’ response to reviewers**

Reviewer reports:

Reviewer #1: Here the authors combine the description of a novel set of patients harboring a recessive loss-of-function mutation in the gene OXR1, followed by a set of cellular experiments to define specific properties of gene 'knockout' in human cells including organoids differentiated from patient iPSCs alongside quantification of histone arginine methylation. The hypothesis is that OXR1 is responsible for maintaining these epigenetic marks via an interaction with protein arginine methyltransferases (PRMTs) and loss of OXR1 leads to disruption of neurodevelopment via this axis. In summary, the data certainly extends previous work and supports previous studies from the submitting authors regarding a role in arginine methylation, yet the considerable variation and quantification methods in the more complex cellular models makes it difficult to define the specific impact of the protein in the suggested range of neurodevelopmental pathways.

Authors: We are very grateful to the reviewer for the critical and insightful evaluation of our manuscript. We agree with the reviewer that it is challenging to define the specific impact of OXR1 on the numerous neurodevelopmental pathways affected. However, the complex cellular models provide new insight addressing important functions of OXR1 in regulating neuronal development, including spatial-temporal regulation of histone arginine methylation.

As acknowledged by the authors, human OXR1 loss-of-function mutations have been described previously (late 2019, not 'very recently' - this should be removed) and this new family shares the core clinical presentation of ataxia and seizures, with early-onset ataxia also shown in the OXR1 knockout mouse. Therefore the Figure 2 characterization of the human lymphoblasts is a useful addition to previous work, although generally in-line with what has been described previously in mammalian and fly cell lines and models, including rescue with shorter isoforms that is consistent with several other studies, from yeast and flies to mouse.

Authors: We replaced ‘very recently’ with ‘in 2019’ in the text. Wang et al. (2019) showed that OXR1-deficient human fibroblasts had similar sensitivity as control cells to oxidative stress (upon H2O2 treatment). We got similar results with our patient’s fibroblast cells. We think that the fibroblast is not sensitive enough to explore the role of OXR1 in antioxidation. Lymphoblast cells harness a higher OXR1 level and are more sensitive to H2O2 treatment. This study shows that OXR1 deficiency compromises cell growth, survival, and response to oxidative stress in patients-derived lymphoblasts. This is, as far as we know, the first human model with minimal artificial intervention showing defects due to OXR1 deficiency.

Figure 2e stats as shown are confusing with comparative 2-way lines - was multiple testing correction included here? where is the OXR1 signal coming from in treated cells? where are the primers in relation to the patient mutation? Is Nrf2 really significantly altered between dosed cell lines?

Authors: We highly appreciated the reviewer´s comments on this issue. We carefully checked all the raw data for Fig. 2e and 2f. As the reviewer suggested, we performed multiple T-tests and used the Holm-Sidak method to correct multiple comparisons when determining the statistical significance. Fig. 2e-f as well as the figure legends have been updated. Due to the size limitation and aesthetic considerations, some genes from Fig.2e are moved to Fig. S2d, together with two new genes, PTGS1(functions as a peroxidase) and CYBG (involved in antioxidation to reduce ROS, such as H2O2). The mRNA level of Nrf2 was not significantly altered between CTRL and patient lymphoblasts. We accidentally pasted the SD values of DUSP1 to NRF2, which is corrected in the updated figure in the revised manuscript.

As for the primers in relation to the patient mutation, one primer pair is specific to the patient’s exon-skipping sequence, and the other primer pair targets OXR1 isoform B at the N terminus. Increased transcripts were detected in the treated patient cells when compared to the CTRL cells using both primer pairs (updated data in Fig. S1c).

In Figure 3, the PCA does show trends of clustering between the technical replicates, however one major concern is not only the variation between samples of one type, but also the very large number of DE genes even at the initial iPSC level - almost 5000 (Figure S3M) - this likely represents 30-40% of all genes detectable in the cultures. Given this is the source of the later cell lines, how can more detailed assessments of pathways and cell trajectories be tracked in a biologically meaningful way? Certainly loss of OXR1 has played a part in these gene lists, but if so many fundamental pathways are disrupted, how can the authors be confident regarding specificity of the effects downstream? Comparisons between a small number of genes '.... which correlated to the formation of a thinner corpus callosum' (in humans) seems a long way off and not realistic.

Authors: We conducted RNAseq with three individual clones of each genotype at three stages of neuronal differentiation: iPSC (iPS_CTRL and iPS_OXR1ΔEx18), neural aggregate (NA) (NA_ CTRL and NA_ OXR1 ΔEx18), and neuron (Neu) (Neu_CTRL and Neu_OXR1ΔEx18). These are biological triplicates, not technical replicates. Some variations among biological replicates are expected. The very large number of DEGs at the initial iPSC level is due to a low log2FC threshold (Log2FC>ǀ0.5ǀ). In the revised manuscript, we introduced a new analysis for the Log2FC distribution of DEGs (Padj <0.05) at each differentiation stage (updated in Fig. 3c, the violin plot). Significantly, the median Log2FC of the DEG population showed a steep increase from the iPSC stage (median Log2FC = 0.8276, 95% CI 1.119-1.158, p<0,0001, Wilcoxon Rank Test), to the NA stage (median Log2FC = 1.069, 95% CI 1.541-1.607, p<0,0001), and to the neuron stage (median Log2FC = 1.342, 95% CI 1.735-1.792, p<0,0001).

A total of 29568 genes were sequenced by RNAseq. In the revised manuscript, we compared the number of DEGs using three different log2FC thresholds, Log2FC>ǀ0.5ǀ, Log2FC>ǀ1ǀ or Log2FC>ǀ1.5ǀ (Padj <0.05), updated in Fig. S3m. When using the Log2FC>ǀ0.5ǀ, the number of DEGs is 4723 at the initial iPSC stage (representing 16% of total genes), 3224 at the stage of neural aggregate (representing 10.9% of total genes), and 6758 at the neuronal stage (representing 22.9% of total genes). When using the Log2FC>ǀ1ǀ, the number of DEGs is reduced to 2226 at the initial iPSC stage (representing 7.5% of total genes), to 2408 at the stage of neural aggregate (representing 8.1% of total genes), and to 5385 at the neuronal stage (representing 18.2% of total genes). When using the Log2FC>ǀ1.5ǀ, the number of DEGs is reduced to 1089 at the initial iPSC stage (representing 3.5% of total genes), to 1680 at the stage of neural aggregate (representing 5.7% of total genes), and to 3793 at the neuronal stage (representing 12.8% of total genes). Significantly, more DEGs with a Log2FC>ǀ1.5ǀ were identified at the neuron stage.

As the selection of DEGs based on the Log2FC is very arbitrary, we performed GSEA analysis using the normalized read counts of all genes from the biological triplicates of neuronal samples. Consistently, Gene Ontology by GSEA revealed an underrepresentation of biological processes required for nervous system development. The top-ranked DEGs at the neuronal stage were involved in axon guidance and migration (Fig.3f.), including the dysregulation of Reelin, NRCAM, Class-3 semaphorin, and Slit/Robo pathways. These core genes have been reported to be highly correlated to the formation of a thinner corpus callosum (see Ref 38-40).

For experimental variation, example graphical representations show wide ranges of DE in many cases; it is accepted that the statistical analysis should not surpass the 'fold-change', however sample c2 Ex18 (KO) (Figure S3f, q, r, s) appears to have quite a alternative signature to the two other Ex18 samples. Some comment is needed here, in particular considering the n=1 starting material.
Authors: As mentioned above, we used biological triplicated samples, which is more desirable than technical replicates. Some variations among biological triplicates are normal, thus, a "perfect" clustering is not expected.

The work assessing PRDM interactions and downstream responses is more solid; this interaction has been demonstrated by the authors previously, therefore it is sensible to focus on this aspect. In this current study, it is concluded that 'OXR1 may mainly affect the catalytic activity of PRMT-induced epigenetic histone modifications during neurogenesis' based on these data. With (almost) no changes at the iPSC stage - what is the explanation for the large number of DE if not via this pathway?

Authors: As mentioned above, we introduced a new analysis for the Log2FC distribution of DEGs (Padj <0.05) at each differentiation stage (updated in Fig. 3c, the violin plot). Significantly, the median Log2FC of the DEG population showed a steep increase from the iPSC stage (median Log2FC = 0.8276, 95% CI 1.119-1.158, p<0,0001), to the NA stage (median Log2FC = 1.069, 95% CI 1.541-1.607, p<0,0001), and to the neuron stage (median Log2FC = 1.342, 95% CI 1.735-1.792, p<0,0001). When using the Log2FC>ǀ1.5ǀ, 1089 DEGs were identified at the initial iPSC stage, representing 3.5% of total genes, which is almost 3.5-fold less than DEGs identified at the neuron stage (3793 DEGs, representing 12.8% of total genes). In addition, the core pluripotency genes, e.g., MYC, SOX2, and NANOG, did not show differential gene expression in OXR1-deficient iPSCs. These results demonstrate that the impact of OXR1 on gene regulation is more pronounced at the neuron stage than at the iPSC stage. This is consistent with our finding that the significant changes in epigenetic histone modifications are identified at the neuron but not the iPSC stage (Fig. 4b). However, the 1089 DEGs (Log2FC>ǀ1.5ǀ) identified at the iPSC stage (in the updated Fig. S3m) may indicate other additional unknown functions of OXR1 that merit further investigations.

The overlap with ChIP-seq was an interesting finding (line 230+); can some statistical analysis should be added to these findings.

Authors: We appreciate that the overlap analysis with ChIP-seq is recognized. We now include the statistical information in Fig. 4c, updated in the revised manuscript.

It is appreciated that work to generate a range of organoid iPSC-derived lines is a considerable task and there is some justification to compare the differentiation routes with respect to the human phenotype, furthermore, the anatomical quantification is not straightforward. In this case, even very early stages showed observable differences in morphology and size, therefore later changes in developmental trajectories are not unexpected. As such, the pathological data in the cultures is expected. What is less convincing is the histone methylation quantification; a second Ex18 exemplar should be shown for 6e, f and j. 6e distribution appears to reflect the graph, but in f and j how the quantification relates to the graph is not clear. An annotated example is needed including the bins etc. as a main figure.

Authors: As the cortical development of OXR1∆Ex18 c2 is arrested in the very early stage (Fig. 6a), the quantification of histone methylations was mainly analyzed in OXR1∆Ex18 c1 samples. We now included data from the newly generated OXR1∆Ex18 c3 (Fig. S6a) and Fig. 6c, d, e, and f are updated. Briefly, the distribution pattern of histone modification in OXR1∆Ex18 c3 is consistent with the data from OXR1∆Ex18 c1, using the same quantification method. By combining analysis from two clones, increased statistical significance is achieved. An annotated example showing the selection of quantification bins (yellow dash line) is added to Fig. 6e.

Discussion:

Overall there is a lack of critique of the approaches used and the context of the work in the field.

i) How have others utilized organoid modelling as a tool for this type of histone modification?

Authors: Recent studies have demonstrated that brain organoids possess comparable transcriptomic profiles to certain stages of fetal brains, as well as similar regulation of epigenetic programs. For example, the transcriptome and ChIPseq of H3K4me3 and H3K27ac of cortical organoids at around day 50 mapped most consistently to isogenic cortical brain tissues at PCW16 (see Ref 83-84). We have added this information to the discussion.

ii) How do the organoid cultures compare to known human OXR1 'KO' and mouse KO neuropathology?

Authors: OXR1 is highly expressed in the brain, higher than any other types of tissues such as skin (fibroblast cells). The main pathological symptoms in OXR1 deficient patients are neurological-related. Organoids allow us to identify pathogenic features arising due to the OXR1 deficiency in brain-like tissues. Brain organoids are the only available human brain models when it is not possible to access the brain tissues of OXR1 patients all over the world. Cortical organoids at around day 50 coincide with the post-conception week (PCW) 14-16 in humans (see Ref. 75). Some developmental processes are human-specific. For instance, the second wave of neurogenesis from oRGCs located in the outer SVZ is believed to be responsible for the massive neocortex expansion in cortical size and neuron abundance, affecting the acquisition of higher cognitive functions in humans. We found that OXR1 deficiency impaired radial glial cells including oRGC proliferation in brain organoids. Although less uniform than monolayer neural culture, brain organoids exhibit more complex cytoarchitecture over time including the organization of progenitor cells and sequential generation lamination of neurons.

Both OXR1 deficient patients (Ref. 4) and OXR1 knockout mouse models (Ref. 5,12) show cerebella-related symptoms after birth. We found up-regulation of EN2, which is required for the organization of the midbrain/hindbrain boundary and cerebellar morphogenesis, was not detected in OXR1ΔEx18 neurons or the early stage of OXR1ΔEx18 midbrain organoids. We tried the protocol of cerebellum organoids described by the Sasai group. Unfortunately, those organoids dissociated at an early stage, which limited our study of OXR1´s roles in the cerebellar progenitor cell development and the maturation processes of cerebellar neuron subtypes. Further refinement of patterning methods may be needed to improve the survival, and maturation, as well as other regions in the hindbrain, ie. the pons.

In the revised manuscript, we have added most of this information to the discussion.

iii) Consideration of cell types not represented in the cultures?

Authors: OXR1 is highly expressed in neuronal cell types and is also detected in OPCs and microglia cells across human adult cortex, cited from http://celltypes.brain-map.org/rnaseq). Although a few glial cells (e.g., astrocytes and oligodendrocytes) are detected in long-term cultured brain organoids, their applicability in disease modelling is limited due to infertility, impaired cellular complexity, and immaturity. Efficient neural induction, especially the application of region-specific patterning factors restricts the generation of non-neuroectodermal cells, such as microglial, endothelial, and meningeal cells that are critical to the development and function of the human brain.

Recently, there are significant advancements and attempts to improve organoid longevity and maturity, enhance glial cell production, reconstitute microglial cells and vasculatures, and fuse multiple region-specific organoids. We believed future improvements will allow organoids to faithfully recapitulate late gestational and early post-natal development, and better model diseases.

iv) Consideration of other TLDc proteins and neurodevelopment e.g. TBC1D24 acute silencing in vivo (PMID 30858606; 24469796)

Authors: Most pathogenic mutations of TBC1D24 are in the TBC domain, not the TLDc domain. Some of them cause a loss-of-function protein means that the whole protein is defective, like the mutations addressed in PMID 30858606 (Ref. 68). Although mutations in the TLDc domain of TBC1D24 can cause DOORS syndrome, epileptic encephalopathy, progressive myoclonus, and progressive postlingual dominant hearing loss (Ref. 66-67), the genotype-phenotype relationships remain largely unknown.

Depletion of TBC1D24 by the acute silencing or mutation leads to defects in axon formation and maturation, and neuronal radial migration (See Ref. 68-69), consistent with our finding that the OXR1 deficiency impaired axon development (Fig.3g, S3j, 5b, c). Interestingly, acute silencing of TBC1D24 in vivo showed reduced corpus callosum projections in rats (Ref. 68), which was one of MRI signs identified in our OXR1 deficiency patient.

In the revised manuscript, we have added this information to the discussion.

v) How are the data (statistically) compared to the author's previous work in KD cells? A few genes are picked out (Figure 2), but is there any correlation with the pathways between studies?

Authors: The patient’s LB cells are more sensitive to H2O2-induced oxidative stress than KD cells. The identified pathways, like P53, p21-NRF2, in the previous work were also affected in LB cells. The differences were detected in patient LBs under the regular culture conditions when compared to LBs derived from her healthy sibling.

Additional comments:

The datasets are based on iPSCs from a single patient and a single control - can the authors justify this n=1 selection and the source/choice of the control?

Authors: The OXR1 deficiency unfortunately is a very rare disorder. We appreciated that patient II-2 donated her skin tissue and allowed us to obtain the fibroblast cells and reprogramme them to iPSCs. The healthy control AG05836 is a gender-matched control fibroblast cell line and has been well-established as a control line in our lab (Ref. PMID: 33264635). We selected at least three qualified clones of each genotype as biological controls in downstream experiments, that is sufficient, we believe.

The conclusion sentence:
'....highlighting the therapeutic potential of OXR1 in numerous neurodegenerative and neurodevelopmental disorders.' the data presented does not support this statement. The data showing overlap with neurological disease pathways is not at all convincing

Authors: DEGs in patient-derived OXR1ΔEx18 neurons overlapped 39%, 41%, and 31% with disease-associated genes or high-confident risk genes of cerebellar atrophy, autism spectrum disorder, and schizophrenia in published databases (Table 2 in the revision), suggesting a role of OXR1 in the protection against a broader spectrum of neurodevelopmental disorders. This result was shown in Fig. S7 of the first submission but only mentioned in the discussion. In the revised manuscript, this information is shown as a new Table 2 and described in both the result and discussion.

Recent studies support the role of OXR1 in protection against different pathological conditions in the central nervous system (CNS), e.g., AD (Ref. 61), PD (Ref. 20), cerebral ischemia (Ref. 62), ALS (Ref. 17, 19), retinal degeneration (Ref. 10, 30), and traumatic spinal cord injury (Ref. 63). Restoring OXR1 using AAVs, targeted miRNAs, or plasmid-containing liposome-based nanoparticles are promising therapeutic strategies (Ref. 3, 17, 19, 20, 31, 62). Therefore, OXR1 is a potential molecular target in the clinical diagnosis and therapy of a broader spectrum of neurological disorders. We have added this information to the discussion.

Reviewer #2: Lin et al. demonstrate a thorough analysis of the role of the gene OXR1, the loss of which induces severe defects to stem cell differentiation in the neural system. They further show that the interaction of OXR1 and PRMT, dramatically affecting Histone 3 methylation. Additionally, they elegantly demonstrate in multiple cell types, including neurons and brain region-specific organoid models, that aberrant OXR1 induces severe deformities in development. The work will be of general interest to those who study neurogenesis as well as the use of stem cell models, broadly. It also characterizes a novel component of histone methylation, which is of broad interest.

Authors: We highly appreciated the reviewer‘s positive assessment of our study.

Though this study is very thorough in the breadth of cell-based models generated, there are a number of major and minor issues that must be addressed before acceptance can be recommended. Many of these issues can be resolved with better explanations of the results, but some require thorough re-analysis of the data presented in order to determine their significance.

Major issues:
- Line 127: "OXR1 mRNA levels were similar in control and patient cells" Where are these data shown?

Authors: This sentence is indeed not precise. The OXR1 transcripts that were specific to the patients’ exon skipping sequence were largely reduced (Fig. S1c). But the specific OXR1B transcripts were increased in patient LBs when compared to the CTRL LBs derived from her healthy sibling (Fig. S1c). The corrected information is updated in the revised manuscript. A new Fig. S1c is included in the additional file.

- Line 142: It is unclear how mtDNA rate is measured. Brief description would help.

Authors: Brief description is included in the Method part – mtDNA mutation frequency measurement.

- Line 150: "OXR1 deficiency reduces cell proliferation…" This is not what the data that this text is describing show, correct? These data show an increase in apoptosis, but no change in cell proliferation is measured.

Authors: Changes in cell proliferation were measured. As shown in Fig. 2a, Reduced cell proliferation was detected in LB-OXR1ΔEx18.

- Figure 2h-i: It is difficult to assess the relative rescue of the OXR1 vector without healthy controls. It is clear that the vector improves phenotypes, but there is no context for the degree of these benefits.

Authors: We do not have data for healthy controls in the experiments. However, the relative differences between cells with OXR1 and vector alone resemble the differences we found between CTRL cells and OXR1 mutant in Figure 2a-b.

- There are strong differences between the values in Figures 2b and 2i, despite some very tight error bars. For example, cell viability numbers for OXR1 mutant with 0.25 mM H2O2 treatment is 60% in 2b but less than 20% in 2i. Similarly, the necrosis values for OXR1 mutant in 2b are 20% for 0.25 mM H2O2 treatment but over 60% in 2i. Are these the same treatments? If so, why are the differences in results so dramatic, yet the data suggest low variance between replicates?

Authors: We have used different batches of growth media and H2O2 in Figures 2b and 2i, which most likely have contributed to the relatively large differences between the two experiments. Importantly, replicates (including all replicates) were examined simultaneously to eliminate batch effects within each individual experiment (Figure 2b and 2i).

-Figure 3c: Some stats say p < 0.000, which is not possible.

Authors: p < 0.001 is now updated in Figure 3c.

- I assume that the numbers to the right of each bar in Figure 3d and S3o represent fold change, but this is not clear.

Authors: The normalized enrichment score (NES) and false discovery rate (FDR) values were indicated next to each bar in Fig. 3d and S3o. All p values are less than 0.001. This information has been clarified in the figure as well as figure legends.

- Figure S3t: While the organization and characterization of each module is appreciated, the nodes are too small to interpret the data.

Authors: The high-resolution figure is now provided.

- In Figure 4b, were NSC-controls only measured with a single replicate? Why are there no error bars?

Authors: The NSC controls in Figure 4b were only measured with a single replicate. This is partially due to the consumption of most NSC control samples in the preliminary tests. However, as we have seen, from the preliminary experiments, the NSC controls were very stable. We are confident that the single replicate of the NSC control is still reliable and representative.

- Fig. 4c: How is partially truncated TLDc able to still simulate methylation? It also appears that not adding any OXR1 to the 1:4 PRMT5/MEP50 mix induces the most methylation. How can this be reasoned?

Authors: Based on the TLDc truncations, we suggest that the entire domain is not required to stimulate PRMT5/MEP50. How the TLDc domain interacts with PRMT5/MEP50 merits further investigations by structural data (e.g., protein crystallography). Our data clearly show that OXR1 stimulates PRMT5/MEP50 methylation 2-3 fold. In the condition with the highest methylation, we used a 4-fold higher PRMT5/MEP50 concentration as compared to the stimulation experiment.

- While qualitatively convincing, very few of the images in Figures 5 and 6 are quantified, making it impossible to assess statistical significance.

Authors: We included data from the newly generated OXR1∆Ex18 c3 (Fig. S6a). The total number of organoids involved in quantification was increased and more images were analyzed. Increased statistical significance is achieved. Fig. 6c-f as well as the related figure legends are updated.

- Some explanation of the images in Figures 5 and 6 is sparse. For example, it is not clear if there is any difference in the midbrain organoids in Figure 6b, and there is no explanation of this in the text. Better explanations of the findings will drastically help the reader understand the significance of the result.

Authors: We tried our best to provide main descriptions for the individual results in Figures 5 and 6. The morphological phenotypes of different brain organoids generated from CTRL and OXR1-deficient clones were shown in Fig. 5e-f and Fig. 6a-b. As for the midbrain organoids in Fig. 6b, we did not observe morphological differences between CTRL and OXR1-deficient clones. The characterization of individual organoid samples was achieved by immunohistochemistry using distinct molecular markers. The OXR1∆Ex18 c2 is often arrested in the very early organoid stage (Day 4-5). Therefore, the characterization of different organoids was mainly assessed in OXR1∆Ex18 c1 samples. In the revised manuscript, we have included data from the newly generated OXR1∆Ex18 c3 (Fig. S6a) and updated Fig. 6c, d, e, and f.

- Were all of the factors shown to be altered in the OXR1 organoids also altered in the RNA-seq datasets? Though it is broadly shown that PRMTs-OXR1 influence neural development, the quality of this manuscript would be vastly improved if this was tied together better with the organoid development in the final two figures. As it stands, the readers must assume that this is the mechanism affecting development or are left with this being unclear.

Authors: We highly appreciated the reviewer´s comments on this issue. Most altered factors in the OXR1-deficient organoids showed a similar trend of differential expression in OXR1-deficient neurons. For example, down-regulation of MAP2 (Log2FC=-3.00) and RELN (Log2FC=-5.36) was identified in OXR1ΔEx18 neurons (Padj<0.05) as detected in OXR1ΔEx18 cerebral organoids (Fig. 5b,c). Reduced expression of TBR1 was observed in OXR1ΔEx18 cerebral organoids (Fig. 5b), but the expression of TBR1 was barely detected in the 2D neural differentiation model. Further, the expression level of FOXA2 (Log2FC=-7.32) and EN2 (Log2FC=-7.80) was strikingly decreased in OXR1ΔEx18 neurons, which is consistent with the sparse detection of FOXA2-positive progenitors and failed induction of EN2 in OXR1ΔEx18 midbrain organoids (Fig. 7b, 7d). Although the level of tyrosine hydroxylase (TH) showed a significant decrease (Log2FC=-2.83) in OXR1ΔEx18 neurons (the 2D neural differentiation model), the guided OXR1ΔEx18 midbrain dopaminergic (mDA) cells showed an abnormal increase of TH at day 50. Of note, the 2D neural differentiation model and the guided 3D brain organoid model represent distinct processes of neurodevelopment. Our work highlights the important role of OXR1 in shaping histone arginine methylations and transcription during early neurodevelopment. In the revised manuscript, this point is clarified in the Discussion.

- H3R2me2a or H3R2me2s are specifically mentioned to be unaltered in the text of Figure 6. Does this not directly undermine previous results which demonstrate that those are decreased in iPSCs?

Authors: The expression levels of H3R2me2a and H3R2me2s were significantly reduced in the OXR1-deficient cortex (Fig. 6d-e and 7e) as well as in OXR-deficient NSCs (Fig. 4b). The difference in iPSCs is not significant (Fig. 4b).

Minor issues:
- Line 76: "Overexpression of Oxr1… shown to delay onset of…" is awkwardly worded.

Authors: The sentence is corrected to ‘Overexpression of Oxr1 in variant ALS mouse models delayed the onset of neuromuscular phenotypes and neuroinflammatory responses and improved motor neuron functions.’

- Line 90: "No human disease models are available to explore the pathological impact of OXR1 deficiency" but Wang et al. 2019 used human fibroblasts, so this line does not seem to be true.

Authors: The patient fibroblasts were generated to test the OXR1 function in oxidative stress [4]. However, no human disease models are available to explore the pathological impact of OXR1 deficiency in the brain. We have improved the sentence in the revised manuscript.

- Line 121: "OXR1A and OXR1D are expressed in human brain, whereas most other cell types express isoform B and D" Is this suggesting that D is expressed everywhere, A is only in the brain, and B is everywhere expect the brain? This is a little unclear. Also, where is C expressed? No experiments are necessary for this, but this is unclear in the text.

Authors: The shortest isoform OXR1D is expressed ubiquitously, including the brain, skin (fibroblast cells), lymphoblast cells, heart, muscles, etc. OXR1A is expressed exclusively in the human brain. Isoform C is expressed only in testis. Isoform B is expressed in other types of tissue, like the heart and liver, mutually exclusive with OXR1A. Detailed information can be found in our previous paper, see Ref. 14.

- Figure 2e: Significance indicators are a bit confusing. Does the bracket mean that significance between the listed bars are all two stars? Also, some of the bars are a light gray color and are difficult to see.

Authors: In the revised manuscript, Fig.2e including the signs of significance has been completely updated. We performed multiple T-tests and used the Holm-Sidak method to correct multiple comparisons when determining the statistical significance. Due to the size limitation and aesthetic considerations, some genes from Fig.2e are moved to Fig.S2d, together with two new genes, PTGS1(functions as a peroxidase) and CYBG (involved in antioxidation to reduce ROS, such as H2O2).

- The evidence that neural differentiation is impacted (Fig. S3i, k) should be better discussed in the text. This is a key point in the upcoming parts of this manuscript but is barely mentioned in passing here in line 172.

Authors: By using the 2D neural differentiation model, we show that (i) further differentiation of iPSCs into neurons revealed irregularities in the morphology of OXR1ΔEx18 neural aggregates (NAs) (Fig. S3g,h); (ii) OXR1-depletion reduced neurite growth (Fig. 3a), as well as the number of neurons, concomitant with reduced expression of neuronal markers, e.g., microtubule-associated protein 2 (MAP2) and class III beta-tubulin (Tuj1) (Fig. S3i, j); (iii) OXR1 mRNA and protein levels increased during neural differentiation in control cells (Fig. S3l); (iv) Genes essential for neurogenesis and neurodifferentiation as well as axon guidance and cell migration were significantly down-regulated in Neu- OXR1ΔEx18 (Fig. 3f-g). These results suggest an important role of OXR1 in neural differentiation, which is now well described in the result part and further discussed in the discussion part of the revised manuscript.

- Line 179: What is the rationale for looking at the OXR1-dependent regulation of transcriptional networks? This should be better explained.

Authors: Our previous work revealed a role of OXR1 in transcriptional regulation in the rodent pituitary gland, including growth hormone expression and signal transduction (Ref. 1). In the hydrogen peroxide-exposed HeLa cells, it regulated the transcriptional networks required to detoxify reactive oxygen species and modulate cell cycle and apoptosis via p53 and its downstream pathways (Ref.15). Thus, in this study, we explored the impact of OXR1-dependent transcriptional regulation on the neural differentiation process.

- Fig. 3e-f: For consistency, could the control and OXR1 mutant samples remain on the same side for both of these panels? As it currently stands, 3e has control on the left and 3f has control on the right.

Authors: Corrected.

- Fig. 5a: Please add a little space between the points in the graph to make it easier to distinguish individual points. Perhaps making the circles "control" smaller would be sufficient.

Authors: Corrected

- The placement of images in 5g-h is a bit disorderly and makes it difficult to identify which conditions are being shown. Particularly in 5g, it was difficult to even realize that the images on the right for Days 59-65 were magnifications of the images to the left because the images are touching. And is there a label missing from the image to the far right of Day 50 in 5g?

Authors: Corrected

- In many cases 2 OXR1 and 2 control lines are shown, but in others its only 1 line. Did the results shown only work in one of the lines in these cases?

Authors: The OXR1∆Ex18 c2 is often arrested in the very early organoid stage (Day 4-5). Therefore, in many cases, the analysis was mainly assessed in OXR1∆Ex18 c1 samples. In the revised manuscript, we have added data from the newly generated OXR1∆Ex18 c3 (Fig. S6a) and updated Fig. 6c, d, e, and f, accordingly.

- Why was H4R3me2s looked at in the cortical organoids? Is this a particular marker for organoid development, or was this looked at for another reason? It does tie things to the previous results in Figure 4, but it's not clear if that was the original intent.

Authors: It has been reported that H4R3me2s, as a repressor of gene transcription, is involved in regulating proliferative neuroepithelium/neural progenitors in mouse cortex development (Ref. 80). But no studies explore the role of H4R3me2s in the brain organoid or human brain development. Although the focus on arginine methylation become more and more popular, the role of arginine methylation in brain development remains elusive.

We examined histone modifications during the differentiation of iPSCs to neural stem cells (NSCs) using an antibody-based screening and revealed increased histone methylations on H3R2/R17, H4R3, H3K4/K9/K27 in the control NSCs (Fig. 4b). In contrast, OXR1-depleted NSCs showed significantly impaired histone arginine/lysine methylations, e.g., H4R3me2s/2a, H3R2me2a, H3R17me2s/2a, H3K4me3, H3K9me3, and H3K27me3. Strikingly, genes enriched with the repressive mark H4R3me2s overlapped with 71% of the down-regulated genes during neural differentiation in control cells, of which 63% of the genes were differentially expressed (up-regulated) in OXR1∆Ex18 cells (Fig. 4c). Further, the TLDc domain of OXR1 stimulated PRMT5-catalyzed H4R3 methylation in vitro (Fig. 4d), suggesting a direct role of OXR1 in regulating H4R3 methylation. Interestingly, we identified a specific distribution pattern of H4R3me2s in the cortical organoids, with low levels in VZ/SVZ like structures but gradually increasing in developing neurons outside the progenitor zone following the radial migration (Fig. 6d). This finding suggests a regulatory role of H4R3me2s during neurogenesis and the establishment of the cortical plate. In OXR1∆Ex18 cortical organoids, the patterned distribution of H4R3me2s was lost by showing an overall reduction H4R3me2s (Fig. 6d). Notably, the regulation of H4R3me2s in the midbrain development was distinct from the cortical brain by showing a high level in the VZ progenitor zone (Fig. 7e). OXR1 deficiency did not impair the distribution pattern of H4R3me2s in the midbrain (Fig. 7e). Our results suggest an essential role of H4R3me2s in the early stage of human cortical development and how OXR1 impacts the histone arginine methylation in the human brain developmental process, which is not known before. These results are clearly described and discussed in the revised manuscript.

- IP methods should be written in the past tense, but in most of the paragraph they are not.

Authors: The writing for IP methods is corrected in the revised manuscript.

**Second round of review**

**Reviewer 1**

In general, the manuscript has been improved. I still have a few comments regarding the conclusions and focus; the data tell a convincing story around
one very defined mechanism in the context of the iPSC-derived cells utilised, but the wider disease relevance or explanation for many of the human clinical features (e.g. epilepsy, ataxia) is ignored or
not convincing, in particular that only a single patient was used with a not well-matched control sample (and no isogenic control) and that they were not able to model cerebellar
structures, the cardinal pathological region of OXR1 loss-of-function.


>> Title

The name of the gene is incorrect (!) - it is 'Oxidation resistance 1' not 'Oxidation resistance gene 1'

The title also suggests the pathway has been studied in the human brain

I suggest:

A loss-of-function mutation in human Oxidation Resistance 1 disrupts the spatial-temporal regulation of histone arginine methylation in neurodevelopment


>> Abstract

'very recently' should be removed (it was corrected in the main text)

The statement

'This study provides new insights into pathological features and molecular underpinnings associated with OXR1
deficiency in patients, highlighting the therapeutic potential of OXR1 in numerous neurodegenerative
and neurodevelopmental disorders.'

I suggest that the manuscript 'provides potential new insights into pathological features and molecular underpinnings associated with OXR1
deficiency in patients'

but it is not

'highlighting the therapeutic potential of OXR1 in numerous neurodegenerative
and neurodevelopmental disorders.'

the second half of the sentence should be removed

(also in the Conclusion to the paper)


>> Table 2

I am still not convinced of the significance of the overlapping DEGs with other disorders from very different data sources warrants an addition to the paper; with this study finding many hundreds/thousands of
DEGs that are expressed in the CNS, the Table does not add any useful information. I disagree that the data presented provides insight into the role of OXR1 in other neurodevelopmental disorders.


>> Discussion page 17-18

The section on disease relevance is confusing, as the data on loss-of-function presented here are mixed with over-expression studies that follow a completely different set of
molecular mechanisms and hypotheses. In my opinion this section should be removed completely. Also the OXR1-D mutation in SLI is mentioned, but there is no discussion of the relevance to the current study.

>> Choice of control

I thank the authors for clarifying the control sample choice; it is becoming 'industry-standard' to provide isogenic controls when claiming the translational relevance of rare disease stem cell-derived
modelling. In my opinion a short section in the Discussion to address this is warranted. e.g. apart from sex, what variables related to ethnicity / age etc. might be influencing the study?

**Reviewer 2**

Lin et al. have done well to address many of this reviewers concerns, especially with regards to describing the rationale for their approaches and explaining the probes used in the later figures. In particular, the explanations added for Figures 5 and 6 are vastly improved and greatly appreciated. There are, however, a few major and minor concerns that remain to be addressed.
Major:
- Regarding Figure 4b: The authors mention that the NSC-CTRL group is from a single biological replicate, and also mention in their own rebuttal that these are preliminary results. While the difference between these controls and the other groups are striking, and though they do show that other phenotypes in these controls are stable, a single replicate for any experiment is highly unorthodox. It is also not possible to perform robust statistical analyses between sample groups with only a single replicate. As this is a key part of the paper, this experiment must be repeated with multiple replicates.
- In their response to my concerns about Figure 4d (formerly 4c), the authors reasoned that increased PRMT5/MEP50 concentration contributed to elevated methylation despite no OXR1 added. But in the text, the authors state that this experiment suggests that OXR1 is essential for methylation signatures. These results seem to suggest that it is not required as long as PRMT5 and MEP50 are upregulated. This needs to be addressed and reasoned in the manuscript.
- In the previous review, this reviewer had suggested that the images from Figure 5 should be quantified to better determine significance. This does not appear to have been addressed, as the authors’ response does not mention this point and the figures still lack quantification. Please quantify the signals that were being measured, particularly in Figures 5b-c and 5g-h. This is also true in some images in Figure 6, such as 6b. The images are convincing, but if the authors are claiming significant differences, quantification is necessary.

Minor:
- Figure 3 says “Figure 7” in the middle of it.
- In the previous draft, the organoid types were labeled in Figure 6 (e.g. “midbrain organoids” or “cortical organoids”). They do not appear in the most recent draft. Please put those labels back in to improve clarity in the figure.
- The point about the authors’ previous work demonstrating OXR1 playing a role in transcriptional regulation is understood following the authors’ response; however, this explanation should be added to the manuscript to explain why they investigated these networks further. Just a simple line stating “As our previous work has demonstrated a role for OXR1 in transcriptional regulation…” or something to that effect should be added to Line 183. It should not be assumed that all readers have read the authors’ previous work and thus would automatically understand the authors’ rationale.

**Authors’ response to reviewers**

Reviewer #1: In general, the manuscript has been improved. I still have a few comments regarding the conclusions and focus; the data tell a convincing story around one very defined mechanism in the context of the iPSC-derived cells utilized, but the wider disease relevance or explanation for many of the human clinical features (e.g. epilepsy, ataxia) is ignored or not convincing, in particular that only a single patient was used with a not well-matched control sample (and no isogenic control) and that they were not able to model cerebellar structures, the cardinal pathological region of OXR1 loss-of-function.

Authors: We appreciated the reviewer´s critical evaluation. Cerebellar ataxia and epilepsy are the key features of OXR1 deficiency. We agree that this study lacks direct evidence to convincingly explain the wider disease relevance to the human clinical features (e.g. epilepsy, ataxia). Unfortunately, we could not establish cerebellar organoids with the published protocols with our iPSC clones (both control and patient). It is a very rare disease, but luckily, we were able to establish several iPSC clones from one of the three patients carrying this mutation in the world. Although this study is limited due to the use of unrelated healthy iPS cells as control, we chose a female control to at least minimize gender affection. We agree that an isogenic control with the same genetic background is very important in iPS-derived disease modelling. Direct correction of OXR1 genetic mutation in patient iPS cells, using genome editing technology, is warranted in future studies.

>> Title
The name of the gene is incorrect (!) - it is 'Oxidation resistance 1' not 'Oxidation resistance gene 1'
The title also suggests the pathway has been studied in the human brain
I suggest:
A loss-of-function mutation in human Oxidation Resistance 1 disrupts the spatial-temporal regulation of histone arginine methylation in neurodevelopment

Authors: We have corrected the title accordingly.

>> Abstract
'very recently' should be removed (it was corrected in the main text)

Authors: ‘very recently’ is replaced by ‘in 2019’ in the abstract

The statement
'This study provides new insights into pathological features and molecular underpinnings associated with OXR1 deficiency in patients, highlighting the therapeutic potential of OXR1 in numerous neurodegenerative
and neurodevelopmental disorders.'

I suggest that the manuscript 'provides potential new insights into pathological features and molecular underpinnings associated with OXR1deficiency in patients', but it is not 'highlighting the therapeutic potential of OXR1 in numerous neurodegenerative and neurodevelopmental disorders.'

the second half of the sentence should be removed
(also in the Conclusion to the paper)

Authors: We have modified our statement according to the reviewer’s advice: "This study provides potential new insights into pathological features and molecular underpinnings associated with OXR1deficiency in patients." Both are corrected in the abstract part (lines 53-54) and the conclusion part (lines 489-490).

>> Table 2
I am still not convinced of the significance of the overlapping DEGs with other disorders from very different data sources warrants an addition to the paper; with this study finding many hundreds/thousands of
DEGs that are expressed in the CNS, the Table does not add any useful information. I disagree that the data presented provides insight into the role of OXR1 in other neurodevelopmental disorders.

Authors: We have removed our statement “suggesting a role of OXR1 in a broader spectrum of neurodevelopmental disorders”. But Table 2 is kept in the manuscript. It is still an interesting observation that DEGs in OXR1ΔEx18 neurons overlap with disease-associated genes in published databases.

>> Discussion page 17-18

The section on disease relevance is confusing, as the data on loss-of-function presented here are mixed with over-expression studies that follow a completely different set of molecular mechanisms and hypotheses. In my opinion this section should be removed completely. Also the OXR1-D mutation in SLI is mentioned, but there is no discussion of the relevance to the current study.

Authors: We have removed this part (lines 354-369).


>> Choice of control

I thank the authors for clarifying the control sample choice; it is becoming 'industry-standard' to provide isogenic controls when claiming the translational relevance of rare disease stem cell-derived modelling. In my opinion, a short section in the Discussion to address this is warranted. e.g. apart from sex, what variables related to ethnicity / age etc. might be influencing the study?

Authors: We agree with the reviewer that the current study is limited due to the use of unrelated healthy iPS cells as control. These control cells share some differences in age, genetic background, and other factors with the patient iPS cells. But we particularly chose a female control to minimize gender affection. In order to precisely generate human rare disease models, illustrate translational relevance, and develop new therapeutic approaches, an isogenic control with the same genetic background is very important in iPSC-derived disease modeling. For example, direct correction of OXR1 genetic mutation in patient iPS cells, using genome editing technology, is warranted in future studies. In fact, we did several attempts to generate OXR1 deficient human iPSC by CRISPR. However, we succeeded in a rat cell line presented in previous work by our group. We have added this information in the Discussion (lines 426-432).


Reviewer's Responses to Questions

**Please provide your comments for the author(s) on the revised manuscript.**


Reviewer #1: The combination of the two reviews of this manuscript covered a wide range of data content, re-analysis and discussion; the authors have done a reasonable job of addressing the concerns, including some new data.


Reviewer #2: Lin et al. have done well to address many of this reviewers concerns, especially with regards to describing the rationale for their approaches and explaining the probes used in the later figures. In particular, the explanations added for Figures 5 and 6 are vastly improved and greatly appreciated. There are, however, a few major and minor concerns that remain to be addressed.

Major:

- Regarding Figure 4b: The authors mention that the NSC-CTRL group is from a single biological replicate, and also mention in their own rebuttal that these are preliminary results. While the difference between these controls and the other groups are striking, and though they do show that other phenotypes in these controls are stable, a single replicate for any experiment is highly unorthodox. It is also not possible to perform robust statistical analyses between sample groups with only a single replicate. As this is a key part of the paper, this experiment must be repeated with multiple replicates.

Authors: We have generated new sets of NSCs adding another two control clones (total 3 control clones) and repeated most Western blot experiments to determine the epigenetic histone modifications during neurogenesis. The statistical analyses showed consistent results with previous data and confirmed impaired histone arginine/lysine methylations in OXR1-depleted NSCs (Fig. 4b and Fig. S4b).

- In their response to my concerns about Figure 4d (formerly 4c), the authors reasoned that increased PRMT5/MEP50 concentration contributed to elevated methylation despite no OXR1 added. But in the text, the authors state that this experiment suggests that OXR1 is essential for methylation signatures. These results seem to suggest that it is not required as long as PRMT5 and MEP50 are upregulated. This needs to be addressed and reasoned in the manuscript.

Authors: We have modified “suggesting the TLDc domain of OXR1 is essential for the regulation of PRMT-catalyzed H4R3 methylation” to “suggesting the TLDc domain of OXR1 may have a role in the regulation of PRMT-catalyzed H4R3 methylation” (Line 256).


- In the previous review, this reviewer had suggested that the images from Figure 5 should be quantified to better determine significance. This does not appear to have been addressed, as the authors’ response does not mention this point and the figures still lack quantification. Please quantify the signals that were being measured, particularly in Figures 5b-c and 5g-h. This is also true in some images in Figure 6, such as 6b. The images are convincing, but if the authors are claiming significant differences, quantification is necessary.

Authors: We quantified most images in Fig. 5 and Fig. 6,
In Fig. 5b, the distribution of TBR1+ fluorescent signal (magenta) and MAP2+ fluorescent signal (yellow) in control and OXR1ΔEx18 organoids at Day 75 is analyzed. The dot lines represent the average relative fluorescence intensity. The correlation analysis (Spearman) supports that the TBR1+ and MAP2+ layers are significantly separated in control organoids, but still intermingled at day 75 in OXR1ΔEx18 organoids as observed in Fig. 5b (CTRL: r = -0,8695 [95% CI, -0,8989 to -0,8323; p< 0,001]; OXR1ΔEx18: r = 0,1495 [95% CI, 0,01518 to 0,2785; p= 0,0249]). The quantification plots are updated in Fig. S5b and cited in the result part (line 272).

In Fig. 5c, the relative fluorescence intensity of REELIN at the superficial and inner layers is analysed, which shows the significant difference between control and OXR1ΔEx18 organoids (Student’s t-test, *, p < 0.05). The quantification plots are updated in Fig. S5c and cited in the result part (line 276).

In Fig. 5g, the percentage of SOX2+ cells in all DAPI-positive cells of hippocampus organoids at Day50 is quantified, which shows the significant difference between control and OXR1ΔEx18 organoids (Student’s t-test, ***, p < 0.001). The quantification plot is updated in Fig. S5e and cited in the result part (line 297).
.

In Fig. 5h, the percentage of NeuN+ neurons in the neuronal layers of hippocampus organoids (Day50) is quantified, which shows the significant difference between control and OXR1ΔEx18 organoids (Student’s t-test, ***, p < 0.001). The quantification plot is updated in Fig. S5f and cited in the result part (line 297).

In Fig. 6b, the percentage of SOX2+ and CTIP2+ cells in total DAPI-positive cells of cortical organoids at Day50 is quantified, which shows the significant difference between control and OXR1ΔEx18 organoids (Student’s t-test, **, p < 0.01; ***, p < 0.001). The quantification plots are updated in Fig. S6b and cited in the result part (line 303).
.

Minor:
- Figure 3 says “Figure 7” in the middle of it.

Authors: Corrected.

- In the previous draft, the organoid types were labeled in Figure 6 (e.g. “midbrain organoids” or “cortical organoids”). They do not appear in the most recent draft. Please put those labels back in to improve clarity in the figure.

Authors: Corrected.

- The point about the authors’ previous work demonstrating OXR1 playing a role in transcriptional regulation is understood following the authors’ response; however, this explanation should be added to the manuscript to explain why they investigated these networks further. Just a simple line stating “As our previous work has demonstrated a role for OXR1 in transcriptional regulation…” or something to that effect should be added to Line 183. It should not be assumed that all readers have read the authors’ previous work and thus would automatically understand the authors’ rationale.

Authors: The sentence ‘Previous work in our lab has demonstrated a role for OXR1 in transcriptional regulation (Ref. 1, 15)’ is added (Line 180).

**Third round of review**

**Reviewer 1**

no further comments

**Reviewer 2**

The authors have addressed all of my concerns. I appreciate their thoroughness in explaining the quantifications of their images as well as the inclusion of these explanations in the text.
